# The influence of smoking and alcohol on bone healing: Systematic review and meta-analysis of non-pathological fractures

**DOI:** 10.1016/j.eclinm.2021.101179

**Published:** 2021-10-31

**Authors:** Bin Xu, David B Anderson, Eun-Sun PARK, Lingxiao Chen, Jae Hyup Lee

**Affiliations:** a Department of Orthopedic Surgery, College of Medicine, Seoul National University, Seoul, Republic of Korea; b Department of Orthopedic Surgery, SMG-SNU Boramae Medical Center, Seoul, Republic of Korea; c Department of Orthopedic Surgery, Tianjin Hospital, Tianjin University, Tianjin, China; d Faculty of Medicine and Health, University of Sydney, Sydney, New South Wales, Australia; e Sydney Spine Institute, Sydney, New South Wales, Australia; f Medical Library, College of Medicine, Seoul National University, Seoul, Republic of Korea; g Institute of Bone and Joint Research, Kolling Institute, Sydney Medical School, Faculty of Medicine and Health, University of Sydney, Sydney, New South Wales, Australia; h Institute of Medical and Biological Engineering, Medical Research Center, Seoul National University, Seoul, Republic of Korea

**Keywords:** Smoking, Alcohol consumption, Bone healing, Wound healing, Meta-analysis

## Abstract

**Background:**

We aimed to comprehensively evaluate the associations between (i) smoking, (ii) preoperative smoking cessation time, (iii) nicotine replacement therapy (NRT), (iv) vaping, and (v) alcohol consumption and non-pathological fracture healing in adult patients. We also assessed the impacts of preoperative smoking cessation time, NRT, and vaping on wound healing and wound complications after any sort of surgery.

**Methods:**

We searched the MEDLINE, Embase, Cochrane CENTRAL, CINAHL, and AMED electronic databases from their inceptions until August 9th, 2021. Primary outcomes included delayed union rate, nonunion rate, and time to union. A random effects model was used. (Protocol registration: PROSPERO—CRD42019131454).

**Findings:**

One hundred and twenty-two studies with 417,767 patients were eligible for the systematic review and 71 of the studies with 39,920 patients were eligible for the meta-analysis. After non-pathological fracture treatment, the nonunion rate was significantly greater in the smoker group than in the non-smoker group (odds ratio [OR], 2·50, 95% confidence interval [1·73–3·61]); additionally, there was no significant difference in the nonunion rate (OR, 0·97 [0·40–2·38]) between the alcohol drinker group and the non-drinker group. The rate of wound infection after surgery was significantly reduced in the smoking cessation group (≥four weeks before surgery) compared to the continuous smoker group (OR, 0·37 [0·16–0·89]).

**Interpretation:**

Smoking is associated with higher rates of nonunion and deep surgical site infection after non-pathological fracture treatment. Smoking cessation (≥four weeks before surgery) is associated with a decreased rate of postoperative wound infection.

**Funding:**

The China Scholarship Council (no. 201809120013).

**Research in context**

**Evidence before this study**

The influence of smoking, preoperative smoking cessation time, nicotine replacement therapy (NRT), vaping, and alcohol consumption on non-pathological fracture healing is still unclear. Although previous meta-analyses have been conducted, they were limited by methodological limitations, including mixed populations within the control group (a combination of non-smokers and former smokers) and a mixture of outcomes (i.e., nonunion and delayed union, as well as a mixture of infection types).

**Added value of this study**

This study presents the largest assessment (122 studies with 417,767 subjects) of (1) the impact of smoking and alcohol consumption on non-pathological fracture healing and (2) the impact of preoperative smoking cessation time on wound complications after any sort of surgery. For non-pathological fracture healing, the results demonstrated that smokers had significantly higher rates of nonunion and deep surgical site infection than non-smokers. For wound complications after any sort of surgery, we found that, compared with continuous smokers, smoking cessation (four weeks or more before surgery) reduced postoperative wound infection.

**Implications of all the available evidence**

This meta-analysis provides further evidence that smoking is harmful to non-pathological fracture healing. However, additional evidence is needed to assess the potential dose-response relationship in relation to smoking consumption. From a policy perspective, smoking cessation should be emphasized in future clinical practice guidelines to reduce postoperative wound infection, wherein further research is needed to establish the minimum smoking cessation time.

Alt-text: Unlabelled box

## Introduction

Bone fractures will affect approximately 1 in 3 people in their lifetime.^1^ The annual incidence of traumatic fractures ranges from 0·25–0·38% per year.^2^^,^^3^ Of the fractures that occur annually, up to 4·9% remain unhealed, which are also known as non-union.^4^ Nonunion of a fracture site is associated with increased health costs and increased rates of premature death, compared with a fracture that heals within six months after treatment.^5^^,^^6^ In addition to operative and conservative treatments for fractures, preventative strategies are also recommended for improving the rates of fracture healing in various studies, including smoking cessation and reduced alcohol consumption.^7^^,^^8^ However, due to the lack of sufficient evidence, the guidelines for fracture management do not provide recommendations for the cessation or reduction of smoking and drinking.^9^, ^10^, ^11^, ^12^ Two previous meta-analyses^13^^,^^14^ have reported that smoking was associated with greater odds of nonunion following a fracture; however, these meta-analyses had several limitations, such as the inclusion of non-union without a description of the healing time period, the possibility that the control group included the exposure group, and limited details on the search strategy. Only one previous review concluded that alcohol consumption did not affect fracture healing rates; however, this study also had the same methodological limitations as the reviews on smoking.^14^ There is insufficient evidence on the impact of preoperative smoking cessation time, nicotine replacement therapy (NRT), and vaping on non-pathological fracture healing. Thus, it is necessary to perform an updated systematic review with the use of valid methods and a detailed analysis framework.

The aim of the current systematic review and meta-analysis was to comprehensively evaluate the associations between smoking, preoperative smoking cessation time, NRT, vaping, and alcohol consumption and non-pathological fracture healing in adult patients.

## Methods

### The protocol and the deviation between the final study and the protocol

The protocol of this meta-analysis was submitted and published with PROSPERO (international prospective register of systematic reviews) (registration number: CRD42019131454) on May 22nd, 2019. A detailed protocol for this study has also been previously published (**Appendix S1**).^15^ The deviation between the final study and the protocol is described as follows:

(1) For the study design, case series were additionally included because very few studies that directly investigated the influences of smoking or alcohol consumption existed, as well as for comprehensive searching.

(2) Exposure groups were changed from current smokers and current alcohol consumers to current or former smokers and current or former alcohol consumers because the exposure groups in most of the available studies included former smokers or drinkers. Definitions of exposure were changed to definitions developed by the Centers for Disease Control and Prevention (CDC) of the US because of authority and reliability.^16^, ^17^, ^18^

(3) For the outcomes, according to actual data extraction from the included studies, we added normal union because (1) we found that although the definition of the time period of delayed union or nonunion was inaccurate in several studies, those studies recorded accurate normal healing outcomes, and (2) there is still a lack of data concerning the effects of smoking and drinking on normal healing.

The time period of nonunion was changed from over 9 months to over 6 months to unify the definitions of the outcomes because we found that the time period (6–9 months) between delayed union (3–6 months) and nonunion (over 9 months) was not clearly classified in Campbell's Operative Orthopaedics 13th edition.

We divided wound infection into superficial infection, deep infection, and undefined infection. In several studies, infection was described with unclear definitions or without definitions. The definitions of superficial infections and deep infections are described in the Introduction section, rather than in the Methods section. This made it impossible for us to be sure as to which type of infection is referred to in the studies. These infections were defined as being undefined infections.

(4) A dose-related meta-analysis was performed by using a traditional meta-analysis, instead of a dose-response meta-analysis, because very few studies have recorded doses of smoking or alcohol drinking, thus making a dose-response meta-analysis unable to be conducted.

(5) Factors that may affect fracture healing, such as gender, age, and diabetes were assessed via subgroup analyses, instead of via meta-regression analyses.

(6) Studies with (1) multiple concurrent fractures, (2) mandible or spine fractures, (3) case series, (4) no description of inclusions or exclusions of non-pathological fractures, and (5) non-operative treatments, a mixing of operative and non-operative treatments, or unknown treatments were additionally analysed as to whether they affect the robustness of results via a sensitivity analysis.

(7) The grading of the quality of evidence by using the Grading of Recommendations Assessment, Development and Evaluation (GRADE) system was not performed because the overall risk of bias that was needed in the grading process could not be developed by using the Quality In Prognosis Studies (QUIPS) tool, according to the recommendation of the QUIPS tool.

(8) The effect size for the dichotomous data was changed from risk ratios (RRs) to odds ratios (ORs) to unify the effect size.

(9) The effects of preoperative smoking cessation time, nicotine replacement therapy (NRT), and vaping on fracture healing, postoperative surgical site infection, and malunion after non-pathological fracture treatments were also investigated.

(10) The effects of preoperative smoking cessation time, NRT, and vaping on bone healing, wound healing, and wound complications after surgery were also investigated.

**Fig. 1:**
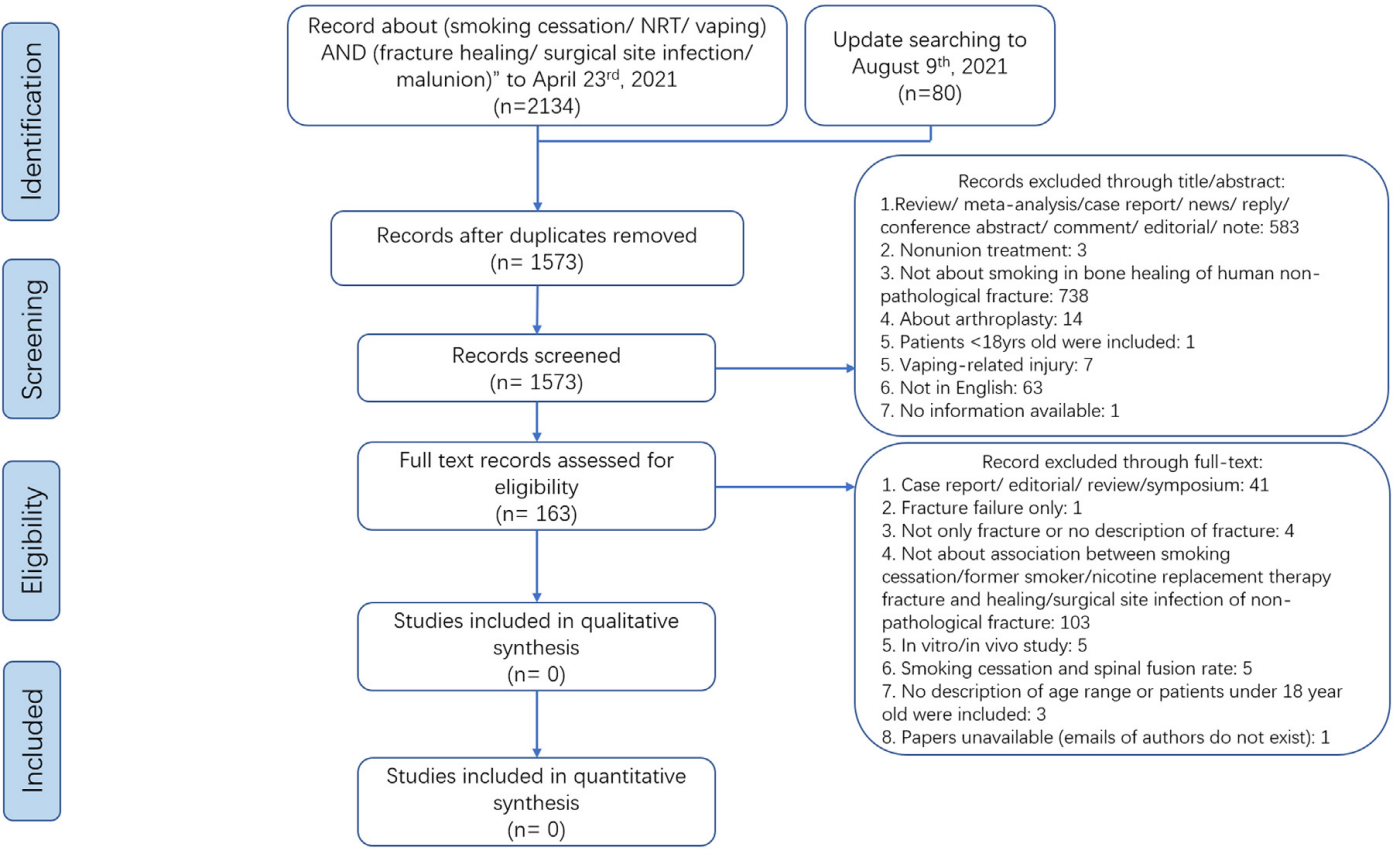
PRISMA flow chart of study identification, screening, and selection for the impact of preoperative smoking cessation, nicotine replacement therapy, and vaping on fracture healing, surgical site infection and malunion.

Through a comprehensive search, we found no studies concerning the impact of preoperative smoking cessation time, NRT, or vaping on fracture healing that met the inclusion and exclusion criteria (Fig. 1). Thus, we expanded the subjects and outcomes of the research as follows: (1) for bone healing, patients undergoing spinal fusion, arthrodesis, or osteotomy were included; and (2) for postoperative wound healing and wound complications, patients undergoing any surgery were included. This systematic review and meta-analysis was conducted according to the Meta-analysis of Observational Studies in Epidemiology (MOOSE) and Preferred Reporting Items for Systematic Reviews and Meta-Analysis (PRISMA) reporting guidelines.^19^^,^^20^ The anonymous and public data from previous published studies was retrieved and analysed and the need for ethics approval and patient informed consent was therefore waived.

### Search strategy

A systematic search was conducted by a librarian by using the MEDLINE (via OvidSP), EMBASE, Cochrane Central Register of Controlled Trials (CENTRAL), CINAHL (via Ebsco), and AMED (via OvidSP) databases from the time of their inceptions until August 9th, 2021. A detailed search strategy is available in **Appendices S2.1.1a to e** through **S2.4.2a to e**. We also performed a manual search of the reference lists of the included studies.

### Study selection and selection criteria

Two authors screened all of the studies by titles, abstracts, and full text. Any disagreements between the two screening authors were resolved by a third author. The inclusion and exclusion criteria are described as follows:

(1) For the impacts of smoking and alcohol consumption on fracture healing, studies were included in this study if they met the following criteria:

(i) Inclusion of a comparison between either smokers (current smokers or former smokers) vs. non-smokers or alcohol drinkers (current alcohol consumers or former alcohol consumers) vs. non-drinkers.

(ii) Individuals≥18 years.

(iii) Diagnoses of a bone fracture (any location) based on surgical records, radiographic data, databases, electronic medical records, or the AO/OTA fracture classification.

(iv) Studies that were randomised controlled trials (RCTs), observational studies, or case series.

(v) Studies that were written in the English language.

Studies were excluded if they met any of the following criteria:

(i) ≥20% of the fractures were attributed to a pathological cause, such as cancer, renal disease, human immunodeficiency virus, or osteoporosis.

(ii) Patients who were initially treated with arthroplasty or amputation because fracture areas were removed.

(2) For the impacts of preoperative smoking cessation time, NRT, and vaping on bone healing, the inclusion criteria were as follows:

(i) Patients who underwent spinal fusion, arthrodesis, or osteotomy.

(ii) NRT or the cessation of nicotine use was compared with continuous smokers or individuals who had never smoked.

(iii) Studies that were RCTs or observational studies.

(iv) Age not being restricted.

(v) Studies written in the English language.

Studies were excluded if smoking cessation time was not clearly described or if smoking was not stopped within the study duration.

(3) For the impacts of preoperative smoking cessation time, NRT, and vaping on wound healing and wound complications after surgery, the inclusion criteria were as follows:

(i) Patients who underwent any surgical procedure.

Inclusion criteria (ii)–(v) and the exclusion criteria were the same as in (2).

### Exposures

According to the CDC in the US, current smokers were defined as those individuals who smoked during the study period. Former smokers were those people who had smoked ≥100 cigarettes in their lifetime but who had stopped smoking during the study period.^16^ Non-smokers were defined as those people who had never smoked or who had smoked <100 cigarettes during their lifetime.^16^ Current drinkers were defined as participants with alcohol consumption in the past year.^17^^,^^18^ Former drinkers were those individuals who had consumed alcohol in any one year but who had consumed nothing within the past year.^17^^,^^18^ Non-drinkers were participants who had consumed alcohol <12 times during their lifetime.^17^^,^^18^ Smoking cessation was defined as the cessation of smoking for one day or longer with the intention of quitting.^16^ NRT was defined as the cessation of smoking medications that provided a small amount of nicotine but no other dangerous chemicals that are found in cigarettes. Types of NRT included patches, chewing gum, lozenge, inhaler, and nasal spray.^21^ Vaping was defined as the use of an e-cigarette that produces an aerosol by heating a liquid containing nicotine.^22^ E-cigarettes are also known as e-cigs, e-hookahs, vapes, and electronic nicotine delivery systems (ENDS), etc.

### Study outcomes

The primary outcomes included the rate of delayed union, rate of nonunion, and time to union. When considering that there were no available studies concerning the impacts of preoperative smoking cessation time, NRT, or vaping on non-pathological fracture healing, as well as the fact that we expanded the subjects and outcomes of the research, the secondary outcomes included (1) rates of common postoperative complications, including superficial surgical site infection (SSSI), deep surgical site infection (DSSI), undefined surgical site infection (SSI), malunion, and normal union for smoking and alcohol consumption; and (2) rates of nonunion of bone, impaired wound healing, and wound complications for preoperative smoking cessation time, NRT, and vaping. The definitions of normal union, delayed union, and nonunion were described as follows: a fracture that healed within three months (normal union), a fracture that healed between three and six months (delayed union), and a fracture that had not healed by at least six months (nonunion).^23^ When both delayed union and nonunion were recorded, we calculated normal union data by subtracting the prior two from the total. Time to union was defined as the time period from treatment to the time point when a radiographic bridging callus was present. Malunion was defined as any fractures that were not found to be anatomically healed on imaging.^24^ SSSI, DSSI, and undefined SSI were defined as infections that occurred only on the skin and in the subcutaneous tissue of the incision, infections in the deep soft tissues of the incision, and infections that were not clearly classified as being superficial or deep SSI, respectively.^25^

### Data extraction and quality assessment

Two authors independently extracted the following information: (1) characteristics of the studies, including author, publication year, journal name, country, study design, inclusion and exclusion criteria, study group, and follow-up period; (2) characteristics of the patients, including sex, age, race, body mass index (BMI), height, and weight; (3) fracture healing related factors, including non-pathological fractures or unclear, fracture location, methods of fracture treatment, concurrent fracture at other sites, the presence of risk factors to bone healing (i.e., diabetes), uses of nonsteroidal anti-inflammatory drugs (NSAIDs), and uses of the fluoroquinolone family of antibiotics; (4) exposure information, including smoking type, smoking dose, alcohol type, and level of alcohol consumption; and (5) data of various outcomes, including delayed union, nonunion, time to union, SSSI, DSSI, undefined SSI, malunion, and normal union. For smoking cessation time, NRT, and vaping, information on diabetes, the use of NSAIDs, the use of the fluoroquinolone family of antibiotics, smoking cessation procedure, the use of NRT, types of surgery, and definitions of outcomes was extracted.

### Assessment of study quality

The risk of bias of the included studies (smoking and alcohol consumption only) was independently assessed by two authors by using the QUIPS tool, based on the following six domains: (1) study participation domain (representativeness of the study sample); (2) study attrition domain (whether participants with follow-up data represented people who were included in the study); (3) prognostic factor measurement domain (adequacy of prognostic factor measurement); (4) outcomes measurement domain (adequacy of outcome measurement); (5) study confounding domain (potential confounding factors); (6) statistical analysis and reporting domain (the appropriateness of the statistical analysis and completeness of reporting of the study).^26^ Each of the six domains of the QUIPS tool was evaluated and rated as exhibiting either low, moderate, or high risks of bias.

### Statistics

Meta-analyses were performed by using a random-effects model with the inverse variance method for the continuous outcomes and the Mantel-Haenszel method for the dichotomous outcomes.^27^ When studies provided effect estimates and *P* values without the number of events, we used the inverse variance method.^27^ The effect size was evaluated by using the mean difference (MD) with 95% confident intervals (CIs) for the continuous outcomes and ORs with 95% CIs for the dichotomous outcomes.^27^ According to the protocol, we planned to assess the dose-response relationship between smoking, alcohol consumption, and outcomes. However, the data were insufficient to perform a dose-response meta-analysis.^28^ Therefore, we conducted a traditional meta-analysis stratified by dose level. We also performed prespecified and post-hoc extensive subgroup analyses as follows: (1) female percentage <50% vs. ≥50%; (2) mean age <60 years old vs. ≥60 years old; (3) BMI <25 vs. 25–30 vs. >30; (4) diabetes percentage <20% vs. ≥20%; (5) sample size ≤100 vs. 101–300 vs. 301–500 vs. 501–700 vs. 701–1000 vs. >1000; (6) at least one confounder adjusted vs. not adjusted; and (7) RCT vs. cohort study vs. case-control study. Prespecified and post-hoc extensive sensitivity analyses were performed to confirm whether the following factors affected the results of the meta-analysis: (1) studies without funding assistance; (2) studies with a low risk of bias; (3) studies without mandible and spinal fractures; (4) studies with fractures in a single location; (5) studies without case series; (6) studies with a clear description of the inclusion of non-pathological fractures; (7) studies with operative treatment; (8) removing one study at a time for outcomes with high heterogeneity; and (9) removing three studies with high ORs for smoking and nonunion as the reviewer suggested.

Statistical heterogeneity was assessed via the I^2^ value.^29^ We considered a I^2^ value greater than 75% to a high degree of heterogeneity. We evaluated publication bias by visually assessing the symmetry of funnel plots and via the Rucker's test^30^^,^^31^, which was performed by using R version 4.0.3 (R Foundation for Statistical Computing), to quantify symmetry. All of the analyses (except for the Rucker's test) were performed by using Review Manager (RevMan) Version 5.3 (Copenhagen: The Nordic Cochrane centre, The Cochrane Collaboration, 2014).

### Role of funding sources

The funding source of this study had no role in the design and conduct of the study, data analysis, data interpretation, or writing.

## Results

### Search results

**Fig. 2:**
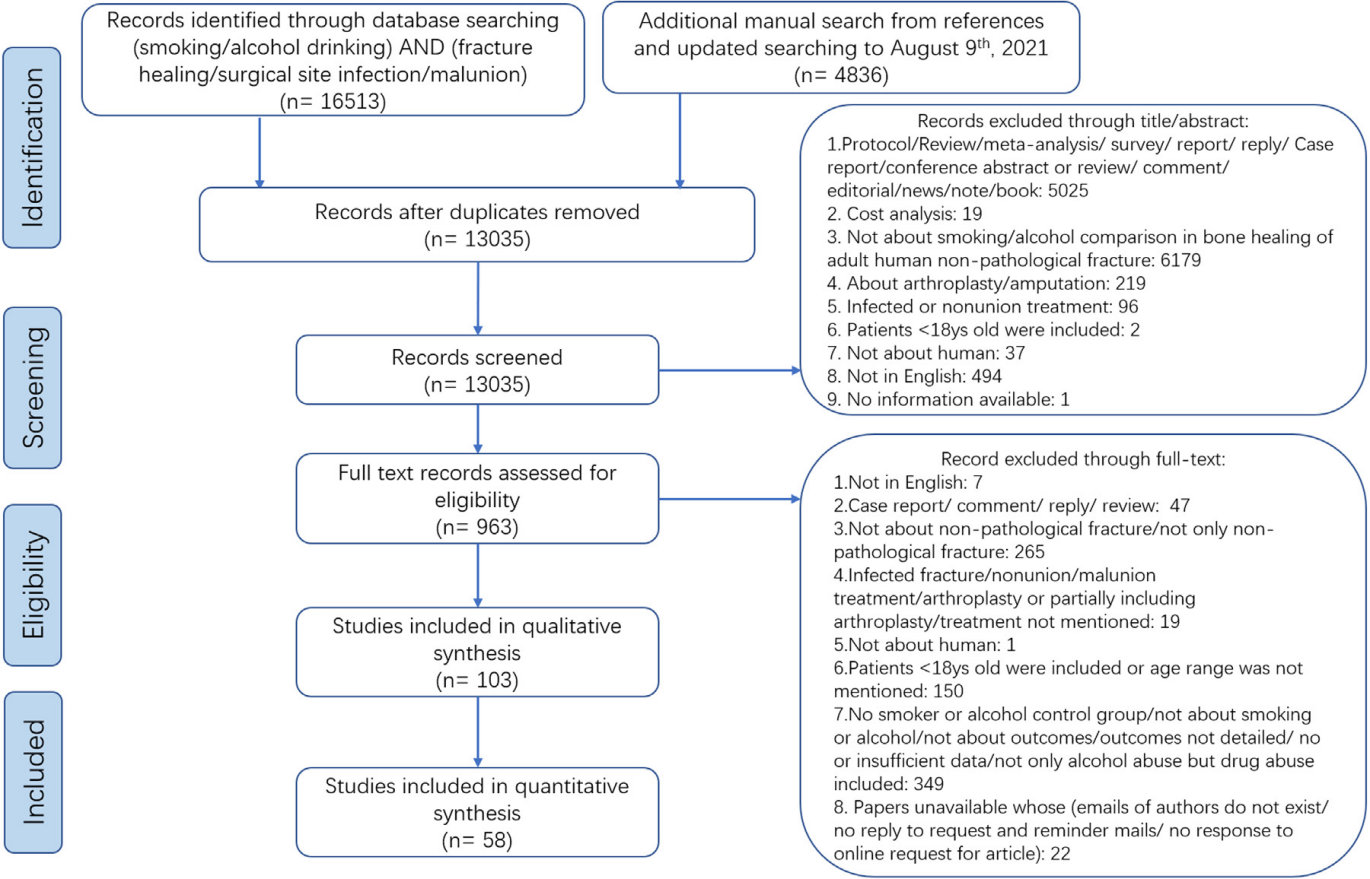
PRISMA flow chart of study identification, screening, and selection for the impact of smoking and alcohol consumption on fracture healing, surgical site infection and malunion.

One hundred and twenty-two studies were included in the qualitative analysis, with 71 studies being eligible for the quantitative synthesis (Fig. 2, **Appendices S3a and S3b**). The search results are described in detail in **Appendices S4a and S4b**. A summary of the characteristics of the included studies is shown in Table 1 and Table 2, and the detailed information is described in **Appendices S5a to d, Appendices S6a to d, and Appendices S7a to d**. The majority of the studies were conducted in North America (*n* = 45, 37%), with the other studies originating from Europe (*n* = 43, 35%), Asia (*n* = 33, 27%), and Africa (*n* = 1, 1%). The mean age of the participants from the included studies was under 60 years of age in 84% of the studies. Thirty-four percent of the studies had sample sizes of less than or equal to 100 patients, and 29% of the studies had sample sizes ranging between 101 and 300 patients.

**Table 1:** Characteristics of 122 included studies.

**Characteristics**	**No (%) of studies**
Publication year	
1971–1980	1 (1)
1981–1990	0 (0)
1991–2000	6 (5)
2001–2010	23 (19)
2011–2021	92 (75)
Follow-up period (months)	
0–12	33 (27)
13–24	22 (18)
25–36	5 (4)
37–48	6 (5)
49–60	4 (3)
>60	1 (1)
Not reported or not detailed	39 (32)
Without follow-up	12 (10)
Geographical region	
Asia	33 (27)
Europe	43 (35)
North America	45 (37)
Africa	1 (1)
Age group (years)	
<60	103 (84)
≥60	11 (9)
Not reported	8 (7)
Proportion female (%)	
<50	80 (66)
≥50	31 (25)
Not reported	11 (9)
BMI (kg/m^2^)	
<25	17 (14)
25–30	23 (19)
>30	6 (5)
Not reported	76 (62)
History of diabetes (%)	
<20	71 (58)
≥20	6 (5)
Not reported	45 (37)
Sample size	
≤100	42 (34)
101–300	35 (29)
301–500	10 (8)
501–700	11 (9)
701–1000	10 (8)
>1000	14 (11)

Note: BMI: body mass index.

**Table 2:** Characteristics of 122 included studies-continued.

**Characteristics**	**No (%) of studies**
Outcomes	
Smoking and alcohol consumption	
Delayed union	8 (7)
Nonunion	39 (32)
Normal union	25 (20)
Time to union	10 (8)
Superficial infection	6 (5)
Deep infection	27 (22)
Undefined infection	35 (29)
Malunion	2 (2)
Preoperative smoking cessation time, NRT, and vaping	
Spinal fusion nonunion	1 (1)
Wound infection	13 (11)
Hematoma	3 (2)
Impaired wound healing	2 (2)
Wound dehiscence	1 (1)
Wound rupture	1 (1)
Seroma	2 (2)
Wound diameter, depth	1 (1)
Mixed wound complication (infection and dehiscence)	1 (1)
Wound healing problem (dehiscence, fat necrosis, infection)	1 (1)
Non-pathological fracture or unclear	
Non-pathological fracture	40 (39)
Unclear	63 (61)
Fracture location	
Mandible	3 (2)
Spine	3 (2)
Clavicle	6 (5)
Humerus	8 (7)
Pelvis	1 (1)
Femur	9 (7)
Tibia	30 (25)
patella	1 (1)
Ankle	14 (11)
Calcaneus	9 (7)
Metatarsal	1 (1)
Elbow	1 (1)
Hip	1 (1)
Acetabulum	1 (1)
Radius and Ulna	1 (1)
Metacarpal and phalanx	1 (1)
Other mixed locations	13 (11)

Note: NRT: nicotine replacement therapy.

The results of the risk of bias for the 58 studies that were included in the meta-analyses of the impact of smoking and alcohol consumption are shown in **Appendix S8**.

### Delayed union

**Fig. 3:**
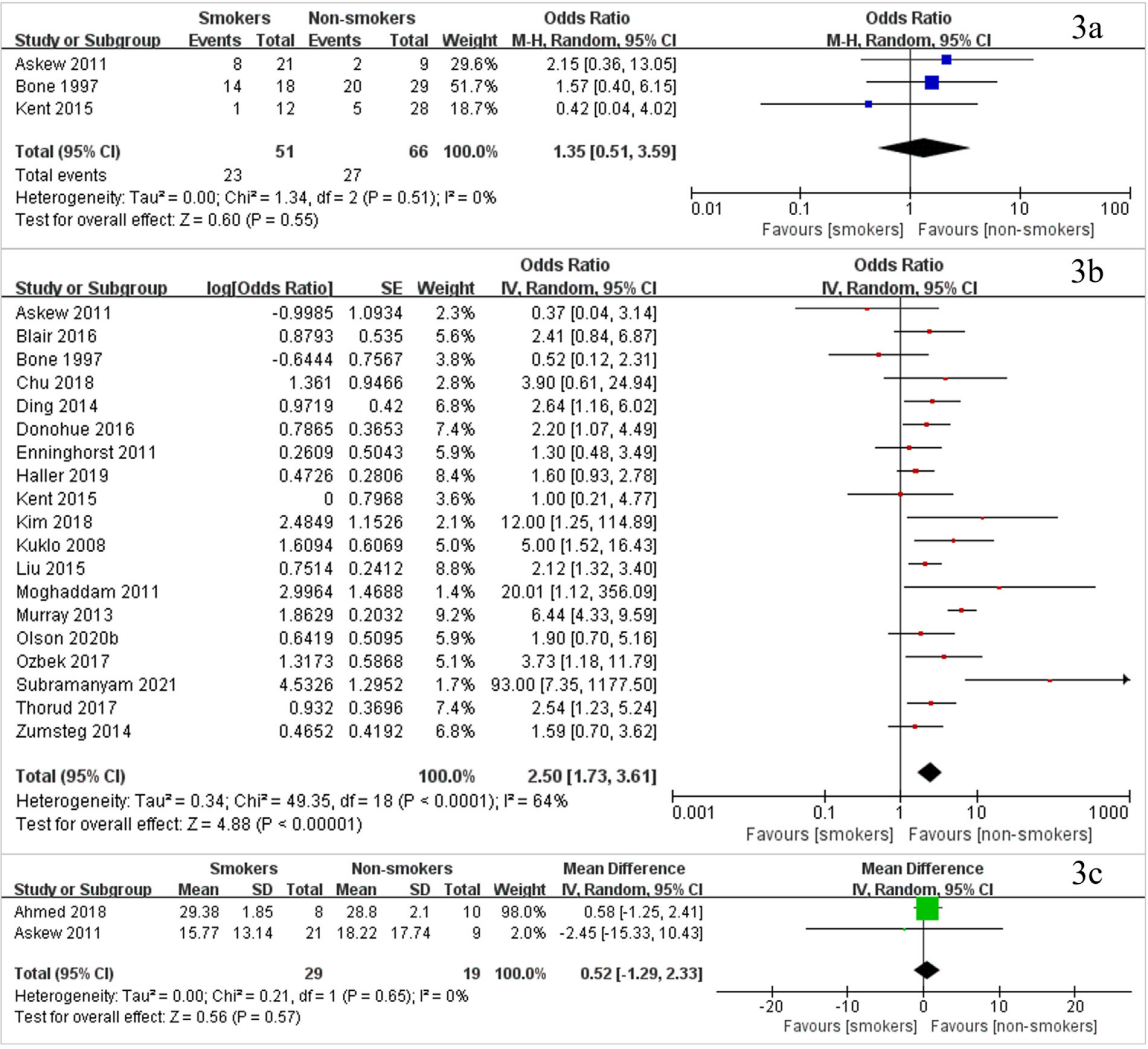
Forest plots of meta-analysis of (3a) delayed union rate, (3b) nonunion rate, and (3c) time to union of smokers vs. non-smokers.

Three studies including 117 patients investigated the delayed union rate between smokers and non-smokers (Fig. 3a, with the study list found in **Appendix S9**) and found no significant difference between smokers and non-smokers (OR 1·35, 95% CI [0·51–3·59], *P* = 0·55, I^2^=0%). There were no studies available that compared delayed union rates between patients who did or did not consume alcohol.

### Nonunion

Nineteen studies including 4726 patients compared data on the nonunion of smokers with non-smokers (Fig. 3b, with the study list found in **Appendix S9,** and the data found in **Appendix S10**). The results showed that the nonunion rate in the smoker group was significantly increased compared with that in the non-smoker group (OR 2·50, 95% CI [1·73–3·61], *P*<0·0001, I^2^=64%). Three studies including 1607 people compared nonunion between alcohol drinkers vs. non-drinkers (**Appendix S11,** with the study list found in **Appendix S9**) and found no significant difference in the rates of non-union (OR 0·97, 95% CI [0·40–2·38], *P* = 0·95, I^2^=77%).

### Time to union

Two studies including 48 patients investigated the time to union between smokers and non-smokers (Fig. 3c, with the study list found in **Appendix S9**), with no significant difference being found between the two groups (mean difference [MD] 0·52 weeks, 95% CI [-1·29–2·33], *P* = 0·57, I^2^=0%). Only one study including 29 patients compared the time to union between alcohol drinkers and non-drinkers and found that the time to union was longer for alcohol drinkers (MD 12·85 weeks, 95% CI [1·23–24·47], *P* = 0·03, I^2^=NA).

### Secondary outcomes

**Fig. 4:**
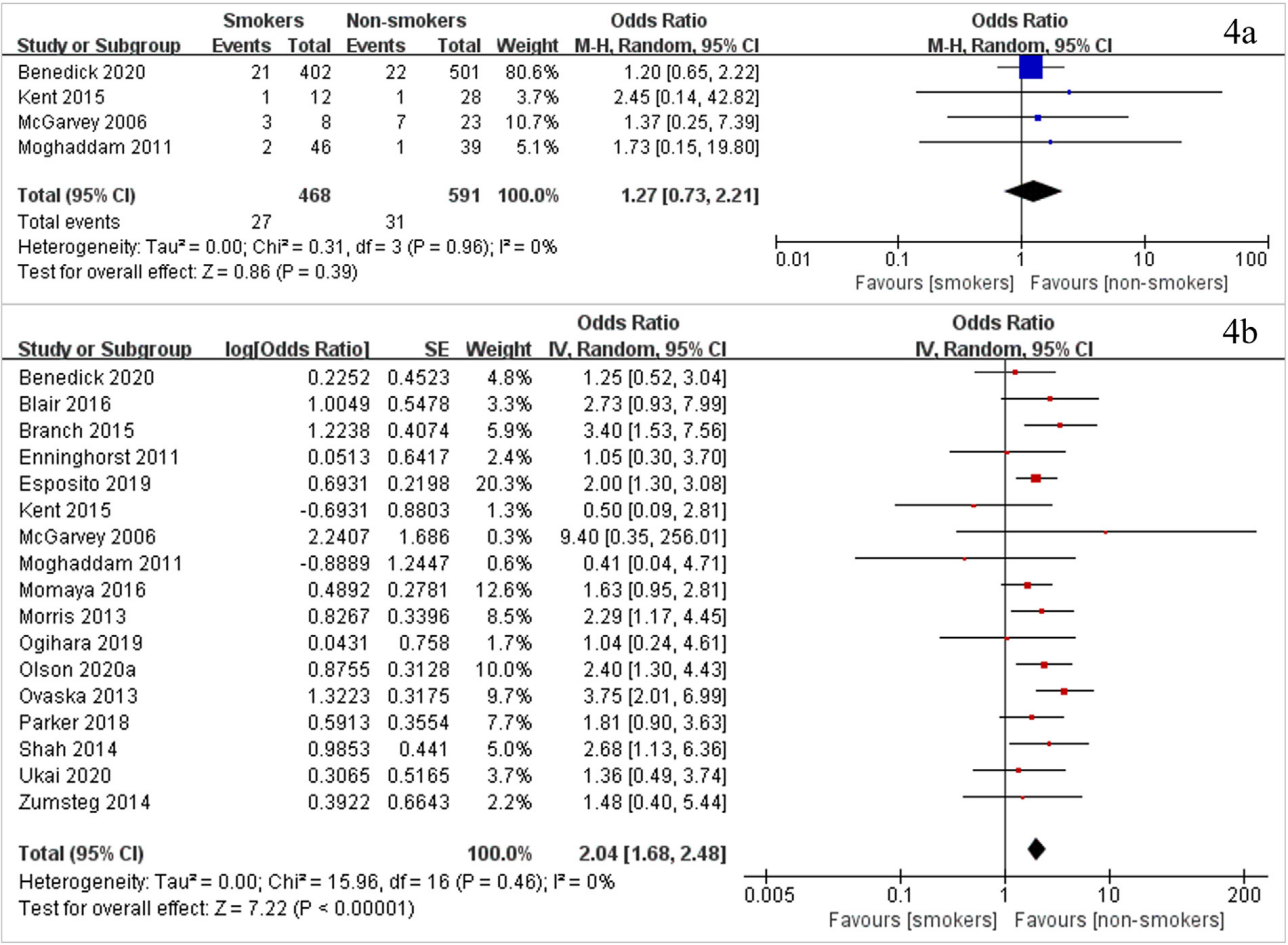
Forest plots of meta-analysis of (4a) superficial surgical site infection rate and (4b) deep surgical site infection rate of smokers vs. non-smokers.

The results of the secondary outcomes are shown in Fig. 4, as well as **Appendices S12** and **S13**. The results of seventeen studies with 14,365 patients showed that a significantly larger incidence of deep surgical site infection (DSSI) was present in smokers than in non-smokers (OR 2·04, 95% CI [1·68–2·48], *P*<0·0001, I^2^=0%) (Fig. 4b). Eighteen studies with 9226 patients and one study that had effect size data (but no number of smokers and non-smokers) investigated undefined surgical site infection (SSI). A significantly larger rate of undefined SSI was found in smokers than in non-smokers (OR 3·11, 95% CI [2·09–4·63], *P*<0·0001, I^2^=66%) (**Appendix S12a**). Four studies including 1059 patients compared the superficial surgical site infection (SSSI) rate (Fig. 4a), and five studies with 435 patients compared the normal union rate (**Appendix S12b**) between smokers and non-smokers. However, no significant difference was found between smokers and non-smokers (for SSSI, OR 1·27, 95% CI [0·73–2·21], *P* = 0·39, I^2^=0%; for normal union, OR 1·60, 95% CI [0·78–3·26], *P* = 0·20, I^2^=0%).

For the impact of alcohol consumption on the DSSI rate, two studies including 3293 participants showed no statistically significant difference between alcohol drinkers and non-drinkers (OR 1·02, 95% CI [0·64–1·62], *P* = 0·94, I^2^=0%) (**Appendix S13a**). Seven studies including 3571 participants and two studies that had effect size data (but unknown sample sizes) showed that undefined SSI rate was not significantly different between the alcohol drinker group and non-drinker group (OR 1·28, 95% CI [0·81–2·03], *P* = 0·29, I^2^=60%) (**Appendix S13b**). A meta-analysis on malunion could not be conducted because of insufficient data.

**Fig. 5:**
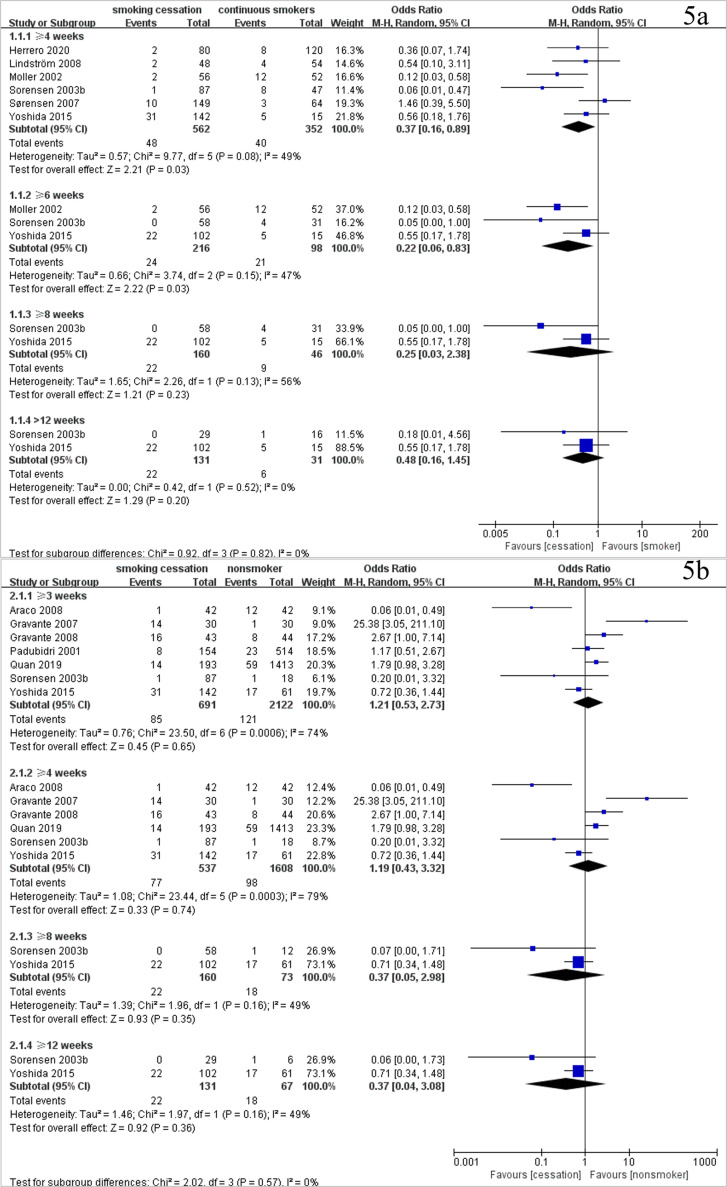
Forest plots of meta-analysis of (5a) wound infection rate of smoking cessation vs. continuous smokers and (5b) wound infection rate of smoking cessation vs. non-smokers.

**Fig. 6:**
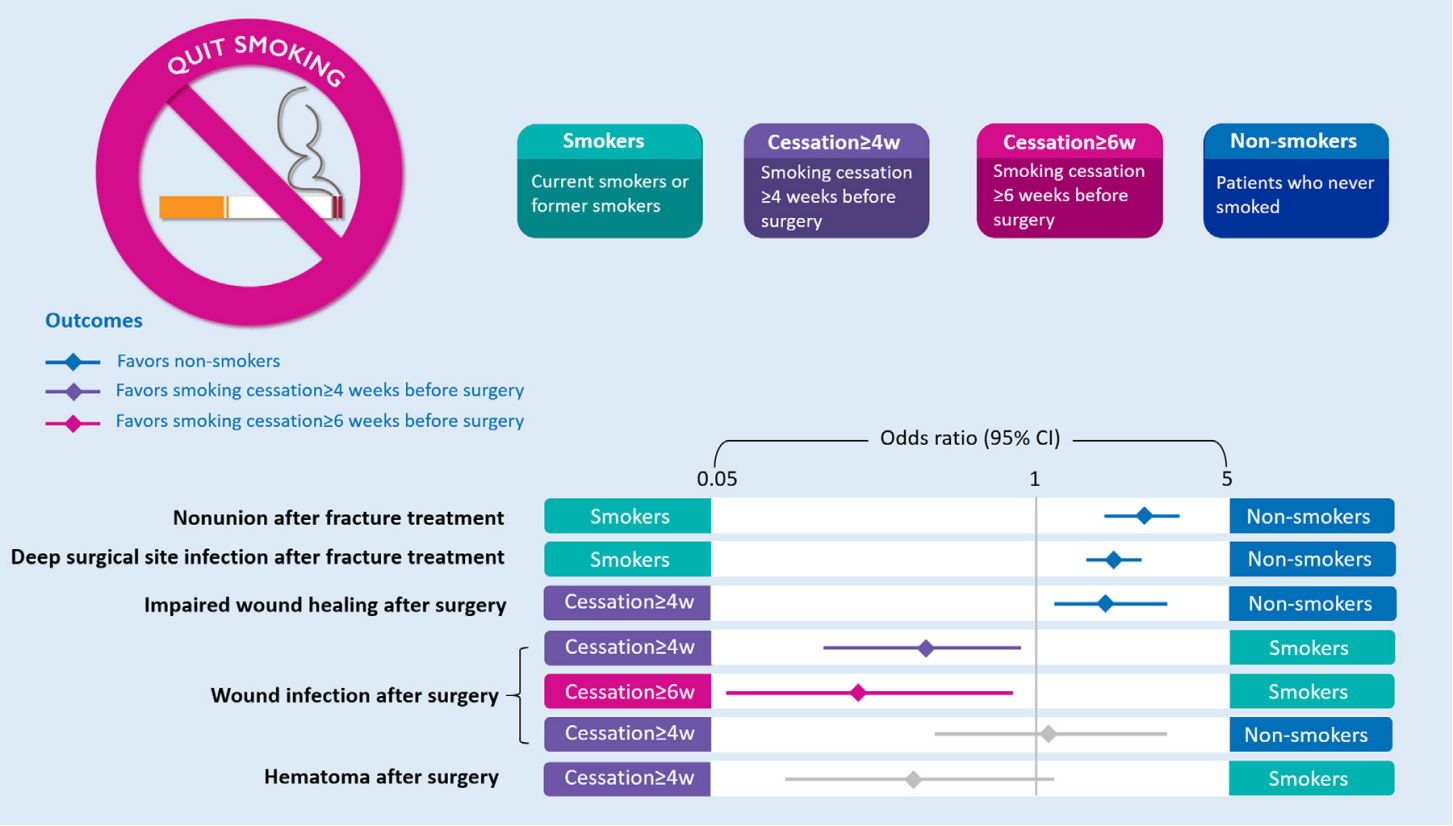
Summary of the impact of smoking and preoperative smoking cessation time.

For wound infection, six studies with 914 patients showed that smoking cessation for four weeks or more (OR 0·37, 95% CI [0·16–0·89], *P* = 0·03, I^2^=49%) and six weeks or more (OR 0·22, 95% CI [0·06–0·83], *P* = 0·03, I^2^=47%) before surgery significantly reduced wound infection rates postsurgery compared with participants in the continuous smokers group (Fig. 5a). However, no significant difference was found when preoperative smoking cessation for four weeks or more (OR 1·19, 95% CI [0·43–3·32], *P* = 0·74, I^2^=79%) was compared with the non-smokers group (Fig. 5b). For wound healing, two studies including 293 patients showed that the impaired wound healing rate was significantly increased in smoking cessation for four weeks or more before surgery (OR 1·93, 95% CI [1·17–3·20], *P* = 0·01, I^2^=0%), compared with non-smokers (study list found in **Appendix S14a** and **Appendix S15a**). In addition, the results of two studies with 210 patients showed that no significant difference was found in the hematoma rates between smoking cessation for four weeks or more before surgery and continuous smokers (OR 0·36, 95% CI [0·11–1·20], *P* = 0·10, I^2^=0%) (study list found in **Appendix S14a** and **Appendix S15b**). A summary of the impacts of smoking and preoperative smoking cessation time is shown in Fig. 6. No studies concerning NRT and vaping could be included in the meta-analysis (**Appendix S14b**).

### Publication bias

For comparisons of nonunion, DSSI, and undefined SSI between smokers and non-smokers, the results of the funnel plots (**Appendices S16a to c**) and Rucker's test (**Appendix S16d**) indicated that there was no obvious publication bias.

### Additional analyses

The results of the dose related meta-analysis **(Appendix S17)**, subgroup analysis (**Appendix S18**), and sensitivity analysis (**Appendix S19**) are shown in appendix, respectively.

## Discussion

Our study included 103 studies determining the impact of smoking or alcohol consumption on rates of bone healing and infection following a non-pathological fracture, and included 19 studies assessing the impact of preoperative smoking cessation time, NRT, or vaping on wound healing and wound complications after any sort of surgery. Eighty-one (79%) of the 103 studies included fractures that were treated with surgical fixation. The study found that smokers had significantly higher rates of nonunion, DSSI, and undefined SSI than non-smokers. When regarding alcohol consumption, there was insufficient evidence concerning the impact of alcohol consumption on fracture healing. In addition, DSSI and undefined SSI significantly increased among male smokers, but not among female smokers. One possible explanation is that male gender is a risk factor of developing a major infection after trauma as one previous study based on 545 trauma population from the US reported that male gender had a 58% greater risk of developing a major infection compared with female gender (OR 1·58, 95% CI [1·01–2·48], *P* = 0·04).^32^ Regarding the impacts of smoking and alcohol consumption on other outcomes, no significant gender differences were found. The study also found that the cessation of smoking for four weeks or more had no effect on bone healing; however, the odds of postoperative wound infection increased 2·70-fold in continuous smokers, compared with participants who had quit smoking for four weeks or more before surgery. The effect of preoperative smoking cessation for four weeks or more on postoperative wound infection was similar to that of non-smokers. However, the odds of impaired wound healing increased 1·93-fold in patients who had quit smoking for four weeks or more, compared to non-smokers.

Regarding the impact of smoking on nonunion, the effect size of the results in the current study (OR 2·50, 95% CI [1·73–3·61], *P*<0·0001) was greater than that of the previous two studies^13^^,^^14^ (OR 1·67, 95% CI [1·43–1·95], *P*<0·0001 and OR 1·69, 95% CI [1·46–1·96], *P*<0·05, respectively). This may be due to the following two reasons: (1) due to comprehensive search, our study included more studies (19 studies) than the two previous studies (14 studies were included in Pearson 2016 [13] and eight studies were included in Tian 2020 [14]) and (2) due to rigorous inclusion and exclusion criteria, many studies included in the previous studies have been excluded from this study (e.g. the definition of nonunion lacked a description of the time period in Adams 2001, OR 1·32, 95% CI [0·91–1.93] and the age range of included patients was not described in Lack 2014, OR 0.82, 95% CI [0·29–2·34]). Our study found that smoking was not associated with delayed union (OR 1·35, 95% CI [0·51–3·59], *P* = 0·55) or normal union (OR 1·60, 95% CI [0·78–3·26], *P* = 0·20), which indicates that smoking may not increase the delayed union rate or decrease the normal union rate. The contradiction between this result and the nonunion result may be due to the small sample sizes that were included in the analysis of delayed union (three studies with 117 patients) and normal union (five studies with 435 patients). For SSI, when compared with two previous studies, the likelihood of DSSI (OR 2·04, 95% CI [1·68–2·48], *P*<0·0001) and undefined SSI (OR 3·11, 95% CI [2·09–4·63], *P*<0·0001) in the current study was similar to the postoperative infection observed in Kortram et al. [33] (risk ratio [RR] 1·29, 95% CI [1·02–1·64], *P* = 0·04). Although SSI was different (OR 1·63, 95% CI [0·95–2·79], *P* = 0·08) from our study, this may be due to an identification error of the included studies in their study.^34^ Moreover, Shao et al. [34] synthesized only five of the seven studies concerning smoking and infection and did not describe what the five studies were. After we recalculated the results from all of the available data, we found that smoking might increase the infection rate (OR 2·12, 95% CI [1·11–4·02], *P* = 0·02, I^2^=88%) (**Appendix S20d**). However, readers need to be cautious when interpreting this result with high heterogeneity present. Problems in the included studies in previous studies about impact of smoking were described in **Appendices S20a to c, S20e, and S20f**. We additionally evaluated SSSI and found no significant difference between smokers and non-smokers (OR 1·27, 95% CI [0·73–2·21], *P* = 0·39).

Alcohol consumption did not significantly affect nonunion of fracture in the current study (OR 0·97, 95% CI [0·40–2·38], *P* = 0·95), which was similar to Tian et al. [14] (OR 1·37, 95% CI [0·96–1·95], *P* = 0·08). We also evaluated the time to union and found that drinking had an adverse influence on fracture healing time (MD 12·85 weeks, 95% CI [1·23–24·47], *P* = 0·03). Problems in the included studies in previous studies about impact of alcohol consumption were described in **Appendices S21a, S21b, and S21d**. For SSI, Shao et al. [34] may have omitted two studies that met the inclusion and exclusion criteria during data synthesis. Also for SSI, one of the studies included in Kortram et al. [33] incorrectly defined the outcome, that is, the outcome was wound necrosis rather than surgical site infection. After recalculation, postoperative SSI was increased in alcohol drinkers, compared with non-drinkers, in the study by Kortram et al. [33] (OR 1·72, 95% CI [1·01–2·93], *P* = 0·05, I^2^=0%) (**Appendix S21e**) and Shao et al. [34] (OR 2·17, 95% CI [1·13–4·13], *P* = 0·02, I^2^=76%) (**Appendix S21c**). The tendencies of DSSI (OR 1·02, 95% CI [0·64–1·62], *P* = 0·94) and undefined SSI (OR 1·28, 95% CI [0·81–2·03], *P* = 0·29) in our study were different from those in previous two studies.^33^^,^^34^ These differences may have been caused by the lack of description of the time period in the definition of nonunion; or the control group including alcohol drinkers (**Appendices S21b and S21d**).

The mechanism by which cigarette smoking inhibits bone healing is considered to be related to the following substances: (1) nicotine, a vasoconstrictor reduces peripheral blood circulation, (2) carbon monoxide reduces the oxygen-carrying capacity by binding to hemoglobin, and (3) hydrogen cyanide inhibits cytochrome c oxidase to prevent aerobic metabolism.^35^ In addition to weakened blood supply caused by acute detrimental vasoactive effect of smoking, the mechanism of smoking inhibits wound healing includes decreased inflammatory healing response and impaired oxidative bacterial killing mechanisms.^36^ There are few studies concerning the mechanism of the impact of alcohol consumption on fracture healing. One possible mechanism is that alcohol inhibits fracture healing by inhibiting the proliferation of osteoblasts.^37^ Acute alcohol exposure is considered to inhibit wound healing through the following mechanisms: (1) impairment of the early inflammatory response, (2) inhibition of wound closure, angiogenesis, and collagen production, and (3) alteration of the protease at the wound site.^38^ However, the mechanism of the impact of chronic alcohol exposure on wound healing is still lacking.

In the study of Sorensen^36^^,^^39^, smoking cessation before surgery significantly reduced the postoperative wound infection rate (OR 0·40, 95% CI [0·20–0·83], *P* = 0·01, I^2^=19·8%), which was consistent with our study (OR 0·37, 95% CI [0·16–0·89], *P* = 0·03, I^2^=49%). However, smoking cessation time was not classified in the study of Sorensen^36^^,^^39^ This limitation resulted in the failure to provide information concerning the impact of the specific preoperative smoking cessation time. Wong et al. [40] reported that preoperative smoking cessation for more than three to four weeks significantly reduced the risk of postoperative wound healing complications (risk ratio [RR] 0·74, 95% CI [0·56–0·98], *P* = 0·04, I^2^=34%). The tendency of this result was also consistent with our study. However, Wong et al. [40] also had the following methodological problems: (i) Wong et al. [40] misinterpreted the nonunion of bone in one of the included studies (Glassman 2000 [41]) as being a wound complication; (ii) combined wound complication was analysed instead of each outcome in detail, which could not provide clear information on the impact of smoking cessation time on specific outcomes; and (iii) in two of the included studies (Chang 1999 [42] and Goodwin 2005 [43]), smoking cessation occurred before the study period, which meant that it was possible to include patients who had quit smoking for a longer period of time. To study the impact of a clear period of time to quit smoking before surgery, these studies were not included in our study. Detailed information on the two previous meta-analyses is shown in **Appendix S22**.

According to the data by the CDC of the US and the Food and Drug Administration (FDA) from 2013 to 2014, the most popular smoking products among adults were cigarettes (17·0%), followed by vaping (3·3%), smokeless tobacco (2·5%), cigars (1·8%), water pipes (0·6%), and regular pipes (0·3%).^44^ Between 2015 and 2018, the prevalence of vaping use increased from 7·4% in 2015 to 9·2% in 2018, whereas the prevalence of cigarette smoking in current vaping users significantly decreased from 56·9% in 2015 to 40·8% in 2018.^45^ A recent meta-analysis also showed that vaping use was associated with increased smoking cessation.^46^ However, the effect of vaping on patients after surgery was unclear. Only two in vivo studies^47^^,^^48^ have investigated the impact of vaping on the postoperative skin flap necrosis area, and the results indicated that vaping increased the postoperative skin flap necrosis area compared with the unexposed group, and the extent of this increase was similar to that of the tobacco smoking group. More clinical studies investigating the impact of vaping on postoperative outcomes need to be conducted.

There were several strengths in the current study. First, to the best of our knowledge, this is the first rigorous and largest systematic review and meta-analysis that investigated the effects of smoking and alcohol consumption on adult non-pathological fractures. Second, in this study, studies including fractures in children and adolescents were excluded as the treatment and healing periods of fractures in children and adolescents are different from those in adults, which reduced the heterogeneity that was present in previous reviews of fracture healing.^49^^,^^50^ Moreover, key outcomes in this study (i.e., superficial and deep infections) were categorized in detail a priori because of clinical differences between the outcomes, such as the prevalence and request for intravenous antibiotics, percutaneous drainage, and reoperation.^51^ However, studies often treat these two types of infection as one to increase the overall event rate, which prevents readers from understanding the impact of smoking or alcohol on each of these infection types individually.^51^ Studies in which the control group may include an exposed population were not included in the meta-analysis, in order to obtain accurate and reliable information concerning the impact of smoking and alcohol consumption on fracture healing. Another strength of this study was the dose-related meta-analyses that were performed to further explore the influence of smoking and alcohol consumption on fracture healing. Finally, we analysed the impacts of preoperative smoking cessation time, NRT, and vaping on bone healing, wound healing, and wound complications.

There were some limitations that were present in this study. First, not all of the included studies adjusted for confounders in their studies, which may have affected their results. Thus, we performed subgroup analyses, and the results were similar between studies that adjusted for confounders versus studies that did not performed adjustments. Second, three case series with 89 patients were included in the quantitative synthesis, which may have affected the results, although the results were similar when we excluded these case series in a sensitivity analysis. Third, 32 of the 58 studies for quantitative synthesis and 31 of the 45 studies for qualitative synthesis for smoking and alcohol consumption did not clearly describe whether the included fractures were non-pathological. However, the results did not change after excluding the studies that lacked clear descriptions of non-pathological fractures. Fourth, high heterogeneity was found in quantitative synthesis for the impact of alcohol consumption on nonunion rate. We conducted a sensitivity analysis by excluding the study one by one and found that although the impact of alcohol consumption on nonunion rate was still not significant, the heterogeneity significantly decreased after the study Throrud 2017 was removed. The small sample size and the expanded definition of nonunion (e.g. failure of fracture union six months after fracture diagnosis or corrective surgery failure within six months after fracture diagnosis) might be the reasons for the heterogeneity (**Appendix S19**). Readers should interpret these results with caution and future studies with large sample sizes are needed to verify this result. Fifth, for the impact of smoking on nonunion, three studies with high ORs were included in meta-analysis that may have affected the results. The high ORs might be due to the small sample size of the studies, which may lead to imprecision of the results. We performed a sensitivity analysis by removing these three studies and found the results did not significantly change, which means the results are robust. Lastly, changes were made to the study post-hoc, which were not stated a-priori in the published protocol. Changes to protocols in meta-analysis increase the risk of bias in the analysis, results and conclusions. Although these changes were made to ensure the analysis answered the study question, they could have been minimised through a more thorough pilot search of the literature.

The results of the current study support smoking cessation as being a part of the management of bone fractures. Although smoking cessation has been recommended for health benefits for over 3 decades, no previous reviews have found sufficient evidence concerning bone healing rates following fractures.^52^ This study confirms that smoking cessation is beneficial for nonunion and DSSI. However, there is no evidence that reductions in alcohol consumption are beneficial in reducing the time to union or in decreasing rates of nonunion, DSSI, and undefined SSI. To explore the dose-response relationship between smoking, alcohol consumption, and fracture healing, future studies should include more high-quality clinical studies that meet the following requirements: (i) the primary purpose is to investigate the influences of smoking and alcohol consumption on fracture healing; (ii) a clear description of the inclusion of non-pathological fractures; (iii) the doses of smoking and alcohol consumption are completely recorded; and (iv) the definitions of outcomes are clear and unified. More studies that investigate the impact of preoperative smoking cessation time, NRT, and vaping on fracture healing also need to be performed in the future.

Our findings suggested that smoking is associated with higher rates of nonunion and deep surgical site infection after non-pathological fracture treatment. Smoking cessation (four weeks or more before surgery) is associated with a decreased rate of wound infection after any sort of surgery. However, there is still a lack of evidence concerning the association between alcohol consumption and these outcomes.

## Declaration of Competing Interest

None.

## Supplementary materials

Image, application 1

## References

[1] Donaldson L.J., Reckless I.P., Scholes S., Mindell J.S., Shelton N.J. (2008). The epidemiology of fractures in England. J Epidemiol Community Health.

[2] Chen W., Lv H., Liu S. (2017). National incidence of traumatic fractures in China: a retrospective survey of 512 187 individuals. Lancet Glob Health.

[3] Swayambunathan J., Dasgupta A., Rosenberg P.S., Hannan M.T., Kiel D.P., Bhattacharyya T. (2020). Incidence of Hip Fracture Over 4 Decades in the Framingham Heart Study. JAMA Intern Med.

[4] Zura R., Xiong Z., Einhorn T. (2016). Epidemiology of fracture nonunion in 18 human bones. JAMA Surg.

[5] Kanakaris N.K., Giannoudis P.V. (2007). The health economics of the treatment of long-bone non-unions. Injury.

[6] Antonova E., Le T.K., Burge R., Mershon J. (2013). Tibia shaft fractures: costly burden of nonunions. BMC Musculoskelet Disord.

[7] Copuroglu C., Calori G.M., Giannoudis P.V. (2013). Fracture non-union: who is at risk?. Injury.

[8] Gaston M.S., Simpson A.H.R.W. (2007). Inhibition of fracture healing. Journal Bone Jt Surg Ser B.

[9] American Academy of Orthopaedic Surgeons. Management of distal radius fractures evidence-based clinical practice guideline. Available from: www.aaos.org/drfcpg. Accessed March 18th, 2021.

[10] American Academy of Orthopaedic Surgeons. Management of hip fractures in the elderly evidence-based clinical practice guideline. Available from: www.aaos.org/globalassets/quality-and-practice-resources/hip-fractures-in-the-elderly/hip-fractures-elderly-clinical-practice-guideline-4-24-19–2.pdf. Accessed March 18th, 2021.

[11] National Institute for Health and Care Excellence. Fractures (complex): assessment and management. Available from: www.nice.org.uk/guidance/ng37. Accessed March 20th, 2021. 26913311

[12] National Institute for Health and Care Excellence. Fractures (non-complex): assessment and management. Available from: www.nice.org.uk/guidance/ng38. Accessed March 20th, 2021.

[13] Pearson R.G., Clement R.G.E., Edwards K.L., Scammell B.E. (2016). Do smokers have greater risk of delayed and non-union after fracture, osteotomy and arthrodesis? A systematic review with meta-analysis. BMJ Open.

[14] Tian R., Zheng F., Zhao W. (2020). Prevalence and influencing factors of nonunion in patients with tibial fracture: systematic review and meta-analysis. J Orthop Surg Res.

[15] Xu B., Chen L., Lee J.H. (2020). Smoking and alcohol drinking and risk of non-union or delayed union after fractures: a protocol for systematic review and dose-response meta-analysis. Medicine.

[16] Centers for Disease Control and Prevention. Adult Tobacco Use Information. Available from: https://www.cdc.gov/nchs/nhis/tobacco/tobacco_glossary.htm. Accessed July 27th, 2020.

[17] Centers for Disease Control and Prevention. Adult Alcohol Use Information. Available from: https://www.cdc.gov/nchs/nhis/alcohol/alcohol_glossary.htm. Accessed August 10th, 2020.

[18] Malin H., Wilson R.W., GD W. (1983). Proceedings of the 1985 public health conference on records and statistics.

[19] Stroup D.F., Berlin J.A., Morton S.C. (2000). Meta-analysis of observational studies in epidemiology: a proposal for reporting. Meta-analysis of observational studies in epidemiology (MOOSE) group. JAMA.

[20] Shamseer L., Moher D., Clarke M. (2015). Preferred reporting items for systematic review and meta-analysis protocols (PRISMA-P) 2015: elaboration and explanation. BMJ.

[21] Centers for Disease Control and Prevention. Learn About Nicotine Replacement Therapy. Available from: https://www.cdc.gov/tobacco/campaign/tips/quit-smoking/guide/explore-medications.html. Accessed August 6th, 2021.

[22] Centers for Disease Control and Prevention. About Electronic Cigarettes (E-Cigarettes). Available from: https://www.cdc.gov/tobacco/basic_information/e-cigarettes/about-e-cigarettes.html. Accessed August 6th, 2021.

[23] Weinlein J.C., Azar F.M., Beaty J.H., Canale S.T. (2016). Campbell's operative orthopaedics.

[24] Whittle A.P., Azar F.M., Beaty J.H., Canale S.T. (2016). Campbell's operative orthopaedics.

[25] National Healthcare Safety Network. Surgical Site Infection (SSI) Event. Available from: https://www.cdc.gov/nhsn/pdfs/pscmanual/9pscssicurrent.pdf. Accessed March 20th, 2021.

[26] Hayden J.A., van der Windt D.A., Cartwright J.L., Côté P., Bombardier C. (2013). Assessing bias in studies of prognostic factors. Ann Intern Med.

[27] Deeks J.J., Higgins J.P.T., Altman D.G. Analysing data and undertaking meta-analyses. In: Higgins J.P.T., Thomas J., Chandler J., Cumpston M., Li T., Page M.J., Welch V.A., editors. Cochrane handbook for systematic reviews of interventions version 61 (updated September 2020): Cochrane; 2020.

[28] Orsini N., Li R., Wolk A., Khudyakov P., Spiegelman D. (2012). Meta-analysis for linear and nonlinear dose-response relations: examples, an evaluation of approximations, and software. Am J Epidemiol.

[29] Khlopas H., Fallat L.M. (2020). Correction of hallux abducto valgus deformity using closing base wedge osteotomy: a study of 101 patients. J Foot Ankle Surg.

[30] Schwarzer G., Carpenter J.R., Rücker G. (2015). Meta-analysis with R.

[31] Sterne J.A., Sutton A.J., Ioannidis J.P. (2011). Recommendations for examining and interpreting funnel plot asymmetry in meta-analyses of randomised controlled trials. BMJ.

[32] Offner P.J., Moore E.E., Biffl W.L. (1999). Male gender is a risk factor for major infections after surgery. Arch Surg.

[33] Kortram K., Bezstarosti H., Metsemakers W.J. (2017). Risk factors for infectious complications after open fractures; a systematic review and meta-analysis. Int Orthop.

[34] Shao J., Zhang H., Yin B., Li J., Zhu Y., Zhang Y. (2018). Risk factors for surgical site infection following operative treatment of ankle fractures: a systematic review and meta-analysis. Int J Surg.

[35] Scolaro J.A., Schenker M.L., Yannascoli S., Baldwin K., Mehta S., Ahn J. (2014). Cigarette smoking increases complications following fracture: a systematic review. J Bone Jt Surg Am.

[36] Sørensen L.T. (2012). Wound healing and infection in surgery. The clinical impact of smoking and smoking cessation: a systematic review and meta-analysis. Arch Surg.

[37] Richards C.J., Graf K.W., Mashru R.P. (2017). The effect of opioids, alcohol, and nonsteroidal anti-inflammatory drugs on fracture union. Orthop Clin N Am.

[38] Guo S., Dipietro L.A. (2010). Factors affecting wound healing. J Dent Res.

[39] Sørensen L.T. (2012). Wound healing and infection in surgery. The clinical impact of smoking and smoking cessation. A systematic review and meta-analysis. Wound Repair Regen.

[40] Wong J., Lam D.P., Abrishami A., Chan M.T., Chung F. (2012). Short-term preoperative smoking cessation and postoperative complications: a systematic review and meta-analysis. Can J Anaesth.

[41] Glassman S.D., Anagnost S.C., Parker A., Burke D., Johnson J.R., Dimar J.R. (2000). The effect of cigarette smoking and smoking cessation on spinal fusion. Spine (Phila Pa 1976).

[42] Chang D.W., Reece G.P., Wang B. (2000). Effect of smoking on complications in patients undergoing free TRAM flap breast reconstruction. Plast Reconstr Surg.

[43] Goodwin S.J., McCarthy C.M., Pusic A.L. (2005). Complications in smokers after postmastectomy tissue expander/implant breast reconstruction. Ann Plast Surg.

[44] Hu S.S., Neff L., Agaku I.T. (2016). Tobacco product use among adults-United States, 2013-2014. MMWR Morb Mortal Wkly Rep.

[45] Owusu D., Huang J., Weaver S.R. (2019). Patterns and trends of dual use of e-cigarettes and cigarettes among U.S. adults, 2015-2018. Prev Med Rep.

[46] Wang R.J., Bhadriraju S., Glantz S.A. (2021). E-cigarette use and adult cigarette smoking cessation: a meta-analysis. Am J Public Health.

[47] Rau A.S., Reinikovaite V., Schmidt E.P., Taraseviciene-Stewart L., Deleyiannis F.W. (2017). Electronic cigarettes are as toxic to skin flap survival as tobacco cigarettes. Ann Plast Surg.

[48] Troiano C., Jaleel Z., Spiegel J.H. (2019). Association of electronic cigarette vaping and cigarette smoking with decreased random flap viability in rats. JAMA Facial Plast Surg.

[49] Shi J., Chen Z., Xu B. (2014). Causes and treatment of mandibular and condylar fractures in children and adolescents: a review of 104 cases. JAMA Otolaryngol Head Neck Surg.

[50] Stanford T.C., Rodriguez R.P., Hayes J.T. (1966). Tibial-shaft fractures in adults and children. JAMA.

[51] Lawson E.H., Hall B.L., Ko C.Y. (2013). Risk factors for Superficial vs deep/organ-space surgical site infections: implications for quality improvement initiatives. JAMA Surg.

[52] Hernigou J., Schuind F. (2019). Tobacco and bone fractures: a review of the facts and issues that every orthopaedic surgeon should know. Bone Jt Res.

