# Supplementary material for: The influence of smoking and alcohol on bone healing: Systematic review and meta-analysis of non-pathological fractures
Source: eClinicalMedicine. 2021 Oct 31;42:101179. doi: 10.1016/j.eclinm.2021.101179 (PMC8571530; doi:10.1016/j.eclinm.2021.101179)
Supplement: Supplementary file 1 [file mmc1.pdf]

Contents

Appendix S1. The published protocol for the systematic review and meta-analysis 2

Appendix S2. Search strategies on electronic databases 8

Appendix S3. PRISMA flow chart of the impact of preoperative smoking cessation time, nicotine replacement therapy (NRT), and vaping 57

Appendix S4. Search results 58

Appendix S5. Summary of 58 studies for the quantitative synthesis about the impacts of smoking and alcohol consumption 59

Appendix S6. Summary of 45 studies for the qualitative synthesis about the impacts of smoking and alcohol consumption 86

Appendix S7. Summary of 19 studies about the impacts of preoperative smoking cessation time, nicotine replacement therapy, and vaping 109

Appendix S8. Risk of bias for 58 studies included in the meta-analyses of the impacts of smoking and alcohol consumption 121

Appendix S9. List of studies about the impacts of smoking and alcohol consumption on fracture healing and surgical site infection 122

Appendix S10. Data of the studies comparing nonunion between smokers and non-smokers 123

Appendix S11. Forest plot of meta-analysis of nonunion rate of alcohol drinkers vs. non-drinkers 124

Appendix S12. Forest plots of the meta-analyses of secondary outcomes of smokers vs. non-smokers 125

Appendix S13. Forest plots of the meta-analyses of secondary outcomes of alcohol drinkers vs. non-drinkers 126

Appendix S14. List of the studies and the results of the impacts of preoperative smoking cessation time, NRT, and vaping 127

Appendix S15. Forest plots of the meta-analyses of 15a) impaired wound healing rate of smoking cessation vs. non-smokers and 15b) hematoma rate of smoking cessation vs. continuous smokers 129

Appendix S16. Funnel plots and Rucker's test for publication bias 130

Appendix S17. Results of the dose-related analysis of outcomes 131

Appendix S18. Results of the subgroup analysis 132

Appendix S19. Results of the sensitivity analysis 140

Appendix S20. Problems in the included studies in four previous systematic reviews concerning the effects of smoking on nonunion and surgical site infection 145

Appendix S21. Problems in the included studies in two previous systematic reviews about effects of alcohol drinking on nonunion and surgical site infection 150

Appendix S22. Problems in the two previous meta-analyses concerning the effects of preoperative smoking cessation time on wound healing and wound complications 153

Appendix S1. The published protocol for the systematic review and meta-analysis

Study Protocol Systematic Review

Medicine

OPEN

Smoking and alcohol drinking and risk of non-union or delayed union after fractures

A protocol for systematic review and dose–response meta-analysis

Bin Xu, MD[a,b], Lingxiao Chen, MD[c,*], Jae Hyup Lee, MD, PhD[a,d,e,*]

Abstract

Introduction: To the best of our knowledge, there is no consensus on dose–response between smoking, alcohol drinking, and bone healing. The aim of the present study is to conduct a comprehensive systematic review and dose–response meta-analysis of studies to estimate the influence of smoking and alcohol use on the success of non-pathologic bone fracture healing in adult patients.

Methods: A systematic search will be performed using MEDLINE, EMBASE and Cochrane CENTRAL, CINAHL, and AMED databases to identify randomized controlled trials and observational studies which have assessed the effect of smoking or alcohol drinking on fracture healing. Primary outcomes include delayed union or nonunion rate and time to union. Secondary outcomes are common complications which occur during bone healing including malunion and wound infection. Risk of bias will be evaluated using the Quality In Prognosis Studies (QUIPS) tool for quality assessment of each study. Dose–response meta-analysis will be performed between smoking, alcohol drinking, and bone healing. Evaluation of the quality of evidence will be conducted using the Grading of Recommendations Assessment, Development, and Evaluation (GRADE) system.

Results: The present study will assess the effects of smoking and alcohol drinking on non-pathologic bone fracture healing in adult patients.

Conclusion: We hope that this systematic review and dose-response meta-analysis will provide high quality evidence on dose–response between smoking, alcohol drinking, and bone fracture healing.

PROSPERO registration number: CRD42019131454.

Abbreviations: CIs = confident intervals, GRADE = grading of recommendations assessment, development, and evaluation, MD = mean difference, MOOSE = Meta-Analysis Of Observational Studies in Epidemiology, MSC = mesenchymal stem cells, NSAIDs = nonsteroidal anti-inflammatory drugs, OPN = osteopontin, PRISMA-P = preferred reporting items for systematic review and meta-analysis protocols, PROSPERO = International Prospective Register of Systematic Reviews, QUIPS = quality in prognosis studies, RCTs = randomized controlled trials, RRs = risk ratios.

Keywords: alcohol drinking, dose–response, fracture healing, smoking, systematic review

BX is sponsored by China Scholarship Council.

The authors have no conflicts of interest to disclose.

[a] Department of Orthopedic Surgery, College of Medicine, Seoul National University, Seoul, Republic of Korea, [b] Department of Orthopedic Surgery, Tianjin Hospital, Tianjin University, Tianjin, China, [c] Institute of Bone and Joint Research, Kolling Institute, Sydney Medical School, Faculty of Medicine and Health, University of Sydney, Sydney, New South Wales, Australia, [d] Department of Orthopedic Surgery, SMG-SNU Boramae Medical Center, [e] Institute of Medical and Biological Engineering, Medical Research Center, Seoul National University, Medical Research Center, Seoul, Republic of Korea.

[*] Correspondence: Jae Hyup Lee, Department of Orthopedic Surgery, College of Medicine, Seoul National University, Seoul, Republic of Korea (e-mail: spinelee@snu.ac.kr); Lingxiao Chen, Institute of Bone and Joint Research, Kolling Institute, Sydney Medical School, Faculty of Medicine and Health, University of Sydney, Sydney, New South Wales, Australia (e-mail: lche4036@uni.sydney.edu.au).

Copyright © 2020 the Author(s). Published by Wolters Kluwer Health, Inc. This is an open access article distributed under the Creative Commons Attribution License 4.0 (CCBY), which permits unrestricted use, distribution, and reproduction in any medium, provided the original work is properly cited.

How to cite this article: Xu B, Chen L, Lee JH. Smoking and alcohol drinking and risk of non-union or delayed union after fractures: A protocol for systematic review and dose-response meta-analysis. Medicine 2020;99:5(e18744).

Received: 11 December 2019 / Accepted: 16 December 2019

http://dx.doi.org/10.1097/MD.0000000000018744

1. Introduction

The annual occurrence rate of fractures in the whole skeletal system is 3.6 per 100 people in England.[1] Although most of the fractures heal eventually, it has been reported that about 0.1 million of 2 million long bone fractures are converted into nonunion annually in USA.[2] Delayed union and nonunion of fractures not only extend the length of hospital stay but also increase economic burden of family.[3–7]

The main clinical factors related to inhibition of bone fracture healing include smoking and alcohol consumption.[8,9] Previous studies have shown that smokers have higher risk for worse mechanical characteristics of intrinsic trabecular bone,[10] bone fractures,[11,12] delayed union and non-union after open or closed fractures,[13–15] postoperative fracture healing complications including surgical site infection,[14,16,17] and mortality after fracture.[18,19] Numerous factors including reduction in peripheral blood flow caused by nicotine, which is a power vasoconstrictor, reduction in the oxygen-carrying capacity of hemoglobin due to bonding of hemoglobin with carbon monoxide, and obstacles in aerobic metabolism through inhibition of cytochrome c oxidase caused by hydrogen cyanide are the negative effects of smoking on bone healing.[13] Exposure

to high dose of nicotine has been reported to inhibit bone regeneration in a rabbit osteotomy and distraction model.[20] Increase in nonunion rate after plating in proximal humeral fractures along with smoking level was also found in a clinical study.[21] Thus, smoking level is considered as an important factor to affect bone healing.

Regarding alcohol drinking, published studies indicate that alcohol consumption has harmful effects on bone health including a higher rate of fracture[22–25] and higher incidence of infection at the surgical site.[26] Decrease in integrin β1 receptor and osteopontin (OPN) expression is considered as the possible mechanism of delayed union and nonunion of fractures caused by alcohol. Interaction between OPN and integrin β1 receptor induces mesenchymal stem cells (MSC) migration to fracture site and endochondral ossification.[27,28] Alcohol consumption has been reported to affect bone repair in a rat fibula osteotomy model in a dose-dependent manner.[8] Consequently, alcohol consumption is considered as an important factor related to bone healing.

Only one meta-analysis has been published with a focus on the effect of smoking on the outcomes of fracture healing.[13] However, the limitations of the study were as follows:

1. absence of dose–response analysis and
2. the search date of the meta-analysis that was May 2015.

In addition, there exists no meta-analysis about the effect of alcohol drinking on fracture healing. Therefore, our aim is to comprehensively perform a meta-analysis that will investigate dose–response among smoking or alcohol drinking and fracture healing in the entire skeletal system. The results of this meta-analysis might identify prognostic factors associated with bone healing which could help develop the impact of prognostic models for individualized prediction of fracture healing outcomes.

The present study presents the protocol for a systematic review and dose–response meta-analysis, which will compare delayed union or nonunion rates, time to union and complications related to bone healing between smokers and non-smokers and between alcohol users and non-alcohol users. Subsequently, the quantitative relationship between extent of smoking and alcohol consumption and the outcomes mentioned above will be explored.

2. Objective

The objective of the current systematic review and dose–response meta-analysis is to investigate the influence of smoking or alcohol use on the success of bone healing in adult patients after non-pathologic fractures by answering the following question: What is the influence of smoking and alcohol drinking on the healing rate, healing time, and complication rate including malunion and wound infection.

3. Methods

The current protocol is presented following the Preferred Reporting Items for Systematic Review and Meta-Analysis Protocols (PRISMA-P) guidance[29] and the reporting of the meta-analysis will follow the guidelines proposed by Meta-analysis Of Observational Studies in Epidemiology (MOOSE).[30] This protocol has been registered in the international prospective register of systematic reviews (PROSPERO; Registration number CRD42019131454).

3.1. Eligibility criteria

3.1.1. Study designs. Randomized controlled trials (RCTs) and observational studies (cohort, case–control, and cross-sectional studies) will be included in the study.

3.1.2. Participants. We will include studies examining adults aged over 18 years who had traumatic bone fractures in any location. Patients with fractures caused by pathological factors, such as cancer, kidney disease, human immunodeficiency virus, and patients who underwent joint replacement using prosthesis or amputation for first-time treatment will be excluded based on the reason of slow healing and low healing rate. If mixed participants with traumatic fractures and pathological fractures exist in a study, we will include the study in which patients with pathological fractures account for <20%.[31]

3.1.3. Exposure. The studies exploring the effect of current smoking and alcohol consumption on bone healing in patients with traumatic fractures will be included. Definitions of current smoking and current alcohol consumption are shown in the data items section.

3.1.4. Comparators. Patients who have never smoked and who never drank will be the comparators of this study. Definitions of never smokers and never drinkers are also presented in the data items section.

3.1.5. Outcomes. The outcomes will comprise of delayed union or nonunion rate, time to union, malunion rate, and wound infection rate.

3.2. Information sources

We will undertake a systematical search using MEDLINE, EMBASE and Cochrane CENTRAL, CINAHL and AMED databases from inception with language restriction of English. Also, the manual search will be performed by screening references of included studies for relevant references.

3.3. Search strategy

Search strategies will be performed by an experienced librarian using keywords of "smoking," "nicotine," "cigarettes," "alcohol," "alcohol drinking," "alcoholism," "fracture," "bone healing," "delayed union," "nonunion," "malunion," and "surgical wound infection" through MEDLINE, EMBASE and Cochrane CENTRAL, CINAHL and AMED databases with the limitation to English. The full search strategy used in MEDLINE via OVID is presented in Table 1.

3.4. Study records

3.4.1. Data management. We will use EndNote X7 to manage search results, remove duplicate literatures, and select studies based on the eligibility criteria.

3.4.2. Selection process. The selection process will be presented in a PRISMA-compliant flow chart (Fig. 1). Study selection will be independently performed by two authors by screening the titles and abstracts of literatures. Moreover, further selection will be done by reading full text of records identified potentially eligible studies. Any disagreement generated during selection process will be resolved based on mutual discussion with each other or with the help from the third reviewer. In addition, the information about the excluded studies whose full

Table 1

MEDLINE via OVID search strategy.

Number	Query
1	exp Smoking/
2	exp "Tobacco Use Disorder"/
3	Nicotine$.ab,ti.
4	Cigarette$.ab,ti.
5	Smok$.ab,ti.
6	tobacco$.ab,ti.
7	1 or 2 or 3 or 4 or 5 or 6
8	exp Alcohol Drinking/
9	exp Alcoholism/
10	exp Drinking Behavior/
11	(Dr?nk$ adj3 (Behavior$ or Alcohol$)).ab,ti.
12	Alcoholism$.ab,ti.
13	alcoholic$.ab,ti.
14	(alcohol$ adj3 (dependen$ or disorder$ or drink$ or misuse or abuse$ or consumption)).ab,ti.
15	8 or 9 or 10 or 11 or 12 or 13 or 14
16	7 or 15
17	exp Fractures, Bone/
18	Broken Bone$.ab,ti.
19	Bone Fracture$.ab,ti.
20	Spiral Fracture$.ab,ti.
21	Torsion Fracture$.ab,ti.
22	exp Fracture Fixation/
23	Fracture Fixation$.ab,ti.
24	Skeletal Fixation$.ab,ti.
25	Fracture Reduction$.ab,ti.
26	exp Fracture Healing/
27	(fractur$ adj3 healing).ab,ti.
28	(non?union or nonunion or un?united or ununited or delayed?union or union or malunion).ab,ti.
29	exp Surgical Wound Infection/
30	Surgical Wound Infection$.ab,ti.
31	Surgical Site Infection$.ab,ti.
32	Postoperative Wound Infection$.ab,ti.
33	(superficial adj2 SSI$).ab,ti.
34	(deep adj2 SSI$).ab,ti.
35	(Organ adj space SSI$).ab,ti.
36	or/17-35
37	16 and 36
38	limit 37 to humans

texts will be read along with the reasons for exclusion would be provided.

3.4.3. Data collection process. Two authors will independently perform data extraction from eligible studies using a standardized form. Disagreement will be resolved based on mutual discussion with each other or by the third reviewer. In the case of uncertainties, we will contact the original investigators for resolving the issue.

3.4.4. Data items. The data that will be extracted are:

1. basic characteristics of the studies (first author, journal name, publication year, group, study design, etc);
2. characteristics of the participants (sex, age, race, BMI, height, weight, and fracture location, etc);
3. other factors that may slow bone healing time (diabetes, use of nonsteroidal anti-inflammatory drugs [NSAIDs], and fluoroquinolone family of antibiotics)[32];
4. exposure details (treatment, smoking type [e.g., cigarette, cigar, and waterpipe], smoking dose, alcohol type [e.g., beer, wine, and spirits], and level of alcohol consumption); and
5. outcome measures (sample sizes, delayed union and nonunion rate, time to union, and complications during bone healing, etc).

Current smokers are defined as participants who smoke at least 10 cigarettes per day for the past 1 year and never smokers are participants who did not smoke during the past year and smoked <100 cigarettes during their lifetime.[33] Alcohol intake will be converted into grams per day based on the standard drink size (1 mL = 0.8 g, 1 oz = 28.35 g, 1 drink = 14 g, and 1 unit = 7.9 g). And level of alcohol drinking will be classified into light (<7 g/day), moderate (7–14 g/day), heavy (>14 g/day), and binge drinking (28–35 g/day).[34] Never drinker is defined as participants with no alcohol consumption within the preceding 12 months.[35] Current drinker is defined as participants who drank alcohol within the past 12 months.

Regarding missing data of eligible studies, we will contact the original authors. Also, reminder emails will be sent twice at most within a period of 8 weeks and will wait for the next 12 weeks at the maximum for the reply.

3.5. Outcomes and prioritization

The primary outcomes comprise of delayed union or nonunion rate and time to union. Delayed union and nonunion are diagnosed when the time frame for fracture union is from 3 to 6 months and generally over 9 months, respectively, which varies based on fracture location and the level of soft-tissue related injury.[2] Time to union is defined as the time from initiation of bone fracture to the occurrence of clinical union (stabilization of fracture site and painlessness) and radiographic union (trabecular or cortical bone crosses the fracture site).[32] Secondary outcomes include common complications occurring during bone healing including malunion rate and wound infection rate.[36] Malunion is defined as a fracture that is not anatomically healed. Wound infection is defined as infection, which is probably related to operation, occurring within 30 days after the operation when there is no implant left or within 1 year when there is implant left in surgical site.[37]

The hypothesis is that outcomes of RCTs and cohort studies might be similar to case-control and cross-sectional studies.

3.6. Methodological quality assessment of individual studies

Two authors will independently evaluate the risk of bias in each included study using the Quality In Prognosis Studies (QUIPS) tool for quality assessment of RCT, cohort study, case-control study, and cross-sectional study. In total, representativeness of the study sample (study participants domain), whether participants with follow-up data represent persons enrolled in the study (study attrition domain), adequacy of prognostic factor measurement (prognostic factor measurement domain), the adequacy of outcome measurement (outcome measurement domain), potential confounding factors (study confounding domain), and the appropriateness of the study's statistical analysis and completeness of reporting (statistical analysis and reporting domain) will be assessed for each study. Studies will be classified into low, moderate, and high risk of bias according to the results.

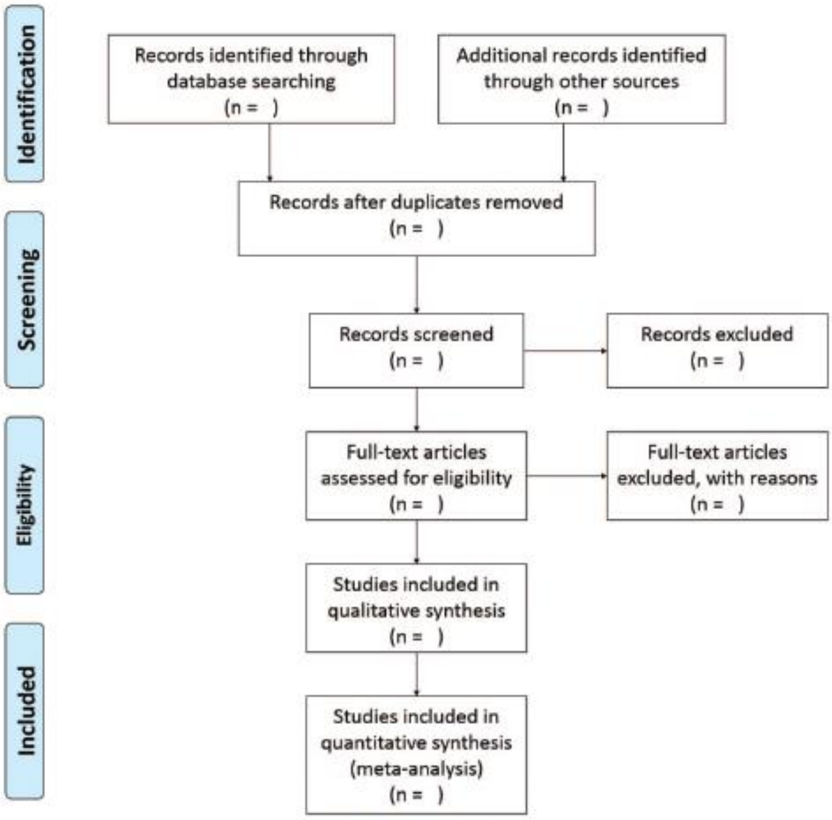

Figure 1. PRISMA flow diagram.

Any controversy during assessment will be managed via discussion or consulting the third author. The results of risk of bias of each study will be shown in a table form.

3.7. Assessment of heterogeneity

We will assess statistical heterogeneity between studies by calculating I^2 value by adhering to the Cochrane Handbook for Systematic Reviews of Interventions.[31] Value of I^2 larger than 75% means considerable statistical heterogeneity. Data of studies with low statistical heterogeneity, clinical heterogeneity, and methodological heterogeneity will be used for quantitative synthesis.

3.8. Data synthesis

If more than 2 studies eligible are included, as data is expected to vary across studies, meta-analysis will be performed using random-effects model[38] to combine data of bone healing outcomes with the tools of RevMan 5.3 (Denmark) and R software 3.6.1 using package "metafor." For continuous data including the time to union, mean difference (MD) with 95% confident intervals (CIs) will be used to evaluate effect size. Dichotomous data comprising of delayed and nonunion rate and complications will be analyzed using risk ratios (RRs) with 95% CIs. If studies show substantial clinical/methodological heterogeneity or <2 studies are included, quantitative synthesis will not be performed. A narrative review of results will be illustrated. In addition, dose–response meta-analysis will be conducted using generalized least squares for trend estimation. Estimation of linear trends will be assessed using the correlated natural logs of RR across exposure categories. We will test the non-linear trend through restricted cubic spline model.[39] The median or mean level of the exposure will be allocated to each relevant exposure category. Calculation of the median will be performed using the midpoint of each category if no available data is acquired. Meta-regression will be performed as follows: age; ratio of male to female; BMI; sample size; use of NSAIDs; use of fluoroquinolone family of antibiotics; and diabetes.

3.8.1. Subgroup analysis and investigation of heterogeneity.

If the necessary data are available, subgroup analyses will be done comparing RCTs vs. cohort vs. case-control and cross-sectional studies.

For primary outcomes (delayed union or nonunion rate and time to union), sensitivity analyses will be conducted in order to confirm whether our findings are destabilized by factors as follows:

1. studies with low risk of bias and
2. studies performed with no commercial fund assistance.

Results of sensitivity analysis will be presented in a table form.

3.9. Publication bias assessment

Publication bias of included studies will be evaluated using funnel plots according to Cochrane Handbook for Systematic Reviews for Interventions[31] if more than 10 studies are included.

3.10. Grading quality of evidence

Quality of evidence will be assessed by two authors independently using the Grading of Recommendations Assessment, Development and Evaluation (GRADE) system.[40] Evidence will be categorized as high, moderate, low, and very low according to study design, risk of bias, inconsistency, indirectness, imprecision, publication bias, magnitude of the effect, dose–response gradient, and confounding bias.

3.11. Ethics and dissemination

Ethical approval is not required for this study because data to be used is from published literatures. Our findings will be expected to provide important data support for clinical treatment and prevention. The results of our review will be published in a peer-reviewed scientific journal and presented at international conferences.

3.12. Patient and public involvement

No patient was involved in this protocol for systematic review and meta-analysis.

4. Discussion

Delayed union and nonunion of non-pathologic bone fracture are major public health problems, that not only reduce quality of life of patients but also increase economic burden of family. To date, only one meta-analysis has evaluated association between smoking and fracture healing. However, search date was May 2015. In addition, no meta-analysis has assessed the effect of alcohol drinking on fracture healing. Moreover, dose–response between smoking, alcohol drinking and fracture healing is still not known. We will perform this systematic review and meta-analysis of the effect of smoking, alcohol drinking on delayed union or nonunion, time to union, malunion and wound infection to provide patients and clinicians high quality evidence on association between them.

Acknowledgments

We thank PARK Eun-Sun at Seoul National university Medical library for her contribution in developing the search strategy in MEDLINE database via Ovid.

Author contributions

Conceptualization: Lingxiao Chen, Jae Hyup Lee.
Methodology: Bin Xu, Lingxiao Chen.
Writing – original draft: Bin Xu.
Writing – review & editing: Bin Xu, Lingxiao Chen, Jae Hyup Lee.
Jae Hyup Lee: 0000-0002-2141-0266.

References

[1] Donaldson LJ, Reckless IP, Scholes S, et al. The epidemiology of fractures in England. J Epidemiol Community Health 2008;62:174–80.
[2] Weinlein JC. Azar FM, Beaty JH, Canale ST. Delayed union and nonunion of fractures. Campbell's Operative Orthopaedics 13 edPhiladelphia, PA: Elsevier; 2016;3081–116.
[3] Antonova E, Le TK, Burge R, et al. Tibia shaft fractures: costly burden of nonunions. BMC Musculoskelet Disord 2013;14.
[4] Dahabreh Z, Calori GM, Kanakaris NK, et al. A cost analysis of treatment of tibial fracture nonunion by bone grafting or bone morphogenetic protein-7. Int Orthop 2009;33:1407–14.
[5] Dahabreh Z, Dimitriou R, Giannoudis PV. Health economics: a cost analysis of treatment of persistent fracture non-unions using bone morphogenetic protein-7. Injury 2007;38:371–7.
[6] Kanakaris NK, Giannoudis PV. The health economics of the treatment of long-bone non-unions. Injury 2007;38(Suppl. 2):S77–84.
[7] Mills LA, Simpson AH. The relative incidence of fracture non-union in the Scottish population (5.17 million): a 5-year epidemiological study. BMJ Open 2013;3:
[8] Chakkalakal DA, Novak JR, Fritz ED, et al. Inhibition of bone repair in a rat model for chronic and excessive alcohol consumption. Alcohol 2005;36:201–14.
[9] Gaston MS, Simpson AHRW. Inhibition of fracture healing. J Bone Joint Surg Ser B 2007;89:1553–60.
[10] Rodrigues AM, Caetano-Lopes J, Vale AC, et al. Smoking is a predictor of worse trabecular mechanical performance in hip fragility fracture patients. J Bone Miner Metab 2012;30:692–9.
[11] Shen GS, Li Y, Zhao G, et al. Cigarette smoking and risk of hip fracture in women: a meta-analysis of prospective cohort studies. Injury 2015;46:1333–40.
[12] Wu ZJ, Zhao P, Liu B, et al. Effect of cigarette smoking on risk of hip fracture in men: a meta-analysis of 14 prospective cohort studies. PLoS One 2016;11:e0168990.
[13] Pearson RG, Clement RG, Edwards KL, et al. Do smokers have greater risk of delayed and non-union after fracture, osteotomy and arthrodesis? A systematic review with meta-analysis. BMJ Open 2016;6:e010303.
[14] Castillo RC, Bosse MJ, MacKenzie EJ, et al. Impact of smoking on fracture healing and risk of complications in limb-threatening open tibia fractures. J Orthop Trauma 2005;19:151–7.
[15] Hernigou J, Schuind F. Smoking as a predictor of negative outcome in diaphyseal fracture healing. Int Orthop 2013;37:883–7.
[16] Sørensen LT. Wound healing and infection in surgery: the clinical impact of smoking and smoking cessation: a systematic review and meta-analysis. Arch Surg 2012;147:373–83.
[17] Kortram K, Bezstarosti H, Metsemakers WJ, et al. Risk factors for infectious complications after open fractures; a systematic review and meta-analysis. Int Orthop 2017;41:1965–82.
[18] Chang W, Lv H, Feng C, et al. Preventable risk factors of mortality after hip fracture surgery: systematic review and meta-analysis. Int J Surg 2018;52:320–8.
[19] Solbakken SM, Meyer HE, Stigum H, et al. Excess mortality following hip fracture: impact of self-perceived health, smoking, and body mass index. A NOREPOS study. Osteoporos Int 2017;28:881–7.
[20] Ma L, Zheng LW, Cheung LK. Influence of nicotine on blood perfusion and bone healing during distraction osteogenesis. Ann R Australas Coll Dent Surg 2008;19:52–4.
[21] Boesmueller S, Wech M, Gregori M, et al. Risk factors for humeral head necrosis and non-union after plating in proximal humeral fractures. Injury 2016;47:350–5.
[22] Zhang X, Yu Z, Yu M, et al. Alcohol consumption and hip fracture risk. Osteoporos Int 2015;26:531–42.
[23] Berg KM, Kunins HV, Jackson JL, et al. Association between alcohol consumption and both osteoporotic fracture and bone density. Am J Med 2008;121:406–18.

[24] Sogaard AJ, Ranhoff AH, Meyer HE, et al. The association between alcohol consumption and risk of hip fracture differs by age and gender in Cohort of Norway: a NOREPOS study. Osteoporos Int 2018;29:2457–67.
[25] Chrcanovic BR. Factors influencing the incidence of maxillofacial fractures. Oral Maxillofac Surg 2012;16:3–17.
[26] Shao J, Zhang H, Yin B, et al. Risk factors for surgical site infection following operative treatment of ankle fractures: a systematic review and meta-analysis. Int J Surg 2018;56:124–32.
[27] Natoli RM, Yu H, Meislin MC, et al. Alcohol exposure decreases osteopontin expression during fracture healing and osteopontin-mediated mesenchymal stem cell migration in vitro. J Orthop Surg Res 2018;13:101.
[28] Roper PM, Abbasnia P, Vuchkovska A, et al. Alcohol-related deficient fracture healing is associated with activation of FoxO transcription factors in mice. J Orthop Res 2016;34:2106–15.
[29] Moher D, Shamseer L, Clarke M, et al. Preferred reporting items for systematic review and meta-analysis protocols (PRISMA-P) 2015 statement. System Rev 2015;4:1.
[30] Stroup DF, Berlin JA, Morton SC, et al. Meta-analysis of observational studies in epidemiology: a proposal for reporting. Meta-analysis of Observational Studies in Epidemiology (MOOSE) group. JAMA 2000;283:2008–12.
[31] Higgins JP, Green S. Cochrane Handbook for Systematic Reviews of Interventions. Hoboken, NJ: John Wiley & Sons; 2011.
[32] Whittle AP. Azar FM, Beaty JH, Canale ST. General principles of fracture treatment. Campbell's Operative Orthopaedics 13 edPhiladelphia, PA: Elsevier; 2016;2656–711.
[33] Weywadt CR, Kiehl KA, Claus ED. Neural correlates of response inhibition in current and former smokers. Behav Brain Res 2017;319:207–18.
[34] Cha BH, Jang MJ, Lee SH. Alcohol consumption can reduce the risk of gallstone disease: a systematic review with a dose-response meta-analysis of case-control and cohort studies. Gut Liver 2019;13:114–31.
[35] Dakhoul L, Ghabril M, Gu J, et al. Heavy consumption of alcohol is not associated with worse outcomes in patients with idiosyncratic drug-induced liver injury compared to non-drinkers. Clin Gastroenterol Hepatol 2018;16:722–9.e722.
[36] Stannard JP, Schmidt AH, Kregor PJ. Surgical Treatment of Orthopaedic Trauma. 1 edNew York: Thieme; 2007.
[37] Mangram AJ, Horan TC, Pearson ML, et al. Guideline for prevention of surgical site infection, 1999. Centers for Disease Control and Prevention (CDC) Hospital Infection Control Practices Advisory Committee. Am J Infect Control 1999;27:97–132. quiz 133-134; discussion 196.
[38] Borenstein M, V Hedges L, PT Higgins J, et al. Introduction to Meta-Analysis. Chichester, UK: John Wiley & Sons; 2009.
[39] Orsini N, Li R, Wolk A, et al. Meta-analysis for linear and nonlinear dose-response relations: examples, an evaluation of approximations, and software. Am J Epidemiol 2012;175:66–73.
[40] Schünemann H, Brożek J, Guyatt G, et al. Handbook for Grading the Quality of Evidence and the Strength of Recommendations Using the GRADE Approach. Updated October 2013. Available at https://gdt.gradepro.org/app/handbook/handbook.html. Accessed April 20, 2019.

Appendix S2. Search strategies on electronic databases

A detailed search strategy was shown in **Appendix S2** as follows:

(1) Search strategy concerning the impacts of smoking and alcohol consumption on fracture healing, surgical site infection (SSI), and malunion was conducted for the initial search (**Appendices S2.1.1a to e**), the first updated search (**Appendices S2.1.2a to e**), the second updated search (**Appendices S2.1.3a to e**), and the third updated search (**Appendices S2.1.4a to e**).

(2) Search strategy concerning the impacts of preoperative smoking cessation time, nicotine replacement therapy (NRT), and vaping on fracture healing, SSI, and malunion were conducted for the initial search (**Appendices S2.2.1a to e)** and the updated search (**Appendices S2.2.2a to e)**.

(3) Search strategies concerning the impacts of preoperative smoking cessation time, NRT, and vaping on bone healing were conducted for the initial search (**Appendices S2.3.1a to e)** and the updated search **(Appendices S2.3.2a to e)**.

(4) Search strategies concerning the impacts of preoperative smoking cessation time, NRT, and vaping on wound healing and wound complications were conducted for the initial search (**Appendices S2.4.1a to e)** and the updated search (**Appendices S2.4.2a to e)**.

S2.1. Search strategies for the impacts of smoking and alcohol consumption on fracture healing, surgical site infection, and malunion

S2.1.1. The first search strategy

S2.1.1a. MEDLINE database (inception of database to October 2nd, 2019)

#	Searches	Results
1	exp Smoking/	142,448
2	exp "Tobacco Use Disorder"/	10,822
3	Nicotine$.ab,ti.	38,488
4	Cigarette$.ab,ti.	68,089
5	Smok$.ab,ti.	266,275
6	tobacco$.ab,ti.	94,768
7	1 or 2 or 3 or 4 or 5 or 6	367,986
8	exp Alcohol Drinking/	66,263
9	exp Alcoholism/	73,662
10	exp Drinking Behavior/	72,625
11	(Dr?nk$ adj3 (Behavior$ or Alcohol$)).ab,ti.	22,655
12	Alcoholism$.ab,ti.	26,529
13	alcoholic$.ab,ti.	61,238
14	(alcohol$ adj3 (dependen$ or disorder$ or drink$ or misuse or abuse$ or consumption)).ab,ti.	93,190
15	8 or 9 or 10 or 11 or 12 or 13 or 14	218,381
16	7 or 15	547,380
17	exp Fractures, Bone/	177,976
18	Broken Bone$.ab,ti.	268

19	Bone Fracture$.ab,ti.	9,706
20	Spiral Fracture$.ab,ti.	338
21	Torsion Fracture$.ab,ti.	42
22	exp Fracture Fixation/	59,384
23	Fracture Fixation$.ab,ti.	3,009
24	Skeletal Fixation$.ab,ti.	522
25	Fracture Reduction$.ab,ti.	2,262
26	exp Fracture Healing/	12,813
27	(fractur$ adj3 healing).ab,ti.	9,296
28	(non?union or nonunion or un?united or ununited or delayed?union or union or malunion).ab,ti.	57,940
29	exp Surgical Wound Infection/	35,173
30	Surgical Wound Infection$.ab,ti.	1,270
31	Surgical Site Infection$.ab,ti.	9,621
32	Postoperative Wound Infection$.ab,ti.	1,589
33	(superficial adj2 SSI$).ab,ti.	310
34	(deep adj2 SSI$).ab,ti.	320
35	(Organ adj space SSI$).ab,ti.	223
36	17 or 18 or 19 or 20 or 21 or 22 or 23 or 24 or 25 or 26 or 27 or 28 or 29 or 30 or 31 or 32 or 33 or 34 or 35	276,386
37	16 and 36	4,614
38	limit 37 to humans	4,120

S2.1.1b. EMBASE database (inception of database to October 2nd, 2019)

No.	Query	Results
#38	#37 AND 'human'/de	10,159
#37	#16 AND #36	11,029
#36	#17 OR #18 OR #19 OR #20 OR #21 OR #22 OR #23 OR #24 OR #25 OR #26 OR #27 OR #2 8 OR #29 OR #30 OR #31 OR #32 OR #33 OR #34 OR #35	439,010
#35	(organ NEAR/1 'space ssi*'):ab,ti	435
#34	(deep NEAR/2 ssi*):ab,ti	557
#33	(superficial NEAR/2 ssi*):ab,ti	571
#32	postoperative wound infection*':ab,ti	2,026
#31	surgical site infection*':ab,ti	14,309
#30	surgical wound infection*':ab,ti	1,615
#29	surgical infection'/exp	46,243
#28	non?union:ab,ti OR nonunion:ab,ti OR un?united:ab,ti OR ununited:ab,ti OR delayed?union:ab,ti OR union:ab,ti OR malunion:ab,ti	78,271
#27	(fractur* NEAR/3 healing):ab,ti	11,699
#26	fracture healing'/exp	48,776

#25	fracture reduction*':ab,ti	2,707
#24	skeletal fixation*':ab,ti	614
#23	fracture fixation*':ab,ti	3,389
#22	fracture fixation'/exp	88,416
#21	torsion fracture*':ab,ti	47
#20	spiral fracture*':ab,ti	391
#19	bone fracture*':ab,ti	13,397
#18	broken bone*':ab,ti	359
#17	fracture'/exp	310,363
#16	#7 OR #15	801,490
#15	#8 OR #9 OR #10 OR #11 OR #12 OR #13 OR #14	291,059
#14	(alcohol* NEAR/3 (dependen* OR disorder* OR drink* OR misuse OR abuse* OR consumption)):ab,ti	132,128
#13	alcoholic*:ab,ti	93,160
#12	alcoholism*:ab,ti	38,539
#11	(dr?nk* NEAR/3 (behavior* OR alcohol*)):ab,ti	31,868
#10	drinking behavior'/exp	48,683
#9	alcoholism'/exp	127,760
#8	drinking behavior'/exp	48,683
#7	#1 OR #2 OR #3 OR #4 OR #5 OR #6	494,653
#6	tobacco*:ab,ti	114,728
#5	smok*:ab,ti	391,841
#4	cigarette*:ab,ti	86,692
#3	nicotine*:ab,ti	48,682
#2	tobacco dependence'/exp	20,241
#1	smoking'/exp	376,274

S2.1.1c. Cochrane library database (inception of database to October 10th, 2019)

ID	Search	Hits
#1	MeSH descriptor: [Smoke] explode all trees	385
#2	MeSH descriptor: [Tobacco Use Disorder] explode all trees	1,516
#3	(Nicotine*):ti,ab,kw	6,366
#4	(Cigarette*):ti,ab,kw	7,739
#5	(Smok*):ti,ab,kw	34,367
#6	(tobacco*):ti,ab,kw	8,119
#7	#1 or #2 or #3 or #4 or #5 or #6	36,995
#8	MeSH descriptor: [Alcohol Drinking] explode all trees	3,611
#9	MeSH descriptor: [Alcohols] explode all trees	35,871

#10	MeSH descriptor: [Drinking Behavior] explode all trees	3,736
#11	((Dr?nk* near/3 (Behavior* or Alcohol*))):ti,ab,kw	6,195
#12	(Alcoholism*):ti,ab,kw	6,292
#13	(alcoholic*):ti,ab,kw	6,503
#14	((alcohol* near/3 (dependen* or disorder* or drink* or misuse or abuse* or consumption))):ti,ab,kw	13,679
#15	#8 or #9 or #10 or #11 or #12 or #13 or #14	53,749
#16	#7 or #15	86,796
#17	MeSH descriptor: [Fractures, Bone] explode all trees	5,511
#18	(Broken Bone*):ti,ab,kw	145
#19	(Bone Fracture*):ti,ab,kw	9,830
#20	(Spiral Fracture*):ti,ab,kw	48
#21	(Torsion Fracture*):ti,ab,kw	43
#22	MeSH descriptor: [Fracture Fixation] explode all trees	1,619
#23	(Fracture Fixation*):ti,ab,kw	3,473
#24	(Skeletal Fixation*):ti,ab,kw	156
#25	(Fracture Reduction*):ti,ab,kw	3,594
#26	MeSH descriptor: [Fracture Healing] explode all trees	510
#27	((fractur* near/3 healing)):ti,ab,kw	1,386
#28	((non?union or nonunion or un?united or ununited or delayed?union or union or malunion)):ti,ab,kw	3,127
#29	MeSH descriptor: [Surgical Wound Infection] explode all trees	3,228
#30	(Surgical Wound Infection*):ti,ab,kw	6,595
#31	(Surgical Site Infection*):ti,ab,kw	3,147
#32	(Postoperative Wound Infection*):ti,ab,kw	5,239
#33	((superficial near/2 SSI*)):ti,ab,kw	90
#34	((deep near/2 SSI*)):ti,ab,kw	76
#35	((Organ near/1 space SSI*)):ti,ab,kw	82
#36	#17 or #18 or #19 or #20 or #21 or #22 or #23 or #24 or #25 or #26 or #27 or #28 or #29 or # 30 or #31 or #32 or #33 or #34 or #35	24,406
#37	#16 and #36	880

S2.1.1d. CINAHL database (inception of database to October 10th, 2019)

#	Query	Results
S28	S12 AND S27	1,272
S27	S13 or S14 or S15 or S16 or S17 or S18 or S19 or S20 or S21 or S22 or S23 or S24 or S25 or S26	39,143
S26	TI Organ w1 space SSI* or AB Organ w1 space SSI*	78
S25	TI deep w2 SSI* or AB deep w2 SSI*	126
S24	TI superficial w2 SSI* or AB superficial w2 SSI*	115

S23	TI Postoperative Wound Infection* or AB Postoperative Wound Infection*	270
S22	TI Surgical Site Infection* or AB Surgical Site Infection*	
S21	TI Surgical Wound Infection* or AB Surgical Wound Infection*	310
S20	(MM "Surgical Wound Infection")	6,310
S19	TI (non?union or nonunion or un?united or ununited or delayed?union or union or malunion) OR AB (non?union or nonunion or un?united or ununited or delayed?union or union or malunion)	17,200
S18	TI fractur* w3 healing OR AB fractur* w3 healing	1,609
S17	(MM "Fracture Healing")	1,460
S16	TI Fracture Fixation* or AB Fracture Fixation* or TI Skeletal Fixation* or AB Skeletal Fixation* o r TI Fracture Reduction* or AB Fracture Reduction*	1,806
S15	(MH "Fracture Fixation+")	12,432
S14	TI Broken Bone* or AB Broken Bone* or TI Bone Fracture* or AB Bone Fracture* or TI Spiral Fracture* or AB Spiral Fracture* or TI Torsion Fracture* or AB Torsion Fracture*	2,472
S13	(MM "Carpal Fractures")	95
S12	S4 or S11	221,951
S11	S5 or S6 or S7 or S8 or S9 or S10	55,189
S10	TI ((alcohol* w3 (dependen* or disorder* or drink* or misuse or abuse* or consumption))) OR A B ((alcohol* w3 (dependen* or disorder* or drink* or misuse or abuse* or consumption)))	29,653
S9	TI (Dr?nk* w3 (Behavior* or Alcohol*)) OR AB (Dr?nk* w3 (Behavior* or Alcohol*))	6,175
S8	TI Alcoholism* or AB Alcoholism*	4,370
S7	(MH "Drinking Behavior+")	28,839
S6	(MM "Alcoholism")	10,755
S5	(MH "Alcohol Drinking+")	27,642
S4	S1 or S2 or S3	201,263
S3	TI Nicotine* OR AB Nicotine* OR TI Cigarette* OR AB Cigarette* OR TI Smok* OR AB Smok* OR TI tobacco* OR AB tobacco*	97,194
S2	(MH "Substance Use Disorders+")	146,077
S1	(MH "Smoking+")	63,985

S2.1.1e. AMED database via EBSCOhost (inception of database to October 28th, 2019)

#	Searches	Results
S27	S10 AND S26	82
S26	S13 or S14 or S15 or S16 or S17 or S18 or S19 or S20 or S21 or S22 or S23 or S24 or S25	1,478
S25	TI Organ w1 space SSI* or AB Organ w1 space SSI*	1
S24	TI Organ w1 space SSI* or AB Organ w1 space SSI*	0
S23	TI deep w2 SSI* or AB deep w2 SSI*	1
S22	TI superficial w2 SSI* or AB superficial w2 SSI*	1
S21	Postoperative Wound Infection* or AB Postoperative Wound Infection*	10
S20	Surgical Site Infection* or AB Surgical Site Infection*	20
S19	Surgical Wound Infection* or AB Surgical Wound Infection*	9

S18	SU Surgical Wound Infection	8
S17	TI non?union or nonunion or un?united or ununited or delayed?union or union or malunion OR AB non?union or nonunion or un?united or ununited or delayed?union or union or malunion	1,035
S16	TI fractur* w3 healing OR AB fractur* w3 healing	145
S15	SU Fracture Healing	84
S14	TI Fracture Fixation* or AB Fracture Fixation* or TI Skeletal Fixation* or AB Skeletal Fixation* or TI Fracture Reduction* or AB Fracture Reduction*	151
S13	TI Broken Bone* or AB Broken Bone* or TI Bone Fracture* or AB Bone Fracture* or TI Spiral Fracture* or AB Spiral Fracture* or TI Torsion Fracture* or AB Torsion Fracture*	159
S12	SU Fracture Fixation	791
S11	SU Carpal Fractures	0
S10	S1 or S2 or S3 or S5 or S6 or S7 or S8 or S9	48,959
S9	TI alcohol* w3 dependen* or disorder* or drink* or misuse or abuse* or consumption OR AB alcohol* w3 dependen* or disorder* or drink* or misuse or abuse* or consumption	46,456
S8	TI Dr?nk* w3 Behavior* or Alcohol* OR AB Dr?nk* w3 Behavior* or Alcohol*	2,430
S7	TI Alcoholism* or AB Alcoholism*	133
S6	SU Drinking Behavior	58
S5	SU Alcoholism	449
S4	SU Alcohol Drinking	244
S3	TI Nicotine* OR AB Nicotine* OR TI Cigarette* OR AB Cigarette* OR TI Smok* OR AB Smok* OR TI tobacco* OR AB tobacco*	1,545
S2	SU Substance Use Disorders	163
S1	SU smoking	

S2.1.2. The second search strategy (the first updated search)

S2.1.2a. MEDLINE database (year of 2019 to July 7th, 2020)

#	Searches	Results
1	exp Smoking/	146,898
2	exp "Tobacco Use Disorder"/	11,185
3	Nicotine$.ab,ti.	40,161
4	Cigarette$.ab,ti.	71,525
5	Smok$.ab,ti.	279,443
6	tobacco$.ab,ti.	99,356
7	1 or 2 or 3 or 4 or 5 or 6	384,957
8	exp Alcohol Drinking/	68,851
9	exp Alcoholism/	74,920
10	exp Drinking Behavior/	75,315
11	(Dr?nk$ adj3 (Behavior$ or Alcohol$)).ab,ti.	23,781
12	Alcoholism$.ab,ti.	26,886
13	alcoholic$.ab,ti.	63,894

14	(alcohol$ adj3 (dependen$ or disorder$ or drink$ or misuse or abuse$ or consumption)).ab,ti.	97,889
15	8 or 9 or 10 or 11 or 12 or 13 or 14	227,022
16	7 or 15	571,108
17	exp Fractures, Bone/	183,577
18	Broken Bone$.ab,ti.	288
19	Bone Fracture$.ab,ti.	10,296
20	Spiral Fracture$.ab,ti.	343
21	Torsion Fracture$.ab,ti.	42
22	exp Fracture Fixation/	61,391
23	Fracture Fixation$.ab,ti.	3,236
24	Skeletal Fixation$.ab,ti.	533
25	Fracture Reduction$.ab,ti.	2,424
26	exp Fracture Healing/	13,353
27	(fractur$ adj3 healing).ab,ti.	9,839
28	(non?union or nonunion or un?united or ununited or delayed?union or union or malunion).ab,ti.	61,320
29	exp Surgical Wound Infection/	36,308
30	Surgical Wound Infection$.ab,ti.	1,304
31	Surgical Site Infection$.ab,ti.	10,730
32	Postoperative Wound Infection$.ab,ti.	1,627
33	(superficial adj2 SSI$).ab,ti.	350
34	(deep adj2 SSI$).ab,ti.	359
35	(Organ adj space SSI$).ab,ti.	248
36	17 or 18 or 19 or 20 or 21 or 22 or 23 or 24 or 25 or 26 or 27 or 28 or 29 or 30 or 31 or 32 or 33 or 34 or 35	287,122
37	16 and 36	4,927
38	limit 37 to humans	4,372
39	limit 38 to yr="2019 - 2020"	274

S2.1.2b. EMBASE database (year of 2019 to July 9th, 2020)

No.	Query	Results
#39	#37 AND 'human'/de AND [2019-2020]/py	1,230
#38	#37 AND 'human'/de	10,773
#37	#16 AND #36	11,660
#36	#17 OR #18 OR #19 OR #20 OR #21 OR #22 OR #23 OR #24 OR #25 OR #26 OR #27 OR #2 8 OR #29 OR #30 OR #31 OR #32 OR #33 OR #34 OR #35	459,247
#35	(organ NEAR/1 'space ssi*'):ab,ti	494
#34	(deep NEAR/2 ssi*):ab,ti	613
#33	(superficial NEAR/2 ssi*):ab,ti	631

#32	postoperative wound infection*':ab,ti	2085
#31	surgical site infection*':ab,ti	15,840
#30	surgical wound infection*':ab,ti	1,666
#29	surgical infection'/exp	49,452
#28	non?union:ab,ti OR nonunion:ab,ti OR un?united:ab,ti OR ununited:ab,ti OR delayed?union:ab,ti OR union:ab,ti OR malunion:ab,ti	81,831
#27	(fractur* NEAR/3 healing):ab,ti	12,370
#26	fracture healing'/exp	51,122
#25	fracture reduction*':ab,ti	2,903
#24	skeletal fixation*':ab,ti	628
#23	fracture fixation*':ab,ti	3,632
#22	fracture fixation'/exp	91,629
#21	torsion fracture*':ab,ti	47
#20	spiral fracture*':ab,ti	397
#19	bone fracture*':ab,ti	14,168
#18	broken bone*':ab,ti	380
#17	fracture'/exp	324,200
#16	#7 OR #15	838,072
#15	#8 OR #9 OR #10 OR #11 OR #12 OR #13 OR #14	302,502
#14	(alcohol* NEAR/3 (dependen* OR disorder* OR drink* OR misuse OR abuse* OR consumption)):ab,ti	137,940
#13	alcoholic*:ab,ti	97,491
#12	alcoholism*:ab,ti	39,109
#11	(dr?nk* NEAR/3 (behavior* OR alcohol*)):ab,ti	33,227
#10	drinking behavior'/exp	50,107
#9	alcoholism'/exp	130,661
#8	drinking behavior'/exp	50,107
#7	#1 OR #2 OR #3 OR #4 OR #5 OR #6	587,909
#6	tobacco*:ab,ti	120,232
#5	smok*:ab,ti	411,994
#4	cigarette*:ab,ti	90,733
#3	nicotine*:ab,ti	50,667
#2	tobacco dependence'/exp	21,177
#1	smoking'/exp	391,649

S2.1.2c. Cochrane library database (year of 2019 to July 9th, 2020)

ID	Search	Hits
#1	MeSH descriptor: [Smoke] explode all trees	389
#2	MeSH descriptor: [Tobacco Use Disorder] explode all trees	1,659

#3	(Nicotine*):ti,ab,kw	6,902
#4	(Cigarette*):ti,ab,kw	8,087
#5	(Smok*):ti,ab,kw	35,554
#6	(tobacco*):ti,ab,kw	8,744
#7	#1 or #2 or #3 or #4 or #5 or #6	38,345
#8	MeSH descriptor: [Alcohol Drinking] explode all trees	3,778
#9	MeSH descriptor: [Alcohols] explode all trees	37,334
#10	MeSH descriptor: [Drinking Behavior] explode all trees	3,911
#11	((Dr?nk* near/3 (Behavior* or Alcohol*))):ti,ab,kw	6,624
#12	(Alcoholism*):ti,ab,kw	6,571
#13	(alcoholic*):ti,ab,kw	6,897
#14	((alcohol* near/3 (dependen* or disorder* or drink* or misuse or abuse* or consumption))):ti,ab,kw	14,293
#15	#8 or #9 or #10 or #11 or #12 or #13 or #14	55,979
#16	#7 or #15	90,320
#17	MeSH descriptor: [Fractures, Bone] explode all trees	5,913
#18	(Broken Bone*):ti,ab,kw	163
#19	(Bone Fracture*):ti,ab,kw	10,221
#20	(Spiral Fracture*):ti,ab,kw	50
#21	(Torsion Fracture*):ti,ab,kw	44
#22	MeSH descriptor: [Fracture Fixation] explode all trees	1,689
#23	(Fracture Fixation*):ti,ab,kw	3,637
#24	(Skeletal Fixation*):ti,ab,kw	159
#25	(Fracture Reduction*):ti,ab,kw	3,742
#26	MeSH descriptor: [Fracture Healing] explode all trees	522
#27	((fractur* near/3 healing)):ti,ab,kw	1,422
#28	((non?union or nonunion or un?united or ununited or delayed?union or union or malunion)):ti,ab,kw	3,168
#29	MeSH descriptor: [Surgical Wound Infection] explode all trees	3,372
#30	(Surgical Wound Infection*):ti,ab,kw	6,892
#31	(Surgical Site Infection*):ti,ab,kw	3,376
#32	(Postoperative Wound Infection*):ti,ab,kw	5,404
#33	((superficial near/2 SSI*)):ti,ab,kw	101
#34	((deep near/2 SSI*)):ti,ab,kw	76
#35	((Organ near/1 space SSI*)):ti,ab,kw	80
#36	#17 or #18 or #19 or #20 or #21 or #22 or #23 or #24 or #25 or #26 or #27 or #28 or #29 or # 30 or #31 or #32 or #33 or #34 or #35	25,399
#37	#16 and #36 with Publication Year from 2019 to 2020, in Trials	124

S2.1.2d. CINAHL database (year of 2019 to July 8th, 2020)

#	Query	Results
S28	S12 AND S27 (Limiters - Published Date: 20190101-20200731)	179
S27	S13 or S14 or S15 or S16 or S17 or S18 or S19 or S20 or S21 or S22 or S23 or S24 or S25 or S26	48,593
S26	TI Organ w1 space SSI* or AB Organ w1 space SSI*	92
S25	TI deep w2 SSI* or AB deep w2 SSI*	157
S24	TI superficial w2 SSI* or AB superficial w2 SSI*	139
S23	TI Postoperative Wound Infection* or AB Postoperative Wound Infection*	307
S22	TI Surgical Site Infection* or AB Surgical Site Infection*	5,547
S21	TI Surgical Wound Infection* or AB Surgical Wound Infection*	352
S20	(MM "Surgical Wound Infection")	7,317
S19	TI (non?union or nonunion or un?united or ununited or delayed?union or union or malunion) OR AB (non?union or nonunion or un?united or ununited or delayed?union or union or malunion)	22,293
S18	TI fractur* w3 healing OR AB fractur* w3 healing	2,016
S17	(MM "Fracture Healing")	1,735
S16	TI Fracture Fixation* or AB Fracture Fixation* or TI Skeletal Fixation* or AB Skeletal Fixation* or TI Fracture Reduction* or AB Fracture Reduction*	2,257
S15	(MH "Fracture Fixation+")	15,001
S14	TI Broken Bone* or AB Broken Bone* or TI Bone Fracture* or AB Bone Fracture* or TI Spiral Fracture* or AB Spiral Fracture* or TI Torsion Fracture* or AB Torsion Fracture*	3,089
S13	(MM "Carpal Fractures")	136
S12	S4 or S11	269,169
S11	S5 or S6 or S7 or S8 or S9 or S10	67,779
S10	TI ((alcohol* w3 (dependen* or disorder* or drink* or misuse or abuse* or consumption))) OR AB ((alcohol* w3 (dependen* or disorder* or drink* or misuse or abuse* or consumption)))	37,601
S9	TI (Dr?nk* w3 (Behavior* or Alcohol*)) OR AB (Dr?nk* w3 (Behavior* or Alcohol*))	7,855
S8	TI Alcoholism* or AB Alcoholism*	5,596
S7	(MH "Drinking Behavior+")	34,887
S6	(MM "Alcoholism")	12,779
S5	(MH "Alcohol Drinking+")	33,405
S4	S1 or S2 or S3	243,891
S3	TI Nicotine* OR AB Nicotine* OR TI Cigarette* OR AB Cigarette* OR TI Smok* OR AB Smok* OR TI tobacco* OR AB tobacco*	119,053
S2	(MH "Substance Use Disorders+")	176,155
S1	(MH "Smoking+")	76,503

S2.1.2e. AMED database via Ovid (inception of database to July 10th, 2020)

#	Searches	Results
1	exp smoking/	624
2	(Nicotine* OR Cigarette* OR Smok* OR tobacco*).ab,ti	1,622
3	exp Alcohol Drinking/	249

4	exp Alcoholism/	384
5	exp Drinking Behavior/	303
6	Alcoholism*.ab,ti	137
7	(Dr?nk* adj3 Behavior* or Behavior* adj3 Dr?nk* or Alcohol*).ab,ti	1,903
8	(alcohol* adj3 dependen* or dependen* adj3 alcohol* or disorder* or drink* or misuse or abuse* or consumption).ab,ti	18,874
9	1 or 2 or 3 or 4 or 5 or 6 or 7 or 8	21,435
10	exp Fracture Fixation/	812
11	(Broken Bone* or Bone Fracture* or Spiral Fracture* or Torsion Fracture*).ab,ti	141
12	(Fracture Fixation* or Skeletal Fixation* or Fracture Reduction*).ab,ti	152
13	exp Fracture Healing/	88
14	(fractur* adj3 healing or healing adj3 fractur*).ab,ti	204
15	(non?union or nonunion or un?united or ununited or delayed?union or union or malunion).ab,ti	1,010
16	Surgical Wound Infection*.ab,ti	1
17	Surgical Site Infection*.ab,ti	24
18	Postoperative Wound Infection*.ab,ti	10
19	(superficial adj2 SSI* or SSI* adj2 superficial).ab,ti	1
20	(deep adj2 SSI* or SSI* adj2 deep).ab,ti	1
21	(Organ adj1 space SSI* or space adj1 Organ SSI*).ab,ti	0
22	10 or 11 or 12 or 13 or 14 or 15 or 16 or 17 or 18 or 19 or 20 or 21	2,066
23	9 and 22	98

S2.1.3. The third search strategy (the second updated search)

S2.1.3a. MEDLINE database (year of 2020 to October 14th, 2020)

#	Searches	Results
1	exp Smoking/	148,321
2	exp "Tobacco Use Disorder"/	11,297
3	Nicotine$.ab,ti.	42,140
4	Cigarette$.ab,ti.	75,518
5	Smok$.ab,ti.	297,163
6	tobacco$.ab,ti.	105,720
7	1 or 2 or 3 or 4 or 5 or 6	407,028
8	exp Alcohol Drinking/	69,599
9	exp Alcoholism/	75,326
10	exp Drinking Behavior/	76,111
11	(Dr?nk$ adj3 (Behavior$ or Alcohol$)).ab,ti.	25,042
12	Alcoholism$.ab,ti.	27,547
13	alcoholic$.ab,ti.	68,264

14	(alcohol$ adj3 (dependen$ or disorder$ or drink$ or misuse or abuse$ or consumption)).ab,ti.	103,534
15	8 or 9 or 10 or 11 or 12 or 13 or 14	237,382
16	7 or 15	601,585
17	exp Fractures, Bone/	185,240
18	Broken Bone$.ab,ti.	316
19	Bone Fracture$.ab,ti.	11,318
20	Spiral Fracture$.ab,ti.	359
21	Torsion Fracture$.ab,ti.	42
22	exp Fracture Fixation/	61,987
23	Fracture Fixation$.ab,ti.	3,625
24	Skeletal Fixation$.ab,ti.	545
25	Fracture Reduction$.ab,ti.	2,701
26	exp Fracture Healing/	13,470
27	(fractur$ adj3 healing).ab,ti.	10,579
28	(non?union or nonunion or un?united or ununited or delayed?union or union or malunion).ab,ti.	66,703
29	exp Surgical Wound Infection/	36,654
30	Surgical Wound Infection$.ab,ti.	1,356
31	Surgical Site Infection$.ab,ti.	12,043
32	Postoperative Wound Infection$.ab,ti.	1,699
33	(superficial adj2 SSI$).ab,ti.	387
34	(deep adj2 SSI$).ab,ti.	401
35	(Organ adj space SSI$).ab,ti.	274
36	17 or 18 or 19 or 20 or 21 or 22 or 23 or 24 or 25 or 26 or 27 or 28 or 29 or 30 or 31 or 32 or 33 or 34 or 35	297,283
37	16 and 36	5,219
38	limit 37 to humans	4,441
39	limit 38 to yr="2020 -Current"	66

S2.1.3b. EMBASE database (year of 2020 to October 14th, 2020)

No.	Query	Results
#39	#37 AND 'human'/de AND [2020-2020]/py	631
#38	#37 AND 'human'/de	11,063
#37	#16 AND #36	11,957
#36	#17 OR #18 OR #19 OR #20 OR #21 OR #22 OR #23 OR #24 OR #25 OR #26 OR #27 OR #2 8 OR #29 OR #30 OR #31 OR #32 OR #33 OR #34 OR #35	467,895
#35	(organ NEAR/1 'space ssi*'):ab,ti	522
#34	(deep NEAR/2 ssi*):ab,ti	645
#33	(superficial NEAR/2 ssi*):ab,ti	659
#32	postoperative wound infection*':ab,ti	2,102
#31	surgical site infection*':ab,ti	16,487
#30	surgical wound infection*':ab,ti	1,688
#29	surgical infection'/exp	50,825
#28	non?union:ab,ti OR nonunion:ab,ti OR un?united:ab,ti OR ununited:ab,ti OR delayed?union:ab,ti OR union:ab,ti OR malunion:ab,ti	83,572

#27	(fractur* NEAR/3 healing):ab,ti	12,617
#26	fracture healing'/exp	52,164
#25	fracture reduction*':ab,ti	2,984
#24	skeletal fixation*':ab,ti	637
#23	fracture fixation*':ab,ti	3,731
#22	fracture fixation'/exp	93,152
#21	torsion fracture*':ab,ti	47
#20	spiral fracture*':ab,ti	405
#19	bone fracture*':ab,ti	14,469
#18	broken bone*':ab,ti	390
#17	fracture'/exp	329,923
#16	#7 OR #15	852,882
#15	#8 OR #9 OR #10 OR #11 OR #12 OR #13 OR #14	307,277
#14	(alcohol* NEAR/3 (dependen* OR disorder* OR drink* OR misuse OR abuse* OR consumption)):ab,ti	140,628
#13	alcoholic*:ab,ti	99,078
#12	alcoholism*:ab,ti	39,317
#11	(dr?nk* NEAR/3 (behavior* OR alcohol*)):ab,ti	33,928
#10	drinking behavior'/exp	50,755
#9	alcoholism'/exp	131,964
#8	drinking behavior'/exp	50,755
#7	#1 OR #2 OR #3 OR #4 OR #5 OR #6	598,937
#6	tobacco*:ab,ti	122,564
#5	smok*:ab,ti	419,722
#4	cigarette*:ab,ti	92,460
#3	nicotine*:ab,ti	51,454
#2	tobacco dependence'/exp	21,484
#1	smoking'/exp	397,892

S2.1.3c. Cochrane library database (year of 2020 to October 14th, 2020)

ID	Search	Hits
#1	MeSH descriptor: [Smoke] explode all trees	394
#2	MeSH descriptor: [Tobacco Use Disorder] explode all trees	1,676
#3	(Nicotine*):ti,ab,kw	6,980
#4	(Cigarette*):ti,ab,kw	8,216
#5	(Smok*):ti,ab,kw	36,008
#6	(tobacco*):ti,ab,kw	8,912
#7	#1 or #2 or #3 or #4 or #5 or #6	38,845
#8	MeSH descriptor: [Alcohol Drinking] explode all trees	3,833
#9	MeSH descriptor: [Alcohols] explode all trees	37,500
#10	MeSH descriptor: [Drinking Behavior] explode all trees	3,967
#11	((Dr?nk* near/3 (Behavior* or Alcohol*))):ti,ab,kw	6,660
#12	(Alcoholism*):ti,ab,kw	6,677
#13	(alcoholic*):ti,ab,kw	7,034
#14	((alcohol* near/3 (dependen* or disorder* or drink* or misuse or abuse* or consumption))):ti,ab,kw	14,512

#15	#8 or #9 or #10 or #11 or #12 or #13 or #14	56,467
#16	#7 or #15	91,303
#17	MeSH descriptor: [Fractures, Bone] explode all trees	6,024
#18	(Broken Bone*):ti,ab,kw	167
#19	(Bone Fracture*):ti,ab,kw	10,386
#20	(Spiral Fracture*):ti,ab,kw	50
#21	(Torsion Fracture*):ti,ab,kw	46
#22	MeSH descriptor: [Fracture Fixation] explode all trees	1,720
#23	(Fracture Fixation*):ti,ab,kw	3,725
#24	(Skeletal Fixation*):ti,ab,kw	165
#25	(Fracture Reduction*):ti,ab,kw	3,833
#26	MeSH descriptor: [Fracture Healing] explode all trees	529
#27	((fractur* near/3 healing)):ti,ab,kw	1,452
#28	((non?union or nonunion or un?united or ununited or delayed?union or union or malunion)):ti,ab,kw	3,245
#29	MeSH descriptor: [Surgical Wound Infection] explode all trees	3,403
#30	(Surgical Wound Infection*):ti,ab,kw	6,970
#31	(Surgical Site Infection*):ti,ab,kw	3,449
#32	(Postoperative Wound Infection*):ti,ab,kw	5,473
#33	((superficial near/2 SSI*)):ti,ab,kw	105
#34	((deep near/2 SSI*)):ti,ab,kw	79
#35	((Organ near/1 space SSI*)):ti,ab,kw	83
#36	#17 or #18 or #19 or #20 or #21 or #22 or #23 or #24 or #25 or #26 or #27 or #28 or #29 or # 30 or #31 or #32 or #33 or #34 or #35	25,856
#37	#16 and #36 with Publication Year from 2020 to 2020, in Trials	32

S2.1.3d. CINAHL database (year of 2020 to October 14th, 2020)

#	Query	Results
S28	S12 AND S27 (Limiters - Published Date: 20200101-20201031)	102
S27	S13 or S14 or S15 or S16 or S17 or S18 or S19 or S20 or S21 or S22 or S23 or S24 or S25 or S26	46,735
S26	TI Organ w1 space SSI* or AB Organ w1 space SSI*	96
S25	TI deep w2 SSI* or AB deep w2 SSI*	149
S24	TI superficial w2 SSI* or AB superficial w2 SSI*	141
S23	TI Postoperative Wound Infection* or AB Postoperative Wound Infection*	306
S22	TI Surgical Site Infection* or AB Surgical Site Infection*	5,397
S21	TI Surgical Wound Infection* or AB Surgical Wound Infection*	343
S20	(MM "Surgical Wound Infection")	7,127
S19	TI (non?union or nonunion or un?united or ununited or delayed?union or union or malunion) O R AB (non?union or nonunion or un?united or ununited or delayed?union or union or malunion)	21,329
S18	TI fractur* w3 healing OR AB fractur* w3 healing	1,946
S17	(MM "Fracture Healing")	1,719
S16	TI Fracture Fixation* or AB Fracture Fixation* or TI Skeletal Fixation* or AB Skeletal Fixation* or TI Fracture Reduction* or AB Fracture Reduction*	2,165
S15	(MH "Fracture Fixation+")	14,785

S14	TI Broken Bone* or AB Broken Bone* or TI Bone Fracture* or AB Bone Fracture* or TI Spiral Fracture* or AB Spiral Fracture* or TI Torsion Fracture* or AB Torsion Fracture*	2,624
S13	(MM "Carpal Fractures")	111
S12	S4 or S11	254,671
S11	S5 or S6 or S7 or S8 or S9 or S10	63,107
S10	TI ((alcohol* w3 (dependen* or disorder* or drink* or misuse or abuse* or consumption))) OR AB ((alcohol* w3 (dependen* or disorder* or drink* or misuse or abuse* or consumption)))	34,567
S9	TI (Dr?nk* w3 (Behavior* or Alcohol*)) OR AB (Dr?nk* w3 (Behavior* or Alcohol*))	7,150
S8	TI Alcoholism* or AB Alcoholism*	5,197
S7	(MH "Drinking Behavior+")	32,700
S6	(MM "Alcoholism")	12,049
S5	(MH "Alcohol Drinking+")	31,305
S4	S1 or S2 or S3	230,971
S3	TI Nicotine* OR AB Nicotine* OR TI Cigarette* OR AB Cigarette* OR TI Smok* OR AB Smok* OR TI tobacco* OR AB tobacco*	113,269
S2	(MH "Substance Use Disorders+")	165,804
S1	(MH "Smoking+")	72,299

S2.1.3e. AMED database (year of 2020 to October 14th, 2020)

#	Searches	Results
1	exp smoking/	629
2	(Nicotine* OR Cigarette* OR Smok* OR tobacco*).ab,ti	1,636
3	exp Alcohol Drinking/	249
4	exp Alcoholism/	388
5	exp Drinking Behavior/	303
6	Alcoholism*.ab,ti	138
7	(Dr?nk* adj3 Behavior* or Behavior* adj3 Dr?nk* or Alcohol*).ab,ti	1,927
8	(alcohol* adj3 dependen* or dependen* adj3 alcohol* or disorder* or drink* or misuse or abuse* or consumption).ab,ti	19,152
9	1 or 2 or 3 or 4 or 5 or 6 or 7 or 8	21,739
10	exp Fracture Fixation/	823
11	(Broken Bone* or Bone Fracture* or Spiral Fracture* or Torsion Fracture*).ab,ti	143
12	(Fracture Fixation* or Skeletal Fixation* or Fracture Reduction*).ab,ti	154
13	exp Fracture Healing/	91
14	(fractur* adj3 healing or healing adj3 fractur*).ab,ti	210
15	(non?union or nonunion or un?united or ununited or delayed?union or union or malunion).ab,ti	1,023
16	Surgical Wound Infection*.ab,ti	1
17	Surgical Site Infection*.ab,ti	26
18	Postoperative Wound Infection*.ab,ti	10
19	(superficial adj2 SSI* or SSI* adj2 superficial).ab,ti	1
20	(deep adj2 SSI* or SSI* adj2 deep).ab,ti	1
21	(Organ adj1 space SSI* or space adj1 Organ SSI*).ab,ti	0
22	10 or 11 or 12 or 13 or 14 or 15 or 16 or 17 or 18 or 19 or 20 or 21	2,091
23	9 and 22	99

24	limit 23 to yr="2020-Current"	4

S2.1.4. The fourth search strategy (the third updated search)

S2.1.4a. MEDLINE database (year of 2020 to August 7th, 2021)

#	Searches	Results
1	exp Smoking/	153,370
2	exp "Tobacco Use Disorder"/	11,673
3	Nicotine$.ab,ti.	42,408
4	Cigarette$.ab,ti.	75,838
5	Smok$.ab,ti.	298,115
6	tobacco$.ab,ti.	105,698
7	1 or 2 or 3 or 4 or 5 or 6	408,689
8	exp Alcohol Drinking/	72,471
9	exp Alcoholism/	76,808
10	exp Drinking Behavior/	79,116
11	(Dr?nk$ adj3 (Behavior$ or Alcohol$)).ab,ti.	25,315
12	Alcoholism$.ab,ti.	27,309
13	alcoholic$.ab,ti.	68,005
14	(alcohol$ adj3 (dependen$ or disorder$ or drink$ or misuse or abuse$ or consumption)).ab,ti.	104,318
15	8 or 9 or 10 or 11 or 12 or 13 or 14	239,073
16	7 or 15	604,414
17	exp Fractures, Bone/	192,917
18	Broken Bone$.ab,ti.	312
19	Bone Fracture$.ab,ti.	11,164
20	Spiral Fracture$.ab,ti.	358
21	Torsion Fracture$.ab,ti.	43
22	exp Fracture Fixation/	64,931
23	Fracture Fixation$.ab,ti.	3,598
24	Skeletal Fixation$.ab,ti.	553
25	Fracture Reduction$.ab,ti.	2,686
26	exp Fracture Healing/	14,083
27	(fractur$ adj3 healing).ab,ti.	10,561
28	(non?union or nonunion or un?united or ununited or delayed?union or union or malunion).ab,ti.	66,632
29	exp Surgical Wound Infection/	37,954
30	Surgical Wound Infection$.ab,ti.	1,345
31	Surgical Site Infection$.ab,ti.	12,338

32	Postoperative Wound Infection$.ab,ti.	1,681
33	(superficial adj2 SSI$).ab,ti.	400
34	(deep adj2 SSI$).ab,ti.	427
35	(Organ adj space SSI$).ab,ti.	280
36	17 or 18 or 19 or 20 or 21 or 22 or 23 or 24 or 25 or 26 or 27 or 28 or 29 or 30 or 31 or 32 or 33 or 34 or 35	303,900
37	16 and 36	5,361
38	limit 37 to humans	4,769
39	limit 38 to yr="2020 -Current"	378

S2.1.4b. EMBASE database (year of 2020 to August 7th, 2021)

No.	Query	Results
#39	#37 AND 'human'/de AND [2020-2021]/py	1,499
#38	#37 AND 'human'/de	12,040
#37	#16 AND #36	12,948
#36	#17 OR #18 OR #19 OR #20 OR #21 OR #22 OR #23 OR #24 OR #25 OR #26 OR #27 OR #28 OR #29 OR #3 0 OR #31 OR #32 OR #33 OR #34 OR #35	494,723
#35	(organ NEAR/1 'space ssi*'):ab,ti	566
#34	(deep NEAR/2 ssi*):ab,ti	709
#33	(superficial NEAR/2 ssi*):ab,ti	727
#32	postoperative wound infection*':ab,ti	2,167
#31	surgical site infection*':ab,ti	18,307
#30	surgical wound infection*':ab,ti	1,740
#29	surgical infection'/exp	54,736
#28	non?union:ab,ti OR nonunion:ab,ti OR un?united:ab,ti OR ununited:ab,ti OR delayed?union:ab,ti OR union:ab,t i OR malunion:ab,ti	88,415
#27	(fractur* NEAR/3 healing):ab,ti	13,399
#26	fracture healing'/exp	55,249
#25	fracture reduction*':ab,ti	3,209
#24	skeletal fixation*':ab,ti	656
#23	fracture fixation*':ab,ti	4,059
#22	fracture fixation'/exp	97,671
#21	torsion fracture*':ab,ti	49
#20	spiral fracture*':ab,ti	420
#19	bone fracture*':ab,ti	15,462
#18	broken bone*':ab,ti	416
#17	fracture'/exp	348,494
#16	#7 OR #15	900,052
#15	#8 OR #9 OR #10 OR #11 OR #12 OR #13 OR #14	322,140
#14	(alcohol* NEAR/3 (dependen* OR disorder* OR drink* OR misuse OR abuse* OR consumption)):ab,ti	148,372

#13	alcoholic*:ab,ti	104,532
#12	alcoholism*:ab,ti	39,870
#11	(dr?nk* NEAR/3 (behavior* OR alcohol*)):ab,ti	35,820
#10	drinking behavior'/exp	52,818
#9	alcoholism'/exp	135,601
#8	drinking behavior'/exp	52,818
#7	#1 OR #2 OR #3 OR #4 OR #5 OR #6	634,513
#6	tobacco*:ab,ti	129,269
#5	smok*:ab,ti	444,665
#4	cigarette*:ab,ti	97,046
#3	nicotine*:ab,ti	53,686
#2	tobacco dependence'/exp	22,544
#1	smoking'/exp	418,522

S2.1.4c. Cochrane library database (year of 2020 to August 7th, 2021)

ID	Search	Hits
#1	MeSH descriptor: [Smoke] explode all trees	415
#2	MeSH descriptor: [Tobacco Use Disorder] explode all trees	1,741
#3	(Nicotine*):ti,ab,kw	7,381
#4	(Cigarette*):ti,ab,kw	8,788
#5	(Smok*):ti,ab,kw	38,541
#6	(tobacco*):ti,ab,kw	9,636
#7	#1 or #2 or #3 or #4 or #5 or #6	41,663
#8	MeSH descriptor: [Alcohol Drinking] explode all trees	4,022
#9	MeSH descriptor: [Alcohols] explode all trees	38,243
#10	MeSH descriptor: [Drinking Behavior] explode all trees	4,167
#11	((Dr?nk* near/3 (Behavior* or Alcohol*))):ti,ab,kw	7,066
#12	(Alcoholism*):ti,ab,kw	6,971
#13	(alcoholic*):ti,ab,kw	7,633
#14	((alcohol* near/3 (dependen* or disorder* or drink* or misuse or abuse* or consumption))):ti,ab,kw	15,509
#15	#8 or #9 or #10 or #11 or #12 or #13 or #14	58,722
#16	#7 or #15	96,041
#17	MeSH descriptor: [Fractures, Bone] explode all trees	6,415
#18	(Broken Bone*):ti,ab,kw	184
#19	(Bone Fracture*):ti,ab,kw	11,248
#20	(Spiral Fracture*):ti,ab,kw	59
#21	(Torsion Fracture*):ti,ab,kw	54
#22	MeSH descriptor: [Fracture Fixation] explode all trees	1,829

#23	(Fracture Fixation*):ti,ab,kw	4,126
#24	(Skeletal Fixation*):ti,ab,kw	179
#25	(Fracture Reduction*):ti,ab,kw	4,176
#26	MeSH descriptor: [Fracture Healing] explode all trees	545
#27	((fractur* near/3 healing)):ti,ab,kw	1,594
#28	((non?union or nonunion or un?united or ununited or delayed?union or union or malunion)):ti,ab,kw	3,605
#29	MeSH descriptor: [Surgical Wound Infection] explode all trees	3,505
#30	(Surgical Wound Infection*):ti,ab,kw	7,420
#31	(Surgical Site Infection*):ti,ab,kw	3,903
#32	(Postoperative Wound Infection*):ti,ab,kw	5,826
#33	((superficial near/2 SSI*)):ti,ab,kw	122
#34	((deep near/2 SSI*)):ti,ab,kw	95
#35	((Organ near/1 space SSI*)):ti,ab,kw	91
#36	#17 or #18 or #19 or #20 or #21 or #22 or #23 or #24 or #25 or #26 or #27 or #28 or #29 or #30 or #31 or #32 o r #33 or #34 or #35	28,087
#37	#16 and #36 with Publication Year from 2020 to 2021, in Trials	113

S2.1.4d. CINAHL database (year of 2020 to August 7th, 2021)

#	Query	Results
S28	S12 AND S27 (Limiters - Published Date: 20201001-20210831)	95
S27	S13 or S14 or S15 or S16 or S17 or S18 or S19 or S20 or S21 or S22 or S23 or S24 or S25 or S26	51,051
S26	TI Organ w1 space SSI* or AB Organ w1 space SSI*	111
S25	TI deep w2 SSI* or AB deep w2 SSI*	168
S24	TI superficial w2 SSI* or AB superficial w2 SSI*	160
S23	TI Postoperative Wound Infection* or AB Postoperative Wound Infection*	319
S22	TI Surgical Site Infection* or AB Surgical Site Infection*	5,924
S21	TI Surgical Wound Infection* or AB Surgical Wound Infection*	352
S20	(MM "Surgical Wound Infection")	7,563
S19	TI (non?union or nonunion or un?united or ununited or delayed?union or union or malunion) OR AB (non? union or nonunion or un?united or ununited or delayed?union or union or malunion)	23,750
S18	TI fractur* w3 healing OR AB fractur* w3 healing	2,082
S17	(MM "Fracture Healing")	1,801
S16	TI Fracture Fixation* or AB Fracture Fixation* or TI Skeletal Fixation* or AB Skeletal Fixation* or TI Fract ure Reduction* or AB Fracture Reduction*	2,369
S15	(MH "Fracture Fixation+")	16,320
S14	TI Broken Bone* or AB Broken Bone* or TI Bone Fracture* or AB Bone Fracture* or TI Spiral Fracture* or AB Spiral Fracture* or TI Torsion Fracture* or AB Torsion Fracture*	2,810
S13	(MM "Carpal Fractures")	119
S12	S4 or S11	267,092
S11	S5 or S6 or S7 or S8 or S9 or S10	66,286

S10	TI ((alcohol* w3 (dependen* or disorder* or drink* or misuse or abuse* or consumption))) OR AB ((alcohol* w3 (dependen* or disorder* or drink* or misuse or abuse* or consumption)))	36,726
S9	TI (Dr?nk* w3 (Behavior* or Alcohol*)) OR AB (Dr?nk* w3 (Behavior* or Alcohol*))	7,588
S8	TI Alcoholism* or AB Alcoholism*	5,329
S7	(MH "Drinking Behavior+")	34,487
S6	(MM "Alcoholism")	12,499
S5	(MH "Alcohol Drinking+")	32,975
S4	S1 or S2 or S3	242,228
S3	TI Nicotine* OR AB Nicotine* OR TI Cigarette* OR AB Cigarette* OR TI Smok* OR AB Smok* OR TI tobacco* OR AB tobacco*	119,612
S2	(MH "Substance Use Disorders+")	173,171
S1	(MH "Smoking+")	74,980

S2.1.4e. AMED database (year of 2020 to August 9th, 2021 via Ovid)

#	Searches	Results
1	exp smoking/	646
2	(Nicotine* OR Cigarette* OR Smok* OR tobacco*).ab,ti	1,683
3	exp Alcohol Drinking/	255
4	exp Alcoholism/	393
5	exp Drinking Behavior/	309
6	Alcoholism*.ab,ti	139
7	(Dr?nk* adj3 Behavior* or Behavior* adj3 Dr?nk* or Alcohol*).ab,ti	2,000
8	(alcohol* adj3 dependen* or dependen* adj3 alcohol* or disorder* or drink* or misuse or abuse* or consumption).ab,ti	19,793
9	1 or 2 or 3 or 4 or 5 or 6 or 7 or 8	22,464
10	exp Fracture Fixation/	843
11	(Broken Bone* or Bone Fracture* or Spiral Fracture* or Torsion Fracture*).ab,ti	147
12	(Fracture Fixation* or Skeletal Fixation* or Fracture Reduction*).ab,ti	158
13	exp Fracture Healing/	92
14	(fractur* adj3 healing or healing adj3 fractur*).ab,ti	214
15	(non?union or nonunion or un?united or ununited or delayed?union or union or malunion).ab,ti	1,075
16	Surgical Wound Infection*.ab,ti	1
17	Surgical Site Infection*.ab,ti	30
18	Postoperative Wound Infection*.ab,ti	10
19	(superficial adj2 SSI* or SSI* adj2 superficial).ab,ti	1
20	(deep adj2 SSI* or SSI* adj2 deep).ab,ti	1
21	(Organ adj1 space SSI* or space adj1 Organ SSI*).ab,ti	0
22	10 or 11 or 12 or 13 or 14 or 15 or 16 or 17 or 18 or 19 or 20 or 21	2,167
23	9 and 22	103

24	limit 23 to yr="2020-Current"	7

S2.2. Search strategies for the impacts of preoperative smoking cessation time, NRT, and vaping on fracture healing, postoperative surgical site infection, and malunion

S2.2.1. The first search strategy

S2.2.1a. MEDLINE database via Ovid (inception of database to April 20th, 2021)

#	Searches	Results
1	exp Fractures, Bone/	189,044
2	Broken Bone$.ab,ti.	302
3	Bone Fracture$.ab,ti.	10,913
4	Spiral Fracture$.ab,ti.	352
5	Torsion Fracture$.ab,ti.	43
6	exp Fracture Fixation/	63,381
7	Fracture Fixation$.ab,ti.	3,486
8	Skeletal Fixation$.ab,ti.	548
9	Fracture Reduction$.ab,ti.	2,603
10	exp Fracture Healing/	13,783
11	(fractur$ adj3 healing).ab,ti.	10,343
12	(non?union or nonunion or un?united or ununited or delayed?union or union or malunion).ab,ti.	65,111
13	exp Surgical Wound Infection/	37,337
14	Surgical Wound Infection$.ab,ti.	1,327
15	Surgical Site Infection$.ab,ti.	11,920
16	Postoperative Wound Infection$.ab,ti.	1,666
17	(superficial adj2 SSI$).ab,ti.	387
18	(deep adj2 SSI$).ab,ti.	407
19	(Organ adj space SSI$).ab,ti.	276
20	1 or 2 or 3 or 4 or 5 or 6 or 7 or 8 or 9 or 10 or 11 or 12 or 13 or 14 or 15 or 16 or 17 or 18 or 19	298,187
21	exp Smoking Cessation/	29,675
22	exp Smoking Reduction/	82
23	((quit$ or stop$ or ceas$ or giv$ or abstain$ or abstinen$ or Cessat$ or reduc$) adj3 (smok$ or tobacco)).ti,ab.	47,526
24	"Tobacco Use Cessation"/	1,238
25	"Tobacco Use Cessation Products"/	1,967
26	Nicotinic Agonists/	7,438
27	Smoking Cessation Agents/	211
28	Nicotine Chewing Gum/	26
29	NRT.ti,ab.	2,180
30	nicotine replacement.tw,kf.	3,605
31	((nicotine or tobacco) adj2 (gum* or lozenge* or patch* or spray*)).tw,kf.	2,481
32	(pharmaco* adj2 (nicotine or tobacco or smoking)).tw,kf.	1,138
33	exp Vaping/	1,666
34	exp Electronic Nicotine Delivery Systems/	4,359
35	(Ecigarette$ or e-cigarette$).ab,ti.	5,132

36	Electronic-Cigarette$.ab,ti.	3,314
37	(electron* adj3 (cig$ or nicotine$)).ab,ti.	3,927
38	(e-cig$ or ecig$).ti,ab.	5,396
39	(vape$ or vaporizer$ or vaporiser$ or vapourizer$ or vapouriser$ or vaping$).ti,ab.	3,368
40	21 or 22 or 23 or 24 or 25 or 26 or 27 or 28 or 29 or 30 or 31 or 32 or 33 or 34 or 35 or 36 o r 37 or 38 or 39	70,169
41	20 and 40	518
42	limit 41 to humans	429

S2.2.1b. EMBASE database (inception of database to April 21st, 2021)

No.	Query	Results
#42	#41 AND 'human'/de	1,241
#41	#20 AND #40	1,386
#40	#21 OR #22 OR #23 OR #24 OR #25 OR #26 OR #27 OR #28 OR #29 OR #30 OR #31 OR #32 OR #33 OR #34 OR #35 OR #36 OR #37 OR #38 OR #39	116,498
#39	vape*:ab,ti OR vaporizer*:ab,ti OR vaporiser*:ab,ti OR vapourizer*:ab,ti OR vapouriser*:ab,ti OR vapin g*:ab,ti	4,216
#38	'e cig*':ab,ti OR ecig*:ab,ti	6,587
#37	(electron* NEAR/3 (cig* OR nicotine*)):ab,ti	4,715
#36	'electronic cigarette*':ab,ti	4,019
#35	ecigarette*:ab,ti OR 'e cigarette*':ab,ti	6,225
#34	'electronic cigarette'/exp	7,725
#33	'vaping'/exp	2,978
#32	(pharmaco* NEAR/2 (nicotine OR tobacco OR smoking)):ab,ti	1,379
#31	((nicotine OR tobacco) NEAR/2 (gum* OR lozenge* OR patch* OR spray*)):ab,ti	2,996
#30	'nicotine replacement':ab,ti	4,560
#29	nrt:ab,ti	2,883
#28	nicotine chewing gum'/exp	2,958
#27	smoking cessation agents'/exp	37
#26	nicotinic agonists'/exp	25,388
#25	tobacco use cessation products'/exp	2,958
#24	'tobacco use cessation'/exp	61,570
#23	((quit* OR stop* OR ceas* OR giv* OR abstain* OR abstinen* OR cessat* OR reduc*) NEAR/3 (sm ok* OR tobacco)):ab,ti	62,145
#22	'smoking reduction'/exp	275
#21	'smoking cessation'/exp	61,570
#20	#1 OR #2 OR #3 OR #4 OR #5 OR #6 OR #7 OR #8 OR #9 OR #10 OR #11 OR #12 OR #13 OR #14 OR #15 OR #16 OR #17 OR #18 OR #19	486,284
#19	(organ NEAR/1 'space ssi*'):ab,ti	550
#18	(deep NEAR/2 ssi*):ab,ti	690
#17	(superficial NEAR/2 ssi*):ab,ti	705
#16	'postoperative wound infection*':ab,ti	2,144
#15	'surgical site infection*':ab,ti	17,707
#14	'surgical wound infection*':ab,ti	1,715
#13	'surgical infection'/exp	53,306
#12	non?union:ab,ti OR nonunion:ab,ti OR un?united:ab,ti OR ununited:ab,ti OR delayed?union:ab,ti OR uni on:ab,ti OR malunion:ab,ti	86,831
#11	(fractur* NEAR/3 healing):ab,ti	13,154

#10	'fracture healing'/exp	54,261
#9	'fracture reduction*':ab,ti	3,138
#8	'skeletal fixation*':ab,ti	653
#7	'fracture fixation*':ab,ti	3,952
#6	'fracture fixation'/exp	96,045
#5	'torsion fracture*':ab,ti	48
#4	'spiral fracture*':ab,ti	415
#3	'bone fracture*':ab,ti	15,151
#2	'broken bone*':ab,ti	407
#1	'fracture'/exp	342,927

S2.2.1c. Cochrane library database (inception of database to April 21st, 2021)

ID	Search	Hits
#1	MeSH descriptor: [Fractures, Bone] explode all trees	6,224
#2	(Broken Bone*):ti,ab,kw	177
#3	(Bone Fracture*):ti,ab,kw	10,926
#4	(Spiral Fracture*):ti,ab,kw	52
#5	(Torsion Fracture*):ti,ab,kw	49
#6	MeSH descriptor: [Fracture Fixation] explode all trees	1,770
#7	(Fracture Fixation*):ti,ab,kw	3,988
#8	(Skeletal Fixation*):ti,ab,kw	176
#9	(Fracture Reduction*):ti,ab,kw	4,052
#10	MeSH descriptor: [Fracture Healing] explode all trees	538
#11	((fractur* near/3 healing)):ti,ab,kw	1,531
#12	((non?union or nonunion or un?united or ununited or delayed?union or union or malunion)):ti,ab,kw	3,486
#13	MeSH descriptor: [Surgical Wound Infection] explode all trees	3,454
#14	(Surgical Wound Infection*):ti,ab,kw	7,242
#15	(Surgical Site Infection*):ti,ab,kw	3,744
#16	(Postoperative Wound Infection*):ti,ab,kw	5,707
#17	((superficial near/2 SSI*)):ti,ab,kw	118
#18	((deep near/2 SSI*)):ti,ab,kw	91
#19	((Organ near/1 space SSI*)):ti,ab,kw	91
#20	#1 or #2 or #3 or #4 or #5 or #6 or #7 or #8 or #9 or #10 or #11 or #12 or #13 or #14 or #15 or #16 or #17 or #18 or #19	27,277
#21	MeSH descriptor: [Smoking Cessation] explode all trees	4,145
#22	MeSH descriptor: [Smoking Reduction] explode all trees	25
#23	((quit* or stop* or ceas* or giv* or abstain* or abstinen* or Cessat* or reduc*) NEAR/3 (smoke* or tobacco)):ti,ab,kw	5,174
#24	MeSH descriptor: [Tobacco Use Cessation Devices] explode all trees	550
#25	MeSH descriptor: [Nicotine Chewing Gum] explode all trees	14
#26	MeSH descriptor: [Nicotinic Agonists] explode all trees	741
#27	(Tobacco Use Cessation Products):ti,ab,kw	287
#28	(Nicotine near/2 patch*):ti,ab,kw	1,299
#29	(Nicotine near/2 delivery device*):ti,ab,kw	103
#30	(nicotine near/2 nasal spray*):ti,ab,kw	97
#31	(smoking near/2 cessation product*):ti,ab,kw	639

#32	(nicotine near/2 replacement):ti,ab,kw	1,837
#33	(nicotine near/2 lozenge*):ti,ab,kw	177
#34	(nicotine polacrilex):ti,ab,kw	50
#35	(nicotine near/2 inhal*):ti,ab,kw	150
#36	(nicotine near/2 chewing gum*):ti,ab,kw	140
#37	nicorette:ti,ab,kw	97
#38	(nicotinic near/2 agonist*):ti,ab,kw	831
#39	(nicotine near/2 tablet*):ti,ab,kw	41
#40	(nicotine near/2 sublingual):ti,ab,kw	25
#41	NRT:ti,ab,kw	926
#42	(nicotine near/3 therap*):ti,ab,kw	2,045
#43	MeSH descriptor: [Vaping] explode all trees	52
#44	MeSH descriptor: [Electronic Nicotine Delivery Systems] explode all trees	148
#45	(Ecigarette* or e-cigarette*):ti,ab,kw	542
#46	(Electronic-Cigarette*):ti,ab,kw	441
#47	((electron* NEAR/3 (cig* OR nicotine*))):ti,ab,kw	543
#48	(e-cig* or ecig*):ti,ab,kw	587
#49	(vape* or vaporizer* or vaporiser* or vapourizer* or vapouriser* or vaping*):ti,ab,kw	2,341
#50	#21 or #22 or #23 or #24 or #25 or #26 or #27 or #28 or #29 or #30 or #31 or #32 or #33 or #34 or #35 or #36 or #37 or #38 or #39 or #40 or #41 or #42 or #43 or #44 or #45 or #46 or #47 or #48 or #49	11,862
#51	#20 and #50 in Trials	38

S2.2.1d. CINAHL database (inception of database to April 21st, 2021)

#	Query	Results
S31	S15 and S30	171
S30	S16 or S17 or S18 or S19 or S20 or S21 or S22 or S23 or S24 or S25 or S26 or S27 or S28 or S29	31,634
S29	TI (vape$ or vaporizer* or vaporiser* or vapourizer* or vapouriser* or vaping*) OR AB (vape$ or vaporizer* or vaporiser* or vapourizer* or vapouriser* or vaping*)	1,624
S28	TI (e-cig* or ecig*) OR AB (e-cig* or ecig*)	3,756
S27	TI (electron* W3 (cig* or nicotine*)) OR AB (electron* W3 (cig* or nicotine*))	2,536
S26	TI Electronic-Cigarette* OR AB Electronic-Cigarette*	2,177
S25	TI (Ecigarette* OR e-cigarette*) OR AB (Ecigarette* OR e-cigarette*)	3,614
S24	(MM "Electronic Cigarettes")	1,919
S23	(MM "Vaping")	197
S22	TI (nicotine and (gum* or lozenge* or spray* or patch*)) OR AB (nicotine and (gum* or lozenge* or spray* or patch*))	1,150
S21	TI nicotine replacement OR AB nicotine replacement	1,836
S20	TI NRT OR AB NRT	947
S19	(MH “Nicotine Chewing Gum” OR (MH “Nicotine Patch”)	454
S18	(MH (“Nicotine Replacement Therapy”) OR (MH “Tobacco Use Cessation Products+”) OR (MH “Nicotinic Agonists+”)	10,955
S17	TI ((quit* or stop* or ceas* or giv* or abstain* or abstinen* or Cessat* or reduc*) W3 (smok* or tobacco)) OR AB ((quit* or stop* or ceas* or giv* or abstain* or abstinen* or Cessat* or reduc*) W3 (smok* or tobacco))	13,454
S16	(MM "Smoking Cessation")	14,099
S15	S1 or S2 or S3 or S4 or S5 or S6 or S7 or S8 or S9 or S10 or S11 or S12 or S13 or S14	48,618

S14	TI Organ w1 space SSI* or AB Organ w1 space SSI*	106
S13	TI deep w2 SSI* or AB deep w2 SSI*	160
S12	TI superficial w2 SSI* or AB superficial w2 SSI*	156
S11	TI Postoperative Wound Infection* or AB Postoperative Wound Infection*	311
S10	TI Surgical Site Infection* or AB Surgical Site Infection*	5,719
S9	TI Surgical Wound Infection* or AB Surgical Wound Infection*	348
S8	(MM "Surgical Wound Infection")	7,394
S7	TI (non?union or nonunion or un?united or ununited or delayed?union or union or malunion) OR A B (non?union or nonunion or un?united or ununited or delayed?union or union or malunion)	22,127
S6	TI fractur* w3 healing OR AB fractur* w3 healing	2,015
S5	(MM "Fracture Healing")	1,766
S4	TI Fracture Fixation* or AB Fracture Fixation* or TI Skeletal Fixation* or AB Skeletal Fixation* or TI Fracture Reduction* or AB Fracture Reduction*	2,276
S3	(MH "Fracture Fixation+")	15,526
S2	TI Broken Bone* or AB Broken Bone* or TI Bone Fracture* or AB Bone Fracture* or TI Spiral Fra cture* or AB Spiral Fracture* or TI Torsion Fracture* or AB Torsion Fracture*	2,740
S1	(MM "Carpal Fractures")	115

S2.2.1e. AMED database (inception of database to April 22nd, 2021)

#	Searches	Results
1	exp Fracture Fixation/	828
2	(Broken Bone* or Bone Fracture* or Spiral Fracture* or Torsion Fracture*).ab,ti.	144
3	(Fracture Fixation* or Skeletal Fixation* or Fracture Reduction*).ab,ti	154
4	exp Fracture Healing/	91
5	(((fractur* adj3 healing) or healing) adj3 fractur*).ab,ti.	213
6	(non?union or nonunion or un?united or ununited or delayed?union or union or malunion).ab,ti.	1,041
7	Surgical Wound Infection*.ab,ti.	1
8	Surgical Surgical Site Infection*.ab,ti.Site Infection*.ab,ti	27
9	Postoperative Wound Infection*.ab,ti.	10
10	(((superficial adj2 SSI*) or SSI*) adj2 superficial).ab,ti.	1
11	(((deep adj2 SSI*) or SSI*) adj2 deep).ab,ti.	1
12	(((Organ adj1 space SSI*) or space) adj1 Organ SSI*).ab,ti.	0
13	1 or 2 or 3 or 4 or 5 or 6 or 7 or 8 or 9 or 10 or 11 or 12	2,116
14	Smoking Cessation.mp. [mp=abstract, heading words, title]	343
15	Smoking Reduction.mp. [mp=abstract, heading words, title]	3
16	((quit* or stop* or ceas* or giv* or abstain* or abstinen* or Cessat* or reduc*) and (smok* or tobac co)).mp. [mp=abstract, heading words, title]	650
17	"Tobacco Use Cessation"/	0
18	"Tobacco Use Cessation Products"/	0
19	Nicotinic Agonists/	0
20	Smoking Cessation Agents/	0
21	Nicotine Chewing Gum/	0
22	NRT.ti,ab.	8
23	nicotine replacement.tw.	18
24	((nicotine or tobacco) adj2 (gum* or lozenge* or patch* or spray*)).tw.	11
25	(pharmaco* adj2 (nicotine or tobacco or smoking)).tw.	10

26	(ecigarette* or e cigarette*).ab,ti.	7
27	electronic cigarette*.ab,ti.	2
28	(electron* adj3 (cig* or nicotine*)).ab,ti.	5
29	(e cig* or ecig*).ab,ti.	7
30	(vape* or vaporizer* or vaporiser* or vapourizer* or vapouriser* or vaping*).ab,ti.	6
31	14 or 15 or 16 or 17 or 18 or 19 or 20 or 21 or 22 or 23 or 24 or 25 or 26 or 27 or 28 or 29 o r 30	665
32	13 and 31	21

S2.2.2. The second search strategy (the first updated search)

S2.2.2a. MEDLINE database via Ovid (year of 2021 to August 6th, 2021)

#	Searches	Results
1	exp Fractures, Bone/	192,917
2	Broken Bone$.ab,ti.	312
3	Bone Fracture$.ab,ti.	11,164
4	Spiral Fracture$.ab,ti.	358
5	Torsion Fracture$.ab,ti.	43
6	exp Fracture Fixation/	64,931
7	Fracture Fixation$.ab,ti.	3,598
8	Skeletal Fixation$.ab,ti.	553
9	Fracture Reduction$.ab,ti.	2,686
10	exp Fracture Healing/	14,083
11	(fractur$ adj3 healing).ab,ti.	10,561
12	(non?union or nonunion or un?united or ununited or delayed?union or union or malunion).ab,ti.	66,632
13	exp Surgical Wound Infection/	37,954
14	Surgical Wound Infection$.ab,ti.	1,345
15	Surgical Site Infection$.ab,ti.	12,338
16	Postoperative Wound Infection$.ab,ti.	1,681
17	(superficial adj2 SSI$).ab,ti.	400
18	(deep adj2 SSI$).ab,ti.	427
19	(Organ adj space SSI$).ab,ti.	280
20	1 or 2 or 3 or 4 or 5 or 6 or 7 or 8 or 9 or 10 or 11 or 12 or 13 or 14 or 15 or 16 or 17 or 18 or 19	303,900
21	exp Smoking Cessation/	30,268
22	exp Smoking Reduction/	90
23	((quit$ or stop$ or ceas$ or giv$ or abstain$ or abstinen$ or Cessat$ or reduc$) adj3 (smok$ or tobacco)).ti,ab.	48,336
24	"Tobacco Use Cessation"/	1,285
25	"Tobacco Use Cessation Products"/	2,065
26	Nicotinic Agonists/	7,515
27	Smoking Cessation Agents/	227
28	Nicotine Chewing Gum/	27
29	NRT.ti,ab.	2,237
30	nicotine replacement.tw,kf.	3,688
31	((nicotine or tobacco) adj2 (gum* or lozenge* or patch* or spray*)).tw,kf.	2,503

32	(pharmaco* adj2 (nicotine or tobacco or smoking)).tw,kf.	1,154
33	exp Vaping/	2,109
34	exp Electronic Nicotine Delivery Systems/	5,020
35	(Ecigarette$ or e-cigarette$).ab,ti.	5,442
36	Electronic-Cigarette$.ab,ti.	3,476
37	(electron* adj3 (cig$ or nicotine$)).ab,ti.	4,139
38	(e-cig$ or ecig$).ti,ab.	5,722
39	(vape$ or vaporizer$ or vaporiser$ or vapourizer$ or vapouriser$ or vaping$).ti,ab.	3,598
40	21 or 22 or 23 or 24 or 25 or 26 or 27 or 28 or 29 or 30 or 31 or 32 or 33 or 34 or 35 or 36 or 37 or 38 or 39	71,553
41	20 and 40	529
42	limit 41 to humans	453
43	limit 42 to yr="2021 -Current"	16

S2.2.2b. EMBASE database (year of 2021 to August 7th, 2021)

No.	Query	Results
#43	#41 AND 'human'/de AND [2021-2021]/py	55
#42	#41 AND 'human'/de	1,263
#41	#20 AND #40	1,408
#40	#21 OR #22 OR #23 OR #24 OR #25 OR #26 OR #27 OR #28 OR #29 OR #30 OR #31 OR #32 OR #33 OR #34 OR #35 OR #36 OR #37 OR #38 OR #39	118,655
#39	vape*:ab,ti OR vaporizer*:ab,ti OR vaporiser*:ab,ti OR vapourizer*:ab,ti OR vapouriser*:ab,ti OR vaping*:ab,ti	4,525
#38	e cig*':ab,ti OR ecig*:ab,ti	6,969
#37	(electron* NEAR/3 (cig* OR nicotine*)):ab,ti	4,943
#36	electronic cigarette*':ab,ti	4,199
#35	ecigarette*:ab,ti OR 'e cigarette*':ab,ti	6,590
#34	electronic cigarette'/exp	8,116
#33	vaping'/exp	3,322
#32	(pharmaco* NEAR/2 (nicotine OR tobacco OR smoking)):ab,ti	1,391
#31	((nicotine OR tobacco) NEAR/2 (gum* OR lozenge* OR patch* OR spray*)):ab,ti	3,027
#30	nicotine replacement':ab,ti	4,629
#29	nrt:ab,ti	2,940
#28	nicotine chewing gum'/exp	2,966
#27	smoking cessation agents'/exp	40
#26	nicotinic agonists'/exp	25,924
#25	tobacco use cessation products'/exp	2,966
#24	tobacco use cessation'/exp	62,328
#23	((quit* OR stop* OR ceas* OR giv* OR abstain* OR abstinen* OR cessat* OR reduc*) NEAR/3 (smok* OR tobacco)):ab,ti	63,027
#22	smoking reduction'/exp	297
#21	smoking cessation'/exp	62,328
#20	#1 OR #2 OR #3 OR #4 OR #5 OR #6 OR #7 OR #8 OR #9 OR #10 OR #11 OR #12 OR #13 OR #14 OR #15 OR #16 OR #17 OR #18 OR #19	494,723
#19	(organ NEAR/1 'space ssi*'):ab,ti	566
#18	(deep NEAR/2 ssi*):ab,ti	709
#17	(superficial NEAR/2 ssi*):ab,ti	727
#16	postoperative wound infection*':ab,ti	2,167

#15	surgical site infection*':ab,ti	18,307
#14	surgical wound infection*':ab,ti	1,740
#13	surgical infection'/exp	54,736
#12	non?union:ab,ti OR nonunion:ab,ti OR un?united:ab,ti OR ununited:ab,ti OR delayed?union:ab,ti OR union:ab,ti OR malunion:ab,ti	88,415
#11	(fractur* NEAR/3 healing):ab,ti	13,399
#10	fracture healing'/exp	55,249
#9	fracture reduction*':ab,ti	3,209
#8	skeletal fixation*':ab,ti	656
#7	fracture fixation*':ab,ti	4,059
#6	fracture fixation'/exp	97,671
#5	torsion fracture*':ab,ti	49
#4	spiral fracture*':ab,ti	420
#3	bone fracture*':ab,ti	15,462
#2	broken bone*':ab,ti	416
#1	fracture'/exp	348,494

S2.2.2c. Cochrane library database (year of 2021 to August 7th, 2021)

ID	Search	Hits
#1	MeSH descriptor: [Fractures, Bone] explode all trees	6,415
#2	(Broken Bone*):ti,ab,kw	184
#3	(Bone Fracture*):ti,ab,kw	11,248
#4	(Spiral Fracture*):ti,ab,kw	59
#5	(Torsion Fracture*):ti,ab,kw	54
#6	MeSH descriptor: [Fracture Fixation] explode all trees	1,829
#7	(Fracture Fixation*):ti,ab,kw	4,126
#8	(Skeletal Fixation*):ti,ab,kw	179
#9	(Fracture Reduction*):ti,ab,kw	4,176
#10	MeSH descriptor: [Fracture Healing] explode all trees	545
#11	((fractur* near/3 healing)):ti,ab,kw	1,594
#12	((non?union or nonunion or un?united or ununited or delayed?union or union or malunion)):ti,ab,kw	3,605
#13	MeSH descriptor: [Surgical Wound Infection] explode all trees	3,505
#14	(Surgical Wound Infection*):ti,ab,kw	7,420
#15	(Surgical Site Infection*):ti,ab,kw	3,903
#16	(Postoperative Wound Infection*):ti,ab,kw	5,826
#17	((superficial near/2 SSI*)):ti,ab,kw	122
#18	((deep near/2 SSI*)):ti,ab,kw	95
#19	((Organ near/1 space SSI*)):ti,ab,kw	91
#20	#1 or #2 or #3 or #4 or #5 or #6 or #7 or #8 or #9 or #10 or #11 or #12 or #13 or #14 or #15 or #16 or #17 or #18 or #19	28,087
#21	MeSH descriptor: [Smoking Cessation] explode all trees	4,217
#22	MeSH descriptor: [Smoking Reduction] explode all trees	27
#23	((quit* or stop* or ceas* or giv* or abstain* or abstinen* or Cessat* or reduc*) NEAR/3 (smoke* or tobacco)):ti,ab,kw	5,304
#24	MeSH descriptor: [Tobacco Use Cessation Devices] explode all trees	571
#25	MeSH descriptor: [Nicotine Chewing Gum] explode all trees	16
#26	MeSH descriptor: [Nicotinic Agonists] explode all trees	748

#27	(Tobacco Use Cessation Products):ti,ab,kw	296
#28	(Nicotine near/2 patch*):ti,ab,kw	1,316
#29	(Nicotine near/2 delivery device*):ti,ab,kw	106
#30	(nicotine near/2 nasal spray*):ti,ab,kw	97
#31	(smoking near/2 cessation product*):ti,ab,kw	659
#32	(nicotine near/2 replacement):ti,ab,kw	1,871
#33	(nicotine near/2 lozenge*):ti,ab,kw	181
#34	(nicotine polacrilex):ti,ab,kw	50
#35	(nicotine near/2 inhal*):ti,ab,kw	151
#36	(nicotine near/2 chewing gum*):ti,ab,kw	142
#37	nicorette:ti,ab,kw	97
#38	(nicotinic near/2 agonist*):ti,ab,kw	840
#39	(nicotine near/2 tablet*):ti,ab,kw	41
#40	(nicotine near/2 sublingual):ti,ab,kw	25
#41	NRT:ti,ab,kw	954
#42	(nicotine near/3 therap*):ti,ab,kw	2,079
#43	MeSH descriptor: [Vaping] explode all trees	60
#44	MeSH descriptor: [Electronic Nicotine Delivery Systems] explode all trees	162
#45	(Ecigarette* or e-cigarette*):ti,ab,kw	584
#46	(Electronic-Cigarette*):ti,ab,kw	463
#47	((electron* NEAR/3 (cig* OR nicotine*))):ti,ab,kw	571
#48	(e-cig* or ecig*):ti,ab,kw	628
#49	(vape* or vaporizer* or vaporiser* or vapourizer* or vapouriser* or vaping*):ti,ab,kw	2,394
#50	#21 or #22 or #23 or #24 or #25 or #26 or #27 or #28 or #29 or #30 or #31 or #32 or #33 or #34 or #35 or #36 or #37 or #38 or #39 or #40 or #41 or #42 or #43 or #44 or #45 or #46 or #47 or #48 or #49	12,110
#51	#20 and #50 with Publication Year from 2021 to 2021, in Trials	1

S2.2.2d. CINAHL database (year of 2021 to August 7th, 2021)

#	Query	Results
S31	S15 and S30 (Limiters - Published Date: 20210401-20210831)	8
S30	S16 or S17 or S18 or S19 or S20 or S21 or S22 or S23 or S24 or S25 or S26 or S27 or S28 or S 29	32,207
S29	TI (vape$ or vaporizer* or vaporiser* or vapourizer* or vapouriser* or vaping*) OR AB (vape$ or va porizer* or vaporiser* or vapourizer* or vapouriser* or vaping*)	1,718
S28	TI (e-cig* or ecig*) OR AB (e-cig* or ecig*)	3,922
S27	TI (electron* W3 (cig* or nicotine*)) OR AB (electron* W3 (cig* or nicotine*))	2,625
S26	TI Electronic-Cigarette* OR AB Electronic-Cigarette*	2,243
S25	TI (Ecigarette* OR e-cigarette*) OR AB (Ecigarette* OR e-cigarette*)	3,779
S24	(MM "Electronic Cigarettes")	1,992
S23	(MM "Vaping")	228
S22	TI (nicotine and (gum* or lozenge* or spray* or patch*)) OR AB (nicotine and (gum* or lozenge* o r spray* or patch*))	1,160
S21	TI nicotine replacement OR AB nicotine replacement	1,872
S20	TI NRT OR AB NRT	970
S19	(MH “Nicotine Chewing Gum” OR (MH “Nicotine Patch”)	456
S18	(MH (“Nicotine Replacement Therapy”) OR (MH “Tobacco Use Cessation Products+”) OR (MH “Nico tinic Agonists+”)	11,179

S17	TI ((quit* or stop* or ceas* or giv* or abstain* or abstinen* or Cessat* or reduc*) W3 (smok* or to bacco)) OR AB ((quit* or stop* or ceas* or giv* or abstain* or abstinen* or Cessat* or reduc*) W3 (smok* or tobacco))	13,671
S16	(MM "Smoking Cessation")	14,364
S15	S1 or S2 or S3 or S4 or S5 or S6 or S7 or S8 or S9 or S10 or S11 or S12 or S13 or S14	51,061
S14	TI Organ w1 space SSI* or AB Organ w1 space SSI*	111
S13	TI deep w2 SSI* or AB deep w2 SSI*	168
S12	TI superficial w2 SSI* or AB superficial w2 SSI*	160
S11	TI Postoperative Wound Infection* or AB Postoperative Wound Infection*	319
S10	TI Surgical Site Infection* or AB Surgical Site Infection*	5,925
S9	TI Surgical Wound Infection* or AB Surgical Wound Infection*	352
S8	(MM "Surgical Wound Infection")	7,563
S7	TI (non?union or nonunion or un?united or ununited or delayed?union or union or malunion) OR A B (non?union or nonunion or un?united or ununited or delayed?union or union or malunion)	23,757
S6	TI fractur* w3 healing OR AB fractur* w3 healing	2,083
S5	(MM "Fracture Healing")	1,801
S4	TI Fracture Fixation* or AB Fracture Fixation* or TI Skeletal Fixation* or AB Skeletal Fixation* or TI Fracture Reduction* or AB Fracture Reduction*	2,369
S3	(MH "Fracture Fixation+")	16,321
S2	TI Broken Bone* or AB Broken Bone* or TI Bone Fracture* or AB Bone Fracture* or TI Spiral Fra cture* or AB Spiral Fracture* or TI Torsion Fracture* or AB Torsion Fracture*	2,810
S1	(MM "Carpal Fractures")	119

S2.2.2e. AMED database (year of 2021 to August 7th, 2021)

#	Searches	Results
1	exp Fracture Fixation/	843
2	(Broken Bone* or Bone Fracture* or Spiral Fracture* or Torsion Fracture*).ab,ti.	147
3	(Fracture Fixation* or Skeletal Fixation* or Fracture Reduction*).ab,ti	158
4	exp Fracture Healing/	92
5	(((fractur* adj3 healing) or healing) adj3 fractur*).ab,ti.	214
6	(non?union or nonunion or un?united or ununited or delayed?union or union or malunion).ab,ti.	1,075
7	Surgical Wound Infection*.ab,ti.	1
8	Surgical Surgical Site Infection*.ab,ti.Site Infection*.ab,ti	30
9	Postoperative Wound Infection*.ab,ti.	10
10	(((superficial adj2 SSI*) or SSI*) adj2 superficial).ab,ti.	1
11	(((deep adj2 SSI*) or SSI*) adj2 deep).ab,ti.	1
12	(((Organ adj1 space SSI*) or space) adj1 Organ SSI*).ab,ti.	0
13	1 or 2 or 3 or 4 or 5 or 6 or 7 or 8 or 9 or 10 or 11 or 12	2,167
14	Smoking Cessation.mp. [mp=abstract, heading words, title]	344
15	Smoking Reduction.mp. [mp=abstract, heading words, title]	3
16	((quit* or stop* or ceas* or giv* or abstain* or abstinen* or Cessat* or reduc*) and (smok* or tobac co)).mp. [mp=abstract, heading words, title]	652
17	"Tobacco Use Cessation"/	0
18	"Tobacco Use Cessation Products"/	0
19	Nicotinic Agonists/	0
20	Smoking Cessation Agents/	0
21	Nicotine Chewing Gum/	0

22	NRT.ti,ab.	8
23	nicotine replacement.tw.	18
24	((nicotine or tobacco) adj2 (gum* or lozenge* or patch* or spray*)).tw.	11
25	(pharmaco* adj2 (nicotine or tobacco or smoking)).tw.	10
26	(ecigarette* or e cigarette*).ab,ti.	7
27	electronic cigarette*.ab,ti.	2
28	(electron* adj3 (cig* or nicotine*)).ab,ti.	5
29	(e cig* or ecig*).ab,ti.	7
30	(vape* or vaporizer* or vaporiser* or vapourizer* or vapouriser* or vaping*).ab,ti.	6
31	14 or 15 or 16 or 17 or 18 or 19 or 20 or 21 or 22 or 23 or 24 or 25 or 26 or 27 or 28 or 29 or 30	667
32	13 and 31	21
33	limit 32 to yr="2021-Current"	0

S2.3. Search strategies for the impacts of preoperative smoking cessation time, NRT, and vaping on bone healing

S2.3.1. The first search strategy

S2.3.1a. MEDLINE database via Ovid (inception of database to April 23rd, 2021)

#	Searches	Results
1	exp Smoking Cessation/	29,685
2	exp Smoking Reduction/	82
3	((quit$ or stop$ or ceas$ or giv$ or abstain$ or abstinen$ or Cessat$ or reduc$) adj3 (smok$ or tobacco)).ti,ab.	47,550
4	"Tobacco Use Cessation"/	1,241
5	"Tobacco Use Cessation Products"/	1,970
6	Nicotinic Agonists/	7,440
7	Smoking Cessation Agents/	211
8	Nicotine Chewing Gum/	26
9	NRT.ti,ab.	2,182
10	nicotine replacement.tw,kf.	3,608
11	((nicotine or tobacco) adj2 (gum* or lozenge* or patch* or spray*)).tw,kf.	2,483
12	(pharmaco* adj2 (nicotine or tobacco or smoking)).tw,kf.	1,138
13	exp Vaping/	1,670
14	exp Electronic Nicotine Delivery Systems/	4,363
15	(Ecigarette$ or e-cigarette$).ab,ti.	5,142
16	Electronic-Cigarette$.ab,ti.	3,322
17	(electron* adj3 (cig$ or nicotine$)).ab,ti.	3,935
18	(e-cig$ or ecig$).ti,ab.	5,407
19	(vape$ or vaporizer$ or vaporiser$ or vapourizer$ or vapouriser$ or vaping$).ti,ab.	3,378
20	1 or 2 or 3 or 4 or 5 or 6 or 7 or 8 or 9 or 10 or 11 or 12 or 13 or 14 or 15 or 16 or 17 or 18 or 19	70,208
21	fusion.ab,ti.	205,233
22	exp Spinal Fusion/	26,584
23	interbody.ab,ti.	6,774
24	Spondylodes*.ab,ti.	748

25	21 or 22 or 23 or 24	214,524
26	exp Osteotomy/	36,408
27	Osteotomy,Le Fort/	2,242
28	gonarthrosis$.tw.	1,070
29	osteotom$.tw.	34,700
30	26 or 27 or 28 or 29	50,185
31	exp Arthrodesis/	35,822
32	Arthrodes*.ab,ti.	12,634
33	31 or 32	40,346
34	25 or 30 or 33	271,936
35	20 and 34	111

S2.3.1b. EMBASE database (inception of database to April 24th, 2021)

No.	Query	Results
#34	#20 AND #33	214
#33	#25 OR #29 OR #32	330,752
#32	#30 OR #31	52,605
#31	arthrodes*:ab,ti	15,260
#30	'arthrodesis'/exp	48,953
#29	#26 OR #27 OR #28	58,834
#28	'osteotomy'/exp	49,720
#27	osteotom*:ab,ti	40,830
#26	gonarthrosis*:ab,ti	1,605
#25	#21 OR #22 OR #23 OR #24	261,726
#24	spondylodes*:ab,ti	1,021
#23	interbody:ab,ti	8,580
#22	'spine fusion'/exp	33,068
#21	fusion:ab,ti	252,103
#20	#1 OR #2 OR #3 OR #4 OR #5 OR #6 OR #7 OR #8 OR #9 OR #10 OR #11 OR #12 OR #13 OR #14 OR #15 OR #16 OR #17 OR #18 OR #19	116,591
#19	vape*:ab,ti OR vaporizer*:ab,ti OR vaporiser*:ab,ti OR vapourizer*:ab,ti OR vapouriser*:ab,ti OR vaping*:ab,ti	4,229
#18	'e cig*':ab,ti OR ecig*:ab,ti	6,596
#17	(electron* NEAR/3 (cig* OR nicotine*)):ab,ti	4,720
#16	'electronic cigarette*':ab,ti	4,023
#15	ecigarette*:ab,ti OR 'e cigarette*':ab,ti	6,234
#14	'electronic cigarette'/exp	7,737
#13	'vaping'/exp	2,989
#12	(pharmaco* NEAR/2 (nicotine OR tobacco OR smoking)):ab,ti	1,381
#11	((nicotine OR tobacco) NEAR/2 (gum* OR lozenge* OR patch* OR spray*)):ab,ti	2,997
#10	'nicotine replacement':ab,ti	4,564
#9	nrt:ab,ti	2,890
#8	'nicotine chewing gum'/exp	2,956
#7	'smoking cessation agents'/exp	36
#6	'nicotinic agonists'/exp	25,417
#5	'tobacco use cessation products'/exp	2,956

#4	'tobacco use cessation'/exp	61,602
#3	((quit* OR stop* OR ceas* OR giv* OR abstain* OR abstinen* OR cessat* OR reduc*) NEAR/3 (smok* OR tobacco)):ab,ti	62,187
#2	'smoking reduction'/exp	278
#1	'smoking cessation'/exp	61,602

S2.3.1c. Cochrane library database (inception of database to April 24th, 2021)

ID	Search	Hits
#1	MeSH descriptor: [Smoking Cessation] explode all trees	4,145
#2	MeSH descriptor: [Smoking Reduction] explode all trees	25
#3	((quit* or stop* or ceas* or giv* or abstain* or abstinen* or Cessat* or reduc*) NEAR/3 (smoke* or tobacco)):ti,ab,kw	5,174
#4	MeSH descriptor: [Tobacco Use Cessation Devices] explode all trees	550
#5	MeSH descriptor: [Nicotine Chewing Gum] explode all trees	14
#6	MeSH descriptor: [Nicotinic Agonists] explode all trees	741
#7	(Tobacco Use Cessation Products):ti,ab,kw	287
#8	(Nicotine near/2 patch*):ti,ab,kw	1,299
#9	(Nicotine near/2 delivery device*):ti,ab,kw	103
#10	(nicotine near/2 nasal spray*):ti,ab,kw	97
#11	(smoking near/2 cessation product*):ti,ab,kw	639
#12	(nicotine near/2 replacement):ti,ab,kw	1,837
#13	(nicotine near/2 lozenge*):ti,ab,kw	177
#14	(nicotine polacrilex):ti,ab,kw	50
#15	(nicotine near/2 inhal*):ti,ab,kw	150
#16	(nicotine near/2 chewing gum*):ti,ab,kw	140
#17	nicorette:ti,ab,kw	97
#18	(nicotinic near/2 agonist*):ti,ab,kw	831
#19	(nicotine near/2 tablet*):ti,ab,kw	41
#20	(nicotine near/2 sublingual):ti,ab,kw	25
#21	NRT:ti,ab,kw	926
#22	(nicotine near/3 therap*):ti,ab,kw	2,045
#23	MeSH descriptor: [Vaping] explode all trees	52
#24	MeSH descriptor: [Electronic Nicotine Delivery Systems] explode all trees	148
#25	(Ecigarette* or e-cigarette*):ti,ab,kw	542
#26	(Electronic-Cigarette*):ti,ab,kw	441
#27	((electron* NEAR/3 (cig* OR nicotine*))):ti,ab,kw	543
#28	(e-cig* or ecig*):ti,ab,kw	587
#29	(vape* or vaporizer* or vaporiser* or vapourizer* or vapouriser* or vaping*):ti,ab,kw	2,341
#30	#1 or #2 or #3 or #4 or #5 or #6 or #7 or #8 or #9 or #10 or #11 or #12 or #13 or #14 or #15 or #16 or #17 or #18 or #19 or #20 or #21 or #22 or #23 or #24 or #25 or #26 or #27 or #28 or #29	11,862
#31	fusion:ti,ab,kw	7,274
#32	MeSH descriptor: [Spinal Fusion] explode all trees	963
#33	Interbody:ti,ab,kw	819
#34	(Spondylodesis or Spondylodeses):ti,ab,kw	57
#35	#31 or #32 or #33 or #34	7,306
#36	MeSH descriptor: [Osteotomy] explode all trees	767

#37	osteotom*:ti,ab,kw	2,063
#38	#36 or #37	2,162
#39	MeSH descriptor: [Arthrodesis] explode all trees	1,035
#40	Arthrodes*:ti,ab,kw	495
#41	#39 or #40	1,382
#42	#35 or #38 or #41	9,609
#43	#30 and #42 in Trials	13

S2.3.1d. CINAHL database (inception of database to April 24th, 2021)

#	Query	Results
S29	S15 and S28	35
S28	S20 or S24 or S27	35,349
S27	S25 or S26	4,686
S26	TI (Arthrodes*) OR AB (Arthrodes*)	3,938
S25	(MH "Arthrodesis")	2,883
S24	S21 or S22 or S23	9,071
S23	TX (osteotom$)	10
S22	TX (gonarthrosis$)	412
S21	(MH "Osteotomy")	8,711
S20	S16 or S17 or S18 or S19	24,067
S19	TI (Spondylodes*) OR AB (Spondylodes*)	77
S18	TI (interbody) OR AB (interbody)	2,272
S17	(MH "Spinal Fusion")	10,181
S16	TI (fusion) OR AB (fusion)	20,583
S15	S1 or S2 or S3 or S4 or S5 or S6 or S7 or S8 or S9 or S10 or S11 or S12 or S13 or S14	31,638
S14	TI (vape$ or vaporizer* or vaporiser* or vapourizer* or vapouriser* or vaping*) OR AB (vape$ or vaporizer* or vaporiser* or vapourizer* or vapouriser* or vaping*)	1,625
S13	TI (e-cig* or ecig*) OR AB (e-cig* or ecig*)	3,757
S12	TI (electron* W3 (cig* or nicotine*)) OR AB (electron* W3 (cig* or nicotine*))	2,538
S11	TI Electronic-Cigarette* OR AB Electronic-Cigarette*	2,179
S10	TI (Ecigarette* OR e-cigarette*) OR AB (Ecigarette* OR e-cigarette*)	3,615
S9	(MM "Electronic Cigarettes")	1,919
S8	(MM "Vaping")	197
S7	TI (nicotine and (gum* or lozenge* or spray* or patch*)) OR AB (nicotine and (gum* or lozenge* or spray* or patch*))	1,150
S6	TI nicotine replacement OR AB nicotine replacement	1,836
S5	TI NRT OR AB NRT	947
S4	(MH “Nicotine Chewing Gum”) OR (MH “Nicotine Patch”)	454
S3	(MH “Nicotine Replacement Therapy”) OR (MH “Tobacco Use Cessation Products+”) OR (MH “Nicotinic Agonists+”)	10,955
S2	TI ((quit* or stop* or ceas* or giv* or abstain* or abstinen* or Cessat* or reduc*) W3 (smok* or tobacco)) OR AB ((quit* or stop* or ceas* or giv* or abstain* or abstinen* or Cessat* or reduc*) W3 (smok* or tobacco))	13,456
S1	(MM "Smoking Cessation")	14,099

S2.3.1e. AMED database (inception of database to April 27th, 2021)

#	Searches	Results
1	Smoking Cessation.mp. [mp=abstract, heading words, title]	343
2	Smoking Reduction.mp. [mp=abstract, heading words, title]	3
3	((quit* or stop* or ceas* or giv* or abstain* or abstinen* or Cessat* or reduc*) and (smok* or tobacco)).mp. [mp=abstract, heading words, title]	650
4	"Tobacco Use Cessation"/	0
5	"Tobacco Use Cessation Products"/	0
6	Nicotinic Agonists/	0
7	Smoking Cessation Agents/	0
8	Nicotine Chewing Gum/	0
9	NRT.ti,ab.	8
10	nicotine replacement.tw.	18
11	((nicotine or tobacco) adj2 (gum* or lozenge* or patch* or spray*)).tw.	11
12	(pharmaco* adj2 (nicotine or tobacco or smoking)).tw.	10
13	(ecigarette* or e cigarette*).ab,ti.	7
14	electronic cigarette*.ab,ti.	2
15	(electron* adj3 (cig* or nicotine*)).ab,ti.	5
16	(e cig* or ecig*).ab,ti.	7
17	(vape* or vaporizer* or vaporiser* or vapourizer* or vapouriser* or vaping*).ab,ti.	6
18	1 or 2 or 3 or 4 or 5 or 6 or 7 or 8 or 9 or 10 or 11 or 12 or 13 or 14 or 15 or 16 or 17	665
19	fusion.ab,ti.	1,122
20	exp Spinal Fusion/	202
21	interbody.ab,ti.	82
22	Spondylodes*.ab,ti.	12
23	19 or 20 or 21 or 22	1,174
24	exp Osteotomy/	1,020
25	Osteotomy,Le Fort/	0
26	gonarthrosis$.tw.	41
27	osteotom$.tw.	1,446
28	24 or 25 or 26 or 27	1,484
29	exp Arthrodesis/	1,242
30	Arthrodes*.ab,ti.	1,306
31	29 or 30	1,635
32	23 or 28 or 31	3,431
33	18 and 32	11

S2.3.2. The second search strategy (the first updated search)

S2.3.2a. MEDLINE database via Ovid (year of 2021 to August 6th, 2021)

#	Searches	Results
1	exp Smoking Cessation/	30,268
2	exp Smoking Reduction/	90
3	((quit$ or stop$ or ceas$ or giv$ or abstain$ or abstinen$ or Cessat$ or reduc$) adj3 (smok$ or tobacco)).ti,ab.	48,336
4	"Tobacco Use Cessation"/	1,285
5	"Tobacco Use Cessation Products"/	2,065

6	Nicotinic Agonists/	7,515
7	Smoking Cessation Agents/	227
8	Nicotine Chewing Gum/	27
9	NRT.ti,ab.	2,237
10	nicotine replacement.tw,kf.	3,688
11	((nicotine or tobacco) adj2 (gum* or lozenge* or patch* or spray*)).tw,kf.	2,503
12	(pharmaco* adj2 (nicotine or tobacco or smoking)).tw,kf.	1,154
13	exp Vaping/	2,109
14	exp Electronic Nicotine Delivery Systems/	5,020
15	(Ecigarette$ or e-cigarette$).ab,ti.	5,442
16	Electronic-Cigarette$.ab,ti.	3,476
17	(electron* adj3 (cig$ or nicotine$)).ab,ti.	4,139
18	(e-cig$ or ecig$).ti,ab.	5,722
19	(vape$ or vaporizer$ or vaporiser$ or vapourizer$ or vapouriser$ or vaping$).ti,ab.	3,598
20	1 or 2 or 3 or 4 or 5 or 6 or 7 or 8 or 9 or 10 or 11 or 12 or 13 or 14 or 15 or 16 or 17 or 18 or 19	71,553
21	fusion.ab,ti.	208,898
22	exp Spinal Fusion/	27,565
23	interbody.ab,ti.	6,984
24	Spondylodes*.ab,ti.	752
25	21 or 22 or 23 or 24	218,416
26	exp Osteotomy/	37,009
27	Osteotomy,Le Fort/	2,282
28	gonarthrosis$.tw.	1,075
29	osteotom$.tw.	35,309
30	26 or 27 or 28 or 29	50,931
31	exp Arthrodesis/	36,942
32	Arthrodes*.ab,ti.	12,841
33	31 or 32	41,520
34	25 or 30 or 33	276,640
35	20 and 34	115
36	limit 35 to yr="2021 -Current"	9

S2.3.2b. EMBASE database (year of 2021 to August 7th, 2021)

No.	Query	Results
#35	#20 AND #33 AND [2021-2021]/py	15
#34	#20 AND #33	218
#33	#25 OR #29 OR #32	335,959
#32	#30 OR #31	53,558
#31	arthrodes*:ab,ti	15,434
#30	'arthrodesis'/exp	49,888
#29	#26 OR #27 OR #28	59,753
#28	'osteotomy'/exp	50,526
#27	osteotom*:ab,ti	41,419

#26	gonarthrosis*:ab,ti	1,614
#25	#21 OR #22 OR #23 OR #24	265,925
#24	spondylodes*:ab,ti	1,025
#23	interbody:ab,ti	8,762
#22	'spine fusion'/exp	33,760
#21	fusion:ab,ti	256,107
#20	#1 OR #2 OR #3 OR #4 OR #5 OR #6 OR #7 OR #8 OR #9 OR #10 OR #11 OR #12 OR #13 OR #14 OR #15 OR #16 OR #17 OR #18 OR #19	118,655
#19	vape*:ab,ti OR vaporizer*:ab,ti OR vaporiser*:ab,ti OR vapourizer*:ab,ti OR vapouriser*:ab,ti OR vaping*:ab,ti	4,525
#18	'e cig*':ab,ti OR ecig*:ab,ti	6,969
#17	(electron* NEAR/3 (cig* OR nicotine*)):ab,ti	4,943
#16	'electronic cigarette*':ab,ti	4,199
#15	ecigarette*:ab,ti OR 'e cigarette*':ab,ti	6,590
#14	'electronic cigarette'/exp	8,116
#13	'vaping'/exp	3,322
#12	(pharmaco* NEAR/2 (nicotine OR tobacco OR smoking)):ab,ti	1,391
#11	((nicotine OR tobacco) NEAR/2 (gum* OR lozenge* OR patch* OR spray*)):ab,ti	3,027
#10	'nicotine replacement':ab,ti	4,629
#9	nrt:ab,ti	2,940
#8	'nicotine chewing gum'/exp	2,966
#7	'smoking cessation agents'/exp	40
#6	'nicotinic agonists'/exp	25,924
#5	'tobacco use cessation products'/exp	2,966
#4	'tobacco use cessation'/exp	62,328
#3	((quit* OR stop* OR ceas* OR giv* OR abstain* OR abstinen* OR cessat* OR reduc*) NEAR/3 (smok* OR tobacco)):ab,ti	63,027
#2	'smoking reduction'/exp	297
#1	'smoking cessation'/exp	62,328

S2.3.2c. Cochrane library database (year of 2021 to August 7th, 2021)

ID	Search	Hits
#1	MeSH descriptor: [Smoking Cessation] explode all trees	4,217
#2	MeSH descriptor: [Smoking Reduction] explode all trees	27
#3	((quit* or stop* or ceas* or giv* or abstain* or abstinen* or Cessat* or reduc*) NEAR/3 (smoke* or tobacco)):ti,ab,kw	5,304
#4	MeSH descriptor: [Tobacco Use Cessation Devices] explode all trees	571
#5	MeSH descriptor: [Nicotine Chewing Gum] explode all trees	16
#6	MeSH descriptor: [Nicotinic Agonists] explode all trees	748
#7	(Tobacco Use Cessation Products):ti,ab,kw	296
#8	(Nicotine near/2 patch*):ti,ab,kw	1,316
#9	(Nicotine near/2 delivery device*):ti,ab,kw	106
#10	(nicotine near/2 nasal spray*):ti,ab,kw	97
#11	(smoking near/2 cessation product*):ti,ab,kw	659
#12	(nicotine near/2 replacement):ti,ab,kw	1,871
#13	(nicotine near/2 lozenge*):ti,ab,kw	181
#14	(nicotine polacrilex):ti,ab,kw	50

#15	(nicotine near/2 inhal*):ti,ab,kw	151
#16	(nicotine near/2 chewing gum*):ti,ab,kw	142
#17	nicorette:ti,ab,kw	97
#18	(nicotinic near/2 agonist*):ti,ab,kw	840
#19	(nicotine near/2 tablet*):ti,ab,kw	41
#20	(nicotine near/2 sublingual):ti,ab,kw	25
#21	NRT:ti,ab,kw	954
#22	(nicotine near/3 therap*):ti,ab,kw	2,079
#23	MeSH descriptor: [Vaping] explode all trees	60
#24	MeSH descriptor: [Electronic Nicotine Delivery Systems] explode all trees	162
#25	(Ecigarette* or e-cigarette*):ti,ab,kw	584
#26	(Electronic-Cigarette*):ti,ab,kw	463
#27	((electron* NEAR/3 (cig* OR nicotine*))):ti,ab,kw	571
#28	(e-cig* or ecig*):ti,ab,kw	628
#29	(vape* or vaporizer* or vaporiser* or vapourizer* or vapouriser* or vaping*):ti,ab,kw	2,394
#30	#1 or #2 or #3 or #4 or #5 or #6 or #7 or #8 or #9 or #10 or #11 or #12 or #13 or #14 or #15 or #16 or #17 or #18 or #19 or #20 or #21 or #22 or #23 or #24 or #25 or #26 or #27 or #28 or #29	12,110
#31	fusion:ti,ab,kw	7,497
#32	MeSH descriptor: [Spinal Fusion] explode all trees	988
#33	Interbody:ti,ab,kw	835
#34	(Spondylodesis or Spondylodeses):ti,ab,kw	60
#35	#31 or #32 or #33 or #34	7,531
#36	MeSH descriptor: [Osteotomy] explode all trees	784
#37	osteotom*:ti,ab,kw	2,125
#38	#36 or #37	2,226
#39	MeSH descriptor: [Arthrodesis] explode all trees	1,062
#40	Arthrodes*:ti,ab,kw	506
#41	#39 or #40	1,417
#42	#35 or #38 or #41	9,896
#43	#30 and #42 with Publication Year from 2021 to 2021, in Trials	0

S2.3.2d. CINAHL database (year of 2021 to August 7th, 2021)

#	Query	Results
S29	S15 and S28 (Published Date: 20210401-20210831)	1
S28	S20 or S24 or S27	36,525
S27	S25 or S26	4,775
S26	TI (Arthrodes*) OR AB (Arthrodes*)	4,019
S25	(MH "Arthrodesis")	2,940
S24	S21 or S22 or S23	9,317
S23	TX (osteotom$)	11
S22	TX (gonarthrosis$)	419
S21	(MH "Osteotomy")	8,949
S20	S16 or S17 or S18 or S19	24,982
S19	TI (Spondylodes*) OR AB (Spondylodes*)	81
S18	TI (interbody) OR AB (interbody)	2,338

S17	(MH "Spinal Fusion")	10,554
S16	TI (fusion) OR AB (fusion)	21,461
S15	S1 or S2 or S3 or S4 or S5 or S6 or S7 or S8 or S9 or S10 or S11 or S12 or S13 or S14	32,207
S14	TI (vape$ or vaporizer* or vaporiser* or vapourizer* or vapouriser* or vaping*) OR AB (vape$ or vaporizer* or vaporiser* or vapourizer* or vapouriser* or vaping*)	1,718
S13	TI (e-cig* or ecig*) OR AB (e-cig* or ecig*)	3,922
S12	TI (electron* W3 (cig* or nicotine*)) OR AB (electron* W3 (cig* or nicotine*))	2,625
S11	TI Electronic-Cigarette* OR AB Electronic-Cigarette*	2,243
S10	TI (Ecigarette* OR e-cigarette*) OR AB (Ecigarette* OR e-cigarette*)	3,779
S9	(MM "Electronic Cigarettes")	1,992
S8	(MM "Vaping")	228
S7	TI (nicotine and (gum* or lozenge* or spray* or patch*)) OR AB (nicotine and (gum* or lozenge* or spray* or patch*))	1,160
S6	TI nicotine replacement OR AB nicotine replacement	1,872
S5	TI NRT OR AB NRT	970
S4	(MH “Nicotine Chewing Gum”) OR (MH “Nicotine Patch”)	456
S3	(MH “Nicotine Replacement Therapy”) OR (MH “Tobacco Use Cessation Products+”) OR (MH “Nicotinic Agonists+”)	11,179
S2	TI ((quit* or stop* or ceas* or giv* or abstain* or abstinen* or Cessat* or reduc*) W3 (smok* or tobacco)) OR AB ((quit* or stop* or ceas* or giv* or abstain* or abstinen* or Cessat* or reduc*) W3 (smok* or tobacco))	13,671
S1	(MM "Smoking Cessation")	14,364

S2.3.2e. AMED database (year of 2021 to August 9th, 2021)

#	Searches	Results
1	Smoking Cessation.mp. [mp=abstract, heading words, title]	344
2	Smoking Reduction.mp. [mp=abstract, heading words, title]	3
3	((quit* or stop* or ceas* or giv* or abstain* or abstinen* or Cessat* or reduc*) and (smok* or tobacco)).mp. [mp=abstract, heading words, title]	652
4	"Tobacco Use Cessation"/	0
5	"Tobacco Use Cessation Products"/	0
6	Nicotinic Agonists/	0
7	Smoking Cessation Agents/	0
8	Nicotine Chewing Gum/	0
9	NRT.ti,ab.	8
10	nicotine replacement.tw.	18
11	((nicotine or tobacco) adj2 (gum* or lozenge* or patch* or spray*)).tw.	11
12	(pharmaco* adj2 (nicotine or tobacco or smoking)).tw.	10
13	(ecigarette* or e cigarette*).ab,ti.	7
14	electronic cigarette*.ab,ti.	2
15	(electron* adj3 (cig* or nicotine*)).ab,ti.	5
16	(e cig* or ecig*).ab,ti.	7
17	(vape* or vaporizer* or vaporiser* or vapourizer* or vapouriser* or vaping*).ab,ti.	6
18	1 or 2 or 3 or 4 or 5 or 6 or 7 or 8 or 9 or 10 or 11 or 12 or 13 or 14 or 15 or 16 or 17	667
19	fusion.ab,ti.	1,143
20	exp Spinal Fusion/	204
21	interbody.ab,ti.	82

22	Spondylodes*.ab,ti.	12
23	19 or 20 or 21 or 22	1,195
24	exp Osteotomy/	1,032
25	Osteotomy,Le Fort/	0
26	gonarthrosis$.tw.	41
27	osteotom$.tw.	1,471
28	24 or 25 or 26 or 27	1,509
29	exp Arthrodesis/	1,254
30	Arthrodes*.ab,ti.	1,330
31	29 or 30	1,661
32	23 or 28 or 31	3,485
33	18 and 32	11
34	limit 33 to yr="2021-Current"	0

S2.4. Search strategies for the impacts of preoperative smoking cessation time, NRT, and vaping on wound healing and wound complications after surgery

S2.4.1. The first search strategy

S2.4.1a. MEDLINE database (inception of database to April 23rd, 2021)

#	Searches	Results
1	exp Smoking Cessation/	29,685
2	exp Smoking Reduction/	82
3	((quit$ or stop$ or ceas$ or giv$ or abstain$ or abstinen$ or Cessat$ or reduc$) adj3 (smok$ or tobacco)).ti,ab.	47,550
4	"Tobacco Use Cessation"/	1,241
5	"Tobacco Use Cessation Products"/	1,970
6	Nicotinic Agonists/	7,440
7	Smoking Cessation Agents/	211
8	Nicotine Chewing Gum/	26
9	NRT.ti,ab.	2,182
10	nicotine replacement.tw,kf.	3,608
11	((nicotine or tobacco) adj2 (gum* or lozenge* or patch* or spray*)).tw,kf.	2,483
12	(pharmaco* adj2 (nicotine or tobacco or smoking)).tw,kf.	1,138
13	exp Vaping/	1,670
14	exp Electronic Nicotine Delivery Systems/	4,363
15	(Ecigarette$ or e-cigarette$).ab,ti.	5,142
16	Electronic-Cigarette$.ab,ti.	3,322
17	(electron* adj3 (cig$ or nicotine$)).ab,ti.	3,935
18	(e-cig$ or ecig$).ti,ab.	5,407
19	(vape$ or vaporizer$ or vaporiser$ or vapourizer$ or vapouriser$ or vaping$).ti,ab.	3,378
20	1 or 2 or 3 or 4 or 5 or 6 or 7 or 8 or 9 or 10 or 11 or 12 or 13 or 14 or 15 or 16 or 17 or 18 or 19	70,208
21	exp Surgical Wound Infection/	37,363
22	exp Surgical Wound Dehiscence/	7,530
23	(surg* adj5 infect*).tw.	36,669

24	(surg* adj5 wound*).tw.	16,551
25	(surg* adj5 site*).tw.	27,165
26	(surg* adj5 incision*).tw.	11,976
27	(surg* adj5 dehisc*).tw.	1,288
28	(wound* adj5 dehisc*).tw.	5,569
29	(wound* adj5 infect*).tw.	36,530
30	(wound adj5 disrupt*).tw.	657
31	wound complication*.tw.	6,174
32	21 or 22 or 23 or 24 or 25 or 26 or 27 or 28 or 29 or 30 or 31	131,927
33	20 and 32	227

S2.4.1b. EMBASE database (inception of database to April 24th, 2021)

No.	Query	Results
#33	#20 AND #32	459
#32	#21 OR #22 OR #23 OR #24 OR #25 OR #26 OR #27 OR #28 OR #29 OR #30 OR #31	189,023
#31	'wound complication*':ab,ti	8,673
#30	(wound NEAR/5 disrupt*):ab,ti	994
#29	(wound* NEAR/5 infect*):ab,ti	50,584
#28	(wound* NEAR/5 dehisc*):ab,ti	7,798
#27	(surg* NEAR/5 dehisc*):ab,ti	1,859
#26	(surg* NEAR/5 incision*):ab,ti	17,515
#25	(surg* NEAR/5 site*):ab,ti	39,941
#24	(surg* NEAR/5 wound*):ab,ti	22,004
#23	(surg* NEAR/5 infect*):ab,ti	52,141
#22	'wound dehiscence'/exp	19,795
#21	'surgical infection'/exp	53,361
#20	#1 OR #2 OR #3 OR #4 OR #5 OR #6 OR #7 OR #8 OR #9 OR #10 OR #11 OR #12 OR #13 OR #14 OR #15 OR #16 OR #17 OR #18 OR #19	116,591
#19	vape*:ab,ti OR vaporizer*:ab,ti OR vaporiser*:ab,ti OR vapourizer*:ab,ti OR vapouriser*:ab,ti OR vaping*:ab,ti	4,229
#18	'e cig*':ab,ti OR ecig*:ab,ti	6,596
#17	(electron* NEAR/3 (cig* OR nicotine*)):ab,ti	4,720
#16	'electronic cigarette*':ab,ti	4,023
#15	ecigarette*:ab,ti OR 'e cigarette*':ab,ti	6,234
#14	'electronic cigarette'/exp	7,737
#13	'vaping'/exp	2,989
#12	(pharmaco* NEAR/2 (nicotine OR tobacco OR smoking)):ab,ti	1,381
#11	((nicotine OR tobacco) NEAR/2 (gum* OR lozenge* OR patch* OR spray*)):ab,ti	2,997
#10	'nicotine replacement':ab,ti	4,564
#9	nrt:ab,ti	2,890
#8	'nicotine chewing gum'/exp	2,956
#7	'smoking cessation agents'/exp	36
#6	'nicotinic agonists'/exp	25,417
#5	'tobacco use cessation products'/exp	2,956

#4	'tobacco use cessation'/exp	61,602
#3	((quit* OR stop* OR ceas* OR giv* OR abstain* OR abstinen* OR cessat* OR reduc*) NEAR/3 (sm ok* OR tobacco)):ab,ti	62,187
#2	'smoking reduction'/exp	278
#1	'smoking cessation'/exp	61,602

S2.4.1c. Cochrane library database (inception of database to April 24th, 2021)

ID	Search	Hits
#1	MeSH descriptor: [Smoking Cessation] explode all trees	4,145
#2	MeSH descriptor: [Smoking Reduction] explode all trees	25
#3	((quit* or stop* or ceas* or giv* or abstain* or abstinen* or Cessat* or reduc*) NEAR/3 (smoke* or tobacco)):ti,ab,kw	5,174
#4	MeSH descriptor: [Tobacco Use Cessation Devices] explode all trees	550
#5	MeSH descriptor: [Nicotine Chewing Gum] explode all trees	14
#6	MeSH descriptor: [Nicotinic Agonists] explode all trees	741
#7	(Tobacco Use Cessation Products):ti,ab,kw	287
#8	(Nicotine near/2 patch*):ti,ab,kw	1,299
#9	(Nicotine near/2 delivery device*):ti,ab,kw	103
#10	(nicotine near/2 nasal spray*):ti,ab,kw	97
#11	(smoking near/2 cessation product*):ti,ab,kw	639
#12	(nicotine near/2 replacement):ti,ab,kw	1,837
#13	(nicotine near/2 lozenge*):ti,ab,kw	177
#14	(nicotine polacrilex):ti,ab,kw	50
#15	(nicotine near/2 inhal*):ti,ab,kw	150
#16	(nicotine near/2 chewing gum*):ti,ab,kw	140
#17	nicorette:ti,ab,kw	97
#18	(nicotinic near/2 agonist*):ti,ab,kw	831
#19	(nicotine near/2 tablet*):ti,ab,kw	41
#20	(nicotine near/2 sublingual):ti,ab,kw	25
#21	NRT:ti,ab,kw	926
#22	(nicotine near/3 therap*):ti,ab,kw	2,045
#23	MeSH descriptor: [Vaping] explode all trees	52
#24	MeSH descriptor: [Electronic Nicotine Delivery Systems] explode all trees	148
#25	(Ecigarette* or e-cigarette*):ti,ab,kw	542
#26	(Electronic-Cigarette*):ti,ab,kw	441
#27	((electron* NEAR/3 (cig* OR nicotine*))):ti,ab,kw	543
#28	(e-cig* or ecig*):ti,ab,kw	587
#29	(vape* or vaporizer* or vaporiser* or vapourizer* or vapouriser* or vaping*):ti,ab,kw	2,341
#30	#1 or #2 or #3 or #4 or #5 or #6 or #7 or #8 or #9 or #10 or #11 or #12 or #13 or #14 or #15 o r #16 or #17 or #18 or #19 or #20 or #21 or #22 or #23 or #24 or #25 or #26 or #27 or #28 or # 29	11,862
#31	MeSH descriptor: [Surgical Wound Infection] explode all trees	3,454
#32	MeSH descriptor: [Surgical Wound Dehiscence] explode all trees	470
#33	(surg* near/5 infect*):ti and (surg* near/5 infect*):ab (Word variations have been searched)	961
#34	(surg* near/5 wound*):ti and (surg* near/5 wound*):ab (Word variations have been searched)	445
#35	(surg* near/5 site*):ti and (surg* near/5 site*):ab (Word variations have been searched)	784

#36	(surg* near/5 incision*):ti and (surg* near/5 incision*):ab (Word variations have been searched)	347
#37	(surg* near/5 dehisc*):ti and (surg* near/5 dehisc*):ab (Word variations have been searched)	8
#38	(wound* near/5 dehisc*):ti and (wound* near/5 dehisc*):ab (Word variations have been searched)	32
#39	(wound* near/5 infect*):ti and (wound* near/5 infect*):ab (Word variations have been searched)	460
#40	(wound near/5 disrupt*):ti and (wound near/5 disrupt*):ab (Word variations have been searched)	9
#41	wound complication*:ti and wound complication*:ab (Word variations have been searched)	567
#42	#31 or #32 or #33 or #34 or #35 or #36 or #37 or #38 or #39 or #40 or #41	5,559
#43	#30 and #42 in Trials	7

S2.4.1d. CINAHL database (inception of database to April 24th, 2021)

#	Query	Results
S28	S15 and S27	140
S27	S16 OR S17 OR S18 OR S19 OR S20 OR S21 OR S22 OR S23 OR S24 OR S25 OR S26	88,226
S26	TX wound complication*	3,240
S25	TX (wound n5 disrupt*)	875
S24	TX (wound* n5 infect*)	34,820
S23	TX (wound* n5 dehisc*)	4,292
S22	TX (surg* n5 dehisc*)	2,573
S21	TX (surg* n5 incision*)	9,626
S20	TX (surg* n5 site*)	24,201
S19	TX (surg* n5 wound*)	32,261
S18	TX (surg* n5 infect*)	37,386
S17	(MH “Surgical Wound Dehiscence”)	1,575
S16	(MH “Surgical Wound Infection”)	11,253
S15	S1 or S2 or S3 or S4 or S5 or S6 or S7 or S8 or S9 or S10 or S11 or S12 or S13 or S14	31,640
S14	TI (vape$ or vaporizer* or vaporiser* or vapourizer* or vapouriser* or vaping*) OR AB (vape$ or vaporizer* or vaporiser* or vapourizer* or vapouriser* or vaping*)	1,625
S13	TI (e-cig* or ecig*) OR AB (e-cig* or ecig*)	3,758
S12	TI (electron* W3 (cig* or nicotine*)) OR AB (electron* W3 (cig* or nicotine*))	2,539
S11	TI Electronic-Cigarette* OR AB Electronic-Cigarette*	2,180
S10	TI (Ecigarette* OR e-cigarette*) OR AB (Ecigarette* OR e-cigarette*)	3,616
S9	(MM "Electronic Cigarettes")	1,919
S8	(MM "Vaping")	197
S7	TI (nicotine and (gum* or lozenge* or spray* or patch*)) OR AB (nicotine and (gum* or lozenge* or spray* or patch*))	1,150
S6	TI nicotine replacement OR AB nicotine replacement	1,836
S5	TI NRT OR AB NRT	947
S4	(MH “Nicotine Chewing Gum” OR (MH “Nicotine Patch”)	454
S3	(MH (“Nicotine Replacement Therapy”) OR (MH “Tobacco Use Cessation Products+”) OR (MH “Nicotinic Agonists+”)	10,955
S2	TI ((quit* or stop* or ceas* or giv* or abstain* or abstinen* or Cessat* or reduc*) W3 (smok* or tobacco)) OR AB ((quit* or stop* or ceas* or giv* or abstain* or abstinen* or Cessat* or reduc*) W3 (smok* or tobacco))	13,457
S1	(MM "Smoking Cessation")	14,099

S2.4.1e. AMED database (inception of database to April 27th, 2021)

#	Searches	Results
1	Smoking Cessation.mp. [mp=abstract, heading words, title]	343
2	Smoking Reduction.mp. [mp=abstract, heading words, title]	3
3	((quit* or stop* or ceas* or giv* or abstain* or abstinen* or Cessat* or reduc*) and (smok* or tobacco)).mp. [mp=abstract, heading words, title]	650
4	"Tobacco Use Cessation"/	0
5	"Tobacco Use Cessation Products"/	0
6	Nicotinic Agonists/	0
7	Smoking Cessation Agents/	0
8	Nicotine Chewing Gum/	0
9	NRT.ti,ab.	8
10	nicotine replacement.tw.	18
11	((nicotine or tobacco) adj2 (gum* or lozenge* or patch* or spray*)).tw.	11
12	(pharmaco* adj2 (nicotine or tobacco or smoking)).tw.	10
13	(ecigarette* or e cigarette*).ab,ti.	7
14	electronic cigarette*.ab,ti.	2
15	(electron* adj3 (cig* or nicotine*)).ab,ti.	5
16	(e cig* or ecig*).ab,ti.	7
17	(vape* or vaporizer* or vaporiser* or vapourizer* or vapouriser* or vaping*).ab,ti.	6
18	1 or 2 or 3 or 4 or 5 or 6 or 7 or 8 or 9 or 10 or 11 or 12 or 13 or 14 or 15 or 16 or 17	665
19	(surg* adj5 infect*).tw.	223
20	(surg* adj5 wound*).tw.	192
21	(surg* adj5 site*).tw.	120
22	(surg* adj5 incision*).tw.	82
23	(surg* adj5 dehisc*).tw.	16
24	(wound* adj5 dehisc*).tw.	71
25	(wound* adj5 infect*).tw.	440
26	(wound adj5 disrupt*).tw.	1
27	wound complication*.tw.	147
28	19 or 20 or 21 or 22 or 23 or 24 or 25 or 26 or 27	941
29	18 and 28	18

S2.4.2. The second search strategy (the first updated search)

S2.4.2a. MEDLINE database (year of 2021 to August 6th, 2021)

#	Searches	Results
1	exp Smoking Cessation/	30,268
2	exp Smoking Reduction/	90
3	((quit$ or stop$ or ceas$ or giv$ or abstain$ or abstinen$ or Cessat$ or reduc$) adj3 (smok$ or tobacco)).ti,ab.	48,336
4	"Tobacco Use Cessation"/	1,285
5	"Tobacco Use Cessation Products"/	2,065
6	Nicotinic Agonists/	7,515
7	Smoking Cessation Agents/	227
8	Nicotine Chewing Gum/	27
9	NRT.ti,ab.	2,237

10	nicotine replacement.tw,kf.	3,688
11	((nicotine or tobacco) adj2 (gum* or lozenge* or patch* or spray*)).tw,kf.	2,503
12	(pharmaco* adj2 (nicotine or tobacco or smoking)).tw,kf.	1,154
13	exp Vaping/	2,109
14	exp Electronic Nicotine Delivery Systems/	5,020
15	(Ecigarette$ or e-cigarette$).ab,ti.	5,442
16	Electronic-Cigarette$.ab,ti.	3,476
17	(electron* adj3 (cig$ or nicotine$)).ab,ti.	4,139
18	(e-cig$ or ecig$).ti,ab.	5,722
19	(vape$ or vaporizer$ or vaporiser$ or vapourizer$ or vapouriser$ or vaping$).ti,ab.	3,598
20	1 or 2 or 3 or 4 or 5 or 6 or 7 or 8 or 9 or 10 or 11 or 12 or 13 or 14 or 15 or 16 or 17 or 18 or 19	71,553
21	exp Surgical Wound Infection/	37,954
22	exp Surgical Wound Dehiscence/	7,583
23	(surg* adj5 infect*).tw.	37,514
24	(surg* adj5 wound*).tw.	16,856
25	(surg* adj5 site*).tw.	27,890
26	(surg* adj5 incision*).tw.	12,237
27	(surg* adj5 dehisc*).tw.	1,326
28	(wound* adj5 dehisc*).tw.	5,681
29	(wound* adj5 infect*).tw.	37,212
30	(wound adj5 disrupt*).tw.	672
31	wound complication*.tw.	6,327
32	21 or 22 or 23 or 24 or 25 or 26 or 27 or 28 or 29 or 30 or 31	134,341
33	20 and 32	227
34	limit 33 to yr="2021 -Current"	7

S2.4.2b. EMBASE database (year of 2021 to August 7th, 2021)

No.	Query	Results
#34	#20 AND #32 AND [2021-2021]/py	20
#33	#20 AND #32	465
#32	#21 OR #22 OR #23 OR #24 OR #25 OR #26 OR #27 OR #28 OR #29 OR #30 OR #31	193,258
#31	'wound complication*':ab,ti	8,870
#30	(wound NEAR/5 disrupt*):ab,ti	1,026
#29	(wound* NEAR/5 infect*):ab,ti	51,713
#28	(wound* NEAR/5 dehisc*):ab,ti	8,024
#27	(surg* NEAR/5 dehisc*):ab,ti	1,957
#26	(surg* NEAR/5 incision*):ab,ti	17,867
#25	(surg* NEAR/5 site*):ab,ti	40,953
#24	(surg* NEAR/5 wound*):ab,ti	22,594
#23	(surg* NEAR/5 infect*):ab,ti	53,362
#22	'wound dehiscence'/exp	20,289
#21	'surgical infection'/exp	54,736

#20	#1 OR #2 OR #3 OR #4 OR #5 OR #6 OR #7 OR #8 OR #9 OR #10 OR #11 OR #12 OR #13 OR #14 OR #15 OR #16 OR #17 OR #18 OR #19	118,655
#19	vape*:ab,ti OR vaporizer*:ab,ti OR vaporiser*:ab,ti OR vapourizer*:ab,ti OR vapouriser*:ab,ti OR vaping*:ab,ti	4,525
#18	'e cig*':ab,ti OR ecig*:ab,ti	6,969
#17	(electron* NEAR/3 (cig* OR nicotine*)):ab,ti	4,943
#16	'electronic cigarette*':ab,ti	4,199
#15	ecigarette*:ab,ti OR 'e cigarette*':ab,ti	6,590
#14	'electronic cigarette'/exp	8,116
#13	'vaping'/exp	3,322
#12	(pharmaco* NEAR/2 (nicotine OR tobacco OR smoking)):ab,ti	1,391
#11	((nicotine OR tobacco) NEAR/2 (gum* OR lozenge* OR patch* OR spray*)):ab,ti	3,027
#10	'nicotine replacement':ab,ti	4,629
#9	nrt:ab,ti	2,940
#8	'nicotine chewing gum'/exp	2,966
#7	'smoking cessation agents'/exp	40
#6	'nicotinic agonists'/exp	25,924
#5	'tobacco use cessation products'/exp	2,966
#4	'tobacco use cessation'/exp	62,328
#3	((quit* OR stop* OR ceas* OR giv* OR abstain* OR abstinen* OR cessat* OR reduc*) NEAR/3 (smok* OR tobacco)):ab,ti	63,027
#2	'smoking reduction'/exp	297
#1	'smoking cessation'/exp	62,328

S2.4.2c. Cochrane library database (year of 2021 to August 7th, 2021)

ID	Search	Hits
#1	MeSH descriptor: [Smoking Cessation] explode all trees	4,217
#2	MeSH descriptor: [Smoking Reduction] explode all trees	27
#3	((quit* or stop* or ceas* or giv* or abstain* or abstinen* or Cessat* or reduc*) NEAR/3 (smoke* or tobacco)):ti,ab,kw	5,304
#4	MeSH descriptor: [Tobacco Use Cessation Devices] explode all trees	571
#5	MeSH descriptor: [Nicotine Chewing Gum] explode all trees	16
#6	MeSH descriptor: [Nicotinic Agonists] explode all trees	748
#7	(Tobacco Use Cessation Products):ti,ab,kw	296
#8	(Nicotine near/2 patch*):ti,ab,kw	1,316
#9	(Nicotine near/2 delivery device*):ti,ab,kw	106
#10	(nicotine near/2 nasal spray*):ti,ab,kw	97
#11	(smoking near/2 cessation product*):ti,ab,kw	659
#12	(nicotine near/2 replacement):ti,ab,kw	1,871
#13	(nicotine near/2 lozenge*):ti,ab,kw	181
#14	(nicotine polacrilex):ti,ab,kw	50
#15	(nicotine near/2 inhal*):ti,ab,kw	151
#16	(nicotine near/2 chewing gum*):ti,ab,kw	142
#17	nicorette:ti,ab,kw	97
#18	(nicotinic near/2 agonist*):ti,ab,kw	840
#19	(nicotine near/2 tablet*):ti,ab,kw	41

#20	(nicotine near/2 sublingual):ti,ab,kw	25
#21	NRT:ti,ab,kw	954
#22	(nicotine near/3 therap*):ti,ab,kw	2,079
#23	MeSH descriptor: [Vaping] explode all trees	60
#24	MeSH descriptor: [Electronic Nicotine Delivery Systems] explode all trees	162
#25	(Ecigarette* or e-cigarette*):ti,ab,kw	584
#26	(Electronic-Cigarette*):ti,ab,kw	463
#27	((electron* NEAR/3 (cig* OR nicotine*))):ti,ab,kw	571
#28	(e-cig* or ecig*):ti,ab,kw	628
#29	(vape* or vaporizer* or vaporiser* or vapourizer* or vapouriser* or vaping*):ti,ab,kw	2,394
#30	#1 or #2 or #3 or #4 or #5 or #6 or #7 or #8 or #9 or #10 or #11 or #12 or #13 or #14 or #15 o r #16 or #17 or #18 or #19 or #20 or #21 or #22 or #23 or #24 or #25 or #26 or #27 or #28 or # 29	12,110
#31	MeSH descriptor: [Surgical Wound Infection] explode all trees	3,505
#32	MeSH descriptor: [Surgical Wound Dehiscence] explode all trees	472
#33	(surg* near/5 infect*):ti and (surg* near/5 infect*):ab (Word variations have been searched)	989
#34	(surg* near/5 wound*):ti and (surg* near/5 wound*):ab (Word variations have been searched)	458
#35	(surg* near/5 site*):ti and (surg* near/5 site*):ab (Word variations have been searched)	814
#36	(surg* near/5 incision*):ti and (surg* near/5 incision*):ab (Word variations have been searched)	359
#37	(surg* near/5 dehisc*):ti and (surg* near/5 dehisc*):ab (Word variations have been searched)	8
#38	(wound* near/5 dehisc*):ti and (wound* near/5 dehisc*):ab (Word variations have been searched)	32
#39	(wound* near/5 infect*):ti and (wound* near/5 infect*):ab (Word variations have been searched)	473
#40	(wound near/5 disrupt*):ti and (wound near/5 disrupt*):ab (Word variations have been searched)	9
#41	wound complication*:ti and wound complication*:ab (Word variations have been searched)	579
#42	#31 or #32 or #33 or #34 or #35 or #36 or #37 or #38 or #39 or #40 or #41	5,671
#43	#30 and #42 with Publication Year from 2021 to 2021, in Trials	1

S2.4.2d. CINAHL database (year of 2021 to August 7th, 2021)

#	Query	Results
S28	S15 and S27 (Published Date: 20210401-20210831)	1
S27	S16 OR S17 OR S18 OR S19 OR S20 OR S21 OR S22 OR S23 OR S24 OR S25 OR S26	89,123
S26	TX wound complication*	3,353
S25	TX (wound n5 disrupt*)	875
S24	TX (wound* n5 infect*)	35,124
S23	TX (wound* n5 dehisc*)	4,352
S22	TX (surg* n5 dehisc*)	2,633
S21	TX (surg* n5 incision*)	9,689
S20	TX (surg* n5 site*)	24,564
S19	TX (surg* n5 wound*)	32,722
S18	TX (surg* n5 infect*)	37,975
S17	(MH "Surgical Wound Dehiscence")	1,615
S16	(MH "Surgical Wound Infection")	11,535
S15	S1 or S2 or S3 or S4 or S5 or S6 or S7 or S8 or S9 or S10 or S11 or S12 or S13 or S14	32,207
S14	TI (vape$ or vaporizer* or vaporiser* or vapourizer* or vapouriser* or vaping*) OR AB (vape$ or vaporizer* or vaporiser* or vapourizer* or vapouriser* or vaping*)	1,718
S13	TI (e-cig* or ecig*) OR AB (e-cig* or ecig*)	3,922

S12	TI (electron* W3 (cig* or nicotine*)) OR AB (electron* W3 (cig* or nicotine*))	2,625
S11	TI Electronic-Cigarette* OR AB Electronic-Cigarette*	2,243
S10	TI (Ecigarette* OR e-cigarette*) OR AB (Ecigarette* OR e-cigarette*)	3,779
S9	(MM "Electronic Cigarettes")	1,992
S8	(MM "Vaping")	228
S7	TI (nicotine and (gum* or lozenge* or spray* or patch*)) OR AB (nicotine and (gum* or lozenge* or spray* or patch*))	1,160
S6	TI nicotine replacement OR AB nicotine replacement	1,872
S5	TI NRT OR AB NRT	970
S4	(MH "Nicotine Chewing Gum" OR (MH "Nicotine Patch")	456
S3	(MH ("Nicotine Replacement Therapy") OR (MH "Tobacco Use Cessation Products+") OR (MH "Nicotinic Agonists+")	11,179
S2	TI ((quit* or stop* or ceas* or giv* or abstain* or abstinen* or Cessat* or reduc*) W3 (smok* or tobacco)) OR AB ((quit* or stop* or ceas* or giv* or abstain* or abstinen* or Cessat* or reduc*) W3 (smok* or tobacco))	13,671
S1	(MM "Smoking Cessation")	14,364

S2.4.2e. AMED database (year of 2021 to August 9th, 2021)

#	Searches	Results
1	Smoking Cessation.mp. [mp=abstract, heading words, title]	344
2	Smoking Reduction.mp. [mp=abstract, heading words, title]	3
3	((quit* or stop* or ceas* or giv* or abstain* or abstinen* or Cessat* or reduc*) and (smok* or tobacco)).mp. [mp=abstract, heading words, title]	652
4	"Tobacco Use Cessation"/	0
5	"Tobacco Use Cessation Products"/	0
6	Nicotinic Agonists/	0
7	Smoking Cessation Agents/	0
8	Nicotine Chewing Gum/	0
9	NRT.ti,ab.	8
10	nicotine replacement.tw.	18
11	((nicotine or tobacco) adj2 (gum* or lozenge* or patch* or spray*)).tw.	11
12	(pharmaco* adj2 (nicotine or tobacco or smoking)).tw.	10
13	(ecigarette* or e cigarette*).ab,ti.	7
14	electronic cigarette*.ab,ti.	2
15	(electron* adj3 (cig* or nicotine*)).ab,ti.	5
16	(e cig* or ecig*).ab,ti.	7
17	(vape* or vaporizer* or vaporiser* or vapourizer* or vapouriser* or vaping*).ab,ti.	6
18	1 or 2 or 3 or 4 or 5 or 6 or 7 or 8 or 9 or 10 or 11 or 12 or 13 or 14 or 15 or 16 or 17	667
19	(surg* adj5 infect*).tw.	227
20	(surg* adj5 wound*).tw.	197
21	(surg* adj5 site*).tw.	125
22	(surg* adj5 incision*).tw.	85
23	(surg* adj5 dehisc*).tw.	16
24	(wound* adj5 dehisc*).tw.	72
25	(wound* adj5 infect*).tw.	452
26	(wound adj5 disrupt*).tw.	1

27	wound complication*.tw.	154
28	19 or 20 or 21 or 22 or 23 or 24 or 25 or 26 or 27	969
29	18 and 28	18
30	limit 29 to yr="2021-Current"	0

Appendix S3. PRISMA flow chart of the impact of preoperative smoking cessation time, nicotine replacement therapy (NRT), and vaping

Flow chart of the impact of preoperative smoking cessation time, NRT, and vaping on bone healing is shown in **Appendix S3a.** Flow chart of the impacts of preoperative smoking cessation time, NRT, and vaping on wound healing and wound complications is shown in **Appendix S3b.**

Appendix S3a. PRISMA flow chart of the impacts of preoperative smoking cessation time, NRT, and vaping on bone healing

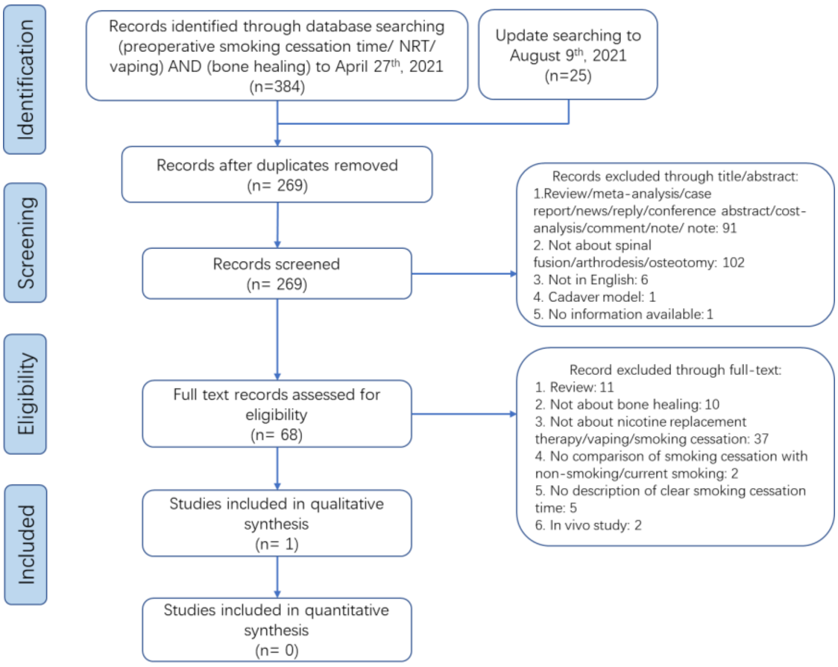

Appendix S3b. PRISMA flow chart of the impacts of preoperative smoking cessation time, NRT, and vaping on wound healing and wound complications after surgery

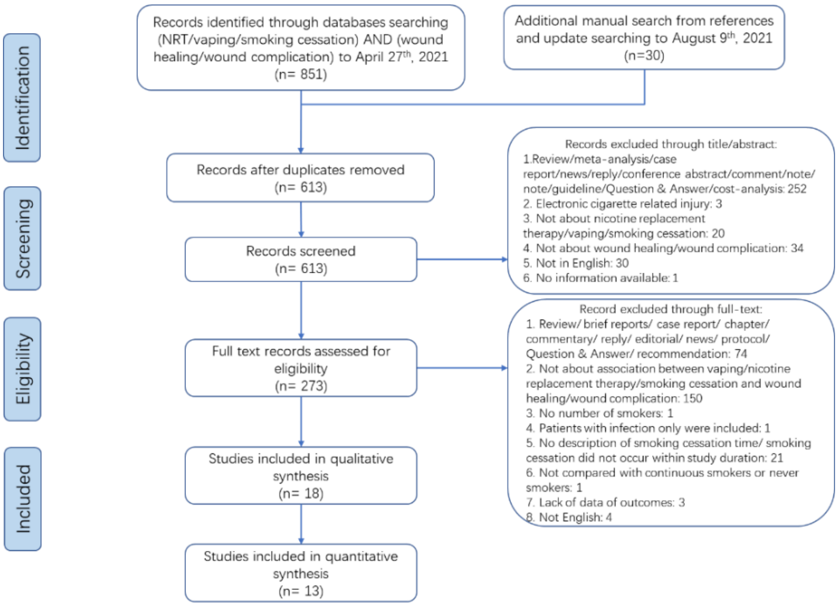

Appendix S4. Search results

Search results about smoking and alcohol consumption were shown in **Appendix S4a**. Search results about preoperative smoking cessation time, nicotine replacement therapy (NRT), and vaping were shown in **Appendix S4b.**

Appendix S4a. Smoking and alcohol consumption

Through the initial literature searching to the date of October 28th, 2019, we acquired 4,120 studies from MEDLINE, 10,159 studies from EMBASE, 880 studies from Cochrane CENTRAL, 1,272 studies from CINAHL, 82 studies from AMED, and four studies from an additional manual search from the references. Updated literature searching was subsequently performed to the date of July 10th, 2020, a date of October 14th, 2020, and then to a date of August 9th, 2021. Additionally, 4,832 articles were obtained. After removing duplicated studies via Endnote X9 software, 13,035 studies remained. By screening through titles and abstracts, 12,072 articles were removed for several reasons, including due to unsuitable types of work (5,025 studies), cost analysis (19 studies), and a lack of comparisons of smoking or alcohol on bone healing of adult human traumatic bone fractures (6,179 studies), etc. After the eligibility process was conducted, another 860 full-text studies were excluded because seven papers were not in English, 47 papers had inapplicable types of work, and 265 papers had unrelated topics, etc. Finally, 103 studies published from 1977 to 2021 were included in the current study.

Forty-five of the 103 studies were not included in the meta-analysis for the following reasons: (1) no outcome events occurred in either group; (2) no information on groups was observed in which outcome events had occurred; (3) a lack of data was observed on outcomes in each group; (4) only mean values were found without standard deviation (SD) values; (5) the control group may have included alcohol drinkers or former smokers; (6) definitions of delayed union, nonunion, or normal union with incorrect time periods or a lack of descriptions of time periods were observed; and (7) regarding time to union, data of patients with nonunion were excluded or were regarded as being the end of the follow-up period. Only two studies mentioned malunion. However, no events occurred in the exposure group or control group. Therefore, a meta-analysis of malunion could not be conducted because of insufficient data.

Appendix S4b. Preoperative smoking cessation time, NRT, and vaping

For bone healing (**Appendix S3a**), wound healing and wound complications (**Appendix S3b**), two additional literature searches were performed to the date of April 27th, 2021. An updated search was performed to the date of August 9th, 2021.

For bone healing, we acquired 120 studies from MEDLINE, 229 studies from EMBASE, 13 studies from Cochrane CENTRAL, 36 studies from CINAHL, and 11 studies from AMED databases. After removing the duplicated studies, 269 studies were screened through titles and abstracts. Among them, 201 studies were removed via screening, and another 67 studies were removed via the eligibility process. Finally, one study concerning bone healing was included in the current study. Due to the fact that no more than one study was included, the quantitative analysis of bone healing could not be performed.

For wound healing and wound complications, we acquired 234 studies from MEDLINE, 479 studies from EMBASE, eight studies from Cochrane CENTRAL, 141 studies from CINAHL, 18 studies from AMED databases, and one study from an additional manual search of the references. After removing the duplicated studies, 613 studies were screened through titles and abstracts. Three hundred and forty studies were excluded via the screening process, and 255 studies were excluded via the full-text. Finally, 18 studies concerning wound healing and wound complications were included in this study. Five of the studies were not included in the quantitative analysis because there was no more than one study on each outcome.

Appendix S5. Summary of 58 studies for the quantitative synthesis about the impacts of smoking and alcohol consumption

Summary of 58 studies for the quantitative synthesis about the impacts of smoking and alcohol consumtion was described in **Appendix S5a to d**.

S5a. Summary of 58 studies for the quantitative synthesis (part 1)

No	First author/ Publication year	Journal name	Country	Study design	Sex M/F	Age (years) mean±SD (range)	Race	BMI (kg/m²) mean±SD (range)	Height (cm) mean±SD (range)	Weight (kg) mean±SD (range)
1	Askew 2011[1]	The Open Bone Journal	USA	retrospective cohort study	29/1	GS 67·33±14·04 (28–88)<br>ConS 68·22±19·14 (38–89)<br>GA 64·25±11·92 (45–80)<br>ConA 69·83±17·30 (28–89)<br>Total 67·6±15·40 (28–89)	NA	NA	NA	NA
2	Bai 2019[2]	Medicine	China	case-control study	372/293	49·4±15·7 (18–77)	NA	NA	NA	NA
3	Ding 2014[3]	Journal of Orthopaedic Science	China	case-control study	431/228	Mean 45·22	NA	NA	NA	NA
4	Kent 2015[4]	European Journal of Orthopaedic Surgery & Traumatology	England	retrospective cohort study	18/22	GS 47·75±11·74 (28–65)<br>ConS 54·43±19·74 (27–87)<br>GA 50±13·71 (28–65)<br>ConA 52·85±18·61 (27–87)<br>Total 52·43±17·84 (27–87)	NA	NA	NA	NA
5	Liu 2015[5]	Orthopaedics & Traumatology: Surgery & Research	China	case-control study	391/413	Mean 54·6	NA	Mean 19·64	Mean 172·71	Mean 73·09
6	Ma 2017[6]	International Wound Journal	China	case-control study	465/211	Mean 44·6	NA	Mean 25·26	NA	NA
7	Mckissack 2019[7]	Injury	USA	retrospective cohort study	NA	NA	NA	NA	NA	NA
8	Meng 2018[8]	International Wound Journal	China	case-control study	1310/1295	Mean 44·4 (18–82)	NA	Mean 24·52	NA	NA
9	Ovaska 2013[9]	The Journal of bone and joint surgery· American volume	Finland	case-control study	NA	NA	NA	NA	NA	NA

10	Parker 2018[10]	The Bone & Joint Journal	UK	case-control study	340/118	NA	White (164)<br>Black Caribbean (13)<br>Black African (149)<br>Black Other (79)<br>Indian (23)<br>Pakistani (24)<br>Unknown (6)	NA	NA	NA
11	Thorud 2017[11]	The Journal of Foot & Ankle Surgery	US	case-control study	56/88	51·1±15·0	White (106)<br>Black (14)<br>Other (24)	31·4±7·1	NA	NA
12	Bowen 2005[12]	Clinical Orthopaedics and Related Research	US	case-control study	96/50	47±18 (18–92)	NA	NA	NA	NA
13	Chan 2019[13]	Injury	England	case-control study	76/99	46·95±18·2	NA	26·1±4·8	NA	NA
14	Claessen 2016[14]	Clinical Orthopaedics and Related Research	USA	case-control study	572/748	Mean 54	NA	NA	NA	NA
15	Flynn 2000[15]	Foot & Ankle International	Puerto Rico	retrospective cohort study	66/32	Mean 48·08 (18–85)	NA	NA	NA	NA
16	Sun 2017[16]	International Journal of Surgery	China	case-control study	673/574	44·1±14·4 (18–81)	NA	Mean 24·64	NA	NA
17	Alzahrani 2018[17]	Journal of Orthopaedics and Traumatology	Canada	retrospective cohort study	74/28	34·9±12·8	NA	NA	NA	NA
18	Basques 2015[18]	Journal of Orthopaedic Trauma	US	case-control study	222/297	52·3±15·8	NA	29·2±7·9	NA	NA
19	Blair 2016[19]	Journal of Orthopaedic Trauma	US	retrospective cohort study	156/28	Mean 42·21	NA	NA	NA	NA
20	Bone 1997[20]	The Journal of Bone and Joint Surgery	USA	retrospective cohort study	28/19	GS 35·5±11·87 (21–57)<br>ConS 37·66±17·87 (18–72)<br>Total 36·83±15·73 (18–72)	NA	NA	NA	NA
21	Branch 2015[21]	The American Journal of Surgery	USA	retrospective cohort study	6900/2506	Mean 29	White (5708)<br>black (2326)<br>others (1372)	NA	NA	NA
22	Chu 2018[22]	Tzu Chi Medical Journal	Taiwan	retrospective cohort study	82/38	Mean 47·95	NA	NA	NA	NA
23	Donohue 2016[23]	Journal of Orthopaedic Trauma	US	retrospective cohort study	186/142	Mean 42·19 (18–97)	NA	NA	NA	NA
24	Enninghorst 2011[24]	the Journal of Trauma	Australia	case-control study	66/23	41±7	NA	NA	NA	NA
25	Haller 2019[25]	Journal of Orthopaedic Trauma	US	case-control study	370/148	Mean 43·45 (18–81)	NA	NA	NA	NA

26	Kim 2018[26]	International Orthopaedics	South Korea	case-control study	14/59	Mean 69·75 (43–87)	NA	Mean 25·54	NA	NA
27	Kuklo 2008[27]	The Journal of bone and joint surgery· British volume	USA	retrospective cohort study	129/0	Mean 26·6 (20–42)	NA	NA	NA	NA
28	McDonald 2018[28]	Foot & Ankle International	USA	case-control study	129/152	Median 51 (39–63)	NA	Median 28·8 (26·3–33·3)	NA	NA
29	Moghaddam 2011[29]	Injury	Germany	prospective cohort study	61/24	GS 46±12 (22–79)<br>ConS 41±18 (18–84)<br>Total 44±15 (18–84)	NA	NA	NA	NA
30	Momaya 2016[30]	Injury	US	case-control study	341/191	47·76±15·2 (20–89)	Black (178)<br>Other (11)<br>White (343)	NA	NA	NA
31	Morris 2013[31]	Journal of Orthopaedic Trauma	US	case-control study	193/109	45·7±14·3 (19–76)	White (257)<br>non-white (45)	NA	NA	NA
32	Murray 2013[32]	The Journal of bone and joint surgery· American volume	UK	case-control study	678/263	Mean 36·8 (18–94)	NA	NA	NA	NA
33	Ozbek 2017[33]	World Neurosurgery	Turkey	prospective cohort study	53/21	Mean 38·98	NA	Mean 26·87	NA	NA
34	Shah 2014[34]	Journal of Orthopaedic Trauma	US	retrospective cohort study	130/52	Mean 44·74 (18–77)	NA	NA	NA	NA
35	Singh 2018[35]	Indian Journal of Orthopaedics	Singapore	case-control study	111/8	Mean 38·2 (18–70)	Chinese (68)<br>Malay (18)<br>Indians (27)<br>Others (6)	NA	NA	NA
36	Snall 2015[36]	Medicina oral, patologia oral y cirugia bucal	Finland	prospective randomised study	36/1	Mean 28 (18·1–50·9)	NA	NA	NA	NA
37	Zumsteg 2014[37]	Journal of Hand Surgery-American Volume	US	case-control study	NA	NA	NA	NA	NA	NA
38	Assous 2001[38]	Injury	UK	case series	NA	Mean 35 (18–52)	NA	NA	NA	NA
39	Hoffmann 2013[39]	Journal of Orthopaedic Surgery and Research	USA	retrospective cohort study	49/57	Mean 54 (18–95)	NA	Mean 29·8 (17–67)	NA	NA
40	Li 2017[40]	International Orthopaedics	China	case-control study	247/123	Mean 46·19 (19–79)	NA	NA	NA	NA
41	Lin 2013[41]	European Journal of Orthopaedic Surgery & Traumatology	USA	case-control study	170/81	Mean 42·98 (18–78)	NA	NA	NA	NA
42	McGarvey 2006[42]	Foot & Ankle International	US	case series	24/7	Mean 41·5 (19–64)	NA	NA	NA	NA
43	Minhas 2019[43]	Journal of Hand Surgery (American	US	retrospective cohort study	2531/975	NA	Caucasian ethnicity (2976)	NA	NA	NA

		Volume)					non-Caucasian (530)			
44	Korim MT 2014[44]	The Bone & Joint Journal	UK	case-control study	NA	Mean 51 (18–100)	NA	NA	NA	NA
45	Ren 2015[45]	Clinics	China	case-control study	NA	NA	NA	NA	NA	NA
46	Spross 2012[46]	Clinical Orthopaedics and Related Research	Switzerland	retrospective cohort study	4/18	Mean 75 (42–93)	NA	NA	NA	NA
47	Benedick 2020[47]	Foot & Ankle International	US	retrospective cohort study	446/457	45·3±16·3	White (570) Black (267) Hispanic (58) Other (5)	31·3±8·0	NA	NA
48	Esposito 2019[48]	Journal of Orthopaedic Trauma	US	case-control study	384/197	median 45 (IQR 35–55)	NA	Median 27 (IQR 24–31)	NA	NA
49	Ogihara 2019[49]	World Neurosurgery	Japan	case-control study	331/292	Mean 63·0 (18–97)	NA	Mean 22·59	NA	NA
50	Li 2020[50]	Orthopaedic Surgery	China	retrospective cohort study	117/98	48·5±3·6	NA	NA	NA	NA
51	Ahmed 2018[51]	Journal of Orthopaedic Trauma	Egypt	case series	12/6	42·17±6·82 (33–55)	NA	NA	NA	NA
52	Ukai 2020[52]	J Orthop Traumatol	Japan	case-control study	81/29	Mean 44·5 (18–84)	NA	Mean 23·22	NA	NA
53	Olson 2020a[53]	Injury	US	retrospective cohort study	262/139	46±14 (18–87)	White (329) other/nonidentified (24) Hispanic/Latino (20) Black (20) Asian (8)	27±6 (24–30)	NA	NA
54	Subramanyam 2021[54]	Injury	India	case-control study	81/19	38·06±13·98	NA	NA	NA	NA
55	Mardanpour 2020[55]	International Journal of Surgery Open	Iran, US	case-control study	512/276	Mean 47·5 (20–70)	NA	NA	NA	NA
56	Tan 2021[56]	International Orthopaedics	China	case-control study	483/337	51·3±14·4 (18–92)	NA	NA	NA	NA
57	Zhu 2021[57]	BMC Musculoskeletal Disorders	China	case-control study	193/171	53·7±17·0	NA	25·6±4·1	NA	NA
58	Olson 2020b[58]	JSES International	US	case-control study	95/69	Mean 44·9	NA	Mean 30·5	NA	NA

Note: M: male; F: female; SD: standard deviation; BMI: bone mineral density; GS: smoking group; ConS: control group in smoking comparison; GA: alcoh ol drinking group; ConA: control group in alcohol drinking comparison; NA: not available; IQR: Interquartile range.

S5b. Summary of 58 studies for the quantitative synthesis (part 2)

No	First author/ Publication year	Inclusion and exclusion criteria	type of fracture (traumatic/non-pathological/non-metastatic fracture, etc)	Non-pathological fracture or unclear	Fracture location	Diabetes	Use of NSAIDs	Use of fluoro-quinolone family of antibiotics
1	Askew 2011	**Inclusion criteria:**<br>Patients between the ages of 28 and 89 were treated operatively at the Omaha Veterans Affairs Medical Center between 1994 and 2000 for fractures of the femur and tibia shaft.<br>**Exclusion criteria:**<br>1. patients who lost to follow up,<br>2. patients who died prior to fracture healing<br>3. pathological fractures	Non-pathological fracture	Non-pathological fracture	Femur, Tibia, Fibula	NA	NA	NA
2	Bai 2019	**Inclusion criteria:**<br>(From March 2014 to February 2018) All patients aged 18 or older with acute distal femur fractures (AO/OTA 33) treated by ORIF with screws/plates or only screws were included.<br>**Exclusion criteria:**<br>1. age <18 years<br>2. pathologic fractures<br>3. metastatic fracture<br>4. patients who had implant removal only<br>5. patients who underwent surgical treatment of distal femur fracture at an outside hospital or who presented with surgical site infection after being treated at another hospital<br>6. those with incomplete data.	Non-pathological fracture, non-metastatic fracture	Non-pathological fracture	Femur	Total (65)	NA	NA
3	Ding 2014	All patients who were treated for a humeral diaphyseal fracture at our institution from January 2005 through January 2011 were retrospectively identified by use of our electronic database.<br>**Inclusion criteria were:**<br>1. patients who underwent surgical fixation of a humeral diaphyseal fracture during the study period;<br>2. age 18 years or greater;<br>3. at least 9 months of clinical and radiographic follow-up.<br>**Exclusion criteria were:**<br>1. patients who presented with wound and/or bone infection;<br>2. fractures treated non-operatively;<br>3. pathological fracture due to metastatic bone tumor.	Non-pathological fracture due to metastatic bone tumor	Non-pathological fracture	Humerus	Total (50)	NA	NA
4	Kent 2015	**Inclusion criteria:**<br>All adult patients with a distal third tibial fracture treated with a locking plate between 01/01/2008 and 31/12/2011 at our institution with outpatient follow-up to the point of fracture union and discharge	Non-pathological fracture	Non-pathological fracture	Tibia	GA (0)/ConA (1)<br>GS (0)/ConS (1)	NA	NA

		were included.<br>These patients were identified from the theatre register and trauma database. Data was also collected from case notes and the PACS system and the Trauma and Orthopaedic Medical Information Officer collated the monetary and resource allocation data from the hospital finance department<br>**Exclusion criteria:**<br>1. patients<18 years of age<br>2. patients with pathological fractures				Total (1)		
5	Liu 2015	**Inclusion criteria:**<br>1. patients≥18 years of age<br>2. a fracture in the middle three-fifths of clavicle<br>3. no fracture in other parts of body<br>4. nonoperative treatment (brace or sling) until either confirmed fracture-healing or the development of nonunion<br>5. adequate documentation of demographic details and clinical and radiographic follow-up until fracture-healing or the development of nonunion<br>**Exclusion criteria:**<br>1. no demographic data could be gained during the follow-up study<br>2. incomplete clinical or demographic data<br>3. lost to follow-up before fracture union was determined<br>4. patients who underwent operative treatment after nonoperative treatment (within two weeks of injury), including (1) skin or neurovascular compromise, (2) pathological fracture, floating shoulder, or other multifocal shoulder girdle injury, (3) a request by the patient, (4) a decision of the treating surgeon.<br>5. patients who underwent early operative treatment from two to 24 weeks after injury before the development of definite nonunion.	Non-pathological fracture	Non-pathological fracture	Clavicle	NA	NA	NA
6	Ma 2017	**Inclusion criteria:**<br>Adult patients (18 years or older) with acute close tibial plateau fractures treated with ORIF at the 6th Hospital of Xinjiang Medical University from January 2012 to February 2017 were included in this study.<br>**The exclusion criteria:**<br>1. patients<18 years<br>2. pathological fractures<br>3. periprosthetic fractures<br>4. old fractures (>21 days from initial injury)	Non-pathological fracture, non-periprosthetic fracture, and non-old fracture	Non-pathological fracture	Tibia	Total (20)	NA	NA
7	Mckissack 2019	**Inclusion criteria:**<br>A retrospective chart review of patients age 18 years and older who underwent ORIF for a medial malleolar, lateral malleolar, posterior malleolar, bimalleolar, or trimalleolar ankle fracture at a single institution between 2008 and 2018 was conducted. Patients were selected using Current Procedural Terminology (CPT) codes 27766,	NA	Unclear	Ankle	NA	NA	NA

		27769, 27784, 27792, 27814, 27822, and 27829.<br>(Note: CPT code mentioned above is a medical procedural code under the range - Fracture and/or Dislocation Procedures on the Leg (Tibia and Fibula) and Ankle Joint.)<br>**Exclusion criteria:**<br>1. patients with polytraumatic injuries<br>2. open fractures<br>3. pilon fractures						
8	Meng 2018	**Inclusion criteria:**<br>From the electronic registry system and patients' electronic medical records, all adult patients (18 years and older) who had undergone ORIF of an ankle fracture from January<br>2013 to December 2016 were identified.<br>**Exclusion criteria:**<br>1. patients <18 years<br>2. pathological fractures<br>3. metastatic fracture<br>4. old fractures (>21 days from initial injury)<br>5. treatments other than ORIF (external fixation, conservative treatments, closed reduction and internal fixation, traction).	Non-pathological fracture, non-metastatic fracture, and non-old fracture	Non-pathological fracture	Ankle	Total (155)	NA	NA
9	Ovaska 2013	**Inclusion criteria:**<br>All patients who had undergone surgery to treat an ankle fracture at our institution from January 2006 through December 2009 were identified by querying the hospital surgical procedure database for diagnoses coded with International Classification of Diseases, Tenth Revision (ICD-10), for fibular fracture (S82.4), medial malleoiar fracture (S82.5), lateral malleoiar fracture (S82.6), and bimalieolar or trimalleolar fracture (S82.8) and for procedure codes for internal or external fixation of ankle fractures. Eligible surgical procedures were restrirted to those performed primarily at our institution in patients eighteen years of age or older. We identified 1923 ankle fracture operations in 1915 patients who were all definitively treated with open reduction and internal fixation (ORIF).<br>**Exclusion criteria:** NA	NA	Unclear	Ankle	Total (29)	NA	NA
10	Parker 2018	**Inclusion criteria:**<br>The trial recruited adult patients with open fractures of the lower limb graded as Gustilo–Anderson11 two or above, all of whom were treated in a United Kingdom Major Trauma Centre or Trauma Unit with joint orthopaedic and plastic surgical care.<br>**Exclusion criteria:** NA	NA	Unclear	lower limb	Total (27)	NA	NA
11	Thorud 2017	**Inclusion criteria:**<br>The present study was a retrospective review of patients aged 18 to 90 years, selected using the International Classification of Diseases, 9th Revision, Clinical Modification (ICD-9-CM), diagnosis codes for fractures of the ankle and metatarsals, presenting from January 1, 2006	Non-pathological fracture	Non-pathological fracture	Ankle, Metatarsals	Total (36)	NA	NA

		to January 1, 2012 to Baylor Scott & White Health Memorial Hospital. We included both patients who had undergone operative and nonoperative treatment of fracture.<br>**Exclusion criteria:**<br>1. patients who died before 18 months after the fracture diagnosis<br>2. pathologic fractures						
12	Bowen 2005	**Inclusion criteria:**<br>The medical records of all patients presenting to a Level I trauma center with an acute, traumatic, open fracture of at least one long bone between June 1, 2000 and June 1, 2003 were reviewed retrospectively. Institutional review board approval was obtained before initiation of the study.<br>**Exclusion criteria:**<br>1. patients<18 years<br>2. patients with preexisting infection<br>3. patients who had penetrating trauma<br>4. patients receiving antibiotic treatment before the injury for a condition not related to the open fracture<br>5. patients with open fractures of the hands, feet, spine, and pelvis without long bone<br>involvement<br>6. patients with pathologic fractures.	Non-pathological fracture	Non-pathological fracture	long bones (leg, thigh, forearm, upper arm)	Total (13)	NA	NA
13	Chan 2019	A retrospective analysis of 210 consecutive patients surgically treated for tibial plateau fractures at two Level 1 trauma centres (Major Trauma Centres) between January 2015 and April 2017 was performed.<br>**Inclusion criteria:**<br>1. patients over 18 years of age<br>2. operative treatment with open reduction internal fixation<br>3. a minimum of 6-months follow-up<br>**Exclusion criteria:**<br>1. patients under 18 years of age<br>2. those treated with circular frames, intramedullary fixation or percutaneous screw<br>fixation	NA	Unclear	Tibia	Total (14)	NA	NA
14	Claessen 2016	**Inclusion criteria:**<br>We retrospectively reviewed 1380 adult patients who underwent surgery for an elbow fracture between January 2002 and July 2014 at four area hospitals. Patients were identified using Current Procedural Terminology (CPT) codes for elbow fractures (Appendix 1. Supplemental materials are available with the online version of CORR1.).<br>**Exclusion criteria:**<br>1. patients who had prior surgery for an elbow fracture elsewhere<br>2. patients who had a fracture through a malignant tumor	Non-metastatic fracture	Non-pathological fracture	Elbow	Total (143)	NA	NA

		3. patients who had a nonunion<br>4. patients who had more than one skin incision<br>5. patients who died owing to respiratory failure after the surgery.						
15	Flynn 2000	**Inclusion criteria:**<br>We studied all patients with closed ankle fractures that were treated from January 1, 1988 to June 31, 1997. Criteria for inclusion in the study was age greater than 18 years, isolated closed lateral malleolar, medial malleolar, bimalleolar, or trimalleolar fracture and adequate follow-up during the period in which the study was performed.<br>**Exclusion criteria:**<br>1. patients with Charcot neuroarthropathy<br>2. patients who died<br>3. patients who were lost to follow-up during initial treatment<br>4. patients who were unable to be contacted for further follow-up.	NA	Unclear	Ankle	Total (25)	NA	NA
16	Sun 2017	**Inclusion criteria:**<br>All adult patients ≥18 years) undergoing ORIF of a closed ankle fracture from July 2015 through January 2017 were identified by querying patients' electronic medical records (EMR).<br>**Exclusion criteria:**<br>1. patients <18 years<br>2. open fractures<br>3. pathological fractures<br>4. old fractures (>21 days from injury)<br>5. other treatments for reduction and fixation of ankle fracture such as external fixation, conservative treatments, manual reduction and internal fixation and traction et al.	Non-pathological fracture and non-old fracture	Non-pathological fracture	Ankle	Total (84)	NA	NA
17	Alzahrani 2018	Inclusion criteria:<br>patients age greater than 18 years with a minimum of 12 months of clinical follow-up after the index surgery. Surgical indications included acute, mid-shaft clavicle fractures with significant shortening (> 2 cm) or displacement (> 100% of clavicle width), open fractures, impending skin compromise, associated neurological or vascular injury and polytrauma patients.<br>Exclusion criteria:<br>1. patients who required operative treatment for a symptomatic nonunion or malunion<br>after failed non-operative treatment<br>2. pathological fractures<br>3. patients with insufficient radiographs that precluded classification of the fracture pattern.	Non-pathological fracture	Non-pathological fracture	Clavicle	NA	NA	NA
18	Basques 2015	**Inclusion criteria:**<br>Patients who underwent ORIF for tibial plateau fracture from 2009 to 2012 were identified in the ACS-NSQIP database. Tibial plateau fracture patients were identified using International Classification of	NA	Unclear	Tibia	Total (55)	NA	NA

		Diseases, ninth Revision, diagnosis codes 823.00, 823.02, 823.10, and 823.12. Only those who received ORIF for tibial plateau fracture [Current Procedural Terminology codes 27535 (unicondylar tibial plateau fracture) and 27536 (bicondylar tibial plateau fracture)] were included for analysis.<br>**Exclusion criteria:**<br>1. patients with incomplete perioperative data<br>2. Current Procedural Terminology codes that indicated a surgical procedure other than tibial plateau ORIF.						
19	Blair 2016	**Inclusion criteria:**<br>Skeletally mature patients must have had an acute tibial plateau fracture operatively treated with a plate and screw construct or tibial shaft fracture operatively treated with an intramedullary rod in the setting of acute compartment syndrome (ACS) requiring fasciotomy. The diagnosis of ACS<br>was made by clinical judgment of the attending orthopaedic surgeon.<br>**Exclusion criteria:**<br>1. patients who had an associated vascular injury (ie, Gustilo-Anderson IIIC open fracture)<br>2. patients<18 years of age<br>3. patients who had incomplete medical records to determine the time at which union or complication occurred<br>4. patients who had a tibial plateau fracture definitively treated with an implant other than a plate and screw construct or a tibial shaft fracture treated with an implant other than an intramedullary rod (eg, circular external fixation).	NA	Unclear	Tibia	NA	NA	NA
20	Bone 1997	**Inclusion criteria:**<br>The criteria for inclusion in the study were an age of at least sixteen years, a closed displaced fracture of the tibial diaphysis (from five centimeters distal to the tibial tuberosity to seven centimeters proximal to the ankle joint), and an absence of associated compartment syndrome or neurovascular injury.<br>**Exclusion criteria:** NA	NA	Unclear	Tibia	NA	NA	NA

21	Branch 2015	**Inclusion criteria:**<br>Patients were identified using International Classification of Diseases, Ninth Revision, Clinical Modification (ICD9-CM) codes for open femoral fractures<br>2. patients ≥418 years old, and underwent ORIF<br>(ICD-9 code: 79.35) of the femur at a level I or level II trauma center.<br>**Exclusion criteria:**<br>1. patients who were dead on arrival<br>2. patients who died in the emergency department<br>3. patients whose race/ethnicity was not documented<br>4. level III trauma center patients<br>5. where the trauma center classification was unknown.	NA	Unclear	Femur	Total (605)	NA	NA
22	Chu 2018	**Inclusion criteria:**<br>1. having a displaced middle-third clavicle fracture (Robinson type 2B1/2B2)<br>2. age between 30 and 65 years<br>3. having a complete record of radiographic and functional evaluations at regular follow-up periods of at least 6 months.<br>**Exclusion criteria:**<br>1. bilateral clavicle fracture<br>2. fracture associated with other fractures or major injuries<br>3. another trauma at the time of follow-up<br>4. initial injury combined with injured skin integrity or skin tenting of the fracture fragment.	NA	Unclear	Clavicle	Total (14)	NA	NA
23	Donohue 2016	**Inclusion criteria:**<br>patients with femoral (OTA 32) or tibial (OTA 42)16 shaft fractures who were treated with reamed, statically locked intramedullary rod fixation were identified, and their electronic medical records were screened to identify patients who were skeletally mature. Both open and closed fractures were included.<br>**Exclusion criteria:**<br>1. 1-year follow-up data were not available<br>2. an NSAID other than ketorolac was administered.	NA	Unclear	Femur，Tibia	Total (46)	received ketorolac in the postanesthesia care unit or on the ward within the first 24 hours after the surgical procedure<br>GS (32) ConS (53)<br>Total (85)	NA

24	Enninghorst 2011	**Inclusion criteria:**<br>A 36-month prospective cohort study was performed between January 1, 2007, and December 31, 2009, at the John Hunter Hospital (University of Newcastle affiliated Level 1 trauma center) in New South Wales, Australia. Consecutive adult (age 18 years) blunt trauma patients with open tibia shaft fractures (Orthopedic Trauma Association code 42A, B, and C) were included in this observational study.<br>**Exclusion criteria:** NA	NA	Unclear	Tibia	NA	NA	NA
25	Haller 2019	**Inclusion criteria:**<br>Patients greater than 18 years of age with a minimum of 12-month follow-up. Medical records were reviewed for demographic data including age, sex, tobacco use, and mechanism of injury.	NA	Unclear	Tibia	Total (31)	NA	NA
26	Kim 2018	**Inclusion criteria:**<br>From January 2009 to December 2015, 86 patients (86 fractures) who had been operatively treated for distal femur fractures (AO/OTA type 33) caused by **low-energy trauma** were retrospectively recruited from five secondary or tertiary trauma centres (Seoul Medical Center, Ajou University Hospital, Hanyang University Hospital, Kangnam Sacred Heart Hospital, and Hallym Sacred Heart Hospital) of South Korea. A low-energy trauma was defined as a fall from a standing height or less.<br>**Exclusion criteria:**<br>1. patients who had associated injuries occurring at the affected leg or pelvis or sustained a periprosthetic fracture around hip prostheses<br>2. patients due to a previous experience of fracture or infection of the effected femur<br>(no pathologic fractures from metastatic malignancies were found)<br>3. patients who experienced a peri-plate fracture after a ground-level fall prior<br>to bone healing within six weeks following surgery<br>4. a new trauma is responsible for the need for further surgery<br>5. patients who were lost to follow-up before bony union<br>6. patients with insufficient data.	Non-prosthetic fracture, Non-pathological fracture	Non-pathological fracture	Femur	Total (22)	NA	NA
27	Kuklo 2008	**Inclusion criteria:**<br>Between March 2004 and March 2005, 138 Gustilo-Anderson type IIIB and IIIC segmental tibial fractures in 138 soldiers returning from Iraq were treated at the Walter Reed Army Medical Centre.<br>**Exclusion criteria:**<br>1. patients with a head injury<br>2. patients who were lost to follow-up	NA	Unclear	Tibia	NA	NA	NA
28	McDonald 2018	**Inclusion criteria:**<br>Patients with an isolated, closed ankle fracture (AO fracture classification 44-A and 44-B) definitively managed with ORIF were	Non-pathological fracture	Non-pathological fracture	Ankle	Total (27)	NA	NA

		included.<br>**Exclusion criteria:**<br>1. patients with pilon fractures, open injuries, polytrauma with other lower extremity fractures<br>2. pathological fractures<br>3. allergy to ketorolac<br>4. renal insufficiency						
29	Moghaddam 2011	**Inclusion criteria:**<br>all consenting patients over 18 years of age exhibiting radiological evidence of a tibial shaft fracture were considered for inclusion in this study.<br>**Exclusion criteria:**<br>1. any fracture line that extended proximally to the knee joint or distally to the ankle joint<br>2. multiple traumas<br>3. significant soft-tissue damage<br>4. injuries other than a lower leg fracture<br>5. type III open fracture according to the Gustilo classification	NA	Unclear	Tibia	Total (1)	NA	NA
30	Momaya 2016	**Inclusion criteria:**<br>1. articular fracture (AO/OTA B/C)<br>2. age >19 years.<br>**Exclusion criteria:**<br>1. patients who were: pregnant<br>2. patients who had insufficient documentation for review<br>3. patients who transferred following any surgery other than damage control orthopaedic procedures.	NA	Unclear	Tibia	Total (69)	NA	NA
31	Morris 2013	**Inclusion criteria:**<br>A total of 302 patients aged 18 years and older were identified as undergoing operative fixation during this time period and were included in the study.<br>**Exclusion criteria:** NA	NA	Unclear	Tibia	Total (22)	NA	NA
32	Murray 2013	**Inclusion criteria:**<br>1. a fracture in the middle three-fifths of the clavicle<br>2. complete displacement of the main fragments with no cortical contact<br>3. initial nonoperative treatment until either confirmed fracture-healing or the development of<br>nonunion, using criteria stipulated below<br>4. adequate documentation of demographic details and clinical and radiographie follow-up until fracture healing or the development of nonunion.<br>**Exclusion criteria:**<br>1. documentation of demographic data was missing and we had been unable to gain further information during the follow-up study<br>2. incomplete documentation of clinical and/or radiographic outcome	Non-pathological fracture	Non-pathological fracture	Clavicle	NA	NA	NA

33		3. patients who were lost to follow-up before union status was determined<br>4. patients who underwent primary operative treatment (within two weeks of injury), which was performed as a result of skin or neurovascular compromise<br>5. pathological fracture<br>6. floating shoulder<br>7. other multifocal shoulder girdle injury<br>8. a request by the patient<br>9. a decision of the treating surgeon<br>10. patients who underwent early operative treatment from two to twenty-four weeks after injury (at their request or as a result of a decision of the surgeon) before the development of definite nonunion.						
33	Ozbek 2017	**Inclusion criteria:**<br>1. Fractures after acute trauma<br>2. Type A3 and A4 fractures (according to AO classification)<br>**Exclusion criteria:**<br>1. Osteoporosis (T-score [BMD] <-2.5 SD)<br>2. Pathologic fracture; History of malignancy<br>3. Neurological damage<br>4. Decompression with laminectomy<br>5. Multiple level fractures<br>6. Other trauma in addition to spinal trauma<br>7. Younger than 20 years and older than 60 years old<br>8. Nonintact bilateral pedicle<br>9. Comorbidity (diabetes, chronic kidney failure, steroid use)	Non-osteoporotic fracture, Non-pathological fracture, Non-metastatic fracture	Non-pathological fracture	T11, T12, L1, and L2 vertebrae	Total (0)	NA	NA
34	Shah 2014	**Inclusion criteria:**<br>1. aged >18 years<br>2. OTA classification 41C and 43C fractures treated with 2-staged provisional external fixation followed by delayed open reduction internal fixation<br>3. patients with follow-up available through radiographic healing.<br>**Exclusion criteria:**<br>1. patients whose injury was associated with compartment syndrome requiring fasciotomy<br>2. patients who had follow-up of less than 3 months.	NA	Unclear	Tibia	Total (22)	NA	NA
35	Singh 2018	**Inclusion criteria:**<br>adults (age ≥18 years) with open diaphyseal tibial fractures who presented during the study. Patients were identified through operative records as well as in-house electronic discharge coding.<br>**Exclusion criteria:**<br>Patients with delayed presentations (i.e., referrals from other local and overseas centers)	NA	Unclear	Tibia	NA	NA	NA
36	Snall 2015	**Inclusion criteria:**<br>patients aged 18 years or more who had participated in a randomised	NA	Unclear	Mandible	NA	NA	NA

		study aimed at clarifying the benefits of dexamethasone on pain, oedema and nausea after open reduction and fixation of a facial fracture.<br>**Exclusion criteria:**<br>1. patients with infected fractures<br>2. a history of liver or kidney dysfunction<br>3. a history of peptic ulcer<br>4. a history of psychosis due to steroid use<br>5. pregnancy<br>6. breastfeeding or allergy to any constituent of the dexamethasone preparation used.						
37	Zumsteg 2014	**Inclusion criteria:**<br>1. patients 18 years of age and older<br>2. presence of an open fracture of the radius and/or ulna<br>3. availability of accurate information regarding time of injury and clinical care within the medical record.<br>**Exclusion criteria:**<br>1. patients with ballistic injuries or traumatic amputations<br>2. patients with inadequate information in the medical record	NA	Unclear	Radius, Ulna	Total (16)	NA	NA
38	Assous 2001	**Inclusion criteria:**<br>Clinical notes of the last 40 patients who had internal fixation of the Os clacis were reviewed. The age of patients ranged from 18 to 52 years (mean 35 years).<br>**Exclusion criteria:** NA	NA	Unclear	Calcaneus	Total (2)	NA	NA
39	Hoffmann 2013	**Inclusion criteria:**<br>All patients with supracondylar femoral fracture treated with locked plate fixation and age equal to or older than 18 years were included in this study.<br>**Exclusion criteria:**<br>1. patients with intramedullary fixation<br>2. patients with metastatic disease<br>3. impaired lower extremity motor or nerve function prior to injury<br>4. supplemental methods for bone healing	Non-metastatic fracture	Non-pathological fracture	Femur	Total (21)	NA	NA
40	Li 2017	**Inclusion criteria:**<br>Our investigation retrospectively identified patients 18 years or older with acute tibial plateau fractures treated by ORIF at our trauma and emergency center from January 2015 to June 2016, with post-operative follow-up ranged from two to 12 months. Patients with complete follow-up data could be included in this study.<br>**Exclusion criteria:**<br>1. patients younger than 18 years<br>2. fractures around the prosthesis<br>3. pathological fractures<br>4. old fractures (>21 days from initial injury).	Non-periprosthetic fracture, non-pathological fracture	Non-pathological fracture	Tibia	Total (22)	NA	NA

41	Lin 2013	**Inclusion criteria:**<br>we retrospectively identified patients who had sustained an intra-articular fracture of the proximal tibia as well as received ORIF for treatment by querying the Hospital Medical Informatics database for admissions coded with International Classification of Diseases, Ninth Revision, Clinical Modification (ICD-9-CM) procedure codes. Eligible operations were restricted to those performed by an orthopedic surgeon in patients 18 years of age or older. Three hundred and twenty-seven consecutive patients with tibial plateau fractures (332 cases) who underwent ORIF between January 2004 and December 2010 were identified.<br>**Exclusion criteria:**<br>1. patients who had <6 months follow-up<br>2. patients who died of polytrauma after definitive fixation<br>3. patients who had insufficient medical record data.	NA	Unclear	Tibia	Total (19)	NA	NA
42	McGarvey 2006	**No inclusion or exclusion criteria**<br>A subset of patients was collected prospectively between October of 1998 and January of 2003. All fractures of the calcaneus were treated at our institution by one surgeon (WCM). Thirty-three fractures in 31 patients were treated with indirect reduction and external fixation. The average age of the 24 men and seven women at the time of injury was 41.5 (19 to 64) years	NA	Unclear	Calcaneus	Total (1)	NA	NA
43	Minhas 2019	**Inclusion criteria:**<br>Patients undergoing operative fixation were identified through Current Procedural Terminology (CPT) codes. These included open and percutaneous stabilization of metacarpal fractures or metacarpal fracture-dislocations (CPT 26615, 26650 26665, 26676, 26685, and 26686) and open and percutaneous stabilization of proximal and middle phalanx fractures or fracture-dislocations (CPT 26706, 26715, 26717, 26727, 26735, 26746, 26776, and 26785).<br>**Exclusion criteria:**<br>1. Distal phalanx fractures<br>2. Polytrauma patients	NA	Unclear	Metacarpal, phalanx	Total (192)	NA	NA
44	Korim MT 2014	**Inclusion criteria:**<br>Patients who underwent ORIF of a fracture of the ankle between April 2010 and February 2013 were identified from the trauma database.<br>**Exclusion criteria:**<br>1. patients aged < 18 years old<br>2. patients with a fracture of the tibial plafond.	NA	Unclear	Ankle	Total (10)	NA	NA
45	Ren 2015	**Inclusion criteria:**<br>1. patient age of 18 years or greater<br>2. patients who underwent surgical fixation of a pilon fracture during the study period<br>3. at least 12 months of clinical and radiographic follow-up.<br>**Exclusion criteria:**	Non-metastatic fracture	Non-pathological fracture	Tibia	NA	NA	NA

		1. pregnancy<br>2. lactation<br>3. sepsis<br>4. human immuno-deficiency virus-1 (HIV) infection<br>5. any site infection within the past thirty days<br>6. patients who underwent initial fracture surgery at an outside hospital or presented with SSIs after being treated at another hospital<br>7. metastatic fractures<br>8. death within 30 days of initial surgery<br>9. severe concomitant disease of other systems.						
46	Spross 2012	**Inclusion criteria:**<br>a traumatic Neer Group VI fracture treated either by the PHILOS1 plate or primary HA and a minimum of 12 months follow-up.<br>**Exclusion criteria:** NA	traumatic fracture	Non-pathological fracture	Humerus	NA	NA	NA
47	Benedick 2020	**Inclusion criteria:**<br>1. age 18 years or older at the time of injury<br>2. patients who sustained an Orthopaedic Trauma Association (OTA) 44B or 44C9 torsional ankle fracture<br>3. patients who underwent fixation of their ankle fracture<br>4. patients who had at least 6 weeks of clinical follow-up from the date of surgery.<br>**Exclusion criteria:**<br>Patients with missing data regarding intraoperative lower extremity tourniquet use	NA	Unclear	Ankle	Total (125)	NA	NA
48	Esposito 2019	**Inclusion criteria:**<br>1. Patients who underwent operative treatment for Arbeitsgemeinschaft für Osteosynthesefragen/Orthopaedic Trauma Association (AO/OTA) 43-B and 43-C fractures<br>2. patients who were 18 years and older<br>**Exclusion criteria:**<br>1. extra-articular AO/OTA 43-A fractures<br>2. fractures deemed mainly tibial shaft with extension into the tibial plafond<br>3. trimalleolar ankle fractures.	NA	Unclear	Tibia	Total (37)	NA	NA
49	Ogihara 2019	**Inclusion criteria:**<br>In this study, spinal trauma was defined as spinal fracture and/or dislocation.<br>Patients who had undergone conventional posterior open instrumented surgery;<br>**Exclusion criteria:**<br>1. patients with pathologic spinal fracture such as tumoral or infectious spinal abnormalities<br>2. patients who had undergone minimally invasive (MI) surgery with percutaneous pedicle screws	Traumatic fracture, non-pathological fracture (such as tumoral or infectious abnormalities)	Non-pathological fracture	spine	Total (76)	NA	NA

		3. patients <18 years and/or who had undergone single-stage anterior-posterior surgery						
50	Li 2020	**Inclusion criteria:**<br>1. patients with open tibial fracture injury time, first debridement time, and complete prognosis data<br>2. hospital stay longer than 2 weeks<br>3. patients followed for more than 10 weeks.<br>**Exclusion criteria:**<br>1. patients with multiple open fractures<br>2. patients with amputated open fractures<br>3. patients with hyperthyroidism and hematological diseases.	NA	Unclear	Tibia	Total (22)	NA	NA
51	Ahmed 2018	**Inclusion criteria:**<br>1. pilon fractures with late presentation more than 1 month without definitive treatment<br>2. management by the Ilizarov fixator<br>3. a minimum follow-up of 1.5 years<br>**Exclusion criteria:**<br>1. acute fractures<br>2. patients who were lost to follow-up	NA	Unclear	Tibia	GS (2), ConS (1)<br>Total (3)	NA	NA
52	Ukai 2020	**Inclusion criteria:**<br>patients with lower limb open fractures of G–A type III who were followed up for at least 2 years after the surgical procedure.<br>**Exclusion criteria:**<br>1. open fractures of the upper limb and foot<br>2. closed fractures<br>3. open fractures of G–A types I, II, and IIIC	NA	Unclear	Femur, tibia, ankle	Total (5)	NA	NA
53	Olson 2020a	**Inclusion criteria:**<br>1. patients with diagnosis of a closed complete articular (OTA/AO 43C1–3) fracture treated with ORIF.<br>2. patients 18 years of age and older.<br>**Exclusion criteria:**<br>Any simple ankle fractures, extraarticular or partial-articular distal tibia fractures, or open fractures	NA	Unclear	Tibia	Total (26)	NA	NA
54	Subramanyam 2021	**Inclusion criteria:**<br>1. displaced fractures (any fracture that was displaced by 2 mm or more or angulated by 5 °or more on radiographs at pre- sentation was diagnosed as a displaced fracture)<br>2. skeletally mature patients (more than 18 years of age)<br>3. presentation within three days of injury.<br>**Exclusion criteria:**<br>1. undisplaced fractures	Non-pathological fracture	Non-pathological fracture	Clavicle	Total (7)	NA	NA

		2. skeletally immature patients (age less than 18 years)<br>3. delayed presentation to our department, open fractures, pathological fractures, bilateral fractures, and associated other fractures or visceral injuries.						
55	Mardanpour 2020	**Inclusion criteria:**<br>adult patients who underwent the ORIF procedure for acetabulum fracture at three educational hospitals<br>**Exclusion criteria:**<br>1. younger patients <18 years, old patient >70 years<br>2. pathological fractures<br>3. old fractures more than two weeks after injury<br>4. other operation rather than ORIF	Non-pathological fracture	Non-pathological fracture	acetabulum	NA	NA	NA
56	Tan 2021	**Inclusion criteria:**<br>1. age > 18 years,<br>2. diagnosed with closed isolated patella fractures<br>3. patients agreed to participate in the study<br>4. patients underwent surgical treatment.<br>**Exclusion criteria:**<br>1. pathologic fracture<br>2. old fractures (> 21 days)<br>3. periprosthetic fractures<br>4. patients with incomplete data<br>5. patients who received SSI treatment in our hospital but did not undergo initial surgery	Non-periprosthetic fracture, Non-pathological fracture	Non-pathological fracture	patella	Total (64)	NA	NA
57	Zhu 2021	**Inclusion criteria:**<br>1. patients who were 18 years old and over with acute distal femur fractures treated by ORIF<br>**Exclusion criteria:**<br>1. age less than 18 years old<br>2. old fractures (> 21 days from earliest trauma)<br>3. pathological fractures<br>4. first treatment at other hospitals.	Non-pathological fracture	Non-pathological fracture	Femur	Total (86)	NA	NA
58	Olson 2020b	**Inclusion criteria:**<br>1. adult patients with humeral shaft fractures who presented to an urban level I trauma center<br>**Exclusion criteria:**<br>1. periprosthetic or pathological fracture<br>2. patients with less than one-year clinical follow-up	Non-pathological fracture, traumatic fracture, non-periprosthetic fracture	Non-pathological fracture	Humerus	Total (19)	NA	NA

Note: NSAIDs: non-steroidal anti-inflammatory drugs; NA: not available; GS: smoking group; ConS: control group in smoking comparison; GA: alcohol drinkin g group; ConA: control group in alcohol drinking comparison; ORIF: open reduction and internal fixation; BMD: bone mineral density; SD: standard deviation.

S5c. Summary of 58 studies for the quantitative synthesis (part 3)

No	First author /Publication year	Treatment	Operative or nonoperative treatment	Smoking type	Smoking dose	Smoking g roup	Smoking Control g roup
1	Askew 2011	1. Dynamic hip screw; 2. ORIF; 3. ORIF/dyn amic hip screw; 4. gamma long nail; 5. ORIF / dynamic condylar screw; 6. retrograde nail; 7. ORIF/ilizarov external fixation; 8. closed ca st; 9. ORIF/plate/cable; 10. intramedullary nai l; 11. iliac crest bone graft /closed rod	mixture of operative and nonoperative treat ment	NA	NA	Smoking hi story (curre nt and for mer smoke rs) (*n*=21)	Non-smoki ng history (*n*=9)
2	Bai 2019	ORIF with screws/plates or only screws	operative treatment	NA	NA	Smoking (*n*=148)	Non-smoki ng (*n*=517)
3	Ding 2014	1. Plate fixation 2. Intramedullary nailing	operative treatment	NA	NA	Smoking hi story (*n*=165)	Non-smoki ng history (*n*=494)
4	Kent 2015	locking plate	operative treatment	NA	NA	Smokers (*n*=12)	Non-smok ers (*n*=28)
5	Liu 2015	treated nonoperatively	nonoperative treatment	NA	NA	Smokers (*n*=155)	Non-smok ers (*n*=649)
6	Ma 2017	ORIF	operative treatment	NA	NA	Current sm oking (*n*=73)	non-curren t smoking (*n*=603)
7	Mckissack 2 019	ORIF	operative treatment	NA	NA	Tobacco us e (*n*=NA)	Non-tobac co use (*n*=NA)
8	Meng 2018	ORIF	operative treatment	NA	NA	Current sm oking (*n*=554)	Non-curren t smoking (*n*=2,063)
9	Ovaska 2013	ORIF	operative treatment	NA	NA	Tobacco us e (*n*=64)	Non-tobac co use (*n*=198)
10	Parker 2018	NA	NA	NA	NA	Smokers (*n*=147)	Non-smok ers (*n*=300)
11	Thorud 2017	operative and nonoperative treatment	mixture of operative and nonoperative treat ment	NA	not mentioned	Active+quit smokers (*n*=55)	Never smo ked (*n*=85)
12	Bowen 2005	all fracture care, including surgery	mixture of operative and nonoperative treat ment	NA	NA	Tobacco us e (*n*=65)	Non-tobac co use (*n*=97)
13	Chan 2019	ORIF	operative treatment	NA	NA	Smoking (*n*=43)	Non-smoki ng

							(n=101)
14	Claessen 201 6	1. plate and screw fixation<br>2. screw alone<br>3. tension band wire<br>4. radial head arthroplasty<br>5. other fixation type<br>6. external fixation before surgery<br>including radial head arthroplasty, maybe com bined with fixation, so maybe there is no nee d to exclude radial head arthroplasty	operative treatment	NA	NA	Smoking (n=343)	Non-smoki ng (n=977)
15	Flynn 2000	closed reduction and immobilization and ORI F.	mixture of operative and nonoperative treat ment	cigarette	1. no smoking ever<br>2. moderate smoking (<1 pack per day)<br>3. heavy smoking (> 1 pack per day)	Heavy smo kers (n=16)<br>Moderate s moking (n=25)	Non-smok ers (n=57)
16	Sun 2017	ORIF	operative treatment	NA	NA	Current sm oking (n=262)	Non-curren t smoking (n=985)
17	Alzahrani 20 18	ORIF	operative treatment	NA	NA	Smokers (n=19)	Non-smok ers (n=83)
18	Basques 201 5	ORIF	operative treatment	NA	NA	Smoking hi story (n=170)	Non-smoki ng history (n=349)
19	Blair 2016	operatively treated with a plate and screw con struct<br>operatively treated with an intramedullary rod in the setting of acute compartment syndrome requiring fasciotomy	operative treatment	NA	NA	Smokers (n=48)	Non-smok ers (n=136)
20	Bone 1997	closed intramedullary nailing, closed reduction and above-the-knee-cast	mixture of operative and nonoperative treat ment	NA	NA	History of smoking (n=18)	Non-histor y of smok ing (n=29)
21	Branch 2015	ORIF	operative treatment	NA	NA	Smoking hi story (n=827)	Non-smoki ng history (n=8,579)
22	Chu 2018	ORIF, conservative treatment	mixture of operative and nonoperative treat ment	NA	NA	Smoking (n=24)	Non-smoki ng (n=96)
23	Donohue 20 16	reamed, statically locked intramedullary rod fi xation	operative treatment	NA	NA	Smoking (n=109)	Non-smoki ng (n=219)
24	Enninghorst	intramedullary nailing	mixture of operative and nonoperative treat	NA	NA	Smokers	Non-smok

	2011	external fixation<br>closed reduction and application of plaster<br>percutaneous plating	ment			(*n*=23)	ers<br>(*n*=66)
25	Haller 2019	NA	NA	NA	NA	Tobacco us e<br>(*n*=118)	Non-tobac co use<br>(*n*=400)
26	Kim 2018	minimally invasive lateral locked plating	operative treatment	NA	NA	Smoking<br>(*n*=5)	Non-smoki ng<br>(*n*=68)
27	Kuklo 2008	ringed fixator or reamed intramedullary nail	operative treatment	NA	NA	Smokers<br>(*n*=14)	non-smoke rs<br>(*n*=115)
28	McDonald 2 018	ORIF	operative treatment	NA	NA	Current+ f ormer smo kers<br>(*n*=73)	Non-smok ers<br>(*n*=208)
29	Moghaddam 2011	Intramedullary nailing; Osteosynthesis plate; E xternal fixation; Cast	mixture of operative and nonoperative treat ment	cigarette	NA	Current sm okers and previous s mokers<br>(*n*=46)	Non-smok ers<br>(*n*=39)
30	Momaya 201 6	tibial plateau ORIF	operative treatment	NA	NA	Tobacco us e<br>(*n*=240)	Non-tobac co use<br>(*n*=292)
31	Morris 2013	splinting or external fixator	mixture of operative and nonoperative treat ment	NA	NA	Smokers<br>(*n*=137)	Non-smok ers<br>(*n*=165)
32	Murray 2013	nonoperative treatment	nonoperative treatment	NA	NA	Smokers<br>(*n*=219)	Non-smok ers<br>(*n*=722)
33	Ozbek 2017	Fusion	operative treatment	NA	NA	Smokers<br>(*n*=19)	Non-smok ers<br>(*n*=56)
34	Shah 2014	2-staged provisional external fixation followed by delayed open reduction internal fixation	operative treatment	NA	NA	Smokers<br>(*n*=71)	Non-smok ers<br>(*n*=111)
35	Singh 2018	Intramedullary fixation;<br>Extramedullary fixation;<br>External fixation	operative treatment	NA	NA	Smokers<br>(*n*=26)	Non-smok ers<br>(*n*=77)
36	Snall 2015	Open reduction and fixation with the aid of ti tanium miniplates	operative treatment	NA	NA	Smokers<br>(*n*=23)	Non-smok ers<br>(*n*=14)
37	Zumsteg 201 4	internal fixation<br>external fixation	operative treatment	NA	NA	Smokers<br>(*n*=63)	Non-smok ers

								(n=137)
38	Assous 2001	internal fixation	operative treatment	cigarette	from 10 to 40 cigar ettes per day	Smokers (n=13)	Non-smok ers (n=27)
39	Hoffmann 2 013	locked plate fixation	operative treatment	NA	NA	Current+ f ormer smo kers (n=41)	Non-smok ers (n=70)
40	Li 2017	ORIF	operative treatment	NA	NA	Tobacco co nsumption (n=62)	Non-tobac co consum ption (n=308)
41	Lin 2013	ORIF	operative treatment	NA	NA	Smokers (n=105)	Non-smok ers (n=151)
42	McGarvey 2 006	indirect reduction and external fixation	operative treatment	NA	NA	Smokers (n=8)	Non-smok ers (n=23)
43	Minhas 2019	open and percutaneous stabilization	operative treatment	NA	NA	Smokers (n=1,112)	Non-smok ers (n=2,394)
44	Korim MT 2014	ORIF	operative treatment	NA	NA	Smokers (n=18)	Non-smok ers (n=692)
45	Ren 2015	External fixation with limited internal fixation; ORIF	operative treatment	NA	NA	Smoking hi story (n=69)	Non-smoki ng history (n=450)
46	Spross 2012	Angular stable reconstruction using the PHILO S plate	operative treatment	cigarette	more than 10 cigaret tes per day	Smokers (n=7)	Non-smok ers (n=15)
47	Benedick 20 20	fixation	operative treatment	NA	NA	Tobacco us e (n=402)	Non-tobac co use (n=501)
48	Esposito 201 9	ORIF	operative treatment	NA	NA	Smoking (n=154)	Non-smoki ng (n=410)
49	Ogihara 201 9	conventional posterior open instrumented surge ry	operative treatment	NA	NA	Smoking (n=60)	Non-smoki ng (n=563)
50	Li 2020	external fixation limited internal fixation	operative treatment	NA	NA	Tobacco us e (n=84)	Non-tobac co use (n=131)
51	Ahmed 2018	Ilizarov fixator	operative treatment	NA	NA	Smoking (n=8)	Non-smoki ng

							(*n*=10)
52	Ukai 2020	ORIF/external fixation	operative treatment	NA	NA	Smoking (*n*=56)	Non-smoking (*n*=58)
53	Olson 2020a	ORIF	operative treatment	NA	NA	Tobacco use (*n*=99)	Non-tobacco use (*n*=302)
54	Subramanyam 2021	eight clavicular brace and broad arm sling	nonoperative treatment	NA	NA	Smokers (*n*=4)	Non-smokers (*n*=96)
55	Mardanpour 2020	ORIF	operative treatment	NA	NA	Current smoker (*n*=NA)	non-current smoker (*n*=NA)
56	Tan 2021	surgery	operative treatment	NA	NA	Current smoker (*n*=99)	non-current smoker (*n*=721)
57	Zhu 2021	ORIF	operative treatment	NA	NA	Smoking (*n*=50)	Non-smoking (*n*=314)
58	Olson 2020b	ORIF, conservative treatment	mixture of operative and nonoperative treatment	NA	NA	Tobacco use (n=75)	Non-tobacco use (n=89)

Note: ORIF: open reduction and internal fixation; NA: not available.

S5d. Summary of 58 studies for the quantitative synthesis (part 4)

No	First author / Publication year	Alcohol type	Level of alcohol consumption	Alcohol group	Alcohol Control	Follow-up mean±SD, range	At least 1 concurrent fracture at other sites or not
1	Askew 2011	NA	NA	Alcoholics (*n*=12)	Never drinking (*n*=17)	Follow–up was performed NA	No
2	Bai 2019	NA	NA	Alcohol consumption (*n*=263)	Non-alcohol consumption (*n*=402)	NA	No
3	Ding 2014	NA	NA	Drinking (*n*=206)	Non-drinking (*n*=453)	Mean 14·8 (9–24) months	No
4	Kent 2015	NA	NA	Alcohol dependency (*n*=6)	Non-alcohol dependency (*n*=34)	follow–up to the point of fracture union 33·88±15·91 (8–78) weeks	No
5	Liu 2015	NA	NA	Alcohol	Non-alcohol	Follow–up was performed	No

				(*n*=233)	(*n*=571)	NA	
6	Ma 2017	NA	NA	Drinking alcohol (*n*=281)	Non-drinking (*n*=395)	NA	Yes
7	Mckissack 2 019	NA	NA	NA	NA	2 weeks, 6 weeks, 3 months, 6 months and 12 months	No
8	Meng 2018	NA	NA	Drinking alcohol (*n*=658)	Non-drinking (*n*=1,959)	NA	Yes
9	Ovaska 2013	NA	NA	Alcohol abuse (*n*=41)	Non-alcohol abuse (*n*=221)	NA	No
10	Parker 2018	NA	Alcohol intake:<br>0–7 units<br>8–14 units<br>15–21 units<br>>21 units	8–14 units (*n*=79)<br>15–21 units (*n*=45)<br>>21 units (*n*=51)	0–7 units (*n*=269)	1 year	No
11	Thorud 2017	NA	NA	Alcohol history (*n*=37)	Non-alcohol history (*n*=107)	Follow–up was performed NA	No
12	Bowen 2005	NA	NA	Alcohol use (*n*=37)	Non-alcohol use (*n*=125)	primary care provider: 486±3 12 (97–1374) days<br>Orthopaedics: 354±260 (97–1 374) days	Yes
13	Chan 2019	NA	≥14 units per week	Alcohol over users (*n*=30)	Non-alcohol over users (*n*=120)	≥6months	No
14	Claessen 201 6	NA	NA	Alcohol abuse (*n*=69)	Non-alcohol abuse (*n*=1,251)	at least 4 months	No
15	Flynn 2000	NA	1. no alcohol consumption ever<br>2. moderate consumption (<2 dri nks per day)<br>3. heavy consumption (>2 drinks per day)	Heavy alcohol consumption (*n*=20)<br>Moderate drinking (*n*=33)	Non-alcohol consumptio n (*n*=45)	3·5±2·4 years (10 months to 10 years)	No
16	Sun 2017	NA	NA	Alcohol drinking (*n*=251)	Non-alcohol drinking (*n*=996)	NA	No
17	Alzahrani 20 18	-	-	-	-	minimum of 12 months	No
18	Basques 201 5	-	-	-	-	NA	No
19	Blair 2016	-	-	-	-	Follow–up was performed NA	No
20	Bone 1997	-	-	-	-	GS 4·36±1·49 (2–6·5) years<br>ConS 4·33±1·48 (2–6·5) year s<br>Total 4·34±1·47 (2–6·5) year s	No
21	Branch 2015	-	-	-	-	NA	No
22	Chu 2018	-	-	-	-	at least 6months	No

23	Donohue 20 16	-	-	-	-	1 year	Yes
24	Enninghorst 2011	-	-	-	-	12 months	No
25	Haller 2019	-	-	-	-	Mean 27 (12–115) months	No
26	Kim 2018	-	-	-	-	17·3±7·5 (6–44) months	No
27	Kuklo 2008	-	-	-	-	Mean 15·6 (12–32) months	No
28	McDonald 2 018	-	-	-	-	minimum 4 months	No
29	Moghaddam 2011	-	-	-	-	GS 38 (6–60) months ConS 35 (7–60) months Total 39 (6–60)	No
30	Momaya 201 6	-	-	-	-	19·5 months·	No
31	Morris 2013	-	-	-	-	14·1 months	No
32	Murray 2013	-	-	-	-	Follow–up was continued unt il clinical and radiographic e vidence of union	No
33	Ozbek 2017	-	-	-	-	33·77 months	Yes
34	Shah 2014	-	-	-	-	12·5 months	No
35	Singh 2018	-	-	-	-	minimum follow up period w as 6 months (maximum 72 months)	No
36	Snall 2015	-	-	-	-	6 (3–9) months	No
37	Zumsteg 201 4	-	-	-	-	at least 6–month	No
38	Assous 2001	-	-	-	-	27 (12–42) months	No
39	Hoffmann 20 13	-	-	-	-	23·3 (6–72) months	Yes
40	Li 2017	-	-	-	-	(2–12) months	No
41	Lin 2013	-	-	-	-	18 months	Yes
42	McGarvey 2 006	-	-	-	-	25 (6–55) months	Yes
43	Minhas 2019	-	-	-	-	Follow–up was performed NA	No
44	Korim MT 2 014	-	-	-	-	minimum of 6 months	No
45	Ren 2015	-	-	-	-	19·1 (12–29) months	No
46	Spross 2012	-	-	-	-	a minimum of 12 months	No
47	Benedick 20 20	NA	NA	-	-	47·6 weeks (6 weeks to 10 years)	No
48	Esposito 201 9	-	-	-	-	NA	Yes
49	Ogihara 201	-	-	-	-	minimum of 12 months	No

	9						
50	Li 2020	NA	NA	Alcohol drinking (*n*=52)	Non-drinking (*n*=163)	(97–1374) days	No
51	Ahmed 2018	-	-	-	-	GS 29·88±9·80 (18–45) mont hs<br>ConS 44·5±44·84 (18–168) months<br>Total 38±34·06 (18–168) mo nths	No
52	Ukai 2020	-	-	-	-	Mean 37 (24–119) months	Yes
53	Olson 2020a	-	-	-	-	median 1·7 (1·1–3·7) years	No
54	Subramanya m 2021	-	-	-	-	1 year	No
55	Mardanpour 2020	-	-	alcohol intake (*n*=NA)	non-alcohol intake (*n*=NA)	8·2±4·7 (6–13) months	No
56	Tan 2021	-	-	alcohol consumption (*n*=70)	non-alcohol consumptio n (*n*=750)	NA	No
57	Zhu 2021	-	-	alcohol consumption (*n*=58)	non-alcohol consumptio n (*n*=306)	1 year	Yes
58	Olson 2020b	-	-	Alcohol abuse (n=74)	Non-alcohol abuse (n=90)	minimum of 1 year	No

Note: SD: standard deviation; NA: not available.

Two 17-year-old male non-smokers and a 16-year-old male non-smoker were included in Bone 1997 (study No.20). However, the information of each patient was clearly recorded in the table of the original article. Therefore, we excluded the data of these three patients. The data of Bone 1997 in the current basi c characteristics table is the information of the remaining patients ≥18.

Appendix S6. Summary of 45 studies for the qualitative synthesis about the impacts of smoking and alcohol consumption

Summary of 45 studies for the qualitative synthesis about the impacts of smoking and alcohol consumption was described in **Appendix S6a to d**.

S6a. Summary of 45 studies for the qualitative synthesis (part 1)

No	First author/ Publication year	Journal name	country	Study design	Sex M/F	Age/yr mean±SD (range)	Race	BMI (kg/m^2)	Height (cm)	Weight (kg)
1	Arastu 2013[59]	International Orthopaedics	England	case series	22/9	Mean 45·6 (24–64)	NA	NA	NA	NA
2	Bhandari 2004[60]	Journal of Orthopaedic Trauma	Canada	case series	17/13	51·6±15·2 (18–81)	NA	NA	NA	NA
3	Sohn 2015[61]	Injury	Korea	retrospective cohort study	30/3	Mean 45·7 (18–70)	NA	Mean 24·21	NA	NA
4	Unlu 2015[62]	The Journal of Foot & Ankle Surgery	Turkey	case series	12/8	Mean 38 (18–71)	NA	NA	NA	NA
5	Formica 2016[63]	Injury	Italy	case series	27/16	Mean 44·8 (23–61)	NA	Mean 26·7 (18·4–33·1)	NA	NA
6	Frantz 2019[64]	Current Orthopaedic Practice	USA	retrospective cohort study	69/66	Mean 40·7 (18–90)	NA	Mean 29·8 (18·5–74·4)	NA	NA
7	Pastides 2015[65]	The Journal of Foot & Ankle Surgery	UK	case series	20/10	Mean 39·64 (27–63)	NA	NA	NA	NA
8	Burnett 2019[66]	Techniques in Orthopaedics	USA	retrospective cohort study	19/6	44·12±19·16 (18–84)	NA	NA	NA	NA
9	Johnston 2014[67]	Hip International	England	retrospective cohort study	NA because data included hemiarthroplasty	NA because data included hemiarthroplasty	NA	NA	NA	NA
10	Nyquist 1997[68]	Alcohol & Alcoholism	Sweden	retrospective cohort study	GA (49/0)<br>ConA (150/0)<br>Total (199/0)	GA 44±10 (19–60)<br>ConA 37 ± 13 (18–60)<br>Total 38·72	NA	NA	NA	NA
11	Tonnesen 1991[69]	The Journal of bone and joint surgery. British volume	Denmark	retrospective cohort study	GA (90/0)<br>ConA (90/0)<br>Total (180/0)	GA 46±1<br>ConA 45±2<br>Total 45·5	NA	NA	NA	GA 81±2<br>ConA 80±1<br>Total 80·5
12	Lin 2018[70]	The Journal of Foot & Ankle Surgery	China	retrospective cohort study	99/30	Mean 38·57	NA	NA	NA	NA
13	White 2010[71]	Journal of Orthopaedic Trauma	Canada	case series	NA	Mean 44 (19–68)	NA	NA	NA	NA
14	krishnan 2015[72]	Craniomaxillofacial Trauma and Reconstruction	India	retrospective cohort study	GS (15/0)<br>ConS (18/1)<br>GA (26/0)	GS 36·8±9·6 (20–53)<br>ConS 32·74±8·89 (21–50)<br>GA 35·11±9·68 (20–53)	NA	NA	NA	NA

					ConA (7/1)<br>Total (33/1)	ConA 32·63±8·23 (25–48)<br>Total 34·53±9·30 (20–53)				
15	Zura 2016[73]	JAMA Surgery	USA	case-control study	130,378/178,952	44·48±13·68 (18–63)	NA	NA	NA	NA
16	Gaal 2016[74]	Journal of Oral and Maxillofacial Surgery	USA	retrospective cohort study	441/69	29·3±11·4	NA	25·22±4·97	NA	NA
17	Sun 2018[75]	Medicine	China	case-control study	843/667	Mean 47·9 (18–86)	NA	Mean 24·74	NA	NA
18	Zura 2017[76]	Bone	USA	case-control study	15,211/41,281	Mean 74·85	NA	NA	NA	NA
19	Boesmueller 2015[77]	Injury	Australia	case-control study	61/93	55·84±16·9 (19–91)	NA	NA	NA	NA
20	Cook 1977[78]	Clinical Orthopaedics and Related Research	US	mixing of prospective and retrospective cohort study	53/14	Tibial shaft fracture<br>Mean 33·46	NA	NA	NA	NA
21	Dailey 2018[79]	Journal of Orthopaedic Trauma	UK	case-control study	739/264	NA	NA	NA	NA	NA
22	Dingemans 2018[80]	International Orthopaedics	Netherlands	prospective cohort study	75/25	Mean 43·10	NA	(16·6–42)	NA	NA
23	Femino 2010[81]	The Iowa Orthopaedic Journal	US	case series	12/1	Mean 45·1 (26–71)	NA	NA	NA	NA
24	Giannoudis 2000[82]	The Journal of bone and joint surgery. British volume	England	case-control study	NA<br>because description of data was wrong	Mean 37·03 (18–85)	NA	NA	NA	NA
25	Moghaddam 2010[83]	Injury	Germany	case-control study	GS (10/4)<br>ConS (10/4)<br>Total (20/8)	GS 43·93±12·97 (23–62)<br>ConS 51·43±9·67 (41–75)<br>Total 47·68±11·86 (23–75)	NA	GS 25·54±2·51 (21·1–29·8)<br>ConS 25·11±1·69 (22·7–27·8)<br>Total 25·33±2·11 (21·1–29·8)	NA	NA
26	Rose 2007[84]	Journal of Shoulder and Elbow Surgery	US	case series	7/9	Mean 51 (18–78)	NA	NA	NA	NA
27	Thompson 2017[85]	The Journal of Foot & Ankle Surgery	US	retrospective cohort study	17/47	Mean 49·16 (19–85)	NA	NA	NA	NA
28	Li 2015[86]	Surgical Infection	China	case-control study	225/113	Mean 37·2±8·4 (29–52)	NA	Mean 22·82	NA	NA
29	Xu 2019[87]	ANZ Journal of Surgery	China	case-control study	273/161	49·6±16·5 (18–79)	NA	NA	NA	NA
30	Zhu 2017[88]	International Wound Journal	China	case-control study	164/71	Mean 45·12 (19–75)	NA	NA	NA	NA
31	Fourgeaux 2019[89]	International Orthopaedics	France	case series	20/6	39±16 (21–90)	NA	NA	NA	NA
32	Hoffmann 2019[90]	Journal of Orthopaedic Surgery and Research	Germany	retrospective cohort study	80/113	Mean 70·2 (19–96)	NA	Mean 26·7 (15·6–54·7)	NA	NA

33	Serrano 2020[91]	Journal of Orthopaedic Trauma	US	case-control study	556/626	Mean 48 (18–101)	African American (197) Caucasian (624) Hispanic (81) Other (40) Not specified (239)	Mean 24 (16–44)	NA	NA
34	Hu 2018[92]	Foot & Ankle International	China	case-control study	127/169	48·6±15·2 (20–84)	NA	27·3±4·3	NA	NA
35	Alemdaroglu 2009[93]	Injury	Turkey	case-control study	NA	Mean 45·3 (19–75)	NA	NA	NA	NA
36	Grun 2020[94]	Foot & Ankle International	Norway	case series	23/2	Mean 44 (21–72)	NA	NA	NA	NA
37	Vander Voort 2020[95]	Iowa Orthop J	US	case-control study	67/33	43·3±17·4 (18–90)	NA	30·2±6·9	NA	NA
38	Baris 2020[96]	Acta Orthopaedica et Traumatologica Turcica	Turkey	retrospective cohort study	41/19	45·3±14·9 (18–75)	NA	NA	NA	NA
39	O'Halloran K 2016[97]	Clin Orthop Relat Res	US	case-control study	289/93	Mean 39·3 (18–85)	White (232) Nonwhite (150)	NA	NA	NA
40	Naumann 2016[98]	Foot and Ankle Surgery	Norway	case-control study	245/322	53·2±15·1 (18·1–86·2)	NA	NA	NA	NA
41	Kawasaki 2021[99]	Eur J Orthop Surg Traumatol	Japan	case-control study	184/74	Mean 45·8 (29–61)	NA	23.26	NA	NA
42	Ahmed 2020[100]	Medical Forum Monthly	Pakistan	case series	55/5	37·17±13·5	NA	NA	NA	NA
43	Cammas 2020[101]	Acta Orthop. Belg	Belgium	case series	187/246	Mean 51 (18–91)	NA	Mean 27 (16·8–53·1)	NA	NA
44	Halonen 2021[102]	Injury	Finland	case-control study	315/680	Mean 80·5 (29–104)	NA	NA	NA	NA
45	Goudie 2021[103]	The Journal of Bone and Joint Surgery	UK	case-control study	547/1683	median 72, 73	NA	NA	NA	NA

Note: M: male; F: female; SD: standard deviation; BMI: body mass index; NA: not available; GS: smoking group; ConS: control group in smoking compari

son; GA: alcohol drinking group; ConA: control group in alcohol drinking comparison.

S6b. Summary of 45 studies for the qualitative synthesis (part 2)

No	First author/ Publication year	Inclusion and exclusion criteria	type of fracture (traumatic/non-pathological/non-metastatic fracture, etc)	Non-pathological fracture or unclear	Fracture location	Diabetes	Use of NSAIDs	Use of fluoro-quinolone family of antibiotics
1	Arastu 2013	**Inclusion criteria:**<br>Between January 2000 and December 2012, 31 patients with displaced intra-articular calcaneal fractures not deemed suitable for an extensile lateral approach and internal fixation were identified. They were treated with MIRF by a single surgeon (senior author) in a Level 1 trauma centre.<br>**Exclusion criteria:**<br>Sanders type IV fractures	NA	Unclear	Calcaneus	Total (2)	NA	NA
2	Bhandari 2004	**Inclusion criteria:**<br>1. age 18 years or older<br>2. isolated injury to the ankle<br>3. unstable ankle fracture (OTA 44B, Weber type B) requiring operative fixation<br>4. patient consent<br>**Exclusion criteria:**<br>1. patients with multiple fractures<br>2. patients who were deemed incapable of completing quality of life questionnaires because of factors such as cognitive impairment or<br>inability to communicate in English.	NA	Unclear	Ankle	Total (2)	Total (2)	NA
3	Sohn 2015	**Inclusion criteria:**<br>1. patients who are skeletally mature (age≥18 years)<br>2. patients with a displaced clavicular midshaft fracture (no cortical contact between medial and distal fragment and/or more than 2-cm shortening)<br>3. patients who were underwent operation with superior plating by either open plating with interfragmentary screw fixation or minimally invasive plate osteosynthesis (MIPO)<br>4. patients who were followed up during a minimum 1-year.<br>**Exclusion criteria:**<br>1. age more than 70 years.<br>2. fractures more than 3 weeks between the injury and the operation.<br>3. open fractures.	Non-pathological fracture	Non-pathological fracture	Clavicle	Total (4)	NA	NA

		4. pathologic fractures.<br>5. previous morbidity of the affected upper extremity, including shoulder girdle.<br>6. bilateral clavicle fractures at the initial trauma or previous fracture history of the contralateral clavicle.<br>7. presence of neurovascular compromise from the initial trauma.						
4	Unlu 2015	**Inclusion criteria:**<br>patients treated in our clinic from April 2009 to January 2011 for distal fibular and tibial fractures at the same level were included in the present study.<br>**Exclusion criteria:**<br>1. patients with grade III open fractures and multiple fragments at the medial distal tibia<br>2. patients with a medial malleolus fracture, patients who had undergone fixation with a low-profile medial distal tibial plate<br>3. patients with accompanying injuries.	NA	Unclear	Fibula and tibia	Total (2)	NA	NA
5	Formica 2016	**Inclusion criteria:**<br>Seventy-six consecutive patients affected by thoracolumbar fractures from January 2012 to December 2013 were included in the study. Indications for management with intermediate screws were:<br>1. fresh burst unstable fractures (AO-type A3, A4) or combined<br>fractures with posterior ligamentous complex injuries (AO-type B2)<br>2. thoracolumbar fractures between T9 and L4<br>3. integrity of the pedicles at the fracture level allowing for the insertion of the bilateral intermediate screws.<br>**Exclusion criteria:**<br>1. stable thoracolumbar fracture (AO-type A1, A2)<br>2. vertebral injury pattern involving the pedicles<br>3. skeletal immaturity or osteoporotic vertebral fractures.	NA	Unclear	Thoracolumbar	Total (5)	NA	NA
6	Frantz 2019	**Inclusion criteria:**<br>A single institution retrospective review of medical records was performed at a Level one academic trauma center on all patients undergoing operative fixation of diaphyseal humeral fractures (AO/OTA Classification – 12-A1 to 12-C3; current procedural terminology [CPT] code 24515) from 2006 to 2016 with plate osteosynthesis.<br>1. All fracture patterns of these subtypes were included.<br>2. Patients had to be older than 18 year of age<br>3. speak English as their primary language.<br>**Exclusion criteria:**<br>1. incomplete records, radiographs, or follow up	Non-pathological fracture	Non-pathological fracture	Humerus	NA	NA	NA

		2. patients with known inflammatory arthropathies, immunomodulating disorders, or metabolic bone disorders<br>3. pathologic fracture<br>4. revision surgical cases<br>5. concurrent injury in the same bone (i.e. undergoing simultaneous arthroplasty).						
7	Pastides 2015	**Inclusion criteria:**<br>All closed displaced calcaneal fractures were considered for surgery and classified according to the Sanders classification on the preoperative computed tomography (CT) scans.<br>**Exclusion criteria:**<br>1. Open fractures were excluded<br>2. Types 1 and 4	NA	Unclear	Calcaneus	NA	NA	NA
8	Burnett 2019	**Inclusion criteria:**<br>1. patients who were skeletally mature, below 85 years of age<br>2. adequate follow-up to fracture healing<br>**Exclusion criteria:**<br>patients who had nontongue type intra-articular fractures.	NA	Unclear	Calcaneus	NA	NA	NA
9	Johnston 2014	**Inclusion criteria:**<br>The data for 7,023 hip fracture patients was collected on admission to a district general hospital. Questionnaires were completed by the admitting doctor from the history given by the patient and carers. Medical history was obtained from the patients and confirmed with their medical notes.<br>**Exclusion criteria:** NA	NA	Unclear	hip	NA because data included hemiarthropla sty	NA	NA
10	Nyquist 1997	**Inclusion criteria:**<br>From 1980 to 1990, a total of 218 male patients between 18 and 60 years of age were treated at Malmd University Hospital because of isolated tibial shaft fractures. Malmo University Hospital is the only hospital providing medical care to the city residents.<br>**Exclusion criteria:**<br>1. primary amputation<br>2. lethal complications<br>3. incomplete or missing records or final treatment elsewhere	NA	Unclear	Tibia	NA	NA	NA
11	Tonnesen 1991	**Inclusion criteria:**<br>Between 1977 and 1988, 626 men with closed ankle fractures were admitted for open reduction and fixation<br>**Exclusion criteria:** NA	NA	Unclear	ankle	GA (2)<br>ConA (2)<br>Total (4)	NA	NA
12	Lin 2018	**Inclusion criteria:**<br>The present retrospective study included 129 calcaneal fracture patients who had undergone open reduction and internal fixation in our hospital from January 2008 to	NA	Unclear	calcaneus	Total (0)	NA	NA

		December 2010.<br>**Exclusion criteria:**<br>1. concurrent fractures that might cause postoperative chronic pain such as tibial, fibula, metatarsal, and phalangeal fractures<br>2. preexisting calcaneal pain<br>3. concurrent peripheral nerve injury, including diabetic foot disease and tarsal tunnel syndrome<br>4. concurrent systemic diseases, such as hypertension, coronary atherosclerotic heart disease<br>5. diabetes.						
13	White 2010	**Inclusion criteria:**<br>patients who had sustained OTA 43.C-type injuries treated by early ORIF with a minimum 1-year follow up.<br>**Exclusion criteria:**<br>1. patients who could not complete follow up<br>2. patients in whom primary treatment could not be performed<br>3. patients who died within 30 days of admission from associated injuries<br>4. patients who were transferred out of the catchment area<br>5. patients who failed to attend follow up.	NA	Unclear	Tibia	Total (6)	NA	NA
14	krishnan 2015	**Inclusion criteria:**<br>All adult patients who reported to the department of dentistry in a public<br>sector, tertiary care hospital and level 1 trauma center, with fractures of the dentate portion of the mandible over a 3-year period (January 2011–January 2014), were assessed for inclusion in the study<br>**Exclusion criteria:** NA	NA	Unclear	Mandible	NA	NA	NA
15	Zura 2016	**Inclusion criteria:**<br>patients with a coded bone fracture in calendar year 2011.<br>**Exclusion criteria:**<br>Patients who had less than 12 months of continuous enrollment following fracture so as to capture all coded nonunions.	NA	Unclear	18 bones	Total (26,875)	Total (23,847)	NA
16	Gaal 2016	**Inclusion criteria:**<br>1. subjects must have been >18 years of age<br>2. patients who have had isolated open mandible fractures operatively managed using a transoral approach (surgery performed only through the mouth). Procedures performed included ORIF of one or multiple sites with or closed reduction and<br>intermaxillary fixation (CR-IMF) using any form of IMF technique.<br>**Exclusion criteria:**<br>1. Subjects who presented with any other fractures	NA	Unclear	Mandible	Total (21)	NA	NA

		2. complex mandibular fractures (e.g. requiring transcervical approach)<br>3. gunshot wounds<br>4. pre-existing concurrent mandibular fractures (e.g. new fractures during the healing of a previous fracture)<br>5. fracture site infections present at the time of initial presentation<br>6. closed mandibular fractures<br>7. immunocompromised status (e.g. human immunodeficiency virus [HIV], any immunosuppressive medication such as steroids)<br>8. patients who received antibiotics for any other indication.						
17	Sun 2018	**Inclusion criteria:**<br>The electronic medical records (EMRs) were searched to identify all adult patients (18 years and older) who had undergone ORIF to treat ankle fracture in the period from January 2015 to December 2016.<br>**Exclusion criteria:**<br>1. patient age<18 years<br>2. pathological fractures<br>3. old fractures (>21 days after initial injury)<br>4. treatments rather than ORIF (external fixation, conservative treatments, manual reduction and internal fixation, traction).	Non-pathological fracture, non-old fracture	Non-pathological fracture	Ankle	Total (104)	NA	NA
18	Zura 2017	**Inclusion criteria:**<br>We analysed all Medicare beneficiaries with ≥1 fracture diagnosis in the 18 bones most frequently fractured.<br>**Exclusion criteria:**<br>1. patients who did not have both Medicare Part A and Part B eligibility in all of 2011 and 2012, so that a code for nonunion could be captured.<br>2. a malunion claimor if the 2011 claim was for nonunion of a prior fracture.	NA	Unclear	18 bones	Total (21,600)	NA	NA
19	Boesmueller 2015	**Inclusion criteria:**<br>1. presence of proximal humeral fracture<br>2. known time of trauma<br>3. surgical treatment with ORIF and Philos plate (Synthes, Oberdorf, Switzerland)<br>4. availability of at least 6 months of radiological follow-up after surgery<br>5. a complete data set.<br>**Exclusion criteria:**<br>1. patients undergoing conservative treatment<br>2. patients presenting with pathologic or open fractures<br>3. patients with implantation of a primary fracture prosthesis<br>4. patients with a previous injury or surgery on the ipsilateral	Non-pathological fracture	Non-pathological fracture	Humerus	NA	NA	NA

		shoulder<br>5. patients with an incomplete data set<br>6. patients with a follow-up of <6 months<br>7. patients under 18 years of age						
20	Cook 1977	**Inclusion criteria:**<br>Skeletally mature men and nonpregnant women who had a closed or Grade I open tibial diaphyseal fracture that was primarily transverse, short<br>oblique, or short spiral and that could be treated effectively with closed reduction and immobilization in a cast were eligible for inclusion in the<br>study.<br>**Exclusion criteria:**<br>1. patients in whom either the prereduction or postreduction anteroposterior (AP) or the lateral radiographs showed that the length of the fracture line was more than twice the diameter of the diaphyseal shaft (a long spiral or long oblique fracture), the postreduction displacement was more than 50% of the width of the shaft, or the postreduction fracture gap averaged more than 0.5 cm.<br>2. open fractures, except Grade I as defined by Gustilo and Andersonx; fractures of the tibial metaphysis; fractures with persistent shortening of more than 1 cm after reduction<br>3. fractures that were not sufficiently stable for treatment with immobilization in an above the knee cast<br>4. fractures with a large butterfly fragment (larger than 2 times the diameter of the tibial shaft)<br>5. pathologic fractures<br>6. comminuted fractures (comminution with fragments of less than 1 cm in length was acceptable)<br>7. patients who were receiving steroids, anticoagulants, prescription nonsteroidal anti-inflammatory medication, calcium channel blockers, or diphosphonate therapy<br>8. patients who had a history of thrombophlebitis or vascular insufficiency<br>9. patients who had a recent history of alcoholism or nutritional deficiency, or both.	Non-pathological fracture	Non-pathological fracture	Tibia	NA	Tibial fracture Total (0)	NA
21	Dailey 2018	**Inclusion criteria:**<br>1. Records for 2,211 tibial fracture patients treated during the study period were filtered for inclusion<br>2. Adult patients (age 18+)<br>3. Primary IM nailing case<br>**Exclusion criteria:**<br>1. Casts<br>2. All other implants, including secondary nailing after failed	Non-pathological fracture	Non-pathological fracture	Tibia	NA	NA	NA

		cast or exfix<br>3. patients who died as a result of injury or before union<br>4. patients who were lost to follow up<br>5. Followed-up elsewhere<br>6. Amputation undertaken<br>7. Pathological fracture						
22	Dingemans 2018	**Inclusion criteria:**<br>This study was a pilot study to investigate the feasibility of using a new, portable, negative pressure wound therapy device in patients undergoing lower extremity fracture surgery. The 3-cm threshold for inclusion was chosen to selectively include patients in whom open surgery and not percutaneous surgery was performed.<br>**Exclusion criteria:**<br>1. open fractures or active infections<br>2. antibiotic treatment at the time of the operation for a concomitant disease or infection<br>3. patients with immune deficiencies<br>4. incision location not suitable for negative pressure wound therapy device,<br>5. inability to adhere to therapy,<br>6. incomprehensive understanding of the Dutch language.	NA	Unclear	Lower extremity	Total (0)	NA	NA
23	Femino 2010	**Inclusion criteria:**<br>We retrospectively reviewed thirteen patients who had undergone open reduction and lateral plate fixation without bone graft of closed displaced intraarticular calcaneus fractures using an extensile sinus tarsi approach<br>**Exclusion criteria:** NA	NA	Unclear	Calcaneus	Total (1)	NA	NA
24	Giannoudis 2000	**Inclusion criteria:**<br>We reviewed retrospectively the record of 377 patients treated by intramedullary nailing for fractures of the shaft of the femur (AO 32) between 1991 and 1997 to identify those with nonunion.<br>**Exclusion criteria:**<br>Patients with head injuries	NA	Unclear	Femur	NA	postoperative use of NSAIDs Total (29)	NA
25	Moghaddam 2010	**Inclusion criteria:**<br>1. radiological evidence of a long bone fracture<br>2. an age of 18–80 years<br>3. informed and written consent prior to the study<br>**Exclusion criteria:**<br>1. multiple traumas (i.e., more than two fractures)<br>2. significant soft tissue damage<br>3. type II or III open fractures (according to the Gustilo classification), and/or postoperative respirator ventilation that was utilised for more than 24h	Non-metastatic fracture	Non-pathological fracture	Long bone	NA	Total (0)	NA

		4. Because serum levels of TGF-b1 can be influenced by various drugs and systemic disorders, patients suffering from systemic or organic diseases, acute or chronic inflammatory diseases as monitored by routine lab programs, malignancy, or obesity<br>5. patients with a history of thiazide use<br>6. immunosuppressive<br>7. long-term therapy with steroidal or non-steroidal anti-inflammatory<br>drugs, estrogens, anabolic steroids, calcium, fluoride, or calciotropic hormones						
26	Rose 2007	**Inclusion criteria:**<br>The Synthes LCP Locking Proximal Humerus Plate (Synthes, Paoli, PA) was used for the treatment of proximal humeral fractures in 16 patients. All patients underwent open reduction–internal fixation of their injuries via a deltopectoral approach while positioned in the beachchair position under general anesthesia.<br>**Exclusion criteria:** NA	NA	Unclear	Humerus	NA	NA	NA
27	Thompson 2017	**Inclusion criteria:**<br>1. patients with surgically managed fifth metatarsal diaphyseal fractures.<br>2. Diaphyseal fractures were chosen for surgical management according to the amount of displacement, shortening, and rotation of the distal fragment.<br>**Exclusion criteria:**<br>1. patients with non-diaphyseal fifth metatarsal fractures<br>2. conservatively managed fractures<br>3. follow-up <1 year<br>4. missing radiographic images<br>5. surgery performed by another surgeon	NA	Unclear	Metatarsal	Total (9)	NA	NA
28	Li 2015	**Inclusion criteria:**<br>All patients who underwent operative acetabular fracture treatment between June 2001 and June 2011 were included in the retrospective study.<br>**Exclusion criteria:** NA	NA	Unclear	Pelvis	Total (18)	NA	NA
29	Xu 2019	**Inclusion criteria:**<br>Between July 2014 and December 2017, patients above 18 years old who had a distal femur intra-articular fracture surgically treated by open plates/screws were included. The minimal post-operative follow-up period was 1 year, based on the postoperative follow-up strategies in our institution.<br>**Exclusion criteria:**<br>1. patients younger than 18 years<br>2. extra-articular femur fractures	Non-pathological fracture, non-metastatic fracture	Non-pathological fracture	Femur	NA	NA	NA

		3. old fractures (>28 days from fracture occurrence)<br>4. pathological or metastasis fractures<br>5. treatment by alternative methods of fracture stabilization other than open plate/screws osteosynthesis<br>6. patients admitted only for treatment of SSI but without undergoing index surgery at our institution.						
30	Zhu 2017	**Inclusion criteria:**<br>All patients aged 18 or older with acute tibial plateau fractures treated by ORIF<br>**Exclusion criteria:**<br>1. patients younger than 18 years<br>2. pathological fractures<br>3. old fractures (>21 days from initial injury) and reoperation for certain reasons.	Non-pathological fracture	Non-pathological fracture	Tibia	Total (11)	NA	NA
31	Fourgeaux2019	**Inclusion criteria:**<br>calcaneal fractures with the presence of a displaced intra-articular calcaneal fracture with loss of congruence of the subtalar joint.<br>**Exclusion criteria:**<br>1. patients with a follow-up period shorter than two years,<br>2. patients with open fracture or undisplaced fracture	NA	Unclear	Calcaneal	NA	NA	NA
32	Hoffmann 2019	**Inclusion criteria:**<br>intertrochanteric femur fracture with long intramedullary nail (IMN) fixation and age equal to or older than 18 years.<br>**Exclusion criteria:**<br>1. intramedullary fixation with a short nail or utilization of an extramedullary implant<br>2. follow up less than 6months<br>3. metastatic disease<br>4. insufficient medical record or radiographic data.	non-metastatic fracture	Non-pathological fracture	Femur	Total (40)	NA	NA
33	Serrano 2020	**Inclusion criteria:**<br>Patients with a humeral shaft fracture managed conservatively with a functional brace from 2005 to 2016 were retrospectively reviewed from 9 academic institutions across the United States.<br>**Exclusion criteria:**<br>1. Pathologic and periprosthetic fractures<br>2. diaphyseal injuries with joint extension<br>3. ipsilateral upper extremity injuries<br>4. Patients younger than 18 years<br>5. patients lost to follow-up before union or before change in treatment modality	Non-pathological fracture, non-periprosthetic fracture	Non-pathological fracture	Humerus	Total (180)	NA	NA

34	Hu J 2018	**Inclusion criteria:**<br>1. patients with a definitive diagnosis of a medial malleolar fracture based on clinical and imaging findings<br>2. patients who underwent ORIF surgery or percutaneous screw fixation<br>3. patients who were skeletally mature at the time of the injury<br>4. patients who had complete clinical follow up data for at least 12 months after the injury.<br>**Exclusion criteria:**<br>1. patients who had removal of the internal fixation device before fracture union due to complications, such as an infection or a wound healing disorder<br>2. patients who had significant complications related to lateral or posterior fractures, such as malreduction, redisplacement, delayed union, or nonunion<br>3. patients with incomplete information and/or with a shorter follow-up of less than 12 months.	NA	Unclear	Ankle	Total (46)	NA	NA
35	Alemdaroglu 2009	**Inclusion criteria:**<br>Adult patients suffering from tibial shaft fractures who were treated with circular external fixator between October 2002 and May 2007 were included in the study. These patients were treated with circular external fixator due to consensus in the department.<br>**Exclusion criteria:**<br>1. Patients with articular extension<br>2. patients who were lost to follow up	NA	Unclear	Tibia	Total (1)	NA	NA
36	Grun 2020	**Inclusion criteria:**<br>1. Closed, dislocated intra-articular calcaneal fracture treated with only percutaneous reduction and fixation and assisted by subtalar arthroscopy between 2013 and September 2018<br>2. Age 18 years or older at the time of injury<br>3. Availability for follow-up<br>**Exclusion criteria:**<br>1. patients who had moved abroad and therefore were unavailable for follow-up<br>2. patients who could not participate due to other relevant illness (eg, dementia)	NA	Unclear	Calcaneal	Total (1)	NA	NA
37	Vander Voort 2020	**Inclusion criteria:**<br>Surgical records were queried at a level I academic trauma center to identify individuals 18 years and older indicated for operative fixation of traumatic open fractures of the tibia and ankle between June 2006 and September 2017.<br>**Exclusion criteria:**<br>1. Patients without preoperative computed tomography (CT) scan including the lumbar spine as part of their trauma work	NA	Unclear	Tibia, ankle	NA	NA	NA

		up<br>2. patients who were lost to follow-up						
38	Baris 2020	**Inclusion criteria:**<br>From January 2011 to November 2015, we reviewed 60 patients (62 tibia) who were treated with open reduction and internal fixation for distal tibia pilon fractures who could be postoperatively followed for three years or more.<br>**Exclusion criteria:**<br>1. multiple fractures<br>2. previous foot or ankle surgery<br>3. accompanying neural or vascular pathology<br>4. neuropathic foot<br>5. pathologic fractures of the tibia<br>6. patients younger than 18 years<br>7. the loss in the follow-up.	Non-pathological fracture	Non-pathological fracture	Tibia	Total (5)	NA	NA
39	O'Halloran K 2016	**Inclusion criteria:**<br>1. patients aged 18 years and older<br>2. tibial shaft fractures treated at authors' center from 2007 to 2014<br>**Exclusion criteria:**<br>1. patients with adequate follow-up but indeterminate healing status<br>2. patients who were treated with anything other than reamed IM nail fixation<br>3. patients who had a tibiotalocalcaneal nail<br>4. patients who died before healing or nonunion<br>5. patients who had undergone early amputations<br>6. patients who had periprosthetic, stress, or pathologic fractures<br>7. patients who had definitive fixation performed at a different hospital<br>8. patients who had fractures involving the tibial plafond or knee joints that required operative intervention separate from the IM nail<br>9. pregnant patients, patients with insufficient cortical contact, patients with missing chart data, and nonoperative patients<br>10. patients with nonunion surgery planned before 3 months and those for whom the possibility of nonunion was noted before 3 months	Non-pathological fracture	Non-pathological fracture	Tibia	Total (23)	Total (47)	NA
40	Naumann 2016	**Inclusion criteria:**<br>all patients ≥18 years of age living in the catchment area who were treated for unstable and closed ankle fractures by ORIF between January 1, 2009 and December 31, 2011 were eligible	NA	Unclear	Ankle	Total (34)	NA	NA

		for inclusion in the study.<br>**Exclusion criteria:**<br>1. high energy trauma<br>2. polytrauma<br>3. other type of fracture in the limb<br>4. not from region AHUS or SOF<br>5. apoplexia/intoxication<br>6. fracture not in year 2009–2011<br>7. conservative treatment<br>8. previous fracture in the limb<br>9. open fracture<br>10. cognitive disease<br>11. patients who died<br>12. patients who moved out of area						
41	Kawasaki 2021	**Inclusion criteria:**<br>patients with tibial shaft fractures who were treated with intramedullary nail at our 12 facilities<br>**Exclusion criteria:**<br>1. patients who lost to follow-up 12 months after the operation<br>2. patients with missing data or inadequate radiographs	NA	Unclear	Tibia	NA	NA	NA
42	Ahmed 2020	**Inclusion criteria:**<br>patients presenting to us with Gustilo-Anderson I, II, IIIAOTFs	NA	Unclear	Tibia	NA	NA	NA
43	Cammas 2020	**Inclusion criteria:**<br>1. Patients treated surgically for ankle malleolus fracture from 2013 to 2017<br>2. All patients were over 18 years old<br>**Exclusion criteria:**<br>1. Patients with missing data or with pilon fractures and metaphyseal and/<br>or diaphyseal tibial fractures<br>2. Patients with history of bone and ankle joint infection, previous ankle fracture and those suffering from ankle osteoarthritis	NA	Unclear	Ankle	Total (30)	NA	NA
44	Halonen 2021	**Inclusion criteria:**<br>ICD-10 diagnoses coded as trochanteric fracture (S72.1) and with a procedure code for intramedullary fixation of proximal femoral fracture (NFJ54) by Nordic Classification of Surgical Procedures (NCSP)	NA	Unclear	Hip	Total (168)	NA	NA
45	Goudie 2021	**Inclusion criteria:**<br>1. Isolated, non-pathologic fracture through the humeral neck located within the epimetaphyseal region defined by the method of squares<br>2. locally resident<br>3. presentation within 2 weeks of injury	Non-pathological fracture	Non-pathological fracture	Humerus	NA	NA	NA

		4. no preexisting shoulder degenerative condition or previous surgery to the shoulder<br>**Exclusion criteria:**<br>1. Missing >3 of the 10 demographic data points studied<br>2. Double entry due to metachronous injury to contralateral shoulder<br>3. Inadequate follow-up until union status determined or loss to follow-up<br>4. Died before union status could be assessed<br>5. Primary operative treatment (Surgeon decision, Patient preference for operative treatment, Skin compromise, Neurovascular compromise, Fracture extending to diaphysis, Other)						

Note: NSAIDs: non-steroidal anti-inflammatory drugs; NA: not available; GA: alcohol drinking group; ConA: control group in alcohol drinking comparison; ORIF: open reduction and internal fixation.

S6c. Summary of 45 studies for the qualitative synthesis (part 3)

No	First author/ Publication year	Treatment	smoking type	smoking dose	Smoking group	Smoking Control group
1	Arastu 2013	minimally invasive reduction and fixation	NA	NA	Smokers (*n*=24)	Non-smokers (*n*=7)
2	Bhandari 2004	fixed with a lateral one-third semi-tubular plate	cigarette	31±25·9 (2–99) pack years	Smoking history (*n*=14)	Non-smoking history (*n*=16)
3	Sohn 2015	superior plating by either open plating with interfragmentary screw fixation or minimally invasive plate osteosynthesis (MIPO)	NA	NA	Current smokers (*n*=13)	Non-current smokers (*n*=20)
4	Unlu 2015	Minimally invasive plate osteosynthesis after fixing with a plate	NA	NA	Smoking history (*n*=3)	Non-smoking history (*n*=17)
5	Formica 2016	short-segment instrumentation with bilateral intermediate screws at the fracture level	NA	NA	Tobacco use (*n*=18)	Non-tobacco use (*n*=25)
6	Frantz 2019	operative fixation with plate osteosynthesis.	NA	NA	Smokers (*n*=36)	Non-smokers (*n*=60)
7	Pastides 2015	treated using arthroscopic reduction and percutaneous fixation: percutaneous arthroscopic calcaneal osteosynthesis (PACO)	cigarette	NA	Smokers (*n*=11)	Non-smokers (*n*=8)
8	Burnett 2019	cast immobilization<br>open reduction and fixation with plate fixation<br>closed reduction and percutaneous screw fixation	NA	NA	Smokers (*n*=NA)	Non-smokers (*n*=NA)
9	Johnston 2014	1. Intracapsular fractures treated by internal fixation<br>2. Extracapsular internal fixation	-	-	-	-
10	Nyquist 1997	1. dosed reduction and trans-fixational plaster	-	-	-	-

		2. Closed or no reduction with plaster from toes to groin<br>3. Closed reduction and intramedullary Ender nailing + PTB-plaster<br>4. External fixation with Hoffman<br>5. Closed reduction and intramedullary Kuntscher's nailing<br>6. Open reduction and lag screw fixation				
11	Tonnesen 1991	ORIF	-	-	-	-
12	Lin 2018	ORIF	-	-	-	-
13	White 2010	ORIF	-	-	-	-
14	krishnan 2015	Maxillomandibular fixation (MMF)/ ORIF/ conservative or minimal intervention	NA	NA	Smokers<br>(*n*=15)	Non-smokers<br>(*n*=19)
15	Zura 2016	any fracture surgery	NA	NA	Former or current smokers<br>(*n*=31,610)	Never smokers<br>(*n*=277,720)
16	Gaal 2016	ORIF of one or multiple sites with or closed reduction and intermaxillary fixation (CR-IMF) using any form of IMF technique	NA	NA	Smokers<br>(*n*=345)	Non-smokers<br>(*n*=165)
17	Sun 2018	ORIF	NA	NA	Current smoking<br>(*n*=355)	Non-current smoking<br>(*n*=1155)
18	Zura 2017	NA	NA	NA	Former or current smokers<br>(*n*=4,584)	Non-smokers<br>(*n*=51,908)
19	Boesmueller 2015	ORIF using the Philos plate	cigarette	heavy smokers (>20 cigarettes per day)	heavy smokers<br>>20cig/d<br>(*n*=12)	smoking≤20cig/d<br>(*n*=42)<br>Non-smokers<br>(*n*=100)
20	Cook 1977	closed reduction and immobilization in a cast	cigarette	NA	Smokers<br>(*n*=21)	Non-smokers<br>(*n*=39)
21	Dailey 2018	Reamed intramedullary nailing	NA	NA	Smokers<br>(*n*=244)	Non-smokers<br>(*n*=261)
22	Dingemans 2018	negative pressure wound therapy following surgery	NA	NA	Smoking<br>(*n*=44)	Non-smoking<br>(*n*=56)
23	Femino 2010	open reduction and lateral plate fixation without bone graft	NA	average 1·5 packs per day	Chronic smokers (*n*=7)	Non-chronic smokers<br>(*n*=6)
24	Giannoudis 2000	intramedullary nailing	cigarette	>20 cigarettes per day	Heavy smokers<br>(*n*=31)	Non-smokers<br>(*n*=68)
25	Moghaddam 2010	osteosynthetic procedure	cigarette	>5 cigarettes/day	Smokers<br>(*n*=14)	Non-smokers<br>(*n*=14)
26	Rose 2007	ORIF	NA	NA	Smokers<br>(*n*=5)	Non-smokers<br>(*n*=11)
27	Thompson 2017	treated surgically within the Beaumont Healthcare System	NA	NA	Current and former smokers<br>(*n*=25)	Non-smokers<br>(*n*=39)
28	Li 2015	ORIF	NA	NA	Active smokers<br>(*n*=100)	Non-smokers<br>(*n*=238)
29	Xu 2019	open plates/screws	NA	NA	Current smoking	Non-current smoking

					(*n*=NA)	(*n*=NA)
30	Zhu 2017	ORIF	NA	NA	Current smoking (*n*=22)	Never smokers (*n*=213)
31	Fourgeaux2019	Calcanail implant	NA	NA	Active smokers (*n*=10)	Non-active smokers (*n*=16)
32	Hoffmann 2019	Open or closed reduction and internal fixation	NA	NA	Active Smoker and History of smoking (*n*=112)	Non-smoking (*n*=81)
33	Serrano 2020	conversion of closed treatment to surgical intervention	NA	NA	Smoking (*n*=287)	Non-smoking (*n*=674)
34	Hu J 2018	ORIF or percutaneous screw fixation (PSF)	NA	NA	Tobacco use (*n*=21)	Non-tobacco use (*n*=275)
35	Alemdaroglu 2009	circular external fixator	NA	NA	Smoking (*n*=13)	Non-smoking (*n*=19)
36	Grun 2020	percutaneous reduction and fixation	NA	NA	Smoking (*n*=10)	Non-smoking (*n*=15)
37	Vander Voort 2020	operative fixation	NA	NA	Current smoker (*n*=35)	Non-current smokers (*n*=65)
38	Baris 2020	ORIF	NA	NA	Smoking (*n*=32)	Non-smoking (*n*=28)
39	O'Halloran K 2016	reamed IM nail fixation	NA	NA	Smoking (*n*=153)	Non-smoking (*n*=229)
40	Naumann 201 6	ORIF	NA	NA	Current smoking (*n*=136)	Non-current smokers (*n*=431)
41	Kawasaki 202 1	Intramedullary nailing	NA	NA	Current smoker (*n*=77)	Non-current smokers (*n*=181)
42	Ahmed 2020	Intramedullary nailing	NA	NA	Smoker (*n*=14)	Non-smoker (*n*=46)
43	Cammas 2020	ORIF	NA	NA	Active Smoker (*n*=94)	Non-smoker (*n*=339)
44	Halonen 2021	intramedullary fixation	NA	NA	Current smoker (*n*=100)	non-current smoker (*n*=895)
45	Goudie 2021	nonoperative treatment	cigarette	1. >5 cigarettes or equivalent /day<br>2. Nonsmoker or smokes ≤5 cigarettes or equivalent/day	Current smoker (>5 cigarettes or equivalent/day) (*n*=235)	Nonsmoker or smokes ≤5 cigarettes or equivalent/day (*n*=1,995)

Note: NA: not available; ORIF: open reduction and internal fixation

S6d. Summary of 45 studies for the qualitative synthesis (part 4)

No	First author/ Publication year	Alcohol type	Level of alcohol consumption	Alcohol group	Alcohol Control	Follow-up mean± SD, range	Description of results and reasons for not including in meta-analysis
1	Arastu 2013	NA	NA	Alcohol abusers (n=3)	Non-abusers (n=28)	14·9 (7–30) months	It was mentioned that there were no problems with wound healing in the follow-up period except one case of superficial wound infection. It means there was no event of deep infection occurred. However, there was no description whether the case was in smoker, alcohol abuser or control group.
2	Bhandari 2004	NA	6·7±17·8 (2–75) drinks/week	Alcohol consumption (n=13)	Non-alcohol consumption (n=17)	24 months	No patient experienced a deep infection, nonunion or malunion during the follow-up period.
3	Sohn 2015	NA	heavy alcoholics: >14 standard drinks per week or 4 drinks per day or more than 600ml of alcohol per day	Heavy alcoholics (n=7)	Non-heavy alcoholics (n=26)	17·6 (12–44) months	There was no superficial infection or deep infection event occurred in both groups. And there was no description that whether the only one nonunion event occurred to current smoker, heavy alcoholic or control group.
4	Unlu 2015	NA	NA	Alcohol history (n=1)	Non-alcohol history (n=19)	15·5 (12–26) months	The mean interval to bony union was 21 (18–25) weeks in total patients. It means that all fractures developed delayed union or nonunion without normal union. However, no data of time to union for each group was described.
5	Formica 2016	-	-	-	-	1 year	No cases of nonunion were observed. There was no description that only one superficial infection occurred to tobacco user or control group.
6	Frantz 2019	-	-	-	-	Follow-up was performed NA	The mean time to union was 22·8 weeks (n=36) in smoker group and 20·3 weeks (n=60) in non-smoker group without description of SD value.
7	Pastides 2015	-	-	-	-	24 (5–57) months	No deep infections developed in both groups.
8	Burnett 2019	-	-	-	-	Follow-up was performed NA	The mean time to full fracture healing assessed via radiographs was 3·7 (2–7) months in total patients. No significant difference was detected in the time to full healing in smokers compared with nonsmokers (p=0·26).
9	Johnston 2014	NA	alcoholics: more than 14 units per week for women and 21 units per week for men	Alcoholics (n=115)	Non-alcoholics (n=4,656)	1 year	Nonunion rates were 12 (n=115) and 289 (n=4,656) in alcohol drinking group and control group, respectively. However, control group (non-alcoholics) may include alcohol drinkers.
10	Nyquist 1997	NA	NA	Alcohol abusers (n=49)	Non-abusers (n=150)	14 years	Delayed union rates (no bone healing within 250 days) were 4 (n=49) and 16 (n=150) in alcohol drinking group and control group, respectively. And no difference was found between two groups. In addition, deep infection was seen in 1 alcohol abuser and 3 non-abusers. However, control group (non-abusers) may include alcohol drinkers

11	Tonnesen 1991	NA	alcohol abusers: at least 5 Danish drinks (60g alcohol) a day	Alcohol abusers (n=90)	Non-abusers (n=90)	12 weeks	Infection rates were 14 (n=90) and 4 (n=90) in alcoholics group and control groups. In addition, no malunion was found in both groups. However, control group (non-alcoholics) may include alcohol drinkers
12	Lin 2018	NA	alcoholism: >60g/day non-alcoholism: ≤60g/day	Alcoholism (n=32)	Non-alcoholism (n=97)	53·39 (48–60) months	Alcoholism (OR 1·679, p<0·05) was found one of the risk factors for postoperative wound infection through logistic regression analysis. However, control group (non-alcoholism) may include alcohol drinkers
13	White 2010	NA	NA	Alcohol abuse (n=5)	Non-abusers (n=90)	minimum 1 year	Two patients with alcohol dependency developed deep infection (n=5) and 4 patients in control group develop deep infection (n=90). However, control group (non-alcohol abuse) may include alcohol drinkers
14	krishnan 2015	NA	NA	Alcohol abuse (n=26)	Non-alcohol abuse (n=8)	(12–14) weeks	Outcomes of infection for each patient were listed in a table. In total, 4 smoking abuse (n=15) and 3 controls (n=18) developed infection, respectively. And 6 alcoholism (n=22) and 1 control (n=7) developed infection. However, control group (non-smoking abuse and non-alcoholism) may include current smokers, former smokers or alcohol drinkers
15	Zura 2016	NA	NA	Alcoholism (n=5,616)	Non-alcoholism (n=303,714)	at least 12 months	Nonunion rate was 2,313 (n=31,610) and 12,936 (n=277,720) in smokers and non-smokers, and the rate was 418 (n=5,616) and 14,831 (n=303,714) in alcoholism and non-alcoholism, respectively. Regarding normal union rate was 29,297 (n=31,610) and 264,784 (n=277,720) in smoking group and non-smoking group, 5,198 (n=5,616) and 288,883 (n=303,714) in alcoholism group and non-alcoholism group, respectively. However, alcohol control group may include alcohol drinkers. Definition of nonunion and normal healing was described without time period.
16	Gaal 2016	NA	NA	Alcohol use (n=337)	Non-alcohol use (n=173)	Follow-up was performed NA	There were 70 smokers (n=345), 10 non-smokers (n=165), 53 drinkers (n=337) and 27 (n=173) developed surgical site infection. However, control group (non-active smoking) may include former smokers.
17	Sun 2018	NA	NA	Alcohol abuse (n=150)	Non-alcohol abuse (n=1,360)	NA	Surgical site infection rate were 13 current smokers (n=355), 53 non-smokers (n=1,155), 11 alcohol abusers (n=150) and 55 non-drinkers (n=1,360), respectively. Control group (non-current smoking, non-alcoholism) may include former smokers and alcohol drinkers.
18	Zura 2017	NA	NA	Alcoholism (n=467)	Non-alcoholism (n=56,025)	NA	Nonunion occurred in 189 current or former smokers (n=4,584), 1251 non-smokers (n=51,908), 24 alcoholism (n=467) and 1,416 non-alcoholism (n=56,025), respectively. Regarding normal union, the results were 4,395 current or former smokers (n=4,584), 50,657 non-smokers (n=51,908), 443 alcoholism (n=467) and 54,609 non-alcoholism (n=56,025), respectively. Alcohol control group may include alcohol drinkers. Definition of nonunion and normal healing was described without time period.

19	Boesmueller 2015	-	-	-	-	Total 479 days (≥6 months)	Nonunion occurred to 4 heavy smokers (*n*=12) and 16 non-heavy smokers (*n*=142), respectively. Control group included never smokers and smokers who smoked ≤20 cigarettes per day.
20	Cook 1977	-	-	-	-	Tibial fracture 52 weeks	Delayed union occurred to 5 smokers (*n*=21) and 6 non-smokers (*n*=39), respectively. Definition of delayed union which were fractures not healed at 150 days is not applicable in the current study.
21	Dailey 2018	-	-	-	-	Follow-up was performed NA	There were 38 of 244 smokers and 36 of 261 non-smokers developed nonunion. Definition of nonunion was described without time period.
22	Dingemans 2018	-	-	-	-	Follow-up was performed NA	One of 44 smokers and 1 of 56 non-smokers developed superficial surgical infection. And no smokers (*n*=44) and 2 non-smokers (*n*=56) developed deep infection. However, control group (non-substance abuse [smoking]) may include former smokers.
23	Femino 2010	-	-	-	-	19 (2–41) months	Surgical site infection rate was 0 of 7 in smoker group and 1 of 6 in non-smoker group, respectively. However, control group (non-chronic smokers) may include former smokers.
24	Giannoudis 2000	-	-	-	-	NA	Logistic regression analysis showed that smoking was not risk factor (OR 2·29, 95%CI [0·85–6·08], *p*=0·11) of nonunion. Definition of nonunion was described without time period.
25	Moghaddam 2010	-	-	-	-	6 months after trauma	Raw data of non-union group and union group was listed in a table. There were 7 of 14 smokers and 7 of non-smokers in non-union group and union group, respectively. No definitions of normal union and nonunion were described.
26	Rose 2007	-	-	-	-	12 (6–20) months	Three of 5 smokers and 1 of 11 non-smokers developed nonunion. In addition, 2 of 5 smokers and 10 of 11 non-smokers normally healed. Definition of fracture union was described without time period.
27	Thompson 2017	-	-	-	-	at least 1 year	There were 2 of 25 smokers developed delayed union, and 1 former smoker developed nonunion. Delayed union or nonunion did not found in non-smokers (*n*=39). And 22 of 25 smokers and all non-smokers were normally healed. However, there were no definitions of delayed union and nonunion.
28	Li 2015	-	-	-	-	Follow-up was performed NA	There were 4 of 100 smokers and 12 of 238 non-smokers developed surgical site infection. However, control group which is non-active smokers may include former smokers.
29	Xu 2019	-	-	-	-	at least 1 year	Multivariate logistic regression analysis showed that current smoking was one of risk factors for post-operative surgical site infection (OR 3·22, 95%CI [1·71–4·68], *p*<0·001). However, control group which is non-current smoking may include former smokers.
30	Zhu 2017	-	-	-	-	12 months	Univariate analysis showed that surgical site infection occurred to 4 of 22 smokers and 8 of 213 non-smokers, respectively. However, control group included history of smoking and very occasionally

							smoking <1 per 3 days, which is not never smoking.
31	Fourgeaux2019	-	-	-	-	2·8 years±8 months (24–48 months)	One of 10 active smokers and no non-smokers developed delayed wound healing. However, control group (non-active smokers) may include former smokers.
32	Hoffmann 2019	-	-	-	-	15·2 (6–97) months	Five of 32 active smokers and 2 of 81 non-smokers developed nonunion. However, definition of nonunion was described without time period.
33	Serrano 2020	NA	NA	Alcohol drinking (*n*=193)	Non-drinking (*n*=768)	4, 8 weeks, 3, 6 months	There were 67 of 287 smokers, 129 of 674 non-smokers, 51 of 193 alcohol drinking and 145 of 768 non-drinking developed nonunion. However, healing period of nonunion was not clear. In the study, 207 developed a nonunion at a mean of 23 weeks without range.
34	Hu J 2018	NA	NA	Alcohol use (*n*=35)	Non-alcohol use (*n*=261)	median 52 (12–118) months	Bone healing time was 11·5±5·7 weeks (*n*=21) in smoking group, 10·2±6·5 weeks (*n*=275) in non-smoking group, 13·5±10·0 weeks (*n*=35) in drinking group and 9·8±5·6 weeks (*n*=261) in non-drinking group, respectively. However, regarding time to union, patients with nonunion were regarded as having invalid data and were excluded in the counts.
35	Alemdaroglu 2009	-	-	-	-	at least 6 months	Time to union was 27·54±11·61 weeks (*n*=13) in smoking group and 21·37±5·08 weeks (*n*=19) in non-smoking group (*p*= 0·16), respectively. However, 2 fractures were still non-united at the 52nd week (end of follow-up period). Time to union of the 2 fractures was recorded as 52 weeks for the statistical analysis purpose of calculating healing time. Thus, the result was not actual.
36	Grun 2020	-	-	-	-	15 (12–33) months	There were no wound-healing complications or surgical site infections.
37	Vander Voort 2020	-	-	-	-	median 20 (3–97) months	Regarding to nonunion, no obvious difference was found between current smokers (7/35) and non-current smokers (11/65). For deep surgical site infection, there was also no obvious difference between current smokers (9/35) and non-current smokers (26/65). However, control group may include former smokers.
38	Baris 2020	-	-	-	-	42·7±4·6 (36–66) months	Time to union was obviously greater in smoking group (*n*=32) compared with nonsmoking group (*n*=28) (17·1±6·0 weeks vs. 15·2±2·3 weeks, *p*=0·04). However, time to union was not clearly defined.
39	O'Halloran K 2016	NA	NA	heavy drinker (*n*=104)	Non-drinker or social drinker (*n*=278)	minimum of 9 months	Nonunion rate was 29 (*n*=153) and 27 (*n*=229) in smokers and non-smokers, and the rate was 20 (*n*=104) and 36 (*n*=278) in heavy drinkers and non-drinkers or social drinkers, respectively. Regarding normal union rate was 124 (*n*=153) and 202 (*n*=229) in smoking group and non-smoking group, 84 (*n*=104) and 242 (*n*=278) in heavy drinker group and non-drinkers or social drinker group, respectively. However, alcohol control group included alcohol drinkers. Definition of nonunion and normal healing was described without time period.
40	Naumann 201 6	-	-	-	-	NA	Undefined infection rate was 10 (*n*=136) and 19 (*n*=431) in c urrent smoker group and non-current smoker group, respectivel

							y. However, control group may include former smoker.
41	Kawasaki 2021	-	-	-	-	12 months	Nonunion rate was 16 (*n*=77) in current smoker group and 26 (*n*=181) in non-current smoker group, respectively. However, control group may include former smoker.
42	Ahmed 2020	-	-	-	-	at least 6 months	Union rate was 8 (*n*=14) in smoker group and 38 (*n*=46) in non-smoker group. However, union was recorded without time period.
43	Cammas 2020	-	-	Alcohol abuse (*n*=NA)	Non-abuser (*n*=NA)	mean 1 year (30 days–5 years)	Nonunion rate was OR 2·998, 95%CI 1·27–7·06 between alcohol abuser and non-abuser. However, control group may include alcohol drinker.
44	Halonen 2021	-	-	Alcohol abuse (*n*=74)	Non-abuser (*n*=921)	2 years or until death	Surgical site infection rate was 6 (*n*=100) in current smoker and 22 (*n*=895) in non-current smoker group, 3 (*n*=74) in alcohol abuser group and 25 (*n*=921) in non-abuser group. However, control group may include former smoker or alcohol drinker.
45	Goudie 2021	-	-	>20 units of alcohol/week (*n*=142)	≤20 units of alcohol/week (*n*=2,088)	24 weeks	Nonunion rate was 70 (*n*=235) in current smoker group (>5 cigarettes or equivalent/day) and 161 (*n*=1,995) in nonsmoker or smoke≤5 cigarettes or equivalent/day group, 26 (*n*=142) in ≥20 units of alcohol/week group, and 205 (*n*=2,088) in ≤20 units of alcohol/week group. However, control group may include smoker and alcohol drinker.

Note: NA: not available; OR: odds ratio.

Appendix S7. Summary of 19 studies about the impacts of preoperative smoking cessation time, nicotine replacement therapy, and vaping

Summary of 19 studies about the impacts of preoperative smoking cessation time, nicotine replacement therapy (NRT), and vaping was described in **Appendix S7a to d**.

S7a. Summary of 19 studies concerning the impacts of preoperative smoking cessation time, NRT, and vaping (part 1)

No	First author/ Publication year	Journal name	country	Study design	Sex M/F	Age/yr mean±SD (range)	Race	BMI (kg/m^2)
1	Glassman 2000[104]	Spine	US	retrospective cohort study	202/155	Mean 43·91	NA	NA
2	Sørensen 2007[105]	Hernia	Denmark	Prospective cohort study	180/33	1. Standard advice group: median 56 (IQR 46–66)<br>2. Telephone group: median 54 (IQR 42–64)<br>3. Outpatient group: median 54 (IQR 45–65)	NA	Mean 24·85
3	Delaunay 2018[106]	Annales de chirurgie plastique esthétique	France	retrospective cohort study	12/209	41·2±9·95 (19–66)	NA	28·09±7·58 (20·1–36)
4	Padubidri 2001[107]	Plastic and Reconstructive Surgery	US	cohort study	0/748	Mean 49·12 (14–77)	NA	NA
5	Moller 2002[108]	Lancet	Denmark	randomised controlled trial	46/62	Mean 65·04 (30–85)	NA	Mean 26·52
6	Araco 2008[109]	Plastic and Reconstructive Surgery	UK, Italy	prospectively	33/51	42±23	NA	26±3
7	Chan 2006[110]	Annals of Plastic Surgery	UK	cohort study	NA	Mean 36·18	NA	Mean 26·72
8	Gravante 2007[111]	Obesity Surgery	UK, Italy	prospective cohort study	15/45	Mean 42.5	NA	55±8
9	Gravante 2008[112]	Aesth Plast Surg	UK, Italy	prospective cohort study	NA	54±5 (41–70)	NA	28·7±4 (20·4–39·8)
10	Herrero 2020[113]	Journal of Orthopaedics	US	cohort study	104/97	Mean 57·64	NA	Mean 30·55
11	Jung 2014[114]	Gastric Cancer	Korea	Retrospective cohort study (prospectively collected)	855/480	Mean 56·04	NA	Mean 23·8
12	Kuri 2005[115]	Anesthesiology	Japan	retrospective cohort study	150/38	Mean 59·06	NA	Mean 21·02
13	Quan 2019[116]	World Journal of Surgical Oncology	China	retrospective cohort study	1624/845	Mean 55·44	NA	Mean 21·83
14	Sorensen 2003a[117]	Colorectal Disease	Denmark	prospective cohort study	37/20	Intervention group: median 65 (34–87)	NA	Mean 23·71

						control group:<br>median 66 (42–83)		
15	Thomsen 2010[118]	Nicotine & Tobacco Research	Denmark	randomised controlled trial	0/130	Intervention group:<br>median 57·5 (35–79)<br>control group:<br>median 56·5 (36–82)	NA	Intervention group:<br>median 24·2 (15·2–36·2)<br>control group:<br>median 23·1 (16·5–37·9)
16	Yoshida 2015[119]	World Journal of Surgery	Japan	retrospective cohort study	208/38	65·8±8·8	NA	NA
17	Sorensen 2003b[120]	Annals of surgery	Denmark	cohort study	39/39	Mean 30·31 (20–40)	NA	Mean 23·28
18	Sorensen 2010[121]	Surgery	UK	cohort study	31/31	Smokers:<br>median 33 (20–40)<br>Never smoker:<br>median 26 (20–40)	NA	NA
19	Lindström 2008	Annals of surgery	Sweden	randomised controlled trial	54/48	intervention:<br>median 55 (IQR 46–60)<br>control:<br>median 57·5 (IQR 49–64)	NA	intervention:<br>median 26 (IQR 24–30)<br>control:<br>median 25 (IQR 23–29)

Note: M: male; F: female; SD: standard deviation; BMI: body mass index; NA: not available; IQR: interquartile range

S7b. Summary of 19 studies concerning the impacts of preoperative smoking cessation time, NRT, and vaping (part 2)

No	First author/ Publication year	Weight (kg)	Height (cm)	Diabetes	Use of NSAIDs	Use of fluoro-quinolone family of antibiotics	Inclusion and exclusion criteria	Surgery type
1	Glassman 2000	NA	NA	NA	NA	NA	Inclusion criteria:<br>1. degenerative collapse with spinal stenosis, pseudarthrosis, mechanical low back pain, discogenic low back pain, or spinal instability. Instability was subclassified as spondylolisthesis, postdiscectomy instability, or mechanical instability based on flexion–extension radiographs.<br>2. patients who had undergone posterior instrumented fusion at either L4–L5 or L4–S1 from 1992 through 1996<br>3. All fusion procedures were performed using similar pedicle screw and rod constructs and autologous iliac crest bone graft	posterior instrumented fusion at either L4–L5 or L4–S1
2	Sørensen 2007	NA	NA	8 (3·76%)	NA	NA	daily smokers scheduled for elective open incisional or inguinal day-case herniotomy were included in the study	elective open incisional or inguinal day-case herniotomy
3	Delaunay 2018	NA	NA	21 (9·5	NA	NA	Inclusion criteria:	Abdominoplasty with umbilical

				0%)			patients who underwent an abdominoplasty with umbilical transposition and lipoaspiration of flancs and epigastriu m, with the handle bar incision, undermining as far as t he xyphoid appendix and the upper tension technique	transposition and lipoaspiration of flancs and epigastrium
4	Padubidri 2001	NA	NA	NA	NA	NA	NA	Breast reconstruction
5	Moller 2002	NA	NA	4 (3·7 0%)	NA	NA	Inclusion criteria:<br>Patients scheduled for primary elective hip or knee allopl asty at three university-affiliated hospitals in Copenhagen were invited to enter the study. All daily smokers were eligible for the study.<br>Exclusion criteria:<br>patients with a weekly alcohol intake greater than 35 uni ts	Elective hip or knee replacemen t
6	Araco 2008	72±8·5	167±13	NA	NA	NA	Inclusion criteria:<br>patients who underwent abdominoplasty for cosmetic purp oses<br>Exclusion criteria:<br>1. patients within 1 year after bariatric surgery<br>2. those with ongoing clinical infections<br>3. that received a complete antibiotic course in the previ ous 6 months<br>4. those under steroid therapy<br>5. those with systemic diseases that could impair tissue oxygenation or wound repair (e.g., arteriosclerosis, diabet es mellitus).	Abdominoplasty for cosmetic pu rposes
7	Chan 2006	NA	NA	NA	NA	NA	Inclusion criteria:<br>Patients who underwentbilateral breast reduction surgery<br>Exclusion criteria:<br>Four patients were excluded due to small volume reducti ons (<400 g in total).	Bilateral breast reduction surger y
8	Gravante 2007	133±16	156±7	NA	NA	NA	Inclusion criteria:<br>Patients who presented to the hospital requesting an abdo minoplasty after massive weight loss obtained after lapar oscopic adjustable gastric banding<br>Exclusion criteria:<br>1. patients with ongoing clinical infections<br>2. patients received a complete course of antibiotic for a n infection<br>in the last 6 months prior to operation<br>3. patients requesting steroid therapy<br>4. patients with systemic diseases that could impair woun d repair (arteriosclerosis, diabetes mellitus)<br>5. those <1 year since their bariatric surgery<br>6. cigar and pipe smokers	abdominoplasty after massive w eight loss obtained after laparos copic adjustable gastric banding

9	Gravante 2008	76±10 (59 to 93)	163±6 (147 to 180)	NA	NA	NA	Inclusion criteria:<br>patients who had undergone breast reductions<br>Exclusion criteria:<br>1. Patients who had previously undergone bariatric surgery<br>2. Patients who had ongoing clinical infections or had received a complete antibiotic course in the 6 months preceding the operation<br>3. Patients receiving steroid therapy<br>4. Patients with systemic diseases that could impair tissue oxygenation or wound repair (arteriosclerosis, diabetes mellitus)	breast reductions
10	Herrero 2020	NA	NA	NA	NA	NA	Inclusion criteria:<br>smokers greater than or equal to 18-years-old undergoing primary, elective total joint arthroplasty (TJA)<br>Exclusion criteria:<br>1. Non-smokers<br>2. Patients who were less than 18-years-old<br>3. Patients who underwent nonelective TJA.	total joint arthroplasty
11	Jung 2014	NA	NA	NA	NA	NA	Inclusion criteria:<br>Patients who were diagnosed with primary gastric adenocarcinoma and underwent radical gastrectomy	radical gastrectomy
12	Kuri 2005	NA	NA	NA	NA	NA	Inclusion criteria:<br>Patients who underwent removal of nasal, oral, pharyngeal, laryngeal,<br>or cervical esophageal cancer and reconstruction requiring a free flap with a vascular pedicle	removal of nasal, oral, pharyngeal, laryngeal,<br>or cervical esophageal cancer and reconstruction requiring a free flap with a vascular pedicle
13	Quan 2019	NA	NA	NA	NA	NA	Inclusion criteria:<br>Adult (≥18 years) patients with gastric cancer (GC) who underwent operation<br>Exclusion criteria:<br>1. Non-resection surgery<br>2. Palliative gastrectomy<br>3. Emergency surgery<br>4. Remnants of gastric cancer<br>5. Other synchronous malignancies<br>6. Stage IV operated with curable intent<br>7. Taking corticosteroid drugs for long term<br>8. Active infections disease<br>9. Missing clinical and/or laboratory data	Open procedure with D2 or D2 + lymphadenectomy.<br>Total laparoscopy or laparoscopy-assisted gastrectomy with D1 + α or β lymphadenectomy.<br>Combined multi-organ resection.
14	Sorensen 2003a	NA	NA	NA	NA	NA	Inclusion criteria:<br>Patients who smoked daily and were scheduled for an open colonic or rectal operative procedure with formation of an enteric anastomosis<br>Exclusion criteria:	1. Colonic resection<br>2. Other colonic procedure<br>3. Rectal resection<br>4. Explorative laparotomy<br>5. Mechanical bowel preparatio

							Patients with inflammatory bowel disease	n<br>6. Postoperative enteral feed tube
15	Thomsen 2010	NA	NA	4 (3·08%)	NA	NA	Inclusion criteria:<br>1. Patients had to be women<br>2. Patients who were daily smokers<br>3. Patients who aged 18 years and above<br>4. Patients who have sufficient language proficiency<br>Exclusion criteria:<br>patients with weekly alcohol intake exceeding 35 units, substance abuse, diagnosed psychiatric disease, dementia, American Society of Anesthesiologists' (ASA) physical status classification-class>3, penetrating cancer, pregnancy and lactation, and preoperative neoadjuvant chemotherapy.	breast cancer surgery
16	Yoshida 2015	NA	NA	28 (11·38%)	NA	NA	Inclusion criteria:<br>patients underwent elective esophagectomy with 2- or 3-field lymphadenectomy for esophageal cancer	elective esophagectomy with 2- or 3-field lymphadenectomy
17	Sorensen 2003b	NA	NA	NA	NA	NA	Exclusion criteria were chronic medical disease, atopic dermatitis or other skin disease, pregnancy, menopause, allergy to local anesthetics, dressing, or patches, and current or recent medication with corticosteroids, NSAID, or aspirin	5-mm full-thickness punch biopsy wound
18	Sorensen 2010	NA	NA	NA	0	NA	Inclusion criteria:<br>Not mentioned.<br>Exclusion criteria:<br>Chronic medical disease, pregnancy, menopause, and current or recent medication with corticosteroids or non-steroidal anti-inflammatory drugs (also known as NSAIDs).	5-mm, full-thickness punch biopsy on the skin over the medial gluteal muscle4–6 cm lateral to the sacrum
19	Lindström 2008	NA	NA	2 (1·96%)	NA	NA	Inclusion criteria:<br>1. patients had to be daily smokers (>2 cigarettes daily for at least 1 year before inclusion)<br>2. aged 18 to 79 years.<br>Exclusion criteria:<br>Patients with overt alcohol or drug abuse, pregnancy, severe mental illness, dementia, and poor Swedish language proficiency.	primary inguinal and umbilical hernia repair, laparoscopic cholecystectomy, or a hip or knee prosthesis

Note: NA: not available.

S7c. Summary of 19 studies concerning the impacts of preoperative smoking cessation time, NRT, and vaping (part 3)

No	First author/ Publication year	Groups	Cessation contents

1	Glassman 2000	1. Non-smokers (*n*=169)<br>2. None cessation (continuous smoking) before surgery (*n*=63)<br>3. Cessation <1 month before surgery (*n*=74)<br>4. Cessation >1 month before surgery (*n*=51)<br>5. None cessation (continuous smoking) after surgery (*n*=68)<br>6. Cessation 1–6 months after surgery (*n*=44)<br>7. Cessation >6 months after surgery (*n*=76)	same as left
2	Sørensen 2007	1. Standard advised group (*n*=48)<br>2. Advisded and reminder group (*n*=101)<br>3. Non-advised group (*n*=64)	1. Standard advised group were given the standard advice and were not contacted until the day of surgery.<br>2. Advisded and reminder group were given the standard advice and were reminded by the study nurse 1 month before surgery during a 10 min telephone conversation, or we re given the standard advice and were reminded 1 month before surgery during a 20 min meeting with a study nurse in the outpatient clinic. At the end of this meeting the patient was advised to use nicotine replacement drugs until 24 h before surgery and a sample of different types (Nicorette Patch (10 and 15 mg), Nicorette Chewing Gum (2 and 4 mg), Nicorette Resoriblet (2 mg), Nicorette Inhalator (10 mg), or Nicorette N asal Spray (500 µg per dose)) was offered for a few days use according to the patient s preference.<br>3. Non-advised group were not advised to quit smoking
3	Delaunay 2018	1. Nonsmokers (*n*=96)<br>2. Active smokers (*n*=71)<br>3. Former smokers (*n*=29)	Active smokers were asked to stop all smoking consumption 4 weeks before and after surgery
4	Padubidri 2001	1. Smokers (*n*=155)<br>2. Ex-smokers (*n*=76)<br>3. Nonsmokers (*n*=517)	Smokers were asked to quit smoking when they were first seen in the outpatients clini c. Elective surgery usually was scheduled after 3 weeks.<br>Patients who reported that they had quit smoking 3 weeks or more before surgery were classified as ex-smokers
5	Moller 2002	1. Intervention group (*n*=56)<br>2. Control group (daily smoker) (*n*=52)	The intervention period was 6–8 weeks before and 10 days after operation.<br>The patients in the intervention group were offered a<br>meeting every week with the project nurse. At the first<br>meeting a Fagerstöm test was done, to estimate the<br>magnitude and profile of nicotine dependence. A personalised nicotine substitution sched ule was devised in accordance with the test results and patient's preference. Patients w ere strongly encouraged to stop smoking completely, but they also had the option to re duce their tobacco consumption by at least 50%.<br>The controls received standard care, which meant little or no information about the risk of tobacco smoking or smoking cessation counselling.
6	Araco 2008	1. Smokers (*n*=42)<br>2. Nonsmokers (*n*=42)	Smokers were invited to stop smoking at least 4 weeks before surgery. Those that faile d had their surgery postponed until 4 weeks of cessation were obtained. Every
7	Chan 2006	1. Continuous smoking (*n*=31)<br>2. Stopped smoking <4 weeks (*n*=19)<br>3. Stopped smoking >4 weeks (*n*=15)<br>4. Nonsmokers (*n*=104)	All patients had been asked to stop smoking at least 1 month before surgery until the wounds had completely healed as a hospital policy.
8	Gravante 2007	1. Smokers (*n*=30)<br>2. Non-smokers (*n*=30)	Smokers were asked to stop at least for 4 weeks before surgery. Those who failed (aft er confirmation of relatives) had their operation postponed until 4 weeks of smoking ce ssation were obtained

9	Gravante 2008	1. Smokers (*n*=43)<br>2. Nonsmokers (*n*=44)	Patients were instructed to stop smoking at least 4 weeks before surgery. Those who fa iled to stop smoking had their surgery postponed until they had obtained 4 weeks of s moking cessation.
10	Herrero 2020	1. Quit smoking (*n*=80)<br>2. Smoking (*n*=120)	Smoking cessation program (SCP) interventions are initiated 4–6 weeks prior to surgery, with a goal of being tobacco free 1–2 weeks prior to surgery. The protocol consists o f four pre-operative telephone sessions including assessment, education, counseling, nicot ine replacement therapy (NRT), and two<br>follow-up sessions within 30 days after surgery.<br>Self smoking cessation was also performed in self-treatment group.
11	Jung 2014	1. Nonsmokers (*n*=813)<br>2. Smoking cessation ≥8 weeks (*n*=193)<br>3. Smoking cessation 4 to <8 weeks (*n*=82)<br>4. Smoking cessation 2 to <4 weeks (*n*=40)<br>5. Smoking cessation <2 weeks (*n*=207)	Smoking cessation period before the surgery (<2, 2 to <4, 4 to <8, and ≥8 weeks) bec ause the interval from the diagnosis of gastric cancer to the surgery in our hospital wa s approximately 2–8 weeks
12	Kuri 2005	1. Smokers (*n*=28)<br>2. Late quitters (*n*=34)<br>3. Intermediate quitters (*n*=20)<br>4. Early quitters (*n*=66)<br>5. Nonsmokers (*n*=40)	The late, intermediate, and early quitters were defined as patients whose smoke-free dur ation was between 8 and 21 days (*n*=34), between 22 and 42 days (*n*=20), and more t han 43 days (*n*=66) before the operation, respectively
13	Quan 2019	1. Non-smokers (*n*=1,413)<br>2. Smoking cessation ≤2 weeks (*n*=823)<br>3. Smoking cessation 2 to 4 weeks (*n*=40)<br>4. Smoking cessation ≥4 weeks (*n*=193)	All smokers were instructed to quit smoking immediately when they were admitted. Sm okers were divided into three categories depending on their smoking cessation period b efore surgery (≤ 2, 2 to 4, and ≥ 4 weeks).
14	Sorensen 2003a	1. Intervention group (*n*=27)<br>2. Control group (daily smoker) (*n*=30)	Patients randomised to the intervention group were asked to stop smoking the following day until the time of removal of sutures after surgery.<br>A pre-operative intervention period of 2–3 weeks was intended to obtain a sufficient s moking-free interval within a pre-operative waiting period.<br>patients in the intervention group were offered pre- and post-operative individual suppor t by the project nurse and nicotine replacement therapy until 24 h before surgery
15	Thomsen 2010	1. Intervention group (*n*=57)<br>2. Control group (daily smoker) (*n*=62)	Complete smoking cessation was recommended from 2 days before to 10 days after sur gery
16	Yoshida 2015	1. Continuous smokers (*n*=15)<br>2. Stop smoking 7 to 30 days (*n*=28)<br>3. Stop smoking 31 to 90 days (*n*=40)<br>4. Stop smoking ≥91 days (*n*=102)<br>5. Never smokers (*n*=61)	Cessation period included 7 to 30 days, 31 to 90 days, ≥91 days before surgery
17	Sorensen 2003b	From the second week, they were randomised to<br>1. continuous smokers (*n*=16)<br>2. smoking abstinence (*n*=32)<br>3. Never smokers (*n*=30)	During the first week, smokers smoked 20 filter cigarettes per day.<br>From the second week, they were randomised to continuous smoking (20 cigarettes per day), smoking abstinence with transdermal nicotine patch (15 mg/16 h plus 10 mg/16 h, Nicorette), or smoking abstinence with placebo patch. Each subgroup included 16 pat ients.<br>In total, each of 4 biopsy wounds were maded at 0, 4, 8, and 12 weeks after randomi sation to groups, respectively.

18	Sorensen 2010	1. Never smokers (*n*=30)<br>2. continuous smokers (*n*=16)<br>3. Abstinent smokers (*n*=32)	During the first week of the study, all smokers smoked 20 standardized filter cigarettes per day.<br>Subsequently, smokers were randomised double-blinded into 3 groups: continued to smo ke 20 filter cigarettes per day, refrained from smoking and used a transdermal nicotine patch (TNP) (15 mg/16 h plus 10 mg/16 h, Nicorette) 24 hours a day, and refrained from smoking and used placebo patches. The latter two subgroups belong to abstinent smokers group.
19	Lindström 2008	1. smoking cessation (*n*=48)<br>2. continuous smokers (*n*=54)	The intervention was intended to start 4 weeks before surgery and last 4 weeks after s urgery, as described in detail elsewhere. The intervention included weekly meetings or t elephone counseling with a nurse professionally trained in smoking cessation therapy, th e telephone number to a hot line providing smoking cessation advice, and free nicotine substitution (Nicorette) offered with an individual schedule for the entire intervention pe riod. No bupropion or varenicline was offered. Nicotine replacement therapy was the on ly drug offered.

S7d. Summary of 19 studies concerning the impacts of preoperative smoking cessation time, NRT, and vaping (part 4)

No	First author/ Publication year	Definition of outcomes	Follow-up mean±SD, range	Description of results and reasons for not including in meta-analysis
1	Glassman 2000	1. nonunion was defined strictly based on surgical exploration, hardware failure, comp uted tomographic (CT) scan with three-dim ensional reconstruction, or tomograms. Patie nts without obvious nonunion based on the se criteria were assumed to have adequate fusion.	49·4 months (> 2 ye ars)	1. Nonsmokers vs. continuous smoking before surgery vs. stopping <1 month before surg ery vs. stopping >1 month before surgery: nonunion rate<br>For nonunion rate, no significant difference was found between nonsmoker (14·2% [24/169]), continuous smoking (22·2% [14/63]), stopping <1 month (20·2% [15/74]), and stopping >1 month (19·6% [10/51]) (*P*>0·05).<br>2. Nonsmokers vs. continuous smoking after surgery vs. stopping 1–6 months after surger y vs. stopping >6 months after surgery: nonunion rate<br>Nonunion rate was increased in continuous smokers, compared with nonsmokers (1 4·20% [24/169] vs· 26·47% [18/68], *P*=**0·025**). However, no significant difference was fo und between other groups (*P*>0·05).
2	Sørensen 2007	1. Postoperative wound infection was deWn ed as a swollen, red, hot, painful wound w ith or without pus discharge and postoperat ive clinical intervention including antibiotic s, extensive wound care, or re-operation.	192 days	Standard advised vs. advised and reminder (1 month before surgery) vs. non-advised (con tinued smokers): postoperative wound infection<br>No significant difference was found between the advised (7% [10/149]) and non-advised (5% [3/64]) cohorts (*P*=0·57) or between the standard (8% [4/48]) and the reminder (6% [6/101]) group (*P*=0·59).
3	Delaunay 2018	1. Delayed healing (wound dehiscence, epi dermolysis, partial or full thickness woun d). A delayed healing was decided after 3 weeks' delay.	3 months	Nonsmokers vs. active smokers (asked to stop 4 weeks before surgery) vs. former smoke rs: delayed wound healing rate<br>nonsmoker vs active smoker cessation *P*=**0·02**<br>active smoker cessation vs former smoker *P*=0·92<br>nonsmoker vs former smoker *P*=0·05<br>However, multivarious analysis showed that difference of delayed healing rate between ac tive smokers who stopped 4 weeks before surgery (1·84[0·89 to 3·78]), former smoker (2·42[0·93 to 6·35]) and nonsmokers were not significant (*P*=0·11)·

4	Padubidri 2001	NA	NA (length of stay in hospital was between 1 and 25 days)	Nonsmokers vs. smokers (asked to stop 3 weeks before surgery) vs. ex-smokers (stopped ≥3 weeks): seroma, hematoma, wound infection, wound dehiscence rate<br>For seroma (smokers 5·84% [9/154] vs. ex-smokers 3·95% [3/76] vs. nonsmokers 4·09% [21/514]), hematoma (smokers 1·30% [2/154] vs. ex-smokers 0% [0/76] vs. nonsmokers 2·14% [11/514]), wound infection (smokers 5·19 [8/154] vs. ex-smokers 6·58% [5/76] vs. nonsmokers 4·47% [23/514]), and wound dehiscence rate (smokers 1·95% [3/154] vs. ex-smokers 0% [0/76] vs. nonsmokers 0·78% [4/514]), there was no significant difference between smokers, ex-smokers and nonsmokers (*P*>0·05).
5	Moller 2002	NA	12 weeks	Intervention group (stopped 6–8 weeks before surgery) vs. control group (continuous smoking): hematoma, wound infection<br>Hematoma:<br>There was no significant difference between intervention group (1·79% [1/56]) and control group (7·69% [4/52]) (*P*=0·19)<br>Infection:<br>Infection rate was significantly decreased in the intervention group (3·57% [2/56]) compared with control group (23·08% [12/52]) (*P*=**0·003**).
6	Araco 2008	Postoperative infections were defined on the basis of clinical signs (e.g., cellulitis, pain, swelling, drainage, elevation of white blood cells, fever), exudate cultures, superficial infections such as those involving the skin and subcutaneous tissues, and deep infections including muscle and fascia.	6 months	Smokers (invited to stop smoking ≥4 weeks before surgery) vs. nonsmokers: infection rate<br>Infection rate of smokers (1·2% [1/42]) significantly increased compared with nonsmokers (14·3% [12/42]) (*P*<**0·001**).
7	Chan 2006	Wound healing problems were divided into dehiscence, fat necrosis, infection, and the need for invasive interventions that included wound irrigation, debridement, or secondary procedures. A dehiscence was recorded when a skin gap of more than 1cm^2 was present, whereas an infection was defined when a wound culture was positive or when antibiotic therapy was commenced. It was also noted if there<br>were immediate postsurgical problems. Isolated hematoma and seroma were not considered to be wound-healing problems and excluded.	NA	Continuous smoking vs. stop smoking <4 weeks vs. stop smoking >4 weeks vs. nonsmokers: wound healing problem<br>Wound-healing problem:<br>1. Still smoking vs. stopped <4 weeks: 67·74% (21/31) vs. 52·63% (10/19), *P*=0·29<br>2. Still smoking vs. stopped >4 weeks: 67·74% (21/31) vs. 33·33% (5/15), *P*=**0·03**<br>3. Still smoking vs. nonsmoker: 67·74% (21/31) vs. 33·65% (35/104), *P*=**0·001**<br>4. Stopped <4 weeks vs. stopped >4 weeks: 52·63% (10/19) vs. 33·33% (5/15), *P*=0·26<br>5. Stopped <4 weeks vs. nonsmoker: 52·63% (10/19) vs. 33·65% (35/104), *P*=0·11<br>6. Stopped >4 weeks vs. nonsmoker: 33·33% (5/15) vs. 33·65% (35/104), *P*=0·98<br>Compared with continuous smoking group, wound-healing problem rate was obviously increased in stopped smoking>4 weeks group (*P*=**0·03**) and nonsmoker group (*P*=**0·001**). However, no significant difference was found in other comparisons (*P*>0·05).
8	Gravante 2007	Postoperative infections were defined on the basis of clinical signs (cellulitis, pain, swelling, drainage, wound dehiscence, elevation of white blood cells, fever) and microbiological cultures.	NA	Smokers (asked to stop smoking ≥4 weeks before surgery) vs. non-smokers: infection rate<br>Infection rate of smokers (46·7% [14/30]) was larger than non-smokers (3·3% [1/30]) (*P*=**0·0001**)
9	Gravante 2008	Postoperative infections were defined on the basis of clinical signs (cellulitis, pain, swelling, drainage, wound dehiscence, elevatio	6 months	Smokers (instructed to stop smoking ≥4 weeks before surgery) vs. nonsmokers: infection rate<br>Total infection rate of smokers (37·2% [16/43]) was increased compared with nonsmokers

		n of white blood cells, fever) and exudate cultures (in cases of wounds dehiscence or drainage).		(18·2% [8/44]) (*P*<**0·05**).<br>However, for superficial infection rate, there was no significant difference between smokers (27·9% [12/43]) and nonsmokers (15·9% [7/44]) (*P*>0·05).<br>For deep infection rate, difference between smokers (5·7% [4/43]) and nonsmokers (9·3% [1/44]) was also not significant (*P*>0·05)
10	Herrero 2020	NA	30 days	Quit smoking (4-6 weeks before surgery) vs. smoking: infection rate<br>Patients who quit smoking were less likely to experience infection (3·7% [2/80] vs 12·5% [8/120], *P*= **0·03**), than smokers.
11	Jung 2014	Surgical site infections were defined as infections that occur at the surgical site within 30 days after operation, which were categorized into three types (superficial, deep, and organspace) according to the US Centers for Disease Control and Prevention.	median 39 (0 to 46) months	Nonsmokers vs. stop smoking ≥8 weeks before surgery vs. stop smoking 4 to <8 weeks before surgery vs. stop smoking 2 to <4 weeks before surgery vs. stop smoking <2 weeks before surgery: wound complication<br>Wound complication (surgical site infection and wound dehiscence):<br>1. Nonsmoker (1·0% [8/813]) vs. smoking cessation ≥8 weeks (2·6% [5/193]), *P*=0·08<br>2. Nonsmoker vs. smoking cessation 4 to <8 weeks (4·9% [4/82]), *P*=**0·003**<br>3. Nonsmoker vs. smoking cessation 2 to <4 weeks (2·5% [1/40]), *P*=0·36<br>4. Nonsmoker vs. smoking cessation <2 weeks (5·3% [11/207]), *P*<**0·001**<br>5. Smoking cessation ≥8 weeks vs. 4 to <8 weeks, *P*=0·33<br>6. Smoking cessation ≥8 weeks vs. 2 to <4 weeks, *P*=1·000<br>7. Smoking cessation ≥8 weeks vs. <2 weeks, *P*=0·17<br>8. Smoking cessation 4 to <8 weeks vs. 2 to <4 weeks, *P*=1·000<br>9. Smoking cessation 4 to <8 weeks vs. <2 weeks, *P*=0·88<br>10. Smoking cessation 2 to <4 weeks vs. <2 weeks, *P*=0·45<br>The study showed that wound complication rate was significantly increased in smoking cessation 4 to <8 weeks and smoking cessation <2 weeks, compared with nonsmokers.
12	Kuri 2005	NA	NA	Smokers vs. late quitters (between 8 and 21 days before surgery) vs. intermediate quitters (between 22 and 42 days before surgery) vs. early quitters (>43 days before surgery) vs. nonsmokers: impaired wound healing<br>Impaired wound healing:<br>1. Smokers (85·7% [24/28]) vs. late quitters (67·6% [23/34]), *P*=0·10<br>2. Smokers vs. intermediate quitters (55·0% [11/20]), *P*=**0·02**<br>3. Smokers vs. early quitters (59·1% [39/66]), *P*=0·01<br>4. Smokers vs. nonsmokers (47·5% [19/40]), *P*=**0·001**<br>5. Late quitters vs. intermediate quitters, *P*=0·35<br>6. Late quitters vs. early quitters, *P*=0·40<br>7. Late quitters vs. nonsmokers, *P*=0·08<br>8. Intermediate quitters vs. early quitters, *P*=0·75<br>9. Intermediate quitters vs. nonsmokers, *P*=0·58<br>10. Early quitters vs. nonsmokers, *P*=0·25<br>Compared with smokers, impaired wound healing rate was obviously decreased in the intermediate quitters, early quitters, and nonsmokers.
13	Quan 2019	NA	30 days	Nonsmokers vs. stop smoking ≤2 weeks before surgery vs. stop smoking 2 to 4 weeks before surgery vs. stop smoking ≥4 weeks before surgery: infection<br>Surgical site infection:<br>1. Nonsmokers vs. smoking cessation ≤2 weeks, *P*=0·25<br>2. Nonsmokers vs. smoking cessation 2 to 4 weeks, *P*=0·36

				3. Nonsmokers vs. smoking cessation ≥4 weeks, *P*=0·05<br>4. Smoking cessation ≤2 weeks vs. smoking cessation 2 to 4 weeks, *P*=0·27<br>5. Smoking cessation ≤2 weeks vs. smoking cessation ≥4 weeks, *P*=0·27<br>6. Smoking cessation 2 to 4 weeks vs. smoking cessation ≥4 weeks, *P*=0·16<br>For surgical site infection rate, there was no significant difference was found between ea ch group.
14	Sorensen 2003a	NA	30 days	Intervention group (asked to stop smoking 2–3 weeks before surgery) vs. control group (continuous smokers): infection rate<br>Wound infection rate:<br>There was no significant difference between intervention group (11% [3/27]) and control group (13% [4/30]) (*P*=1·00).
15	Thomsen 2010	NA	12 months	Intervention group (recommended from 2 days before to 10 days after surgery) vs. contro l group (continuous smokers): infection, any wound complication<br>Wound infection:<br>There was no significant difference between intervention group (14% [8/57]) and control group (13% [8/62]) (*P*=0·86).<br>Any wound complication:<br>There was also no significant difference between intervention group (44% [25/57]) and c ontrol group (45% [28/62]) (*P*=1·00)
16	Yoshida 2015	Surgical site infection (SSI) was defined as an infection that occurred within 30 days after surgery in the area of the body affect ed by surgery and included superficial incis ional, deep incisional, and organ/ space SSI	NA	No cessation (continuous smokers) vs. stop smoking 7 to 30 days before surgery vs. stop smoking 31 to 90 days before surgery vs. stop smoking ≥91 days before surgery vs. ne ver smokers: infection<br>For surgical site infection rate, there was no significant difference between continuous sm okers group (33·3% [5/15]), stop smoking 7 to 30 days (32·1% [9/28]), stop smoking 31 to 90 days (22·5% [9/40]), stop smoking ≥91 days (21·6% [22/102]), and never smoker s (27·9% [17/61]) (*P*>0·05).
17	Sorensen 2003b	1. A wound infection was defined accordin g to the National Prevalence Survey study group as purulent discharge with or withou t wound dehiscence or painful, hot extendi ng erythema indicative of cellulitis<br>2. A wound rupture was defined as a com plete separation of wound edges without si gns of infection.	14 weeks<br>(each wound was fol lowed up for 2 week s)	Continuous smokers vs. smoking abstinence (stopped 0, 4, 8, 12 weeks before surgery) v s. never smokers: wound infection rate and wound rupture rate<br>Wound infection:<br>After 4 weeks of randomisation, wound infections were significantly fewer in the abstine nt smoker group (3·45% [1/29]) compared with the group of continuous smokers (25% [4/16]), *P*=0·047). Similar differences were found after 8 weeks (current smokers vs. abst inent smokers: 20% [3/15] vs. 0% [0/29], *P*=0·03) and 12 weeks (current smokers vs. ab stinent smokers: 16·67% [1/6] vs. 0% [0/29], *P*=0·03) of randomisation.<br>No significant difference was found in the rest comparisons.<br>Wound rupture:<br>At 4 and 12 weeks, no rupture occurred in the three groups.<br>At 8 weeks, no significant difference was found in nonsmoker (0% [0/6], *P*=1·00) or ex-smoker (6·90% [2/29], *P*=1·00), compared with continuous smoker (6·67% [1/15]).

18	Sorensen 2010	NA	13 weeks<br>(each wound was followed up for 1 week)	Never smokers vs. continuous smokers vs. abstinent smokers (0, 4, 8, 12 weeks before surgery): wound diameter, wound depth<br>The smokers' wounds including continuous and abstinent smokers were smaller and more superficial than the wounds of the never smokers ($P<0{\cdot}01$). After smoking cessation, the abstinent smokers' wound diameter remained unchanged compared with the smoker's wounds ($P=0{\cdot}17$).<br>The wound depth was not affected after the first 4 weeks of abstinence, but thereafter, the abstinent smoker's wound depth increased ($P<0{\cdot}01$). No significant difference in wound<br>depth was found between the abstinent smokers and the never smokers. Also, no significant difference was found between transdermal nicotine patch (TNP) and the placebo group (data not described).
19	Lindström 2008	Wound complications Seroma (wound revision, wound drainage or need for repeated wound dressings)<br>Superficial wound infection (infection treated with antibiotics or repeated wound dressings)<br>Deep wound infection (treated with surgical debridement)<br>Hematoma (treated with surgical intervention, blood transfusion, or extra wound checks)<br>Wound dehiscence (redo surgery)<br>Skin necrosis (surgical wound revision or repeated wound checks)<br>Pressure wounds (wound revision or need for repeated wound dressings)	30 days	Smoking cessation 4 weeks before surgery vs. continuous smokers<br>hematoma:<br>smoking cessation 4 weeks (6% [3/48] vs. 13% [7/54], $P=0{\cdot}33$)<br>Wound infection:<br>Smoking cessation 4 weeks (4% [2/48] vs. 7% [4/54], $P=0{\cdot}68$)<br>Seroma:<br>Smoking cessation 4 weeks (6% [3/48] vs. 9% [5/54], $P=0{\cdot}72$)

Note: NA: not available.

Appendix S8. Risk of bias for 58 studies included in the meta-analyses of the impacts of smoking and alcohol consumption

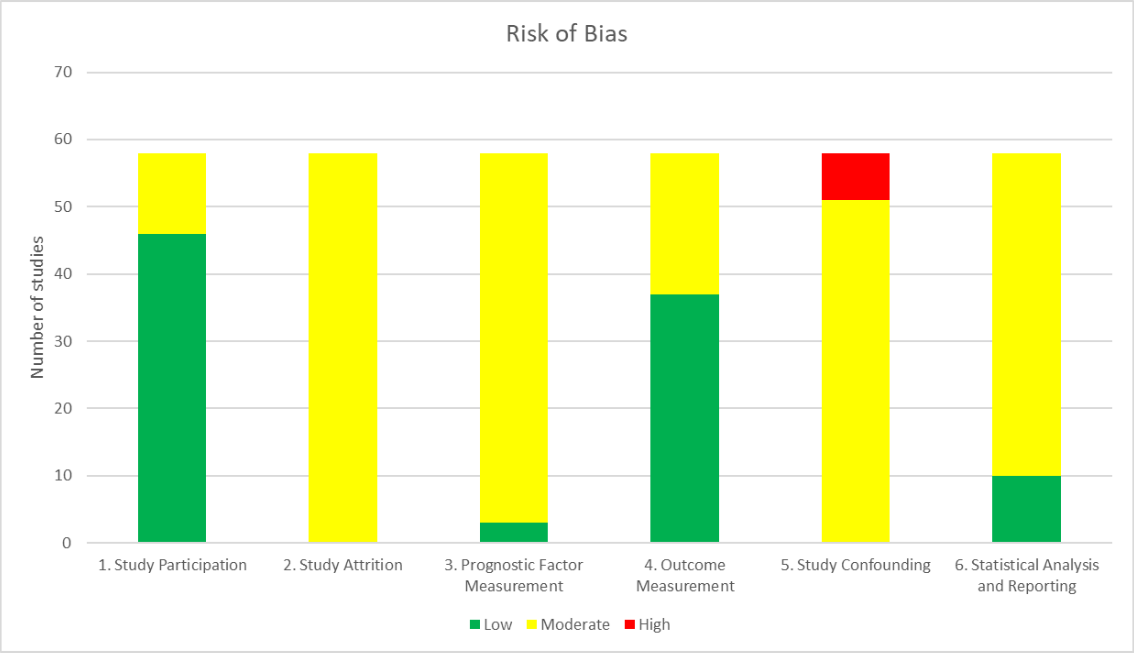

(1) In the study participation domain, 46 studies (79%) exhibited a low risk of bias, and the remaining 12 studies exhibited a moderate risk of bias (21%).

(2) All of the 58 (100%) studies were graded as having a moderate risk of bias in the study attrition domain.

(3) All but three of the low-risk-of-bias studies exhibited a moderate risk of bias in the prognostic fact or measurement domain.

(4) In the outcome measurement domain, 37 (64%) studies exhibited a low risk of bias, and 21 (36%) of the studies exhibited a moderate risk of bias.

(5) All except seven high-risk studies exhibited a moderate risk of bias in the study confounding doma in.

(6) In the statistical analysis and reporting domain, 10 studies were rated as having a low risk of bias, and 48 studies (83%) were rated as having a moderate risk of bias.

Appendix S9. List of studies about the impacts of smoking and alcohol consumpti on on fracture healing and surgical site infection

Comparison	Outcomes	No. of studies with sample size	No. of studies withou t sample size
Smokers vs. non-smokers	delayed union	3[1,4,20]	-
	nonunion	18[1,3-5,11,19,20,22-27,29,32,33,37,54]	-
	time to union	2[1,51]	-
	superficial surgical site infection	4[4,29,42,47]	-
	deep surgical site infection	17[4,9,10,19,21,24,29-31,34,37,42,47-49,52,53]	-
	undefined surgical site infection	18[2,12-15,17,18,35,38-41,43-46,50,57]	1[7]
	normal union	5[1,4,20,28,36]	-
alcohol drinkers vs. non-drinkers	nonunion	3[3,5,11]	-
	time to union	1[1]	-
	deep surgical site infection	2[6,8]	-
	undefined surgical site infection	8[2,12,15,16,50,55-57]	1[7]

Appendix S10. Data of the studies comparing nonunion between smokers and non -smokers

No	Study	nonunion in smokers	total smokers	nonunion in non-smokers	total non-smokers	Time point for diagnosis of nonunion after injury/surgery
1	Askew 2011	2	21	2	9	6 months after surgery
2	Blair 2016	7	48	9	136	9 months
3	Bone 1997	3	18	8	29	6 months
4	Chu 2018	OR 3.90, 95%CI (0.61–25.21)	24	NA	96	6 months after surgery
5	Ding 2014	11	165	13	494	9 months after injury
6	Donohue 2016	17	109	17	219	NA
7	Enninghorst 2011	15	23	39	66	6 months
8	Haller 2019	22	118	50	400	9 months
9	Kent 2015	9	12	21	28	6 months
10	Kim 2018	4	5	17	68	6 months after surgery
11	Kuklo 2008	6	14	15	115	12 months after surgery
12	Liu 2015	30	155	66	649	6 months after injury
13	Moghaddam 2011	9	46	0	39	6 months
14	Murray 2013	73	219	52	722	6 months after injury
15	Ozbek 2017	14	19	24	56	12 months after surgery
16	Subramanyam 2021	3	4	3	96	6 months after injury
17	Thorud 2017	25	55	21	85	6 months after injury
18	Zumsteg 2014	12	63	17	137	6 months
19	Olson 2020b	OR 1.9, 95%CI (0.7–5.0)	75	NA	89	6 months

Note: OR: odds ratio; NA: not available.

Appendix S11. Forest plot of meta-analysis of nonunion rate of alcohol drinkers vs. non-drinkers

	Alcohol drinkers		Non-drinkers			Odds Ratio
Study or Subgroup	Events	Total	Events	Total	Weight	M-H, Random, 95% CI
Ding 2014	5	206	19	453	28.4%	0.57 [0.21, 1.54]
Liu 2015	21	233	75	571	38.5%	0.66 [0.39, 1.09]
Thorud 2017	18	37	30	107	33.2%	2.43 [1.13, 5.25]
**Total (95% CI)**		**476**		**1131**	**100.0%**	**0.97 [0.40, 2.38]**
Total events	44		124			

Heterogeneity: $Tau^2 = 0.47$; $Chi^2 = 8.71$, df = 2 (P = 0.01); $I^2 = 77\%$

Test for overall effect: Z = 0.06 (P = 0.95)

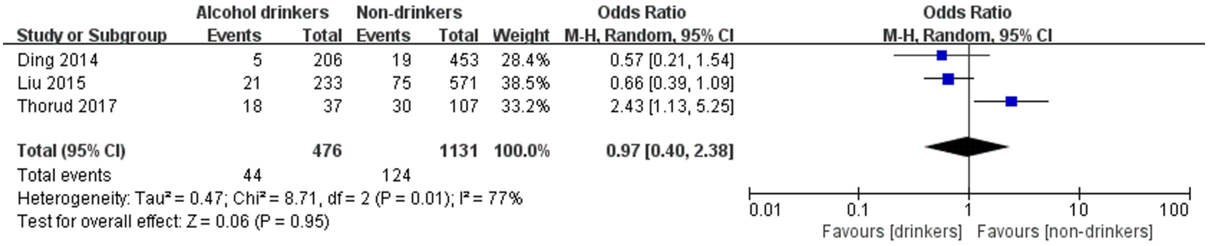

Appendix S12. Forest plots of the meta-analyses of secondary outcomes of smoker s vs. non-smokers

Forest plots of the meta-analyses of S12a) undefined surgical site infection rate and S12b) normal unio n rate of smokers vs. non-smokers

Study or Subgroup	log[Odds Ratio]	SE	Weight	Odds Ratio IV, Random, 95% CI
Alzahrani 2018	0.393	1.1838	2.2%	1.48 [0.15, 15.08]
Assous 2001	2.5601	0.8091	3.7%	12.94 [2.65, 63.17]
Bai 2019	0.9569	0.425	6.4%	2.60 [1.13, 5.99]
Basques 2015	1.0986	0.4265	6.4%	3.00 [1.30, 6.92]
Bowen 2005	1.1676	0.5001	5.8%	3.21 [1.21, 8.57]
Chan 2019	-0.6733	0.8313	3.6%	0.51 [0.10, 2.60]
Claessen 2016	0.3681	0.3128	7.4%	1.44 [0.78, 2.67]
Flynn 2000	0.723	0.5844	5.2%	2.06 [0.66, 6.48]
Hoffmann 2013	2.817	1.0816	2.5%	16.73 [2.01, 139.34]
Korim 2014	3.2411	0.5236	5.6%	25.56 [9.16, 71.33]
Li 2017	2.0761	0.4667	6.1%	7.97 [3.19, 19.90]
Li 2020	1.035	0.4734	6.0%	2.82 [1.11, 7.12]
Lin 2013	-0.0457	0.4752	6.0%	0.96 [0.38, 2.42]
Mckissack 2019	0.392	0.2595	7.8%	1.48 [0.89, 2.46]
Minhas 2019	1.0986	0.3537	7.1%	3.00 [1.50, 6.00]
Ren 2015	1.2238	0.6266	4.8%	3.40 [1.00, 11.61]
Singh 2018	1.752	0.4897	5.9%	5.77 [2.21, 15.06]
Spross 2012	-0.4394	1.6938	1.2%	0.64 [0.02, 17.82]
Zhu 2021	1.4065	0.4728	6.0%	4.08 [1.62, 10.31]
**Total (95% CI)**			**100.0%**	**3.11 [2.09, 4.63]**

Heterogeneity: Tau² = 0.46; Chi² = 53.33, df = 18 (P < 0.0001); I² = 66%
Test for overall effect: Z = 5.59 (P < 0.00001)

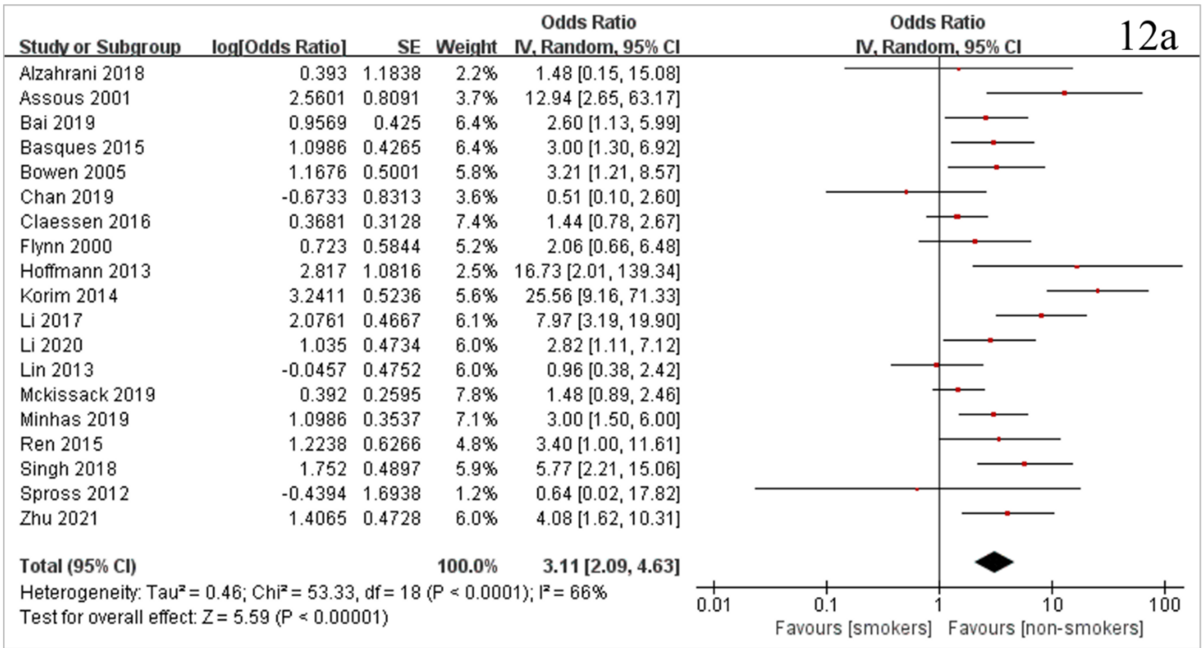

Study or Subgroup	Smokers Events	Smokers Total	Non-smokers Events	Non-smokers Total	Weight	Odds Ratio M-H, Random, 95% CI
Askew 2011	11	21	5	9	20.7%	0.88 [0.18, 4.23]
Bone 1997	1	18	1	29	6.3%	1.65 [0.10, 28.09]
Kent 2015	2	12	2	28	11.6%	2.60 [0.32, 21.05]
McDonald 2018	69	73	192	208	39.8%	1.44 [0.46, 4.45]
Snall 2015	19	23	9	14	21.6%	2.64 [0.57, 12.25]
**Total (95% CI)**		**147**		**288**	**100.0%**	**1.60 [0.78, 3.26]**
Total events	102		209			

Heterogeneity: Tau² = 0.00; Chi² = 1.21, df = 4 (P = 0.88); I² = 0%
Test for overall effect: Z = 1.29 (P = 0.20)

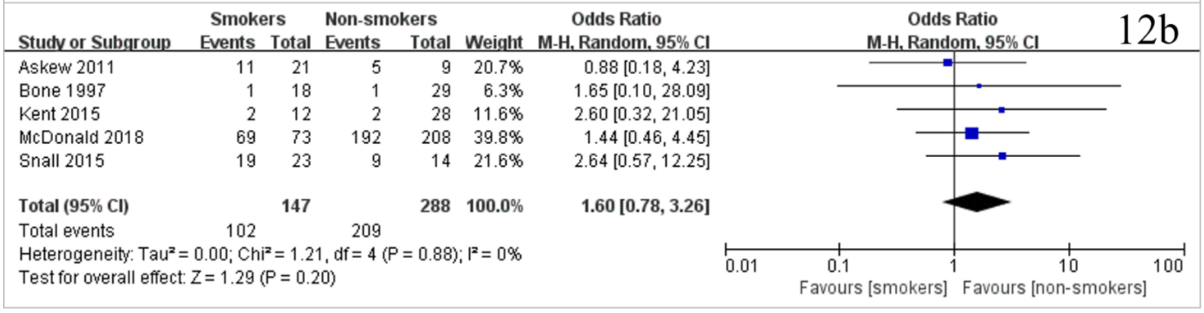

Appendix S13. Forest plots of the meta-analyses of secondary outcomes of alcohol drinkers vs. non-drinkers

Forest plots of meta-analyses of S13a) deep surgical site infection rate and S13b) undefined surgical sit e infection rate of alcohol drinkers vs. non-drinkers

13a

Study or Subgroup	Alcohol drinkers Events	Alcohol drinkers Total	Non-drinkers Events	Non-drinkers Total	Weight	Odds Ratio M-H, Random, 95% CI
Ma 2017	7	281	10	395	22.6%	0.98 [0.37, 2.62]
Meng 2018	19	658	55	1959	77.4%	1.03 [0.61, 1.75]
**Total (95% CI)**		**939**		**2354**	**100.0%**	**1.02 [0.64, 1.62]**
Total events	26		65			

Heterogeneity: Tau² = 0.00; Chi² = 0.01, df = 1 (P = 0.94); I² = 0%
Test for overall effect: Z = 0.08 (P = 0.94)

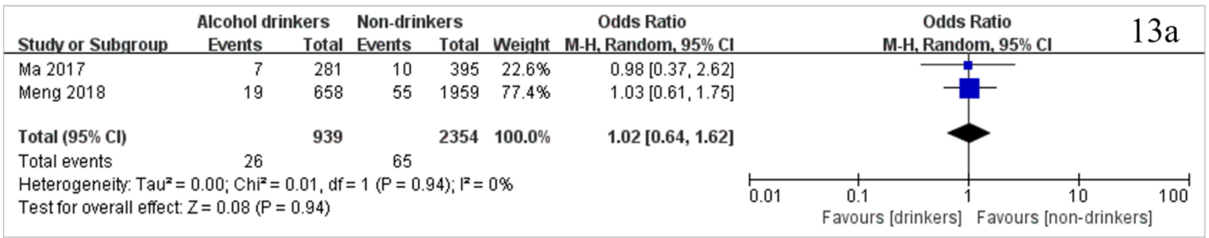

13b

Study or Subgroup	log[Odds Ratio]	SE	Weight	Odds Ratio IV, Random, 95% CI
Bai 2019	0.2671	0.4176	12.0%	1.31 [0.58, 2.96]
Bowen 2005	0.6983	0.5119	10.1%	2.01 [0.74, 5.48]
Flynn 2000	0.1446	0.5827	8.8%	1.16 [0.37, 3.62]
Li 2020	0.2523	0.5117	10.1%	1.29 [0.47, 3.51]
Mardanpour 2020	-0.9416	0.4546	11.2%	0.39 [0.16, 0.95]
Mckissack 2019	-0.4005	0.2761	15.2%	0.67 [0.39, 1.15]
Sun 2017	0.3511	0.3435	13.7%	1.42 [0.72, 2.79]
Tan 2021	0.8561	0.6489	7.8%	2.35 [0.66, 8.40]
Zhu 2021	1.4206	0.4603	11.1%	4.14 [1.68, 10.20]
**Total (95% CI)**			**100.0%**	**1.28 [0.81, 2.03]**

Heterogeneity: Tau² = 0.29; Chi² = 20.15, df = 8 (P = 0.010); I² = 60%
Test for overall effect: Z = 1.05 (P = 0.29)

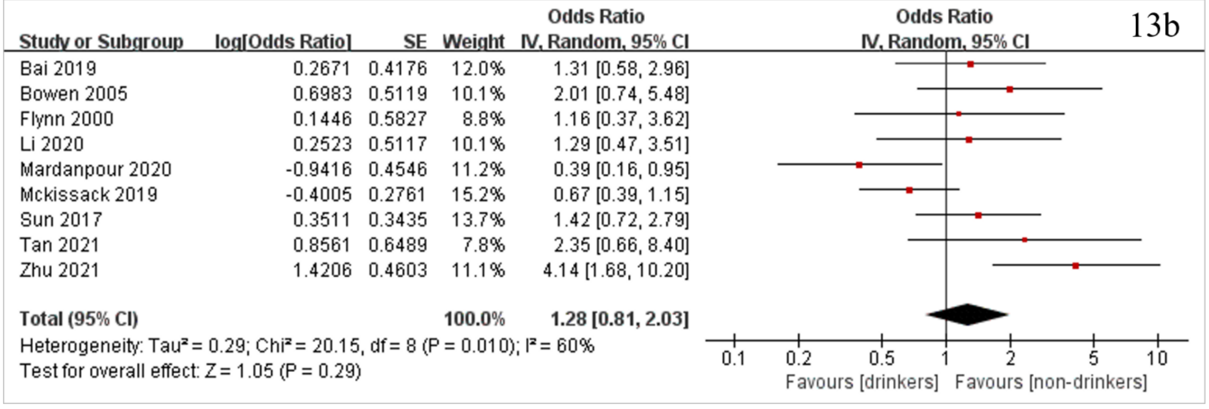

Appendix S14. List of the studies and the results of the impacts of preoperative smoking cessation time, NRT, and vaping

S14a. List of the studies and the results about the impacts of smoking cessation time on bone healing, wound healing, and wound complications

Outcomes	Preoperative cessation time	Smoking cessation vs. continuous smokers	Smoking cessation vs. non smokers
Bone healing	<1 month,<br>>1 month	1 study[104]	
	1 to 6 months,<br>>6 months post-operatively		
Impaired wound healing	>3 weeks	-	2 studies[106,115]
	≥4 weeks	1 study[115]	2 studies[106,115]
Wound infection	2 days before to 10 days after surgery	1 study[118]	-
	2 to 3 weeks	1 study[117]	-
	≥3 weeks	-	7 studies[107,109,111,112,116,119,120]
	≥4 weeks	6 studies[105,108,113,119,120,122]	6 studies[109,111,112,116,119,120]
	≥6 weeks	3 studies[108,119,120]	-
	≥8 weeks	2 studies[119,120]	
	≥12 weeks		
Wound hematoma	3 weeks	-	1 study[107]
	4 weeks	1 study[122]	
	6 to 8 weeks	1 study[108]	
Dehiscence	3 weeks	-	1 study[107]
Seroma	3 weeks	-	1 study[107]
	4 weeks	1 study[122]	-
Wound infection plus dehiscence	<2 weeks<br>2 to <4 weeks<br>4 to <8 weeks<br>≥8 weeks	-	1 study[114]
Wound healing problems (dehiscence, fat necrosis, infection)	<4 weeks<br>>4 weeks	-	1 study[110]

One study including 357 patients showed that compared with continued smokers (22·2%) or non-smokers (14·2%), the cessation of smoking at less than one month (20·2%) or more than one month (19·6%) before surgery, did not significantly change the bone nonunion rate after spinal fusion surgery.[104] The results of the study also showed that postoperative smoking cessation for one to six months (18·2%) or for more than six months (17·1%) did not change the nonunion rate, compared with continuous smokers (26·5%) or non-smokers (14·2%).

One study also showed that compared with continuous smokers, smoking cessation for between three and six weeks (55·0% vs. 85·7%, *P*=0·02) and for more than six weeks (59·1% vs. 85·7%, *P*=0·01) significantly reduced the impaired wound healing rate.[115]

However, one study[118] with 130 patients and another study[117] with 57 patients showed that no statistically significant difference was found in the comparison of smoking cessation for two days before surgery vs. continuous smokers (14% vs. 13%, respectively; *P*=0·86) and smoking cessation for between two and three weeks vs. continuous smokers (11% vs. 13%, respectively; *P*=1·00).

For other wound complications, one study including 748 patients showed that no significant difference was found in dehiscence or seroma between smoking cessation for three weeks before surgery and non-smokers.[107] Another study with 108 patients also showed that smoking cessation for six to eight weeks before surgery did not significantly reduce the hematoma rate, compared with continuous smokers.[108] One study[110] with 169 patients and another study[114] with 1,335 patients showed that smoking cessation for four weeks or more significantly reduced the combination of wound complications, compared with continuous smokers, whereas there was no significant difference compared with non-smokers.

S14b. List of studies and results about the impact of NRT and vaping on bone healing and woun d complication

Outcomes	NRT	NRT vs. continuous smokers
Wound infection	NRT (unknown in detail)	1 study[117]
	NRT as a partially way to quit smoking	1 study[113]
	Nicorette chewing gum as a partially way to quit smoking	1 study[122]
	Nicorette patch, nicorette chewing gum, nicorette resoriblet, ni corette inhalat, or nicorette nasal spray	1 study[105]

Note: NRT: nicotine replacement therapy

In two studies, NRT did not significantly reduce wound infection, compared with continued smokers.[105,117] I n two studies, NRT was used as a part of the smoking cessation methods.[113,122] The results of wound infectio n were inconsistent (3·7% vs. 12·5%, P=0·03; 4% vs. 7%, P=0·68). However, the cessation methods were differ ent from each other.[113,122] In addition, no study concerning vaping that met inclusion and exclusion criteria was found.

Appendix S15. Forest plots of the meta-analyses of 15a) impaired wound healing rate of smoking cessation vs. non-smokers and 15b) hematoma rate of smoking cessation vs. continuous smokers

15a

Study or Subgroup	smoking cessation Events	smoking cessation Total	nonsmoker Events	nonsmoker Total	Weight	Odds Ratio M-H, Random, 95% CI
**1.1.1 >3 weeks**						
Delaunay 2018	31	71	25	96	57.1%	2.20 [1.14, 4.23]
Kuri 2005	50	86	19	40	42.9%	1.54 [0.72, 3.26]
**Subtotal (95% CI)**		**157**		**136**	**100.0%**	**1.89 [1.15, 3.09]**
Total events	81		44			
Heterogeneity: Tau² = 0.00; Chi² = 0.50, df = 1 (P = 0.48); I² = 0%						
Test for overall effect: Z = 2.52 (P = 0.01)						
**1.1.2 ≥4 weeks**						
Delaunay 2018	31	71	25	96	59.4%	2.20 [1.14, 4.23]
Kuri 2005	39	66	19	40	40.6%	1.60 [0.72, 3.52]
**Subtotal (95% CI)**		**137**		**136**	**100.0%**	**1.93 [1.17, 3.20]**
Total events	70		44			
Heterogeneity: Tau² = 0.00; Chi² = 0.38, df = 1 (P = 0.54); I² = 0%						
Test for overall effect: Z = 2.56 (P = 0.01)						

Test for subgroup differences: Chi² = 0.00, df = 1 (P = 0.95), I² = 0%

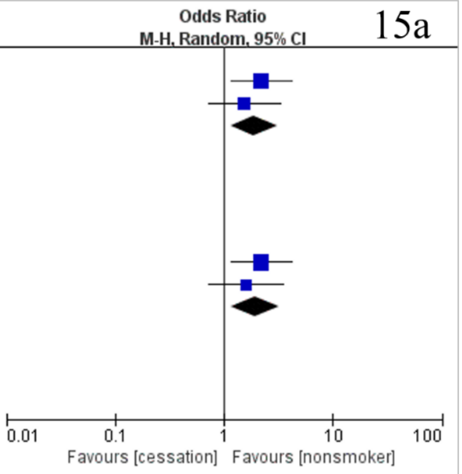

15b

Study or Subgroup	smoking cessation Events	smoking cessation Total	continuous smokers Events	continuous smokers Total	Weight	Odds Ratio M-H, Random, 95% CI
**1.3.1 ≥4 weeks**						
Lindström 2008	3	48	7	54	71.3%	0.45 [0.11, 1.84]
Moller 2002	1	56	4	52	28.7%	0.22 [0.02, 2.02]
**Subtotal (95% CI)**		**104**		**106**	**100.0%**	**0.36 [0.11, 1.20]**
Total events	4		11			
Heterogeneity: Tau² = 0.00; Chi² = 0.29, df = 1 (P = 0.59); I² = 0%						
Test for overall effect: Z = 1.66 (P = 0.10)						

Test for subgroup differences: Not applicable

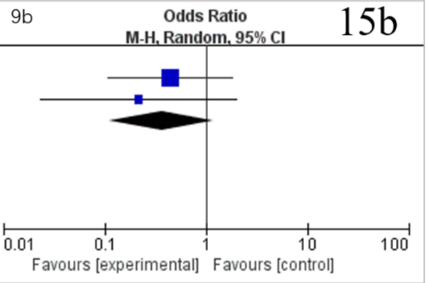

Appendix S16. Funnel plots and Rucker's test for publication bias

Funnel plots of the meta-analyses of S16a) nonunion rate, S16b) deep surgical site infection (DSSI) rate, and S16c) undefined surgical site infection (SSI) rate of smokers vs. non-smokers

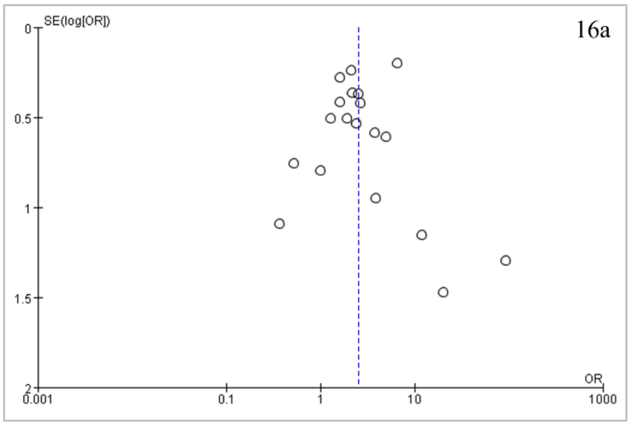

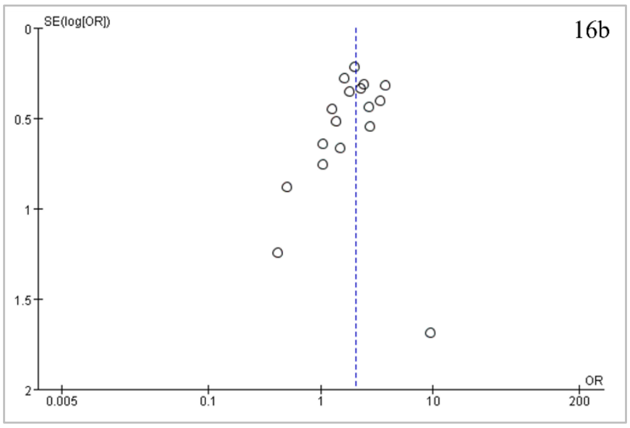

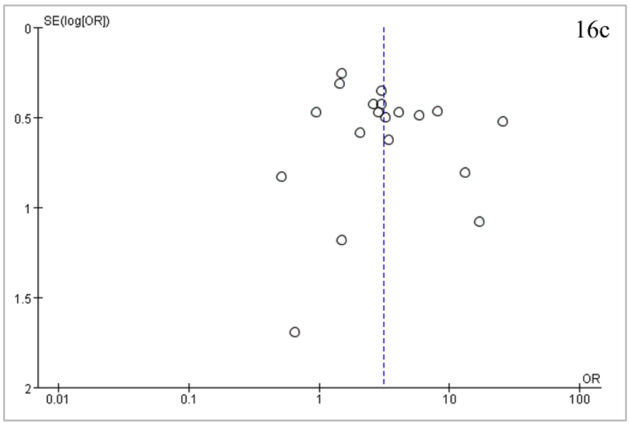

Appendix S16d. Rucker's test for publication bias

Outcomes	*t* value	*P* value	bias	se.bias	intercept
Nonunion	-0.41	0.69	-0.33	0.80	1.13
DSSI	-1.41	0.18	-0.79	0.56	1.01
Undefined SSI	1.17	0.26	1.23	1.05	0.46

Appendix S17. Results of the dose-related analysis of outcomes

When regarding the time to union, only one study[1] investigated alcohol drinkers who drank more than 28 g/day and non-drinkers. The results showed that the time to union in drinkers was significantly longer than that in non-drinkers (OR 12·85, 95% CI [1·23–24·47], *P*=0·03, I^2=NA). For undefined SSI, three studies[15,38,46] compared the effects of smoking more than 10 cigarettes/day, and one of the three studies[15] compared the effect of smoking more than 20 cigarettes/day with non-smokers. The results indicated that patients who had smoked more than 10 cigarettes/day were associated with an obviously larger undefined SSI rate (OR 4·93, 95% CI [1·36–17·89], *P*=0·02, I^2=33%). However, no significant difference was found between smokers who had smoked 20 cigarettes/day and non-smokers (OR 3·86, 95% CI [1·00–14·96], *P*=0·05, I^2=NA). Moreover, one study[15] compared undefined SSI between patients who drank more than 28 g/day and non-drinkers. However, no significant difference was found between the two groups (OR 2·79, 95% CI [0·77–10·08], *P*=0·12, I^2=NA).

Appendix S18. Results of the subgroup analysis

The results showed that the difference in the nonunion rate between smokers and non-smokers was not significant in studies where (1) age ≥60-years-old (two studies with 103 patients,[1,26] odds ratio [OR] 2.06, 95% CI [0·07–62·65], *P*=0·68, I^2=79%); (2) diabetes ≥20% (two studies with 217 patients,[11,26] OR 3·76, 95% CI [1·00–14·12], *P*=0·05, I^2=39%); and (3) sample size ≤100 individuals (eight studies with 538 patients,[1,4,20,24,26,29,33,54] OR 2·64, 95% CI [0·93–7·55], *P*=0·07, I^2=68%). For a comparison of drinkers vs. non-drinkers, the difference in the nonunion rate was not significant in studies in which (1) body mass index (BMI) >30 kg/m^2 (one study with 144 patients,[11] OR 2·43, 95% CI [1·13–5·25], *P*=0·02, I^2=NA), (2) diabetes ≥20% (one study with 144 patients,[11] OR 2·43, 95%CI [1·13–5·25], *P*=0·02, I^2=NA), and (3) sample size of between 101 and 300 individuals (one study with 144 patients,[11] OR 2·43, 95% CI [1·13–5·25], *P*=0·02, I^2=NA).

Deep infection rate was no different in studies with (1) percentage of females ≥50% (two studies[4,47], OR 1·03, 95% CI [0·47–2·27], *P*=0·93, I^2=0%); (2) mean or median ages ≥60-years-old (one study[49], OR 1·04, 95% CI [0·24–4·61], *P*=0·95, I^2=NA); (3) mean or median BMI <25 kg/m^2 (two studies[49,52], OR 1·25, 95% CI [0·54–2·89], *P*=0·60, I^2=0%) and BMI >30 kg/m^2 (one study[47], OR 1·25, 95% CI [0·52–3·04], *P*=0·62, I^2=NA); and (4) sample size ≤100 individuals (four studies[4,24,29,42], OR 0·89, 95% CI [0·36–2·19], *P*=0·80, I^2=0%) and sample size between 701 and 1,000 individuals (one study[47], OR 1·25, 95% CI [0·52–3·04], *P*=0·62, I^2=NA). In addition, for undefined infection rate, the significant difference disappeared in studies with (1) percentage of females ≥50% (six studies[13,14,18,39,46,98], OR 1·86, 95% CI [1·01–3·43], *P*=0·05, I^2=44%); (2) mean or median ages ≥60-years-old (one study[46], OR 0·64, 95% CI [0·02–17·82], *P*=0·80, I^2=NA); and (3) sample size ≤100 individuals (three studies[15,38,46], OR 3·42, 95% CI [0·74–15·77], *P*=0·12, I^2=55%) and sample size >1,000 individuals (two studies[14,43], OR 2·04, 95% CI [1·00–4·18], *P*=0·05, I^2=58%). However, for the remaining outcomes, no obvious differences were found when the studies were divided into subgroups.

For a comparison of drinkers vs. non-drinkers, a significant difference in nonunion rate was shown in studies (1) in which mean or median BMI >30 kg/m^2 (one study[11], OR 2·43, 95% CI [1·13–5·25], *P*=0·02, I^2=NA); (2) percentage of diabetes ≥20% (one study[11], OR 2·43, 95% CI [1·13–5·25], *P*=0·02, I^2=NA); and (3) sample size between 101 and 300 individuals (one study[11], OR 2·43, 95% CI [1·13–5·25], *P*=0·02, I^2=NA). A significant difference was found in undefined SSI in studies with (1) BMI between 25 to 30 kg/m^2 (one study,[57] OR 4·14, 95% CI [1·68–10·20], *P*=0·002, I^2=NA); (2) percentage of diabetes <20% (five studies,[2,12,16,50,56] OR 1·52, 95% CI [1·02–2·27], *P*=0·04, I^2=0%); and (3) sample size between 301 and 500 individuals (one study,[57] OR 4·14, 95% CI [1·68–10·20], *P*=0·002, I^2=NA).

		**Smokers vs. Non-smokers**					**Alcohol drinkers vs. Non-drinkers**				
		n	I^2	OR [95% CI]	*p*	*p* for subgroup differences	*n*	I^2	OR [95% CI]	*p*	*p* for subgroup differences
Delayed union											
Female%	<50%	2	0%	1·76 (0·60–5·23)	0·31	0·26	-	-	-	-	-
	≥50%	1	-	0·42 (0·04–4·02)	0·45		-	-	-	-	

Age	<60	2	0%	1·11 (0·34–3·56)	0·86	0·54	-	-	-	-	-
	≥60	1	-	2·15 (0·36–13·05)	0·40		-	-	-	-	
Diabetes%	<20%	1	-	0·42 (0·04–4·02)	0·45	-	-	-	-	-	-
	≥20%	0	-	-	-		-	-	-	-	
Sample size	≤100	3	0%	1·35 (0·51–3·59)	0·55	-	-	-	-	-	-
	101–300	0	-	-	-		-	-	-	-	
	301–500	0	-	-	-		-	-	-	-	
	501–700	0	-	-	-		-	-	-	-	
	701–1000	0	-	-	-		-	-	-	-	
	>1000	0	-	-	-		-	-	-	-	
Confounders adjusted or not	Adjusted	0	-	-	-	-	-	-	-	-	-
	Not adjusted	3	0%	1·35 (0·51–3·59)	0·55		-	-	-	-	
Study design	RCT	0	-	-	-	-	-	-	-	-	-
	cohort study	3	0%	1·35 (0·51–3·59)	0·55		-	-	-	-	
	case-control study	0	-	-	-		-	-	-	-	
Nonunion											
Female%	<50%	14	69%	2·67 (1·64–4·35)	<0·0001	0·61	1	-	0·57 (0·21–1·54)	0·27	0·36
	≥50%	4	9%	2·25 (1·47–3·46)	0·0002		2	87%	1·22 (0·34–4·41)	0·76	
Age	<60	16	65%	2·62 (1·78–3·84)	<0·0001	0·89	3	77%	0·97 (0·40–2·38)	0·95	-
	≥60	2	79%	2·06 (0·07–62·65)	**0·68**		0	-	-	-	
BMI	<25	1	-	2·12 (1·32–3·40)	0·002	0·37	1	-	0·66 (0·39–1·09)	0·10	0·005
	25–30	2	0%	4·75 (1·70–13·23)	0·003		0	-	-	-	
	>30	2	0%	2·30 (1·28–4·13)	0·005		1	-	2·43 (1·13–5·25)	**0·02**	
Diabetes%	<20%	10	39%	2·37 (1·54–3·65)	<0·0001	0·52	1	-	0·57 (0·21–1·54)	0·27	0·02

	≥20%	2	39%	3·76 (1·00–14·12)	**0·05**		1	-	2·43 (1·13–5·25)	**0·02**	
Sample size	≤100	8	68%	2·64 (0·93–7·55)	**0·07**	0·81	0	-	-	-	0·01
	101–300	6	0%	2·37 (1·59–3·53)	<0·0001		1	NA	2·43 (1·13–5·25)	**0·02**	
	301–500	1	NA	2·20 (1·07–4·49)	0·03		0	-	-	-	
	501–700	2	0%	1·87 (1·18–2·96)	0·007		1	NA	0·57 (0·21–1·54)	0·27	
	701–1000	2	92%	3·72 (1·25–11·07)	0·02		1	NA	0·66 (0·39–1·09)	0·10	
	>1000	0	-	-	-		0	-	-	-	
Confounders adjusted or not	Adjusted	4	0%	2·71 (1·52–4·85)	0·0008	0·74	0	-	-	-	-
	Not adjusted	15	70%	2·40 (1·56–3·69)	<0·0001		3	77%	0·97 (0·40–2·38)	0·95	
Study design	RCT	0	-	-	-	0·56	0	-	-	-	-
	cohort study	9	36%	2·19 (1·25–3·84)	0·006		0	-	-	-	
	case-control study	10	75%	2·73 (1·68–4·46)	<0·0001		3	77%	0·97 (0·40–2·38)	0·95	
Time to union											
Female%	<50%	2	0%	0·52 (-1·29–2·33)	0·57	-	1	-	12·85 (1·23–24·47)	0·03	-
	≥50%	0	-	-	-		0	-	-	-	
Age	<60	1	-	0·58 (-1·25–2·41)	0·53	0·65	0	-	-	-	-
	≥60	1	-	-2·45 (-15·33–10·43)	0·71		1	-	12·85 (1·23–24·47)	0·03	
Diabetes%	<20%	1	-	0·58 (-1·25–2·41)	0·53	-	-	-	-	-	-
	≥20%	0	-	-	-		-	-	-	-	
Sample size	≤100	2	0%	0·52 (-1·29–2·33)	0·57	-	1	-	12·85 (1·23–24·47)	0·03	-
	101–300	0	-	-	-		0	-	-	-	
	301–500	0	-	-	-		0	-	-	-	
	501–700	0	-	-	-		0	-	-	-	
	701–1000	0	-	-	-		0	-	-	-	

	>1000	0	-	-	-		0	-	-	-	
Confounders adjusted or not	Adjusted	0	-	-	-	-	0	-	-	-	-
	Not adjusted	2	0%	0·52 (-1·29–2·33)	0·57		1	-	12·85 (1·23–24·47)	0·03	
Study design	RCT	0	-	-	-	-	0	-	-	-	-
	cohort study	1	-	-2·45 (-15·33–10·43)	0·71		1	-	12·85 (1·23–24·47)	0·03	
	case-control study	0	-	-	-		0	-	-	-	
Normal union											
Female%	<50%	3	0%	1·56 (0·56–4·33)	0·40	0·94	-	-	-	-	-
	≥50%	2	0%	1·64 (0·61–4·44)	0·33		-	-	-	-	
Age	<60	4	0%	1·87 (0·84–4·16)	0·13	0·40	-	-	-	-	-
	≥60	1	-	0·88 (0·18–4·23)	0·87		-	-	-	-	
BMI	<25	0	-	-	-	-	-	-	-	-	-
	25–30	1	-	1·44 (0·46–4·45)	0·53		-	-	-	-	
	>30	0	-	-	-		-	-	-	-	
Diabetes%	<20%	2	0%	1·64 (0·61–4·44)	0·62	-	-	-	-	-	-
	≥20%	0	-	-	-		-	-	-	-	
Sample size	≤100	4	0%	1·72 (0·69–4·31)	0·25	0·81	-	-	-	-	-
	101–300	1	-	1·44 (0·46–4·45)	0·53		-	-	-	-	
	301–500	0	-	-	-		-	-	-	-	
	501–700	0	-	-	-		-	-	-	-	
	701–1000	0	-	-	-		-	-	-	-	
	>1000	0	-	-	-		-	-	-	-	
Confounders adjusted or not	Adjusted	0	-	-	-	-	-	-	-	-	-
	Not adjusted	5	0%	1·60 (0·78–3·26)	0·20		-	-	-	-	

Study design	RCT	1	-	2·64 (0·57–12·25)	0·22	0·77	-	-	-	-	-
	cohort study	3	0%	1·35 (0·43–4·26)	0·61		-	-	-	-	
	case-control study	1	-	1·44 (0·46–4·45)	0·53		-	-	-	-	
SSSI											
Female%	<50%	2	0%	1·48 (0·37–5·91)	0·58	0·82	-	-	-	-	-
	≥50%	2	0%	1·24 (0·68–2·25)	0·48		-	-	-	-	
Age	<60	4	0%	1·27 (0·73–2·21)	0·39	-	-	-	-	-	-
	≥60	0	-	-	-		-	-	-	-	
BMI	<25	0	-	-	-	-	-	-	-	-	-
	25–30	0	-	-	-		-	-	-	-	
	>30	1	-	1·20 (0·65–2·22)	0·56		-	-	-	-	
Diabetes%	<20%	4	0%	1·27 (0·73–2·21)	0·39	-	-	-	-	-	-
	≥20%	0	-	-	-		-	-	-	-	
Sample size	≤100	3	0%	1·63 (0·47–5·66)	0·44	0·67	-	-	-	-	-
	101–300	0	-	-	-		-	-	-	-	
	301–500	0	-	-	-		-	-	-	-	
	501–700	0	-	-	-		-	-	-	-	
	701–1000	1	-	1·20 (0·65–2·22)	0·56		-	-	-	-	
	>1000	0	-	-	-		-	-	-	-	
Confounders adjusted or not	Adjusted	1	-	1·20 (0·65–2·22)	0·56	0·67	-	-	-	-	-
	Not adjusted	3	0%	1·63 (0·47–5·66)	0·44		-	-	-	-	
Study design	RCT	0	-	-	-	-	-	-	-	-	-
	cohort study	3	0%	1·26 (0·71–2·26)	0·43		-	-	-	-	
	case-control study	0	-	-	-		-	-	-	-	

DSSI											
Female%	<50%	13	0%	2·02 (1·63–2·50)	<0·0001	0·11	2	0%	1·02 (0·64–1·62)	0·94	-
	≥50%	2	0%	1·03 (0·47–2·27)	0·93		0	-	-	-	
Age	<60	13	0%	1·96 (1·58–2·44)	<0·0001	0·41	2	0%	1·02 (0·64–1·62)	0·94	-
	≥60	1	-	1·04 (0·24–4·61)	0·95		0	-	-	-	
BMI	<25	2	0%	1·25 (0·54–2·89)	0·60	0·34	1	-	1·03 (0·61–1·75)	0·91	0·94
	25–30	2	0%	2·12 (1·49–3·02)	<0·0001		1	-	0·98 (0·37–2·62)	0·97	
	>30	1	-	1·25 (0·52–3·04)	0·62		0	-	-	-	
Diabetes%	<20%	15	4%	2·05 (1·67–2·52)	<0·0001	-	2	0%	1·02 (0·64–1·62)	0·94	-
	≥20%	0	-	-	-		0	-	-	-	
Sample size	≤100	4	0%	0·89 (0·36–2·19)	**0·80**	0·14	0	-	-	-	0·94
	101–300	5	0%	2·63 (1·77–3·91)	<0·0001		0	-	-	-	
	301–500	3	0%	2·17 (1·49–3·17)	<0·0001		0	-	-	-	
	501–700	3	0%	1·80 (1·29–2·50)	0·0005		1	-	0·98 (0·37–2·62)	0·97	
	701–1000	1	-	1·25 (0·52–3·04)	**0·62**		0	-	-	-	
	>1000	1	-	3·40 (1·53–7·56)	0·003		1	-	1·03 (0·61–1·75)	0·91	
Confounders adjusted or not	Adjusted	4	0%	1·92 (1·39–2·64)	<0·0001	0·67	0	-	-	-	-
	Not adjusted	13	12%	2·10 (1·60–2·75)	<0·0001		2	0%	1·02 (0·64–1·62)	0·94	
Study design	RCT	0	-	-	-	0·82	0	-	-	-	-
	cohort study	7	23%	2·09 (1·38–3·17)	0·0005		0	-	-	-	
	case-control study	9	0%	1·98 (1·56–2·50)	<0·0001		2	0%	1·02 (0·64–1·62)	0·94	
Undefined SSI											
Female%	<50%	10	32%	3·06 (2·12–4·43)	<0·0001	0·33	8	53%	1·44 (0·89–2·32)	0·14	-
	≥50%	5	55%	1·93 (0·82–4·55)	**0·13**		0	-	-	-	

Age	<60	15	69%	3·44 (2·11–5·60)	<0·0001	0·33	8	53%	1·44 (0·89–2·32)	0·14	-
	≥60	1	-	0·64 (0·02–17·82)	**0·80**		0	-	-	-	
BMI	<25	0	-	-	-	-	1	-	1·42 (0·72–2·79)	0·31	-
	25–30	4	60%	2·98 (1·12–7·95)	0·03		1	-	4·14 (1·68–10·20)	**0·002**	
	>30	0	-	-	-		0	-	-	-	
Diabetes%	<20%	12	74%	3·44 (1·98–5·98)	<0·0001	0·43	5	0%	1·52 (1·02–2·27)	0·04	0·54
	≥20%	2	0%	3·11 (1·52–6·40)	0·002		2	66%	2·30 (0·66–8·00)	0·19	
Sample size	≤100	3	55%	3·42 (0·74–15·77)	**0·12**	0·001	1	-	1·16 (0·37–3·62)	0·80	0·96
	101–300	7	58%	2·44 (1·19–5·04)	0·02		2	0%	1·61 (0·79–3·27)	0·19	
	301–500	2	2%	5·73 (2·97–11·04)	<0·0001		1	-	4·14 (1·68–10·20)	0·002	
	501–700	3	0%	2·90 (1·70–4·93)	<0·0001		1	-	1·31 (0·58–2·96)	0·52	
	701–1000	1	-	25·56 (9·16–71·33)	<0·0001		2	81%	0·90 (0·16–5·24)	0·91	
	>1000	2	58%	2·04 (1·00–4·18)	**0·05**		1	-	1·42 (0·72–2·79)	0·31	
Confounders adjusted or not	Adjusted	5	5%	2·59 (1·70–3·93)	<0·0001	0·36	4	63%	1·18 (0·51–2·74)	0·71	0·78
	Not adjusted	14	73%	3·55 (2·07–6·09)	<0·0001		5	67%	1·37 (0·75–2·50)	0·31	
Study design	RCT	0	-	-	-	0·37	0	-	-	-	0·12
	cohort study	7	20%	2·23 (1·45–3·44)	0·0003		3	0%	0·82 (0·53–1·28)	0·39	
	case-control study	11	75%	3·24 (1·85–5·69)	<0·0001		6	66%	1·51 (0·81–2·83)	0·20	

Note: OR: odds ratio; CI: confidence interval; RCT: randomised controlled trial; BMI: body mass index; SSSI: superficial surgical site infection; DSSI: deep surgical site infection; SSI: surgical site infection.

The subgroup analyses showed that the impact of smoking on increasing rates of nonunion, DSSI, and undefined SSI was not obvious in elderly patients an d studies with small sample sizes. For diabetic patients, smoking did not have an obvious impact on nonunion. Moreover, there was no significant impact of smoking on infection in female patients. In addition, smoking also had no impact on DSSI in normal weight and obese patients, as well as in studies with sample sizes of 701 to 1,000 individuals. For undefined SSI, smoking had no impact in studies with sample sizes over 1,000 individuals. However, due to the small number of studies that were included in these subgroups, this result requires more clinical studies to verify its stability. This was the same study i n which drinking had a negative effect on nonunion in diabetic and obese patients, as well as the study with a small sample size. In addition, drinking had

a negative impact on undefined SSI in overweight patients, non-diabetic patients, and studies with 301 to 500 patients. These results also require more research to verify.

Appendix S19. Results of the sensitivity analysis

We also conducted a sensitivity analysis for the outcomes to identify potential heterogeneity. The results of the sensitivity analysis showed that the nonunion rate was not different between smokers and non-smokers in one study[11] including 144 patients with a low risk of bias in the prognostic factor measurement domain (odds ratio [OR] 2·43, 95% CI [1·13–5·25], *P*=0·02) and two studies[22,58] including 284 patients with a low risk of bias in the statistical analysis and reporting domain (OR 2·23, 95% CI [0·93–5·38], *P*=0·07)· For the comparison between alcohol drinkers and non-drinkers, the difference in the nonunion rate became significant in one study[11] with a low risk of bias in the prognostic factor measurement domain (OR 2·43, 95% CI [1·13–5·25], *P*=0·02).

We found that two studies[15,46] investigating undefined SSI (OR 1·82, 95% CI [0·62–5·38], *P*=0·28, I^2=0%), which had a low risk of bias in the prognostic factor measurement domain, were possible causes of heterogeneity. One study[22] that evaluated the effects of smoking on nonunion (OR 3·90, 95% CI [0·61–24·94], *P*=0·15, I^2=NA) could also be a reason for the heterogeneity because of the low risk of bias in the statistical analysis and reporting domain. In addition, two studies[4,49] investigating DSSI (OR 0·76, 95% CI [0·25–2·35], *P*=0·64, I^2=0%) may be possible causes of heterogeneity because of a clear description of non-pathological fracture. The remaining factors did not show obvious causes of heterogeneity.

For the comparison of alcohol drinkers vs. non-drinkers, we found that one study[11] investigating the nonunion rate (OR 1·02, 95% CI [1·13–5·25], *P*= 0·02, I^2=NA), which had a low risk of bias in the prognostic factor measurement domain, was a possible cause of heterogeneity. Six studies[2,12,15,50,56,57] investigating the impact of alcohol consumption on undefined SSI (OR 1·84, 95% CI [1·23–2·77], *P*=0·003, I^2=1%), which had a low risk of bias in the outcome measurement domain, were possible causes of heterogeneity.

	**Smokers vs. Non-smokers**				**Alcohol drinkers vs. Non-drinkers**			
	No. of studies	I^2	OR (95% CI)	*p*	No. of studies	I^2	OR (95% CI)	*p*
1. Studies without fund assisted								
Delayed union rate	2	0%	1·11 (0·34–3·56)	0·86	-	-	-	-
Nonunion rate	15	64%	2·55 (1·71–3·81)	<0·0001	3	77%	0·97 (0·40–2·38)	0·95
Normal union rate	4	0%	1·87 (0·84–4·16)	0·13	-	-	-	-
Time to union	-	-	-	-	-	-	-	-
SSSI	3	0%	1·25 (0·71–2·20)	0·43	-	-	-	-

DSSI	13	8%	2·13 (1·67–2·71)	<0·0001	1	-	1·03 (0·61–1·75)	0·91
Undefined SSI	15	71%	3·33 (1·98–5·60)	<0·0001	5	25%	1·12 (0·75–1·67)	0·58
2. Studies with low risk_study Participation								
Delayed union rate	2	19%	1·10 (0·23–5·36)	0·90	-	-	-	-
Nonunion rate	15	53%	2·75 (1·92–3·95)	<0·0001	3	77%	0·97 (0·40–2·38)	0·95
Normal union rate	3	0%	1·37 (0·59–3·18)	0·46	-	-	-	-
Time to union	2	0%	0·52 (-1·29–2·33)	0·57	1	-	12·85 (1·23–24·47)	0·03
SSSI	3	0%	1·26 (0·71–2·26)	0·43	-	-	-	-
DSSI	12	0%	1·91 (1·52–2·40)	<0·0001	2	0%	1·02 (0·64–1·62)	0·94
Undefined SSI	16	65%	3·20 (2·10–4·88)	<0·0001	8	53%	1·44 (0·89–2·32)	0·14
3. Studies with low risk_prognostic Factor Measurement								
Nonunion rate	1	-	2·15 (0·88–5·23)	**0·09**	1	-	2·43 (1·13–5·25)	**0·02**
Undefined SSI	2	0%	1·82 (0·62–5·38)	**0·28**	1	-	1·16 (0·37–3·62)	0·80
4. Studies with low risk_outcome Measurement								
Delayed union rate	1	-	2·15 (0·36–13·05)	0·40	-	-	-	-
Nonunion rate	13	70%	2·72 (1·76–4·22)	<0·0001	3	77%	0·97 (0·40–2·38)	0·95
Normal union rate	1	-	0·88 (0·18–4·23)	0·87	-	-	-	-
Time to union	1	-	-2·45 (-15·33–10·43)	0·71	1	-	12·85 (1·23–24·47)	0·03
SSSI	1	-	1·20 (0·65–2·22)	0·56	-	-	-	-
DSSI	9	0%	1·97 (1·57–2·48)	<0·0001	2	0%	1·02 (0·64–1·62)	0·94
Undefined SSI	14	67%	3·63 (2·32–5·67)	<0·0001	6	1%	1·84 (1·23–2·77)	**0·003**
5. Studies with low risk_statistical Analysis and Reporting								
Nonunion rate	2	0%	2·23 (0·93–5·38)	**0·07**	-	-	-	-
SSSI	1	-	1·20 (0·65–2·22)	0·56	-	-	-	-

DSSI	4	0%	1·92 (1·39–2·64)	<0·0001	-	-	-	-
Undefined SSI	4	37%	2·12 (1·18–3·82)	0·01	2	0%	2·14 (0·97–4·70)	**0·06**
6. Studies without mandible and spine fracture								
Delayed union rate	3	0%	1·35 (0·51–3·59)	0·55	-	-	-	-
Nonunion rate	18	65%	2·45 (1·67–3·59)	<0·0001	3	77%	0·97 (0·40–2·38)	0·95
Normal union rate	4	0%	1·39 (0·62–3·12)	0·42	-	-	-	-
Time to union	2	0%	0·52 (-1·29–2·33)	0·57	-	-	-	-
SSSI	4	0%	1·27 (0·73–2·21)	0·39	-	-	-	-
DSSI	16	1%	2·07 (1·70–2·52)	<0·0001	2	0%	1·02 (0·64–1·62)	0·94
Undefined SSI	19	66%	3·11 (2·09–4·63)	<0·0001	9	60%	1·28 (0·81–2·03)	0·29
7. Studies with fractures in single location								
Delayed union rate	3	0%	1·35 (0·51–3·59)	0·55	-	-	-	-
Nonunion rate	17	67%	2·47 (1·63–3·74)	<0·0001	3	77%	0·97 (0·40–2·38)	0·95
Normal union rate	5	0%	1·60 (0·78–3·26)	0·20	-	-	-	-
Time to union	2	0%	0·52 (-1·29–2·33)	0·57	1	-	12·85 (1·23–24·47)	0·03
SSSI	3	0%	1·26 (0·71–2·26)	0·43	-	-	-	-
DSSI	14	10%	2·06 (1·62–2·62)	<0·0001	-	-	-	-
Undefined SSI	15	69%	3·15 (2·00–4·97)	<0·0001	7	39%	1·00 (0·66–1·51)	1·00
8. Studies without case series								
Delayed union rate	3	0%	1·35 (0·51–3·59)	0·55	-	-	-	-
Nonunion rate	19	64%	2·50 (1·73–3·61)	<0·0001	3	77%	0·97 (0·40–2·38)	0·95
Normal union rate	5	0%	1·60 (0·78–3·26)	0·20	-	-	-	-
Time to union	1	-	-2·45 (-15·33–10·43)	0·71	1	-	12·85 (1·23–24·47)	0·03
SSSI	3	0%	1·26 (0·71–2·26)	0·43	-	-	-	-

DSSI	16	1%	2·03 (1·67–2·47)	<0·0001	2	0%	1·02 (0·64–1·62)	0·94
Undefined SSI	18	66%	2·94 (1·98–4·38)	<0·0001	9	60%	1·28 (0·81–2·03)	0·29
9. Studies with non-pathological fractures								
Delayed union rate	2	19%	1·10 (0·23–5·36)	0·90	-	-	-	-
Nonunion rate	10	70%	3·03 (1·76–5·22)	<0·0001	3	77%	0·97 (0·40–2·38)	0·95
Normal union rate	3	0%	1·37 (0·59–3·18)	0·46	-	-	-	-
Time to union	1	-	-2·45 (-15·33–10·43)	0·71	1	-	12·85 (1·23–24·47)	0·03
SSSI	1	-	2·45 (0·14–42·82)	0·54	-	-	-	-
DSSI	2	0%	0·76 (0·25–2·35)	0·64	2	0%	1·02 (0·64–1·62)	0·94
Undefined SSI	6	3%	3·73 (2·37–5·87)	<0·0001	6	66%	1·51 (0·81–2·83)	0·20
10. Studies with operative treatment								
Delayed union rate	1	-	0·42 (0·04–4·02)	0·45	-	-	-	-
Nonunion rate	9	43%	2·90 (1·76–4·79)	<0·0001	1	-	0·57 (0·21–1·54)	0·27
Normal union rate	3	0%	2·07 (0·80–5·41)	0·14	-	-	-	-
Time to union	1	-	0·58 (-1·25–2·41)	0·53	-	-	-	-
SSSI	3	0%	1·25 (0·71–2·20)	0·43	-	-	-	-
DSSI	13	7%	2·11 (1·67–2·65)	<0·0001	2	0%	1·02 (0·64–1·62)	0·94
Undefined SSI	17	70%	3·19 (2·05–4·96)	<0·0001	7	68%	1·23 (0·71–2·15)	0·46
11. Removing one study at a time for outcomes with high hetero geneity								
Nonunion rate excluding Ding 2014					2	87%	1·22 (0·34–4·41)	0·76
Nonunion rate excluding Liu 2015					2	81%	1·22 (0·29–5·09)	0·79
Nonunion rate excluding Thorud 2017					2	0%	0·64 (0·40–1·00)	0·05
12. Removing three studies with high OR for smoking and nonun ion as reviewer suggested (Kim 2018, OR 12; Moghaddam 2011, OR 20.01; Subramanyam 2021, OR 93.00)								

Nonunion rate	16	61%	2·22 (1·57–3·14)	<0·0001	

Note: OR: odds ratio; SSSI: superficial surgical site infection; DSSI: deep surgical site infection; SSI: surgical site infection.

The sensitivity analysis showed that studies with a low risk of bias in the prognostic factor measurement domain, outcome measurement domain, and statistical analysis and reporting domain changed the trend of the results regarding nonunion and undefined SSI. This change may be because the number of studies that met the sensitivity analysis was only one or two studies, which indicates that more studies with a low risk of bias in these two domains are needed to verify the results. High heterogeneity was found in quantitative synthesis for the impact of alcohol consumption on nonunion rate. After removing one study at a time for nonunion rate, we found that although the impact of alcohol consumption on nonunion rate was still not significant, the heterogeneity significantly decreased after the study Throrud 2017 was removed (OR 0·97, 95% CI [0·40–2·38], I^2=77% to OR 0·64, 95% CI [0·40–1·00], I^2=0%). Therefore, Thorud 2017 may lead to the heterogeneity. The possible reasons for this heterogeneity might be (1) the included sample size is small (n=144) and (2) the expanded definition of nonunion, that not only included fractures that failed to union after 6 months after fracture diagnosis, but also included fractures that failed to meet criteria with correctional surgery before 6 months after fracture diagnosis. The authors in the Thorud 2017 did not describe what "failure to meet criteria with correctional surgery before 6 months after fracture diagnosis" meant. We thought this might mean that the radiographic results show that the fracture alignment is not satisfactory within 6 months after fracture diagnosis, which might lead to reoperation and increase the number of final nonunion cases. For the impact of smoking on nonunion, three studies with high ORs (Kim 2018, Moghaddam 2011, and Subramanyam 2021) were included in the meta-analysis. The high ORs of the three studies may be due to the small sample size (Kim 2018, *n*=73; Moghaddam 2011, *n*=85; Subramanyam 2021, *n*=100). We conducted a sensitivity analysis by removing these three studies to confirm the stability of the results and found the results did not significantly changed (from OR 2·50, 95% CI [1·73–3·61] to OR 2·22, 95% CI [1·57–3·14]), which means that the results are stable.

Appendix S20. Problems in the included studies in four previous systematic reviews concerning the effects of smoking on nonunion and surgical site infection

Problems in the included studies in four previous systematic reviews about smoking and nonunion were described in **Appendix S20a and S20b**. Problems in the included studies in four previous systematic reviews about smoking and surgical site infection were described in **Appendix S20c and S20e**. Result of recalculation of smoking and surgical site infection data from Shao J 2018 was shown in **Appendix S20d**. Conclusion of problems in the included studies was described in **Appendix S20f**.

S20a. Problems in the included studies about smoking and nonunion in Tian RF 2020

No.	No. of reference in previous systematic reviews	Author/Publication year	outcomes (smokers)	total (smokers)	outcomes (non-smokers)	total (non-smokers)	Problems in the included studies
1	19	O'Halloran K 2016	nonunion (29)	153	nonunion (27)	229	
2	39	Jin PF 2018	nonunion (13)	63	nonunion (13)	134	This is a clinical study in Chinese with low quality. In addition, no definition of nonunion was described.
3	45	Dailey HL 2018	nonunion (38)	244	nonunion (36)	261	No time period of nonunion was described.
4	46	Singh A 2018	nonunion (16)	26	nonunion (28)	77	There was no definition of nonunion. In addition, the study uses nonunion in several sentences to describe this outcome, and uses delayed union in other sentences and Table 3, which is difficult to understand.
5	50	Zura R 2017	nonunion (178)	1573	nonunion (766)	11235	No time period of nonunion was described.
6	57	Blair JA 2016	nonunion (7)	48	nonunion (9)	136	
7	94	Lack WD 2014	nonunion (6)	90	nonunion (7)	86	The study did not describe whether the included patients were adults or the age range of the patients.
8	113	Enninghorst N 2011	nonunion (10)	23	nonunion (16)	63	
9	135	Harvey EJ 2002	nonunion (42)	62	nonunion (20)	48	Patients under 18-years old (15–76 years) were included. In addition, Tian RF et al miscalculated the outcome of nonunion. Only ununited (not healed at the end of the study) was considered non-union, but late union (between 270 days and final follow-up) was not included.

S20b. Problems in the included studies about smoking and nonunion in Pearson RG 2016

No.	No. of reference in previou s systematic reviews	Author/Publication year	outcomes (smokers)	total (smokers)	outcomes (non-smokers)	total (non-smokers)	Problems in the included studies
1	5	Kyro A 1993	nonunion (36)	72	nonunion (20)	63	We were unable to verify whether the study met the inclusion and exclusion criteria because we could not obtain the literature even by email.
2	6	Schmitz MA 1999	nonunion (2)	44	nonunion (0)	64	
3	7	Adams CI 2001	nonunion (46)	140	nonunion (33)	133	Patients under 18-years old (13–90 years) were i ncluded. In addition, no time period of nonunion was described.
4	8	Harvey EJ 2002	nonunion (42)	62	nonunion (20)	48	Patients under 18-years old (15–76 years) were i ncluded. In addition, Pearson RG et al. miscalcu lated the outcome of nonunion. Only ununited (not healed at the end of the study) was consid ered non-union, but late union (between 270 day s and final follow-up) was not included.
5	9	Mckee MD 2003	nonunion (10)	47	nonunion (2)	39	Patients under 18-years old (17–70 years) were i ncluded. In addition, no definition of nonunion was described.
6	10	Castillo RC 2005	nonunion (34)	187	nonunion (8)	81	Patients under 18-years old (16–69 years) were i ncluded.
7	11	Ristiniemi J 2007	delayed union (7)	16	delayed union (5)	31	Patients under 18-years old (15–79 years) were i ncluded. Control group (non-current smokers) ma y include former smokers. In addition, Pearson RG et al. mistook delayed union (16–24 weeks) as nonunion in their study.
8	12	Enninghorst N 2011	nonunion (10)	23	nonunion (16)	63	
9	13	Moghaddam A 2011	nonunion (9)	46	nonunion (0)	39	
10	14	Ahmed 2018	nonunion (8)	8	nonunion (10)	10	Although no definition of nonunion was describe d, time to union of each fracture was recorded. However, Pearson RG et al. incorrectly defined the time period of nonunion.
11	15	Ding L 2014	nonunion (11)	165	nonunion (13)	494	
12	16	Tay WH 2014	delayed union or nonunion (66)	161	delayed union or nonunion (72)	262	Delayed union and nonunion were mixed.
13	17	Liu W 2015	nonunion (30)	155	nonunion (66)	649	
14	18	Ruffolo MR 2015	nonunion (5)	54	nonunion (9)	86	Patients under 18-years old (16–78 years) were i ncluded.

S20c. Problems in the included studies about smoking and surgical site infection in Shao J 2018

In Shao J 2018, five studies were included in the meta-analysis, but the authors did not describe which five studies were. We re-screened the literature included in the Shao J 2018 and found that two more studies could be included in meta-analysis. Therefore, we listed these seven studies below and performed meta-analysis again using the data (**Appendix S20d**).

No.	No. of reference in Shao J 2018	Author/Publication year	outcomes (smokers)	total (smokers)	outcomes (non-smokers)	total (non-smokers)	Problems in the included studies
1	4	Korim MT 2014	infection (8)	18	infection (21)	692	
2	13	Nasell H 2011	DSSI (9)<br>SSSI (27)	183	DSSI (6)<br>SSSI (69)	708	Patients under 18-years old (≥15 years) were included.
3	22	Olsen LL 2017	DSSI (21)<br>infection (39)	283	DSSI (43)<br>infection (106)	760	Patients under 18-years old (9–95 years) were included.
4	25	Sun R 2017	infection (11)	262	infection (35)	985	Control group (non-current smokers) may include former smokers.
5	26	Sun Y 2018	infection (13)	355	infection (53)	1155	Control group (non-current smoking) may include former smokers.
6	28	Naumann MG 2016	infection (10)	136	infection (19)	431	Control group (non-current smokers) may include former smokers.
7	48	Ovaska MT 2013	DSSI (47)	64	DSSI (84)	198	

S20d. Resynthesis of data on smoking and surgical site infection from Shao J 2018

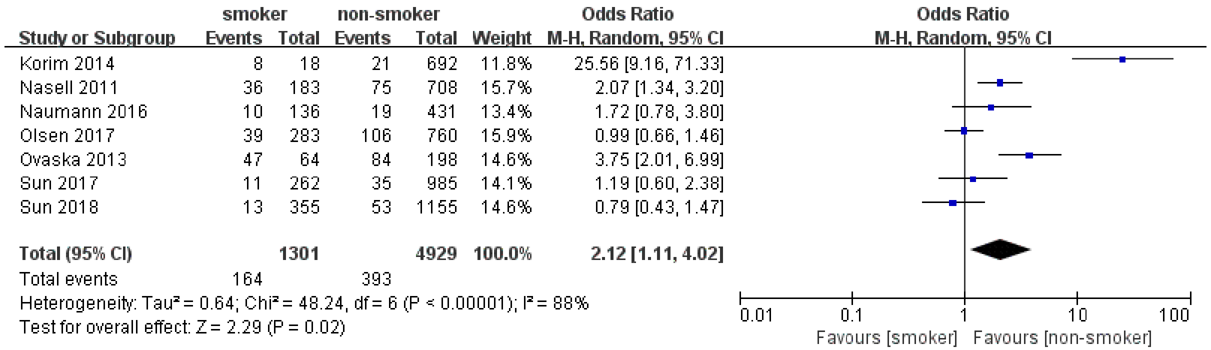

S20e. Problems in the included studies about smoking and surgical site infection in Kortram K 2017

No.	No. of reference in previous systematic reviews	Author/Publication year	outcomes (smokers)	total (smokers)	outcomes (non-smokers)	total (non-smokers)	Problems in the included studies

1	20	Enninghorst N 2011	infection (4)	23	infection (11)	66	
2	23	Molina CS 2015	DSSI (30)	149	DSSI (27)	206	Patients under 18-years old (≥15 years) were included. In addition, control group (non-active smoker) may include former smokers.
3	24	Ovaska MT 2016	no available data	32	no available data	78	Patients under 18-years old (≥16 years) were included. Kortram K et al. mistook wound necrosis as wound infection in their study.
4	25	Lawing CR 2015	SSSI (4)<br>DSSI (11)	73	SSSI (12).<br>DSSI (25)	278	Patients under 18-years old (≥10 years) were included. Control group (None to less than half a pack per day) may include smokers.
5	27	Lack WD 2014	infection (8)	39	infection (16)	98	The study did not describe the patients' age range.
6	33	Zumsteg JW 2014	DSSI (4)	63	DSSI (6)	137	
7	36	Reuss BL 2007	infection (4)	20	infection (3)	34	Patients under 18-years old (>17 years) were included.
8	37	Castillo RC 2005	infection (37)	187	infection (12)	81	Patients under 18-years old (16–69 years) were included.
9	38	Adams CI 2001	Pin track infection (8)<br>soft tissue infection (21)<br>DSSI (14)	140	Pin track infection (9)<br>soft tissue infection (13)<br>DSSI (14)	133	Patients under 18-years old (13–90 years) were included.
10	39	Merritt K 1988	infection (6)	34	infection (7)	36	The study did not describe minimum age of the patients (<20, 21–40, 41–60, >60 years). Therefore, patients under 18 years old may be included.
11	40	Penn-Barwell JG 2016	infection (5)	23	infection (12)	44	The study did not describe whether the included patients were adults or the age range of the patients.

Note: SSSI: superficial surgical site infection; DSSI: deep surgical site infection.

S20f. Conclusion of problems in previous studies that led to unreliable results

Most of the studies that were included in previous systematic reviews were inappropriate for the following reasons:

(1) Lack of time period in the definition of nonunion used for evaluation.[35,79,123-128]

(2) Delayed union and nonunion were mixed.[129]

(3) Patients under 18-years-old[123,124,126,127,130-137] were included, or the studies had no descriptions of the age ranges of patients.[138-140]

(4) The control group may have included former smokers.[16,75,98,134,141,142]

(5) One study[125] in the Chinese language was included in the data synthesis, which may have reduced the quality of evidence and may have created bias.[143]

(6) Uncomprehensive search strategies were performed.

Appendix S21. Problems in the included studies in two previous systematic reviews about effects of alcohol drinking on nonunion and surgical site infection

S21a. Problems in the included studies about alcohol drinking and nonunion in Tian RF 2020

No.	No. of reference in previous systematic reviews	Author/Publication year	outcomes (alcohol drinkers)	total (alcohol drinkers)	outcomes (non-drinkers)	total (non-drinkers)	Problems in the included studies
1	19	O'Halloran K 2016	nonunion (20)	104	nonunion (36)	278	Control group (non-drinkers or social drinkers) may include alcohol drinkers.
2	50	Zura R 2017	nonunion (22)	244	nonunion (922)	12564	Control group (non-alcoholism) may include alcohol drinkers. In addition, no time period of nonunion was described.

S21b. Problems in the included studies about alcohol drinking and surgical site infection in Shao J 2018

In Shao J 2018, three studies were included in the meta-analysis, but the authors did not describe which three studies were. We re-screened the literature included in the Shao J 2018 and found that two more studies could be included in meta-analysis. Therefore, we listed these five studies below and performed meta-analysis again using the data (**Appendix S21c**).

No.	No. of reference in previous systematic reviews	Author/Publication year	outcomes (alcohol drinkers)	total (alcohol drinkers)	outcomes (non-drinkers)	total (non-drinkers)	Problems in the included studies
1	4	Korim MT 2014	infection (5)	6	infection (24)	704	Control group (non-excess alcohol drinkers) may include drinkers.
2	22	Olsen LL 2017	DSSI (22)<br>infection (47)	243	DSSI (42)<br>infection (97)	800	Patients under 18-years old (9–95) were included. In addition, control group (non-alcohol overuse) may include alcohol drinkers.
3	25	Sun R2017	infection (12)	251	infection (34)	996	
4	26	Sun Y2018	infection (11)	150	infection (55)	1360	Control group (non-alcoholism) may include alcohol drinkers.
5	48	Ovaska MT2013	DSSI (29)	41	DSSI (102)	160	Control group (non-alcohol abuser) may include drinkers.

Note: DSSI: deep surgical site infection.

S21c. Resynthesis of data on alcohol drinking and surgical site infection from Shao J 2018

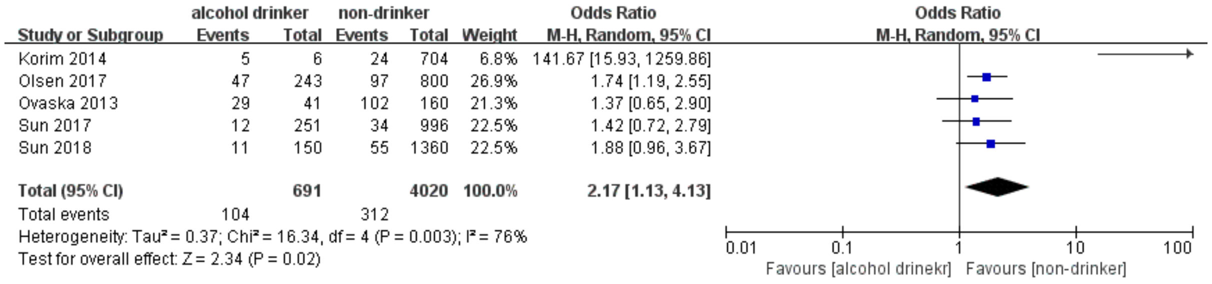

S21d. Problems in the included studies about alcohol drinking and surgical site infection in Kortram K 2017

In Kortram K 2017, three studies were included in the meta-analysis, and the authors clearly described which three studies were. However, outcome that wa s described in one study Ovaska MT 2016 was wound necrosis rather than wound infection. In Ovaska MT 2016, 10 of 18 patients with wound necrosis w ere considered patients with wound infection. However, Kortram K 2017 considered those 18 patients with wound necrosis as wound infection. Since the nu mber of infection events in alcohol drinker group and non-drinker group was not clearly described in Ovaska MT 2016, we used the data of remaining two studies to perform resynthesis (**Appendix S21e**).

No.	No. of reference in previo us systematic reviews	Author/Publication y ear	outcomes (alcohol drinkers)	total (alcohol drinkers)	outcomes (non-drinkers)	total (non-drinkers)	Problems in the included studies
1	24	Ovaska MT 2016	no available data	39	no available data	71	Firstly, patients under 18-years old (≥16 years) were included. Secondly, control group (non-a lcohol abuser) may include drinker. Lastly, out come in Ovaska MT 2016 was necrosis rather than infection. Ten of 18 patients with wound necrosis were considered to have a deep infect ion. However, Kortram K 2017 considered tho se 18 patients with necrosis as patients with wound infection.
2	25	Lawing CR 2015	SSSI (8)<br>DSSI (22)	159	SSSI (8)<br>DSSI (14)	192	Patients under 18-years old (≥10 years) were i ncluded. In addition, control group (none or o ccasional drinker) may include drinker.

3	39	Merritt K 1988	infection (7)	35	infection (6)	35	The study did not describe minimum age of the patients (<20, 21–40, 41–60, >60 years). Therefore, patients under 18 years old may be included.

Note: SSSI: superficial surgical site infection; DSSI: deep surgical site infection.

S21e. Resynthesis of data on alcohol drinking and surgical site infection from Kortram K 2017

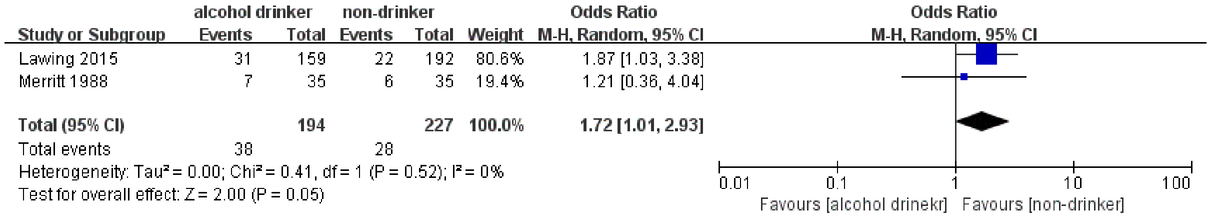

Appendix S22. Problems in the two previous meta-analyses concerning the effects of preoperative smoking cessation time on wound healing and wound complications

No	Study/study design	Included studies	Surgery	Smoking cessation time	Outcomes (cessation)	total (cessation)	Outcomes (continued smokers)	total (continued smokers)	Results and problems
1	Sorensen 2012[144]	Lindstrom 2008	Herniotomy, cholecystectomy, hip, or knee arthroplasty	4 weeks pre-op through 4 weeks after	combined wound complication (6)<br>wound infection (2)	48	combined wound complication (14)<br>wound complication (4)	54	Results: The meta-analysis disclosed that perioperative smoking cessation did not significantly reduce healing complications combined (OR 0·48, 95%CI [0·19–1·25], P=0·14, I^2=61·1%).<br>In contrast, surgical site infections were significantly reduced by perioperative smoking cessation as shown by a sensitivity analysis (OR 0·40, 95%CI [0·20–0·83], P=0·01, I^2=19·8%).<br>Problem:<br>Smoking cessation time was not classified.
		Moller 2002	Hip or knee arthroplasty	6–8 weeks pre-op through 10 days after	combined wound complication (3)<br>wound infection (2)	56	combined wound complication (16)<br>wound infection (12)	52	
		Sorensen 2003	Colorectal resection	2–3 weeks pre-op through 10 days after	combined wound complication (9)<br>wound infection (3)	27	combined wound complication (8)<br>wound infection (4)	30	
		Sorensen 2007	Inguinal or incisional herniotomy	4 weeks pre-op through 10 days after	wound infection (6)	101	wound infection (4)	48	
2	Wong 2012[145]	Chan 2006	Bilateral breast reduction surgery	≥1 month pre-op	wound healing problem (5)	15	wound healing problem (10)	19	Results:<br>Ex-smokers who quit more than 3 to 4 weeks before surgery had lower relative risks of postoperative wound healing complications than those who quit less than 3 to 4 weeks before surgery (RR 0·74, 95%CI [0·56–0·98], P=0·04, I^2=34%).<br>Problems:<br>1. In Chang 1999 and Goodwin 2005, patients were classified as former smokers if they had stopped smoking at least 4 weeks before surgery. However, smoking cessation may not be done during the study, that is, patients who had quit smoking for a long time may also be included in the study.<br>In order to study the effect of a clear time to quit smoking before surgery, we did not include these studies.<br>2. The data of Glassman 2000 was bone nonunion. However, the author of the meta-analysis misjudged it
		Chang 1999	Free TRAM flap breast reconstruction	≥4 weeks pre-op	wound healing complication (15)	150	wound healing complication (23)	90	
		Glassman 2000	spinal fusion	>1 month pre-op	wound healing complication (10)	51	wound healing complication (15)	74	
		Goodwin 2005	tissue expander/implant breast reconstruction	4 weeks pre-op	overall complication (31)	78	overall complication (20)	54	
		Kuri 2005	removal of nasal, oral, pharyngeal, laryngeal, or cervical esophageal cancer and reconstruction	8–21 days, 22–42 days	impaired wound healing (11)	20	impaired wound healing (23)	34	
		Lindstrom 2008	Herniotomy, cholecystectomy, hip, or knee arthroplasty	4 weeks pre-op through 4 weeks after	combined wound complication (2)	20	combined wound complication (0)	9	

		Padubidri 2001	Breast reconstructio n	≥3 weeks pre-op	combined wound h ealing complication (19)	76	combined wound hea ling complication (6 1)	155	as wound healing complication.<br>3. The overall complication or wound complication th at was combined outcomes was analysed instead of e ach outcome in detail.

Note: OR: odds ratio; RR: risk ratio

References

[1] Askew A, Chakkalakal D, Fang X, McGuire M. Delayed fracture healing in alcohol Abusers-A preliminary retrospective study. Open Bone Journal. 2011;3:1-5.

[2] Bai Y, Zhang X, Tian Y, Tian D, Zhang B. Incidence of surgical-site infection following open reduction and internal fixation of a distal femur fracture: An observational case-control study. Medicine (United States). 2019;98(7).

[3] Ding L, He Z, Xiao H, Chai L, Xue F. Factors affecting the incidence of aseptic nonunion after surgical fixation of humeral diaphyseal fracture. Journal of Orthopaedic Science. 2014;19(6):973-7.

[4] Kent M, Mumith A, McEwan J, Hancock N. The service impact of failed locking plate fixation of distal tibial fractures: a service and financial evaluation at a major trauma centre. European Journal of Orthopaedic Surgery and Traumatology. 2015;25(8):1333-42.

[5] Liu W, Xiao J, Ji F, Xie Y, Hao Y. Intrinsic and extrinsic risk factors for nonunion after nonoperative treatment of midshaft clavicle fractures. Orthopaedics and Traumatology: Surgery and Research. 2015;101(2):197-200.

[6] Ma Q, Aierxiding A, Wang G, Wang C, Yu L, Shen Z. Incidence and risk factors for deep surgical site infection after open reduction and internal fixation of closed tibial plateau fractures in adults. International Wound Journal. 2018;15(2):237-42.

[7] McKissack HM, Viner GC, Jha AJ, et al. Comparison of risk factors for postoperative complications across age groups in patients undergoing ORIF of the ankle. Injury. 2019.

[8] Meng J, Sun T, Zhang F, Qin S, Li Y, Zhao H. Deep surgical site infection after ankle fractures treated by open reduction and internal fixation in adults: A retrospective case-control study. International Wound Journal. 2018;15(6):971-7.

[9] Ovaska MT, Mäkinen TJ, Madanat R, et al. Risk factors for deep surgical site infection following operative treatment of ankle fractures. Journal of Bone and Joint Surgery - Series A. 2013;95(4):348-53.

[10] Parker B, Petrou S, Achana F, Masters JPM, Costa ML. Economic outcomes associated with deep surgical site infection in patients with an open fracture of the lower limb. Bone and Joint Journal. 2018;100B(11):1506-10.

[11] Thorud JC, Mortensen S, Thorud JL, Shibuya N, Maldonado YM, Jupiter DC. Effect of Obesity on Bone Healing After Foot and Ankle Long Bone Fractures. Journal of Foot & Ankle Surgery. 2017;56(2):258-62.

[12] Bowen TR, Widmaier JC. Host classification predicts infection after open fracture. Clinical Orthopaedics and Related Research. 2005(433):205-11.

[13] Chan G, Iliopoulos E, Jain A, Turki M, Trompeter A. Infection after operative fixation of tibia plateau fractures. A risk factor analysis. Injury. 2019.

[14] Claessen FMAP, Braun Y, van Leeuwen WF, Dyer GS, van den Bekerom MPJ, Ring D. What

Factors are Associated With a Surgical Site Infection After Operative Treatment of an Elbow Fracture? Clinical Orthopaedics and Related Research. 2016;474(2):562-70.

[15] Flynn JM, Rodriguez-del Rio F, Piza PA. Closed ankle fractures in the diabetic patient. Foot Ankle Int. 2000;21(4):311-9.

[16] Sun R, Li M, Wang X, et al. Surgical site infection following open reduction and internal fixation of a closed ankle fractures: A retrospective multicenter cohort study. International Journal of Surgery. 2017;48:86-91.

[17] Alzahrani MM, Cota A, Alkhelaifi K, et al. Are clinical outcomes affected by type of plate used for management of mid-shaft clavicle fractures? Journal of Orthopaedics and Traumatology. 2018;19(1).

[18] Basques BA, Webb ML, Bohl DD, Golinvaux NS, Grauer JN. Adverse events, length of stay, and readmission following surgery for tibial plateau fractures. Journal of Orthopaedic Trauma. 2014.

[19] Blair JA, Stoops TK, Doarn MC, et al. Infection and nonunion after fasciotomy for compartment syndrome associated with tibia fractures: A matched cohort comparison. Journal of Orthopaedic Trauma. 2016;30(7):392-6.

[20] Bone LB, Sucato D, Stegemann PM, Rohrbacher BJ. Displaced isolated fractures of the tibial shaft treated with either a cast or intramedullary nailing: An outcome analysis of matched pairs of patients. Journal of Bone and Joint Surgery - Series A. 1997;79(9):1336-41.

[21] Branch NN, Obirieze A, Wilson RH. Assessment of racial and sex disparities in open femoral fractures. American Journal of Surgery. 2015;209(4):666-74.

[22] Chu JY, Yeh KT, Lee RP, et al. Open reduction and internal fixation with plating is beneficial in the early recovery stage for displaced midshaft clavicular fractures in patients aged 30-65 years old. Tzu Chi Medical Journal. 2018;30(4):242-6.

[23] Donohue D, Sanders D, Serrano-Riera R, et al. Ketorolac administered in the recovery room for acute pain management does not affect healing rates of femoral and tibial fractures. Journal of Orthopaedic Trauma. 2016;30(9):479-82.

[24] Enninghorst N, McDougall D, Hunt JJ, Balogh ZJ. Open tibia fractures: Timely debridement leaves injury severity as the only determinant of poor outcome. Journal of Trauma - Injury, Infection and Critical Care. 2011;70(2):352-7.

[25] Haller JM, Githens M, Rothberg D, Higgins T, Nork S, Barei D. Risk Factors for Tibial Plafond Nonunion: Medial Column Fixation May Reduce Nonunion Rates. Journal of orthopaedic trauma. 2019;33(9):443-9.

[26] Kim SM, Yeom JW, Song HK, Hwang KT, Hwang JH, Yoo JH. Lateral locked plating for distal femur fractures by low-energy trauma: what makes a difference in healing? International Orthopaedics. 2018;42(12):2907-14.

[27] Kuklo TR, Groth AT, Anderson RC, Frisch HM, Islinger RB. Recombinant human bone morphogenetic protein-2 for grade III open segmental tibial fractures from combat injuries in Iraq.

Journal of Bone and Joint Surgery - Series B. 2008;90(8):1068-72.
[28] McDonald E, Winters B, Nicholson K, et al. Effect of Postoperative Ketorolac Administration on Bone Healing in Ankle Fracture Surgery. Foot and Ankle International. 2018;39(10):1135-40.
[29] Moghaddam A, Zimmermann G, Hammer K, Bruckner T, Grützner PA, Von Recum J. Cigarette smoking influences the clinical and occupational outcome of patients with tibial shaft fractures. Injury. 2011;42(12):1435-42.
[30] Momaya AM, Hlavacek J, Etier B, et al. Risk factors for infection after operative fixation of Tibial plateau fractures. Injury. 2016;47(7):1501-5.
[31] Morris BJ, Unger RZ, Archer KR, Mathis SL, Perdue AM, Obremskey WT. Risk factors of infection after ORIF of bicondylar tibial plateau fractures. Journal of Orthopaedic Trauma. 2013;27(9):e196-e200.
[32] Murray IR, Foster CJ, Eros A, Robinson CM. Risk factors for nonunion after nonoperative treatment of displaced midshaft fractures of the clavicle. Journal of Bone and Joint Surgery - Series A. 2013;95(13):1153-8.
[33] Özbek Z, Özkara E, Önner H, et al. Treatment of Unstable Thoracolumbar Fractures: does Fracture-Level Fixation Accelerate the Bone Healing? World neurosurgery. 2017;107:362-70.
[34] Shah CM, Babb PE, McAndrew CM, et al. Definitive plates overlapping provisional external fixator pin sites: Is the infection risk increased? Journal of Orthopaedic Trauma. 2014;28(9):518-22.
[35] Singh A, Tan Jiong Hao J, Tan Wei D, et al. Gustilo IIIB Open Tibial Fractures: An analysis of Infection and Nonunion Rates. Indian Journal of Orthopaedics. 2018;52(4):406-10.
[36] Snäll J, Apajalahti S, Suominen AL, Törnwall J, Thorén A. Influence of perioperative dexamethasone on delayed union in mandibular fractures: A clinical and radiological study. Medicina Oral, Patologia Oral y Cirugia Bucal. 2015;20(5):e621-e6.
[37] Zumsteg JW, Molina CS, Lee DH, Pappas ND. Factors influencing infection rates after open fractures of the radius and/or ulna. Journal of Hand Surgery. 2014;39(5):956-61.
[38] Assous M, Bhamra MS. Should Os calcis fractures in smokers be fixed? A review of 40 patients. Injury. 2001;32(8):631-2.
[39] Hoffmann MF, Jones CB, Sietsema DL, Tornetta 3rd P, Koenig SJ. Clinical outcomes of locked plating of distal femoral fractures in a retrospective cohort. Journal of orthopaedic surgery and research. 2013;8:43.
[40] Li J, Zhu Y, Liu B, Dong T, Chen W, Zhang Y. Incidence and risk factors for surgical site infection following open reduction and internal fixation of adult tibial plateau fractures. International Orthopaedics. 2018;42(6):1397-403.
[41] Lin S, Mauffrey C, Hammerberg EM, Stahel PF, Hak DJ. Surgical site infection after open reduction and internal fixation of tibial plateau fractures. European Journal of Orthopaedic Surgery and Traumatology. 2014;24(5):797-803.
[42] McGarvey WC, Burris MW, Clanton TO, Melissinos EG. Calcaneal fractures: Indirect reduction

and external fixation. Foot and Ankle International. 2006;27(7):494-9.
[43] Minhas SV, Catalano LW. Comparison of Open and Closed Hand Fractures and the Effect of Urgent Operative Intervention. Journal of Hand Surgery. 2019;44(1):65.e1-.e7.
[44] Korim MT, Payne R, Bhatia M. A case-control study of surgical site infection following operative fixation of fractures of the ankle in a large U.K. trauma unit. Bone Joint J. 2014;96-b(5):636-40.
[45] Ren T, Ding L, Xue F, He Z, Xiao H. Risk factors for surgical site infection of pilon fractures. Clinics (São Paulo, Brazil). 2015;70(6):419-22.
[46] Spross C, Platz A, Erschbamer M, Lattmann T, Dietrich M. Surgical treatment of neer group vi proximal humeral fractures: Retrospective comparison of PHILOS® and hemiarthroplasty. Clinical Orthopaedics and Related Research. 2012;470(7):2035-42.
[47] Benedick A, Rivera T, Vallier HA. Effect of Tourniquet Use During Ankle Fracture Fixation on Wound Healing and Infectious Complications. Foot and Ankle International. 2020.
[48] Esposito JG, van der Vliet QMJ, Heng M, et al. Does surgical approach influence the risk of post-operative infection following surgical treatment of tibial pilon fractures? Journal of orthopaedic trauma. 2019.
[49] Ogihara S, Yamazaki T, Shiibashi M, et al. Risk Factor Analysis of Deep Surgical Site Infection After Posterior Instrumented Fusion Surgery for Spinal Trauma: A Multicenter Observational Study. World Neurosurgery. 2020;134:e524-e9.
[50] Li J, Wang Q, Lu Y, et al. Relationship Between Time to Surgical Debridement and the Incidence of Infection in Patients with Open Tibial Fractures. Orthopaedic Surgery. 2020.
[51] Ahmed ASA, Singer MS, El Bigawi HA. Neglected tibial pilon fractures: Can arthrodesis be avoided? Journal of Orthopaedic Trauma. 2018;32(7):369-75.
[52] Ukai T, Hamahashi K, Uchiyama Y, Kobayashi Y, Watanabe M. Retrospective analysis of risk factors for deep infection in lower limb Gustilo–Anderson type III fractures. Journal of Orthopaedics and Traumatology. 2020;21(1).
[53] Olson JJ, Anand K, von Keudell A, et al. Judicious Use of Early Fixation of Closed, Complete Articular Pilon Fractures Is Not Associated With an Increased Risk of Deep Infection or Wound Complications. J Orthop Trauma. 2021;35(6):300-7.
[54] Subramanyam KN, Mundargi AV, Gopakumar KU, Bharath T, Prabhu MV, Khanchandani P. Displaced midshaft clavicle fractures in adults - is non-operative management enough? Injury. 2021;52(3):493-500.
[55] Mardanpour K, Rezaei M, Rahbar M, Mardanpour S. Risk factors for surgical site infections after open reduction and internal fixation of acetabulum fracture in the west of Iran. International Journal of Surgery Open. 2020;27:119-22.
[56] Tan Z, Wang Z, Wang Y, Hu H, Zhang Y, Chen W. Prevalence and risk factors of surgical site infection after closed isolated patella fracture surgery: A prospective cohort study. Int Orthop. 2021.

[57] Zhu C, Zhang J, Li J, et al. Incidence and predictors of surgical site infection after distal femur fractures treated by open reduction and internal fixation: a prospective single-center study. BMC Musculoskelet Disord. 2021;22(1):258.
[58] Olson JJ, Entezari V, Vallier HA. Risk factors for nonunion after traumatic humeral shaft fractures in adults. JSES international. 2020;4(4):734-8.
[59] Arastu M, Sheehan B, Buckley R. Minimally invasive reduction and fixation of displaced calcaneal fractures: Surgical technique and radiographic analysis. International Orthopaedics. 2014;38(3):539-45.
[60] Bhandari M, Spargue S, Hanson B, et al. Health-related quality of life following operative treatment of unstable ankle fractures: A prospective observational study. Journal of Orthopaedic Trauma. 2004;18(6):338-45.
[61] Sohn HS, Kim WJ, Shon MS. Comparison between open plating versus minimally invasive plate osteosynthesis for **acute displaced clavicular shaft fractures**. Injury. 2015;46(8):1577-84.
[62] Unlu S, Catma MF, Bilgetekin YG, et al. Minimally Invasive Plate Osteosynthesis of Distal Tibia and Fibular Fractures Through a Single Distal Anterolateral Incision. Journal of Foot and Ankle Surgery. 2015;54(6):1081-4.
[63] Formica M, Cavagnaro L, Basso M, et al. Which patients risk segmental kyphosis after short segment thoracolumbar fracture fixation with intermediate screws? Injury. 2016;47:S29-S34.
[64] Frantz TL, Meschbach NT, Niedermeier SR, Pettit RJ, Plummer DR, Harrison RK. Factors influencing time to union of diaphyseal humeral fractures after plate fixation: A retrospective cohort study. Current Orthopaedic Practice. 2019.
[65] Pastides PS, Milnes L, Rosenfeld PF. Percutaneous Arthroscopic Calcaneal Osteosynthesis: A Minimally Invasive Technique for Displaced Intra-Articular Calcaneal Fractures. Journal of Foot and Ankle Surgery. 2015;54(5):798-804.
[66] Burnett Z, Barzee BM, Hymes R, Cannada LK. Clinical Outcomes following Tongue Type Calcaneus Fractures. Techniques in Orthopaedics. 2019.
[67] Johnston LE, Parker MJ. Hip fractures and chronic alcohol excess: A series of 7,023 cases. HIP International. 2014;24(6):644-9.
[68] Nyquist F, Berglund M, Nilsson BE, Obrant KJ. Nature and healing of tibial shaft fractures in alcohol abusers. Alcohol and Alcoholism. 1997;32(1):91-5.
[69] Tonnesen H, Pedersen A, Jensen MR, Moller A, Madsen JC. Ankle fractures and alcoholism. The influence of alcoholism on morbidity after malleolar fractures. Journal of Bone and Joint Surgery - Series B. 1991;73(3):511-3.
[70] Lin S, Xie J, Yao X, Dai Z, Wu W. The Use of Cryotherapy for the Prevention of Wound Complications in the Treatment of Calcaneal Fractures. Journal of Foot and Ankle Surgery. 2018;57(3):436-9.
[71] White TO, Guy P, Cooke CJ, et al. The results of early primary open reduction and internal

fixation for treatment of OTA 43.C-type tibial pilon fractures: A cohort study. Journal of Orthopaedic Trauma. 2010;24(12):757-63.

[72] Krishnan B. The Role of a Conservative Minimal Interventional Management Protocol in the Fractures of the Dentate Portion of the Adult Mandible. Craniomaxillofacial Trauma and Reconstruction. 2015;9(1):20-8.

[73] Zura R, Xiong Z, Einhorn T, et al. Epidemiology of fracture nonunion in 18 human bones. JAMA Surgery. 2016;151(11).

[74] Gaal A, Bailey B, Patel Y, et al. Limiting Antibiotics When Managing Mandible Fractures May Not Increase Infection Risk. Journal of Oral and Maxillofacial Surgery. 2016;74(10):2008-18.

[75] Sun Y, Wang H, Tang Y, et al. Incidence and risk factors for surgical site infection after open reduction and internal fixation of ankle fracture. Medicine (United States). 2018;97(7).

[76] Zura R, Braid-Forbes MJ, Jeray K, et al. Bone fracture nonunion rate decreases with increasing age: A prospective inception cohort study. Bone. 2017;95:26-32.

[77] Boesmueller S, Wech M, Gregori M, et al. Risk factors for humeral head necrosis and non-union after plating in proximal humeral fractures. Injury. 2016;47(2):350-5.

[78] Cook SD, Ryaby JR, McCabe J, Frey JJ, Heckman JD, Kristiansen TK. Acceleration of tibia and distal radius fracture healing in patients who smoke. Clinical Orthopaedics and Related Research. 1997(337):198-207.

[79] Dailey HL, Wu KA, Wu PS, McQueen MM, Court-Brown CM. Tibial fracture nonunion and time to healing after reamed intramedullary nailing: Risk factors based on a single-center review of 1003 patients. Journal of Orthopaedic Trauma. 2018;32(7):E263-E9.

[80] Dingemans SA, Birnie MFN, Backes M, et al. Prophylactic negative pressure wound therapy after lower extremity fracture surgery: a pilot study. International Orthopaedics. 2018;42(4):747-53.

[81] Femino JE, Vaseenon T, Levin DA, Yian EH. Modification of the sinus tarsi approach for open reduction and plate fixation of **intra-articular calcaneus fractures**: the limits of proximal extension based upon the vascular anatomy of the lateral calcaneal artery. The Iowa orthopaedic journal. 2010;30:161-7.

[82] Giannoudis PV, MacDonald DA, Matthews SJ, Smith RM, Furlong AJ, De Boer P. Nonunion of the femoral diaphysis. Journal of Bone and Joint Surgery - Series B. 2000;82(5):655-8.

[83] Moghaddam A, Weiss S, Wölfl CG, et al. Cigarette smoking decreases TGF-β1 serum concentrations after long bone fracture. Injury. 2010;41(10):1020-5.

[84] Rose PS, Adams CR, Torchia ME, Jacofsky DJ, Haidukewych GG, Steinmann SP. Locking plate fixation for proximal humeral fractures: Initial results with a new implant. Journal of Shoulder and Elbow Surgery. 2007;16(2):202-7.

[85] Thompson P, Patel V, Fallat LM, Jarski R. Surgical Management of Fifth Metatarsal Diaphyseal Fractures: A Retrospective Outcomes Study. Journal of Foot and Ankle Surgery. 2017;56(3):463-7.

[86] Li Q, Liu P, Wang G, et al. Risk Factors of Surgical Site Infection after Acetabular Fracture

Surgery. Surgical Infections. 2015;16(5):577-82.
[87] Xu H, Yu L, Li Y, Gong Z. Prolonged surgical duration, higher body mass index and current smoking increases risk of surgical site infection after intra-articular fracture of distal femur. ANZ journal of surgery. 2019;89(6):723-8.
[88] Zhu Y, Liu S, Zhang X, Chen W, Zhang Y. Incidence and risks for surgical site infection after adult tibial plateau fractures treated by ORIF: a prospective multicentre study. International Wound Journal. 2017;14(6):982-8.
[89] Fourgeaux A, Estens J, Fabre T, Laffenetre O, Lucas y Hernandez J. Three-dimensional computed tomography analysis and functional results of calcaneal fractures treated by an intramedullary nail. International Orthopaedics. 2019;43(12):2839-47.
[90] Hoffmann MF, Khoriaty JD, Sietsema DL, Jones CB. Outcome of intramedullary nailing treatment for intertrochanteric femoral fractures. Journal of orthopaedic surgery and research. 2019;14(1):360.
[91] Serrano R, Mir HR, Sagi HC, et al. Modern Results of Functional Bracing of Humeral Shaft Fractures: A Multicenter Retrospective Analysis. Journal of orthopaedic trauma. 2020;34(4):206-9.
[92] Hu J, Zhang C, Zhu K, et al. Adverse Radiographic Outcomes Following Operative Treatment of Medial Malleolar Fractures. Foot and Ankle International. 2018;39(11):1301-11.
[93] Alemdaroğlu KB, Tiftikçi U, Iltar S, et al. Factors affecting the fracture healing in treatment of tibial shaft fractures with circular external fixator. Injury. 2009;40(11):1151-6.
[94] Grün W, Molund M, Nilsen F, Stødle AH. Results After Percutaneous and Arthroscopically Assisted Osteosynthesis of Calcaneal Fractures. Foot and Ankle International. 2020;41(6):689-97.
[95] Vander Voort W, Davison J, Hendrickson N, et al. Sarcopenia is Associated with Nonunion of Open Tibia and Ankle Fractures. The Iowa orthopaedic journal. 2020;40(1):153-8.
[96] Barış A, Çirci E, Demirci Z, Öztürkmen Y. Minimally invasive medial plate osteosynthesis in tibial pilon fractures: Longterm functional and radiological outcomes. Acta orthopaedica et traumatologica turcica. 2020;54(1):20-6.
[97] O'Halloran K, Coale M, Costales T, et al. Will My Tibial Fracture Heal? Predicting Nonunion at the Time of Definitive Fixation Based on Commonly Available Variables. Clin Orthop Relat Res. 2016;474(6):1385-95.
[98] Naumann MG, Sigurdsen U, Utvåg SE, Stavem K. Functional outcomes following surgical-site infections after operative fixation of closed ankle fractures. Foot and Ankle Surgery. 2017;23(4):311-6.
[99] Kawasaki N, Takegami Y. Prediction of delayed union of tibial shaft fracture treated with intramedullary nailing: multicenter-study analysis and literature review -the TRON study. 2021.
[100] Ahmed U, Khalid Z, Hassan M, Ullah UK. Outcome of open fractures of Tibia treated with interlocking & intermedullary nail in terms of infection and union. Medical Forum Monthly. 2020;31(12):90-3.

[101] Cammas C, Ancion A, Detrembleur C, Tribak K, Putineanu D, Cornu O. Frequency and risk factors of complications after surgical treatment of ankle fractures : a retrospective study of 433 patients. Acta Orthop Belg. 2020;86(3):563-74.

[102] Halonen LM, Stenroos A, Vasara H, Huotari K, Kosola J. Infections after intramedullary fixation of trochanteric fractures are uncommon and implant removal is not usually needed. Injury. 2021;52(6):1511-6.

[103] Goudie EB, Robinson CM. Prediction of Nonunion After Nonoperative Treatment of a Proximal Humeral Fracture. J Bone Joint Surg Am. 2021;103(8):668-80.

[104] Glassman SD, Anagnost Sc Fau - Parker A, Parker A Fau - Burke D, Burke D Fau - Johnson JR, Johnson Jr Fau - Dimar JR, Dimar JR. The effect of cigarette smoking and smoking cessation on spinal fusion. 2000(0362-2436 (Print)).

[105] Sørensen LT, Hemmingsen U, Jørgensen T. Strategies of smoking cessation intervention before hernia surgery--effect on perioperative smoking behavior. Hernia. 2007;11(4):327-33.

[106] Delaunay F, Coquerel-Beghin D, Magalon G, et al. A preoperative cotininury test for abdominoplasty reduces peri-operative complications. Ann Chir Plast Esthet. 2018;63(4):307-15.

[107] Padubidri AN, Yetman R, Browne E, et al. Complications of postmastectomy breast reconstructions in smokers, ex-smokers, and nonsmokers. Plast Reconstr Surg. 2001;107(2):342-9; discussion 50-1.

[108] Møller AM, Villebro N, Pedersen T, Tønnesen H. Effect of preoperative smoking intervention on postoperative complications: a randomised clinical trial. Lancet. 2002;359(9301):114-7.

[109] Araco A, Gravante G, Sorge R, Araco F, Delogu D, Cervelli V. Wound infections in aesthetic abdominoplasties: The role of smoking. Plastic and Reconstructive Surgery. 2008;121(5):305e-10e.

[110] Chan LK, Withey S, Butler PE. Smoking and wound healing problems in reduction mammaplasty: is the introduction of urine nicotine testing justified? Ann Plast Surg. 2006;56(2):111-5.

[111] Gravante G, Araco A, Sorge R, Araco F, Delogu D, Cervelli V. Wound infections in post-bariatric patients undergoing body contouring abdominoplasty: The role of smoking. Obesity Surgery. 2007;17(10):1325-31.

[112] Gravante G, Araco A, Sorge R, et al. Postoperative wound infections after breast reductions: The role of smoking and the amount of tissue removed. Aesthetic Plastic Surgery. 2008;32(1):25-31.

[113] Herrero C, Tang A, Wasterlain A, et al. Smoking cessation correlates with a decrease in infection rates following total joint arthroplasty. J Orthop. 2020;21:390-4.

[114] Jung KH, Kim SM, Choi MG, et al. Preoperative smoking cessation can reduce postoperative complications in gastric cancer surgery. Gastric Cancer. 2015;18(4):683-90.

[115] Kuri M, Nakagawa M, Tanaka H, Hasuo S, Kishi Y. Determination of the duration of preoperative smoking cessation to improve wound healing after head and neck surgery. Anesthesiology. 2005;102(5):892-6.

[116] Quan H, Ouyang L, Zhou H, Ouyang Y, Xiao H. The effect of preoperative smoking cessation and smoking dose on postoperative complications following radical gastrectomy for gastric cancer: A retrospective study of 2469 patients. World Journal of Surgical Oncology. 2019;17(1).

[117] Sørensen LT, Jørgensen T. Short-term pre-operative smoking cessation intervention does not affect postoperative complications in colorectal surgery: a randomized clinical trial. Colorectal Dis. 2003;5(4):347-52.

[118] Thomsen T, Tønnesen H, Okholm M, et al. Brief smoking cessation intervention in relation to breast cancer surgery: a randomized controlled trial. Nicotine Tob Res. 2010;12(11):1118-24.

[119] Yoshida N, Baba Y, Hiyoshi Y, et al. Duration of Smoking Cessation and Postoperative Morbidity After Esophagectomy for Esophageal Cancer: How Long Should Patients Stop Smoking Before Surgery? World journal of surgery. 2016;40(1):142-7.

[120] Sorensen LT, Karlsmark T, Gottrup F. Abstinence from smoking reduces incisional wound infection: a randomized controlled trial. Annals of surgery. 2003;238(1):1-5.

[121] Sørensen LT, Toft BG, Rygaard J, et al. Effect of smoking, smoking cessation, and nicotine patch on wound dimension, vitamin C, and systemic markers of collagen metabolism. Surgery. 2010;148(5):982-90.

[122] Lindström D, Sadr Azodi O, Wladis A, et al. Effects of a perioperative smoking cessation intervention on postoperative complications: a randomized trial. Ann Surg. 2008;248(5):739-45.

[123] Adams CI, Keating JF, Court-Brown CM. Cigarette smoking and open tibial fractures. Injury. 2001;32(1):61-5.

[124] Dinah AF, Vickers RH. Smoking increases failure rate of operation for established non-union of the scaphoid bone. International Orthopaedics. 2007;31(4):503-5.

[125] Jin PF, Wang ZQ, Song HP. Rist factors for nonunion of closed tibial shaft fractures. J North China Univ Sci Technol (Health Sciences Edition). 2018;20(5):378–83.

[126] Little CP, Burston BJ, Hopkinson-Woolley J, Burge P. Failure of surgery for scaphoid non-union is associated with smoking. Journal of Hand Surgery. 2006;31(3):252-5.

[127] McKee MD, DiPasquale DJ, Wild LM, Stephen DJG, Kreder HJ, Schemitsch EH. The effect of smoking on clinical outcome and complication rates following Ilizarov reconstruction. Journal of Orthopaedic Trauma. 2003;17(10):663-7.

[128] Zura R, Watson JT, Einhorn T, et al. An inception cohort analysis to predict nonunion in tibia and 17 other fracture locations. Injury. 2017;48(6):1194-203.

[129] Tay WH, De Steiger R, Richardson M, Gruen R, Balogh ZJ. Health outcomes of delayed union and nonunion of femoral and tibial shaft fractures. Injury. 2014;45(10):1653-8.

[130] Olsen LL, Møller AM, Brorson S, Hasselager RB, Sort R. The impact of lifestyle risk factors on the rate of infection after surgery for a fracture of the ankle. Bone and Joint Journal. 2017;99-B(2):225-30.

[131] Castillo RC, Bosse MJ, MacKenzie EJ, et al. Impact of smoking on fracture healing and risk

of complications in limb-threatening open tibia fractures. Journal of orthopaedic trauma. 2005;19(3):151-7.

[132] Harvey EJ, Agel J, Selznick HS, Chapman JR, Henley MB. Deleterious effect of smoking on healing of open tibia-shaft fractures. American journal of orthopedics (Belle Mead, NJ). 2002;31(9):518-21.

[133] Hernigou J, Schuind F. Smoking as a predictor of negative outcome in diaphyseal fracture healing. International Orthopaedics. 2013;37(5):883-7.

[134] Ristiniemi J, Flinkkilä T, Hyvönen P, et al. Two-ring hybrid external fixation of distal tibial fractures: A review of 47 cases. Journal of Trauma - Injury, Infection and Critical Care. 2007;62(1):174-83.

[135] Ruffolo MR, Gettys FK, Montijo HE, Seymour RB, Karunakar MA. Complications of high-energy bicondylar tibial plateau fractures treated with dual plating through 2 incisions. Journal of Orthopaedic Trauma. 2015;29(2):85-90.

[136] Schmitz MA, Finnegan M, Natarajan R, Chainpine J. Effect of smoking on tibial shaft fracture healing. Clinical Orthopaedics and Related Research. 1999(365):184-200.

[137] Nåsell H, Ottosson C, Törnqvist H, Lindé J, Ponzer S. The impact of smoking on complications after operatively treated ankle fractures-a follow-up study of 906 patients. Journal of Orthopaedic Trauma. 2011;25(12):748-55.

[138] Lack WD, Starman JS, Seymour R, et al. Any Cortical Bridging Predicts Healing of Tibial Shaft Fractures. J Bone Joint Surg Am. 2014;96(13):1066-72.

[139] Matson AP, Hamid KS, Adams SB. Predictors of Time to Union After Operative Fixation of Closed Ankle Fractures. Foot & ankle specialist. 2017;10(4):308-14.

[140] Penn-Barwell JG, Bennett PM, Mortiboy DE, Fries CA, Groom AF, Sargeant ID. Factors influencing infection in 10 years of battlefield open tibia fractures. Strategies Trauma Limb Reconstr. 2016;11(1):13-8.

[141] Lawing CR, Lin FC, Dahners LE. Local Injection of Aminoglycosides for Prophylaxis Against Infection in Open Fractures. J Bone Joint Surg Am. 2015;97(22):1844-51.

[142] Molina CS, Stinner DJ, Fras AR, Evans JM. Risk factors of deep infection in operatively treated pilon fractures (AO/OTA: 43). Journal of Orthopaedics. 2015;12:S7-S13.

[143] Zhang D, Freemantle N, Cheng KK. Are randomized trials conducted in China or India biased? A comparative empirical analysis. J Clin Epidemiol. 2011;64(1):90-5.

[144] Sørensen LT. Wound healing and infection in surgery. The clinical impact of smoking and smoking cessation. A systematic review and meta-analysis. Wound Repair and Regeneration. 2012;20(5):A113.

[145] Wong J, Lam DP, Abrishami A, Chan MT, Chung F. Short-term preoperative smoking cessation and postoperative complications: a systematic review and meta-analysis. Canadian journal of anaesthesia = Journal canadien d'anesthesie. 2012;59(3):268-79.
